# A World Malaria Map: Plasmodium falciparum Endemicity in 2007

**DOI:** 10.1371/journal.pmed.1000048

**Published:** 2009-03-24

**Authors:** Simon I Hay, Carlos A Guerra, Peter W Gething, Anand P Patil, Andrew J Tatem, Abdisalan M Noor, Caroline W Kabaria, Bui H Manh, Iqbal R. F Elyazar, Simon Brooker, David L Smith, Rana A Moyeed, Robert W Snow

**Affiliations:** 1 Malaria Public Health and Epidemiology Group, Centre for Geographic Medicine, Kenya Medical Research Institute (KEMRI)–University of Oxford–Wellcome Trust Collaborative Programme, Nairobi, Kenya; 2 Spatial Ecology and Epidemiology Group, Department of Zoology, University of Oxford, Oxford, United Kingdom; 3 Centre for Geographical Health Research, School of Geography, University of Southampton, Highfield, Southampton, United Kingdom; 4 Department of Geography, University of Florida, Gainesville, Florida, United States of America; 5 Emerging Pathogens Institute, University of Florida, Gainesville, Florida, United States of America; 6 Centre for Tropical Medicine, Nuffield Department of Clinical Medicine, University of Oxford, Centre for Clinical Vaccinology and Tropical Medicine (CCVTM), Oxford, United Kingdom; 7 Oxford University Clinical Research Unit, Bach Mai Hospital, National Institute of Infectious and Tropical Diseases, Ha Noi, Vietnam; 8 Eijkman-Oxford Clinical Research Unit, Jakarta, Indonesia; 9 Department of Infectious and Tropical Diseases, London School of Hygiene and Tropical Medicine, United Kingdom; 10 Department of Zoology, University of Florida, Gainesville, Florida, United States of America; 11 School of Mathematics and Statistics, University of Plymouth, Plymouth, Devon, United Kingdom; Papua New Guinea Institute of Medical Research, Papua New Guinea

## Abstract

**Background:**

Efficient allocation of resources to intervene against malaria requires a detailed understanding of the contemporary spatial distribution of malaria risk. It is exactly 40 y since the last global map of malaria endemicity was published. This paper describes the generation of a new world map of Plasmodium falciparum malaria endemicity for the year 2007.

**Methods and Findings:**

A total of 8,938 P. falciparum parasite rate (*Pf*PR) surveys were identified using a variety of exhaustive search strategies. Of these, 7,953 passed strict data fidelity tests for inclusion into a global database of *Pf*PR data, age-standardized to 2–10 y for endemicity mapping. A model-based geostatistical procedure was used to create a continuous surface of malaria endemicity within previously defined stable spatial limits of P. falciparum transmission. These procedures were implemented within a Bayesian statistical framework so that the uncertainty of these predictions could be evaluated robustly. The uncertainty was expressed as the probability of predicting correctly one of three endemicity classes; previously stratified to be an informative guide for malaria control. Population at risk estimates, adjusted for the transmission modifying effects of urbanization in Africa, were then derived with reference to human population surfaces in 2007. Of the 1.38 billion people at risk of stable P. falciparum malaria, 0.69 billion were found in Central and South East Asia (CSE Asia), 0.66 billion in Africa, Yemen, and Saudi Arabia (Africa+), and 0.04 billion in the Americas. All those exposed to stable risk in the Americas were in the lowest endemicity class (*Pf*PR_2−10_ ≤ 5%). The vast majority (88%) of those living under stable risk in CSE Asia were also in this low endemicity class; a small remainder (11%) were in the intermediate endemicity class (*Pf*PR_2−10_ > 5 to < 40%); and the remaining fraction (1%) in high endemicity (*Pf*PR_2−10_ ≥ 40%) areas. High endemicity was widespread in the Africa+ region, where 0.35 billion people are at this level of risk. Most of the rest live at intermediate risk (0.20 billion), with a smaller number (0.11 billion) at low stable risk.

**Conclusions:**

High levels of P. falciparum malaria endemicity are common in Africa. Uniformly low endemic levels are found in the Americas. Low endemicity is also widespread in CSE Asia, but pockets of intermediate and very rarely high transmission remain. There are therefore significant opportunities for malaria control in Africa and for malaria elimination elsewhere. This 2007 global P. falciparum malaria endemicity map is the first of a series with which it will be possible to monitor and evaluate the progress of this intervention process.

## Abstract 2

Incorporating data from nearly 8,000 surveys of*Plasmodium falciparum* parasite rates, Simon Hay and colleagues employ a model-based geostatistical procedure to create a map of global malaria endemicity.

## Editors' Summary

**Background.:**

Malaria is one of the most common infectious diseases in the world and one of the greatest global public health problems. The Plasmodium falciparum parasite causes approximately 500 million cases each year and over one million deaths in sub-Saharan Africa. More than 40% of the world's population is at risk of malaria. The parasite is transmitted to people through the bites of infected mosquitoes. These insects inject a life stage of the parasite called sporozoites, which invade human liver cells where they reproduce briefly. The liver cells then release merozoites (another life stage of the parasite), which invade red blood cells. Here, they multiply again before bursting out and infecting more red blood cells, causing fever and damaging vital organs. The infected red blood cells also release gametocytes, which infect mosquitoes when they take a blood meal. In the mosquito, the gametocytes multiply and develop into sporozoites, thus completing the parasite's life cycle. Malaria can be prevented by controlling the mosquitoes that spread the parasite and by avoiding mosquito bites by sleeping under insecticide-treated bed nets. Effective treatment with antimalarial drugs also helps to decrease malaria transmission.

**Why Was This Study Done?:**

In 1998, the World Health Organization and several other international agencies launched Roll Back Malaria, a global partnership that aims to reduce the human and socioeconomic costs of malaria. Targets have been continually raised since this time and have culminated in the Roll Back Malaria Global Malaria Action Plan of 2008, where universal coverage of locally appropriate interventions is called for by 2010 and the long-term goal of malaria eradication again tabled for the international community. For malaria control and elimination initiatives to be effective, financial resources must be concentrated in regions where they will have the most impact, so it is essential to have up-to-date and accurate maps to guide effort and expenditure. In 2008, researchers of the Malaria Atlas Project constructed a map that stratified the world into three levels of malaria risk: no risk, unstable transmission risk (occasional focal outbreaks), and stable transmission risk (endemic areas where the disease is always present). Now, researchers extend this work by describing a new evidence-based method for generating continuous maps of P. falciparum endemicity within the area of stable malaria risk over the entire world's surface. They then use this method to produce a P. falciparum endemicity map for 2007. Endemicity is important as it is a guide to the level of morbidity and mortality a population will suffer, as well as the intensity of the interventions that that will be required to bring the disease under control or additionally to interrupt transmission.

**What Did the Researchers Do and Find?:**

The researchers identified nearly 8,000 surveys of P. falciparum parasite rates (*Pf*PR; the percentage of a population with parasites detectable in their blood) completed since 1985 that met predefined criteria for inclusion into a global database of *Pf*PR data. They then used “model-based geostatistics” to build a world map of P. falciparum endemicity for 2007 that took into account where and, importantly, when and all these surveys were done. Predictions were comprehensive (for every area of stable transmission globally) and continuous (predicted as a endemicity value between 0% and 100%). The population at risk of three levels of malaria endemicity were identified to help summarize these findings: low endemicity, where *Pf*PR is below 5% and where it should be technically feasible to eliminate malaria; intermediate endemicity where *Pf*PR is between 5% and 40% and it should be theoretically possible to interrupt transmission with the universal coverage of bed nets; high endemicity is where *Pf*PR is above 40% and suites of locally appropriate intervention will be needed to bring malaria under control. The global level of malaria endemicity is much reduced when compared with historical maps. Nevertheless, the resulting map indicates that in 2007 almost 60% of the 2.4 billion people at malaria risk were living in areas with a stable risk of P. falciparum transmission—0.69 billion people in Central and South East Asia (CSE Asia), 0.66 billion in Africa, Yemen, and Saudi Arabia (Africa+), and 0.04 billion in the Americas. The people of the Americas were all in the low endemicity class. Although most people exposed to stable risk in CSE Asia were also in the low endemicity class (88%), 11% were in the intermediate class, and 1% were in the high endemicity class. By contrast, high endemicity was most common and widespread in the Africa+ region (53%), but with significant numbers in the intermediate (30%), and low (17%) endemicity classes.

**What Do These Findings Mean?:**

The accuracy of this new world map of P. falciparum endemicity depends on the assumptions made in its construction and critically on the accuracy of the data fed into it, but because of the statistical methods used to construct this map, it is possible to quantify the uncertainty in the results for all users. Thus, this map (which, together with the data used in its construction, will be freely available) represents an important new resource that clearly indicates areas where malaria control can be improved (for example, Africa) and other areas where malaria elimination may be technically possible. In addition, planned annual updates of the global P. falciparum endemicity map and the *Pf*PR database by the Malaria Atlas Project will help public-health experts to monitor the progress of the malaria control community towards international control and elimination targets.

**Additional Information.:**

Please access these Web sites via the online version of this summary at http://dx.doi.org/10.1371/journal.pmed.1000048.

- A *PLoS Medicine* Health in Action article (Hay SI, Snow RW (2006) The Malaria Atlas Project: Developing Global Maps of Malaria Risk. *PLoS Med* 3(12): e473) and a Research Article (Guerra CA, Gikandi PW, Tatem AJ, Noor AM, Smith DL, et al. (2008) The Limits and Intensity of *Plasmodium falciparum* Transmission: Implications for Malaria Control and Elimination Worldwide. *PLoS Med* 5(2): e38) also provide further details about the global mapping of malaria risk, and a further Research Article (Snow RW, Guerra CA, Mutheu JJ, Hay SI (2008) International Funding for Malaria Control in Relation to Populations at Risk of Stable *Plasmodium falciparum* Transmission. *PLoS Med* 5(7): e142) discusses the financing of malaria control in relation to this risk
- Additional national and regional level maps and more information on the global mapping of malaria are available at the Malaria Atlas Project
- The MedlinePlus encyclopedia contains a page on malaria (in English and Spanish)
- Information is available from the World Health Organization on malaria (in several languages)
- The US Centers for Disease Control and Prevention provide information on malaria (in English and Spanish)
- Information is available from the Roll Back Malaria Partnership on its approach to the global control of malaria, and on malaria control efforts in specific parts of the world

## Introduction

Maps are essential for all aspects of the coordination of malaria control [1]. In an international policy environment where the malaria control community has been challenged to rethink the plausibility of malaria elimination [2–4], malaria cartography will become an increasingly important tool for planning, implementing, and measuring the impact of malaria interventions worldwide. The last global map of P. falciparum endemicity was published in 1968 [5]. In common with all previous maps of the global distribution of malaria [6–10], and to a large extent those that followed [11–16], the map (i) suffered from an incomplete description of the input data used; (ii) defined contours of “risk” using subjective and poorly explained expert-opinion rules; and (iii) provided no quantification of the uncertainty around predictions. Here we describe the generation of a new global map of malaria endemicity that overcomes these major deficiencies.

### Geographic Scope of the Modelling

**Figure 1:**
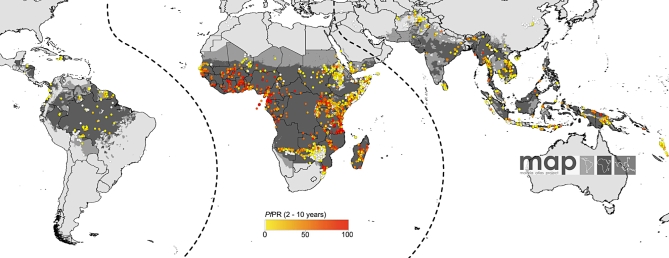
The Spatial Limits of P. falciparum Malaria Risk Defined by *Pf*API with Further Medical Intelligence, Temperature, and Aridity Masks Areas were defined as stable (dark grey areas, where *Pf*API ≥ 0.1 per 1,000 pa), unstable (medium grey areas, where *Pf*API < 0.1 per 1,000 pa), or no risk (light grey, where *Pf*API = 0 per 1,000 pa) [17–19]. The community surveys of P. falciparum prevalence conducted between January 1, 1985 and July 31, 2008 are plotted. Of the 8,938 surveys collected, 7,953 satisfied our inclusion criteria for modelling (see Methods and Protocol S1.2) and are shown here. The survey data shown are age-standardized [51] (*Pf*PR_2−10_) and shown as a continuum of yellow to red from 0%–100% (see map legend). The dashed lines separate the America; Africa+; and the CSE Asia regions.

The global spatial limits of P. falciparum malaria transmission have been mapped recently by triangulating nationally reported case incidence data, other medical intelligence, and biological rules of transmission exclusion, derived from temperature and aridity limits to the bionomics of locally dominant *Anopheles* vectors [17,18]. The results of this exercise stratified the world into three classes: the spatial representation of no risk, unstable risk (P. falciparum annual parasite incidence [*Pf*API] < 0.1 per 1,000 people per annum [pa]), and stable risk (*Pf*API ≥ 0.1 per 1,000 people pa) of P. falciparum transmission for 2007. These classes are shown in Figure 1. The stable-unstable classification of *Pf*API was based on a review of the statistical, logistical, programmatic, and pragmatic reasons underpinning the *Pf*API levels used to define action points during the global malaria eradication campaign [19–21].

The mapping exercise described here extends this work substantively. The largest ever global assembly of malariometric surveys is used to predict P. falciparum malaria prevalence values at every point within the stable spatial limits of transmission to make a continuous P. falciparum endemicity surface. To facilitate this process the spatial limits required majority resampling to a 5 × 5 km grid using ArcView GIS 3.2 (ESRI, 1999) because the computer-intensive mapping techniques adopted, and described next, could not be implemented at 1 × 1 km spatial resolution at a global scale.

### Recent Approaches to Malaria Endemicity Mapping

Numerous approaches exist for the production of continuous endemicity maps using data from malariometric surveys, all of which require the use of a model to predict endemicity values at locations where survey data are unavailable [22–26]. The maps resulting from such models have an inherent uncertainty and its quantification is a primary concern in disease mapping.

A number of recent studies have adopted a predictive framework known as model-based geostatistics (MBG) [27] for the spatial prediction of malaria endemicity [28–33] and the prevalence of other vector-borne and intermediate host-borne diseases [34–38]. MBG provides a formal statistical interpretation of classical geostatistical tools for spatial prediction [39–41] and allows the incorporation of Bayesian methods of statistical inference [42,43]. The principal advantage of MBG for disease mapping is the rigorous handling of uncertainty introduced at different stages in the modelling process [27]. By modelling the interaction of these different sources of uncertainty, a probability distribution is generated for each predicted location, which can be summarised to provide robust metrics of confidence around predicted values. This resulting map will therefore provide an evidence-based contemporary benchmark of global malaria endemicity, using MBG techniques to assess the confidence in the predictions, and provide those who utilize the map a clear estimate of the fidelity of the predictions [36].

An underlying principle of geostatistics is that a mapped prediction becomes increasingly uncertain as the density of and proximity to nearby data points decreases. When data are collected at different times, as well as different locations, this principle is as applicable through time as it is across space. Examples of epidemiological studies that extend spatial geostatistics to incorporate time are rare [44–47], but in this study a full spatiotemporal geostatistical modelling framework was developed. Incorporating the dimension of time allows for unambiguous comparison of this benchmark with future map iterations. The map will thus provide an explicit geographical framework for monitoring and evaluation of the impact of the malaria control community on P. falciparum malaria worldwide.

## Methods

### Analysis Outline

The objective of these analyses is to use a contemporary database of P. falciparum parasite rate (*Pf*PR) surveys to make a continuous, global, P. falciparum malaria endemicity surface for 2007, implemented with transparent and reproducible methods, and which documents robustly the uncertainty associated with its predictions.

**Figure 2:**
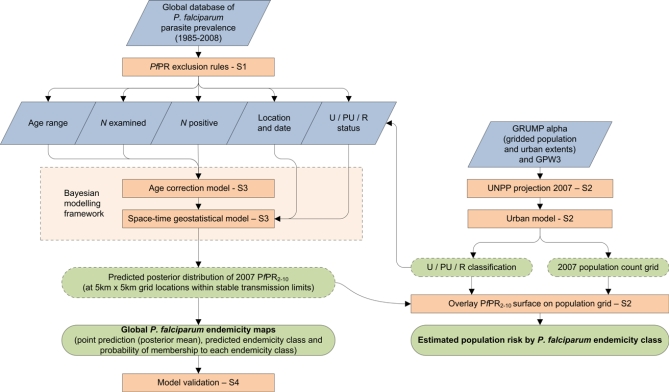
Schematic Overview of the Mapping Procedures and Methods Blue diamonds describe input data. Orange boxes denote models and experimental procedures; S1, Protocol S1; S2, Protocol S2; S3, Protocol S3; and S4, Protocol S4. Green rods indicate output data; dashed lines intermediate output, solid lines final outputs. U, urban; PU, peri-urban; and R, rural extents.

The main steps used to define the continuous global map of P. falciparum prevalence within our analytic framework are outlined in Figure 2. First, it was necessary to search for and preprocess the *Pf*PR data in order to create a robustly geo-positioned, geographically extensive dataset of malariometric surveys for mapping and examine potential environmental covariates (Protocol S1) and the influence of human settlement patterns (Protocol S2) [48–50]. Second, the refined *Pf*PR database was used to make a continuous, age-standardized and urban-corrected malaria prevalence surface with MBG in a Bayesian statistical framework (Protocol S3). Third, extensive validation procedures were implemented to assess the accuracy of endemicity predictions and uncertainty metrics (Protocol S4). Finally, populations at risk (PAR) of P. falciparum malaria estimates were extracted globally and presented at the regional level, stratified by age class.

### Assembling a Global Database of P. falciparum Parasite Prevalence

Of all the potential metrics available to measure malaria endemicity, the parasite rate (the proportion of people sampled showing detectable parasites in the peripheral blood) was preferred as a basis for mapping, due to its global ubiquity [18] and its sensitivity across a wide range of the P. falciparum malaria transmission spectrum [19]. A categorization of the malaria endemicity spectrum in the epidemiologically informative 2 (2.00)- up to 10 (9.99)-y age group has been suggested [51], guided by the potential impact on malaria endemicity using the most widely deployed contemporary malaria intervention—insecticide treated bed nets (ITNs) [19]. The lowest class of *Pf*PR in the 2- up to 10-y age group (hereafter *Pf*PR_2−10_), corresponds to ≤5%. This is the point below which *Pf*PR surveys require sample sizes of the population that become prohibitive logistically, to measuring endemicity accurately and surveillance-based malariometrics are therefore favoured [52–54]. We regard intermediate, stable transmission as represented by *Pf*PR_2−10_ > 5% to < 40%, since a range of mathematical models predict that the interruption of malaria transmission could be achieved with universal coverage of ITNs in all areas with *Pf*PR_2−10_ < 40% [19,55]. Despite being subject to some uncertainty owing to the behaviour and bionomics of the dominant local *Anopheles* vectors [56], the *Pf*PR_2−10_ < 40% level is considered a conservative benchmark, since ITNs are rarely deployed independently of other interventions that will further reduce transmission. The areas of high stable transmission, where mixed intervention suites need to be considered if the interruption of transmission is ever to be achieved, are identified as all prevalences above this level: *Pf*PR_2−10_ ≥ 40%. This malaria classification is used to guide the interpretation of the predicted endemicity surface throughout and is a departure from traditional endemicity benchmarks [57] that have been shown not to scale meaningfully with opportunities for control and elimination in most models [19,55].

The process of identifying, assembling, and geo-locating community-based survey estimates of parasite prevalence undertaken since 1985 has been described [58]. Searches for *Pf*PR data are an ongoing activity of the Malaria Atlas Project (MAP, http://www.map.ox.ac.uk) and were completed on July 31, 2008 for this 2007 iteration of the global endemicity map (Protocol S1.1). A total of 8,938 cross-sectional survey estimates of *Pf*PR were assembled from 78 of the 87 P. falciparum malaria endemic countries (*Pf*MECs) [18]. Those countries not represented in the database were Bangladesh, Belize, Bhutan, Djibouti, Dominican Republic, Guyana, Iran, Kyrgyzstan, and Panama.

After six levels of exclusion (removing surveys located only to large [>100 km^2^] and small [>25 km^2^] polygons [58]; removing those surveys that could not be, or were only geo-positioned imprecisely; and removing those that could not be temporally disaggregated into independent surveys or for which the date was unknown), 7,991 *Pf*PR surveys remained (Figure S1.2 in Protocol S1).

All *Pf*PR data were then age-standardized to the 2- to 10-y age range before mapping using an algorithm based on catalytic conversion models first adapted for malaria by Pull and Grab [59]. This algorithm was found to perform best out of a set of candidate standardization procedures and is described in detail elsewhere (Protocol S1.3) [51].

The final dataset was stratified into three major global regions (Figure 1): the Americas; Africa, Yemen, and Saudi Arabia (Africa+); and Central and South and East Asia (CSE Asia) (Protocol S1.4). This division allowed these biogeographically, entomologically, and epidemiologically distinct regions [8,16] to be considered separately, whilst retaining sufficient data in each region for meaningful analysis. These global divisions were further supported by observing the distinct spatial structure of the *Pf*PR_2−10_ data in each region, illustrated by their semi-variograms (Figure S1.1 in Protocol S1).

Malaria transmission-specific approaches to mapping urban, peri-urban, and rural extents were developed, the rationale for which is described in detail elsewhere (Protocol S2) [50]. In brief, all urban extents (UEs) defined by the Global Rural Urban Mapping Project (GRUMP) alpha version UE mask (GRUMP UE) [60,61] were identified at 1 × 1 km spatial resolution (Protocol S2.1) [50]. Within these extents, those areas containing population densities greater than 1,000 people per km^2^ according to the Gridded Population of the World version 3 population density surface [60,61] were then mapped [48]. All surveys were then assigned as either urban (Gridded Population of the World version 3 ≥ 1,000 km^2^ within GRUMP UE), peri-urban (Gridded Population of the World version 3 < 1,000 km^2^ within GRUMP UE), or rural (outside GRUMP UE) (Protocol S2.2).

Extreme statistical outliers in the rural *Pf*PR_2−10_ data were then identified using a geostatistical filter (Protocol S1.5). This process used semi-variogram statistics to assess whether each point differed significantly from neighbouring points given their separation distances and regional patterns of spatial variation. This procedure identified 38 nonurban *Pf*PR_2−10_ records, which were removed from the dataset before further modelling. Details of these surveys are available on request.

The final set of *Pf*PR_2−10_ data (*n =* 7,953) used is shown in Figure 1. The attributes of this *Pf*PR_2−10_ database are described (Table S1.2 in Protocol S1), along with a plot of the median *Pf*PR_2−10_ by year for the observation period (Figure S1.3 in Protocol S1), indicating that time was an important source of variation to include in the MBG model. Similar preliminary explorations of the relationships of these data with a range of climate [62] and remotely sensed [63] environmental covariates showed no strong relationships (Figure S1.5 and S1.6 in Protocol S1), supporting the predominantly univariate approach to the analyses.

There is a common misconception that malariometric surveys are only conducted in areas of high prevalence. In fact, an increasing tendency to conduct national surveys powered to be representative of all regions of a country, and the confirmation of the absence of P. falciparum transmission when sampling for *P. vivax,* result in many zero prevalence values being recorded in surveys. In total, 119 of 261 surveys report zero values in America, 1,010 of 5,307 surveys report zero values in Africa+, and 775 of 2,385 surveys report zero values in the CSE Asia region (Figure 1).

### Statistical Details

Geostatistical algorithms generate continuous maps by predicting values at unsampled locations using linear combinations of the available sample data. In the mapping task described in this study, it is intuitive that the confidence attached to a prediction of *Pf*PR_2−10_ at a given unsampled location will be affected by (i) the distribution of survey points around that location (the spatial density of the training data), (ii) the extent to which *Pf*PR_2−10_ varies smoothly across space (the spatial heterogeneity of the training data), and (iii) the number of people sampled in each survey (the precision of the component surveys in the training data). An MBG approach [27] was implemented in a Bayesian statistical framework to incorporate these factors in the generation of continuous maps of *Pf*PR_2−10_ (Protocol S3). Because the data were collected at different times throughout the study period 1985–2008, it was important to extend the spatial-only geostatistical approach to a space-time framework that accounted simultaneously for the density and heterogeneity of the data in both space and time. The age-standardization algorithm was incorporated as a submodel in the framework to allow the errors inherent in this process to be estimated and propagated into the MBG stage (Protocol S3).

For each region, a Bayesian geostatistical model was constructed in which the underlying value of *Pf*PR_2−10_ in 2007, *Pf*PR_2−10_(*x*_i_), at each location *x*_i_ was modelled as a transformation *g*(·) of a spatiotemporally structured field superimposed with unstructured (random) variation ɛ(*x*_i_). The number of P. falciparum positive responses *N*_i_^+^ from a total sample of *N*_i_ at each survey location was modelled as a conditionally independent binomial variate given the unobserved underlying age-standardized *Pf*PR_2−10_ value [36]. The spatiotemporal component was represented by a stationary Gaussian process *f*(*x*_i_,*t*_i_) with mean *μ* and covariance defined by a spatially anisotropic version of the space-time covariance function proposed by Stein [64]. A modification was made to the Stein covariance function to allow the time-marginal model to include a periodic component of wavelength 12 mo, providing the capability to model seasonal effects in the observed temporal covariance structure. These effects arise when studies performed in different years but during similar calendar months have a tendency to be more similar to each other than would be expected in the absence of seasonality. The mean component *μ* was modelled as a linear function of time *t* and whether the prediction location *x* was urban, or peri-urban (denoted by the indicator variables 1*_u_*(*x*) and 1*_p_*(*x*), respectively) rather than rural: *μ* = *β_x_* + *β_t_t* + *β_u_*1*_u_*(*x*) + *β_p_*1*_p_*(*x*). Each survey was referenced temporally using the mid-point (in decimal years) between the recorded start and end months. Urban, peri-urban, or rural status was assigned to each prediction location using the modified GRUMP UE surface described previously (Protocol S2.2), resampled to a 5 × 5 km grid. The unstructured component ɛ(*x*_i_) was represented as Gaussian with zero mean and variance *V*. Bayesian inference was implemented using Markov Chain Monte Carlo (MCMC) to generate samples from the posterior distribution of: the Gaussian field *f*(*x*_i_,*t*_i_) at each data location; the unobserved parameters *β_x_*, *β_t_*, *β_u_*, *β_p_*, and *V* as stated above and further unobserved parameters defining the structure and anisotropy of the exponential space-time covariance function (Protocol S3.4). Distances between locations were computed in great-circle distance to incorporate the effect of the curvature of the Earth, which becomes important at the regional scale. Samples were generated from the 2007 annual mean of the posterior distribution of *f*(*x*_i_,*t*_i_) at each prediction location. For each sample of the joint posterior, predictions were made using space-time conditional simulation over the 12 mo of 2007 (*t* = 2007_Jan_, …, 2007_Dec_} [44,65]. These predictions were made at points on a regular 5 × 5 km spatial grid within the spatial limits of stable P. falciparum transmission. Model output therefore consisted of samples from the predicted posterior distribution of the 2007 annual mean *Pf*PR_2−10_ at each grid location, which were used to generate point estimates (computed as the mean of each set of posterior samples), endemicity class membership probabilities, and standard variance estimates (Protocol S3.4). Further description of how geostatistical outputs were used to generate the various maps described is provided (Protocol S3.5).

### Model Validation

An assessment of the plausibility of the mapped surface was essential and several nontrivial descriptive methods were implemented (Protocol S4). The ability of the model to predict point-values of *Pf*PR_2−10_ and the most probable endemicity class was tested using a hold-out procedure. A validation set was generated by the selection via spatially declustered stratified random sampling of 10% of the data (*n =* 800), which were then removed from the dataset (Protocol S4.1). The model was then run in full using the remaining 7,153 data points to generate predictive posterior distributions of *Pf*PR_2−10_ for comparison with known values at the locations of the 800 held-out data. In contrast to the main model run, in which annual means were predicted for 2007, the validation run predicted *Pf*PR_2−10_ for the month corresponding to the mid-point of each held-out survey, to provide temporally comparable values. Given the large size of the dataset, a single validation set was considered sufficient to generate validation statistics with the required level of precision.

The ability to predict known values of *Pf*PR_2−10_ was summarised using mean error as a measure of overall bias, mean absolute error as a measure of overall accuracy, and the correlation coefficient as a measure of linear association [44,66]. These statistics were presented as both absolute values and as a proportion of the mean *Pf*PR_2−10_ in each region as calculated from the validation set. The ability to predict endemicity class membership was tested using the area-under-curve (AUC) statistic derived from receiver-operating-characteristic curves, which plot sensitivity versus 1-specificity for each endemicity class [34,67]. AUC values above 0.9 indicate excellent agreement between actual and predicted class membership, values above 0.7 indicate a moderately good agreement, and values of 0.5 indicate that the model performs no better than a random allocation of class membership [34,67]. A procedure was also implemented [44,68] to test the extent to which predicted posterior distributions at each prediction location provided a suitable measure of uncertainty. This procedure allowed the probability assigned to predicted values of *Pf*PR_2−10_ at each prediction location to be compared to the corresponding observed probabilities within each region. Further details of this procedure are provided (Protocol S4.2).

Frequency distributions of *Pf*PR_2−10_ were visualised for both input data and the output predicted surface using violin plots [69]. These plots display a smoothed approximation of the frequency distribution (a kernel density plot) of *Pf*PR_2−10_ for each region overlaid on a central bar showing median and inter-quartile range values. Separate plots were computed using age-standardized *Pf*PR_2−10_ data from all years in the database and for 2007 only, and a further plot was computed using point estimates for every location on the predicted output *Pf*PR_2−10_ surface for 2007.

### Estimating Human Population Density in 2007

The GRUMP alpha version provides gridded population counts and population density estimates at 1 × 1 km spatial resolution for the years 1990, 1995, and 2000, both adjusted and unadjusted to the United Nations' national population estimates (Protocol S2.3) [60,61]. The adjusted population counts for the year 2000 were projected to 2007 by applying the relevant national, medium variant, inter-censal growth rates by country [70] using methods described previously (Protocol S2.4) [71]. These population counts were then stratified nationally by age group using United Nations-defined [72] population age structures for the year 2005 to obtain 0–4 years, 5–14 y, and ≥15 y population count surfaces.

Digital boundaries of the 87 P. falciparum malaria endemic countries were overlaid on the urban-adjusted endemicity class surface (reprojected to an equal area projection), and areas of each endemicity class were extracted using ArcView GIS 3.2 (ESRI, 1999) (Protocol S2.4). These layers were also overlaid on the GRUMP data [60,61] to extract urban adjusted estimates of PAR of P. falciparum by endemicity and age class (Protocol S2.4). Finally these surfaces were combined with the uncertainty maps to provide a population-weighted index of uncertainty (the product of the log of population density and the reciprocal of the probability of correct class assignment).

## Results

### Accuracy of the Predicted *P. falciparum* Malaria Endemicity Map

**Figure 3:**
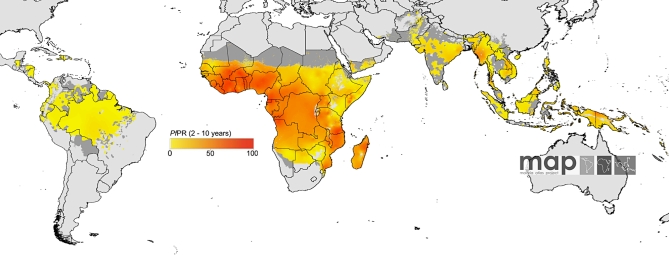
The Spatial Distribution of P. falciparum Malaria Endemicity The data are the model-based geostatistical point estimates of the annual mean *Pf*PR_2−10_ for 2007 within the stable spatial limits of P. falciparum malaria transmission, displayed as a continuum of yellow to red from 0%–100% (see map legend). The rest of the land area was defined as unstable risk (medium grey areas, where *Pf*API < 0.1 per 1,000 pa) or no risk (light grey, where *Pf*API = 0 per 1,000 pa) [17–19].

**Figure 4:**
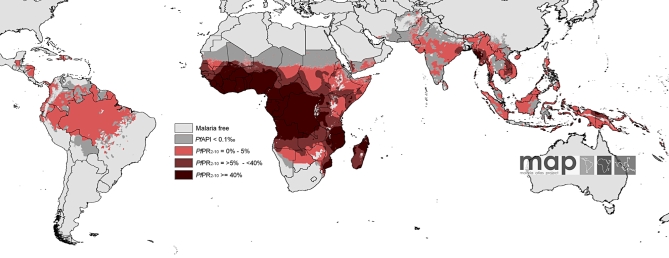
The Spatial Distribution of P. falciparum Malaria *Pf*PR_2−10_ Predictions Stratified by Endemicity Class They are categorized as low risk *Pf*PR_2−10_ ≤ 5%, light red; intermediate risk *Pf*PR_2−10_ > 5% to < 40%, medium red; and high risk *Pf*PR_2−10_ ≥ 40%, dark red. The map shows the class to which *Pf*PR_2−10_ has the highest predicted probability of membership. The rest of the land area was defined as unstable risk (medium grey areas, where *Pf*API < 0.1 per 1,000 pa) or no risk (light grey) [17–19].

**Figure 5:**
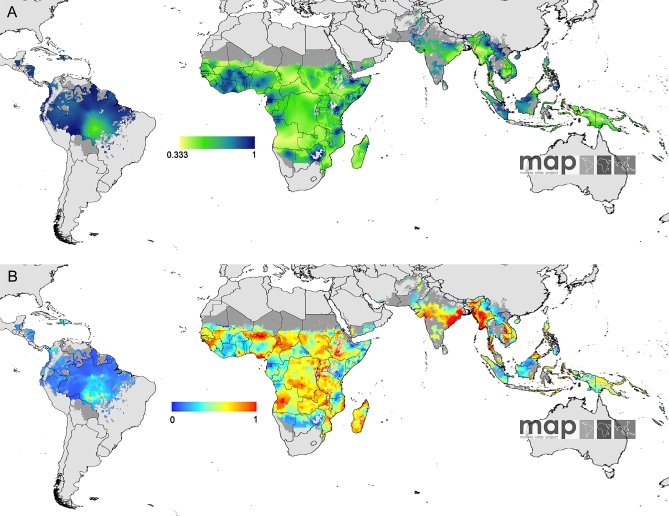
Maps of Model Uncertainty (A) The probability of *Pf*PR_2−10_ being in the class to which it was assigned is mapped and shown as a yellow to blue continuum from 0.3̇ − 1. Any value above 0.3̇ is better than a chance allocation. The rest of the land area was defined as unstable risk (medium grey areas, where *Pf*API < 0.1 per 1,000 pa) or no risk (light grey) [17–19]. (B) The population-weighted index of uncertainty. This index shows the likely importance of uncertainty assessed by the product of the log of population density (Protocol S2.3) and the reciprocal of the probability of correct class assignment, rescaled from 0–1. The index is shown for the most probable *Pf*PR_2−10_ endemicity class. Unstable and no risk are as (A).

The continuous predicted surface of P. falciparum malaria endemicity is shown in Figure 3. The control related endemicity class for which membership is most probable is shown in Figure 4. The actual probability of predicting each class correctly is given in Figure 5A. A detailed description of the regional variation of the area at these different levels of stable risk and the associated PAR, follows a description of the accuracy of the predictions in the text. Alternative measures of the uncertainty of the predictions are provided (Protocol S4.3).

### Predicting Point-Values of *Pf*PR_2−10_

**Figure 6:**
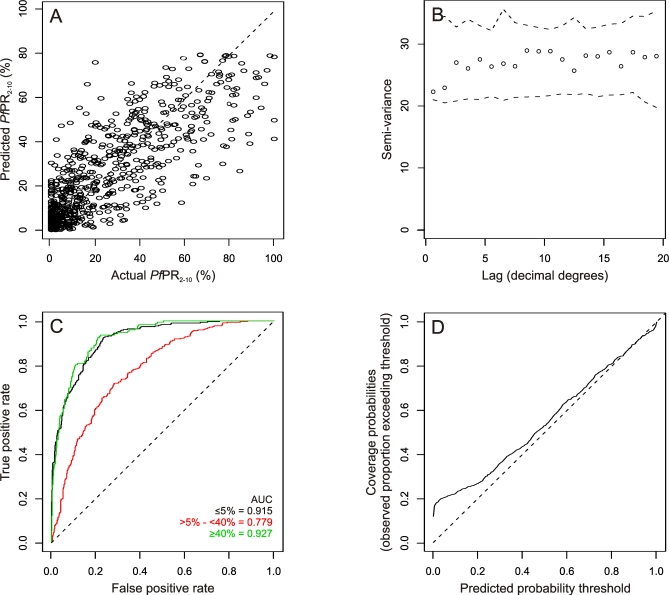
Model Validation Plots (A) Scatter plot of actual versus predicted point-values of *Pf*PR_2−10_. (B) Sample semi-variogram of standardized model Pearson residuals estimated at discrete lags (circles) and compared to a Monte Carlo envelope (dashed lines) representing the range of values expected by chance in the absence of spatial autocorrelation. (C) Receiver-Operating-Characteristic curves for each *Pf*PR_2−10_ endemicity class (black line, *Pf*PR_2−10_ ≤ 5%; red line, *Pf*PR_2−10_ > 5% to < 40%; green line, *Pf*PR_2−10_ ≥ 40%) and associated AUC statistics. (D) Probability-probability plot comparing predicted probability thresholds with the actual percentage of true values exceeding those thresholds. In the top left and bottom left plots the 1:1 line is also shown (dashed line) for reference. See text for full explanation of validation procedures and interpretation of results.

Examination of the mean error in the generation of the P. falciparum malaria endemicity surface (Figure 3) revealed minimal overall bias in predicted *Pf*PR_2−10_ values with a global value of 0.91 revealing an overall tendency to overestimate *Pf*PR_2−10_ by less than 1% (Americas = 0.63, Africa+ = 0.80, CSE Asia = 1.18) (Table 1). Examination of the mean absolute error revealed an average magnitude of error in *Pf*PR_2−10_ predictions of 9.75 (Americas = 3.52, Africa+ = 11.02, CSE Asia = 7.71) (Table 1). The global correlation coefficient between actual and predicted values was 0.82, indicating excellent linear agreement at the global level and this was further illustrated in the scatter plot (Figure 6A; Table 1). The regional level correlations for the Americas and CSE Asia were generally weaker (Americas = 0.03, Africa+ = 0.82, CSE Asia = 0.70) (Table 1). A semi-variogram of standardised model residuals (Figure 6B) showed some evidence of very weak spatial autocorrelation, up to lags of around two decimal degrees, although comparison with a simulated null-envelope revealed that this was not statistically significant (Protocol S4.2).

**Table 1:**
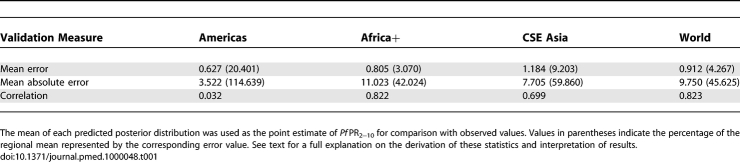
Summary of the Validation Statistics for Predicting Continuous *Pf*PR_2−10_ by Region

### Predicting Endemicity Class

The receiver-operating-characteristic curves and AUC statistics for each endemicity class are shown (Figure 6C; Table 2). Global AUC values for all three endemicity classes exceeded the 0.7 threshold for fair to good discrimination, whilst those for both the *Pf*PR_2−10_ ≤ 5% and *Pf*PR_2−10_ ≥ 40% classes exceeded the 0.9 threshold for excellent discrimination. Overall, 70.8% of points were classified correctly (Americas = 80.0%, Africa+ = 70.6%, CSE Asia = 69.9%) and importantly, only 1.1% of points were grossly misclassified to a nonadjacent class (Americas = 0.0%, Africa+ = 0.6%, CSE Asia = 2.5%) (Table 2). A full contingency table for each class is provided (Protocol S4.3).

**Table 2:**
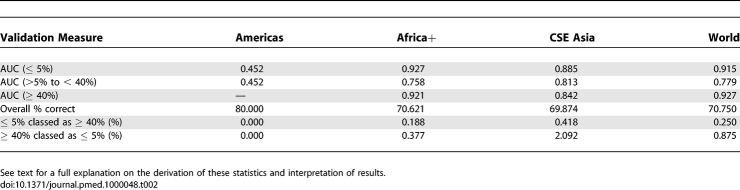
Summary of the Validation Statistics for Predicting *Pf*PR_2−10_ Endemicity Class by Region

### Providing Realistic Measures of Uncertainty for Each Prediction

The probability-probability plot comparing predicted probability thresholds with observed coverage probabilities (Figure 6D) shows generally close correspondence between these two measures, suggesting that the model provides a reasonably faithful representation of the uncertainty in the point predictions. However, the plotted line falls slightly above the 1:1 line across most threshold values, most substantially for probability thresholds between 0.00 and around 0.25. This means that a predicted probability threshold of, for example, 0.1, is likely to relate to an “actual probability threshold” of around 0.2. In other words, the model has a tendency to underestimate the probability of *Pf*PR_2−10_ taking low values (Figure S4.1A in Protocol S4). This tendency may have led, in turn, to overestimates of *Pf*PR_2−10_ in some low endemicity areas.

### Global *P. falciparum* Malaria Endemicity Map

**Figure 7:**
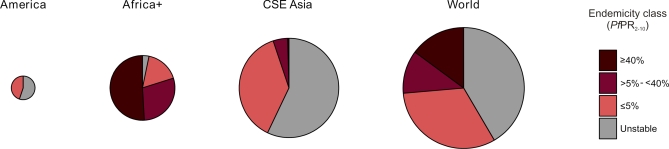
Pie Charts Showing the PAR of P. falciparum Malaria in 2007 The charts show the proportion of the population living in each predicted *Pf*PR_2−10_ endemicity classes for the America, Africa+, CSE Asia regions, and worldwide. The charts are scaled proportionally to the total population at risk in each region and the segments are coloured to match the endemicity classes shown in Figure 4.

In 2007 the global area at risk of stable P. falciparum malaria was 29.73 million km^2^, distributed between the Americas (6.03 million km^2^, 20.30%), Africa+ (18.17 million km^2^, 61.10%), and CSE Asia regions (5.53 million km^2^, 18.60%) (Table 3). We have estimated previously that there are 2.37 billion people at any risk of P. falciparum transmission worldwide and that 0.98 billion of these live where the risk is unstable [17,18]. Those exposed to stable risk, 1.383 billion, are distributed between the Americas (0.041 billion, 2.94%), Africa+ (0.657 billion, 47.48%), and CSE Asia (0.686 billion, 49.58%) (Figure 7; Table 4). The regional variation in stable P. falciparum risk, stratified by the low (*Pf*PR_2−10_ ≤ 5%), intermediate (*Pf*PR_2−10_ > 5 to < 40%), and high (*Pf*PR_2−10_ ≥ 40%) endemicity classes facilitated by these analyses are described below. In the Americas and CSE Asia, children (the 0–4 y and 5–14 y age groupings) approach a third (32% each) of the total PAR. In Africa+ this proportion rises to 43%.

**Table 3:**
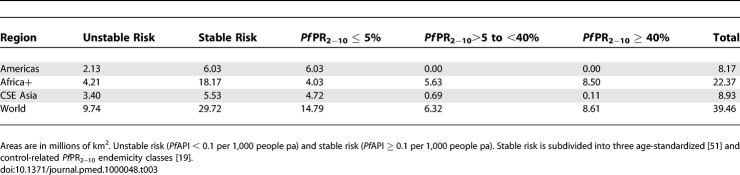
Area at Risk of P. falciparum Malaria in 2007

**Table 4:**
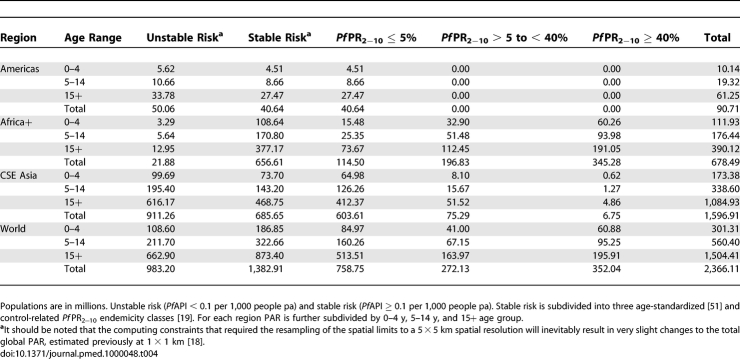
PAR of P. falciparum Malaria in 2007

### The Americas

**Figure 8:**
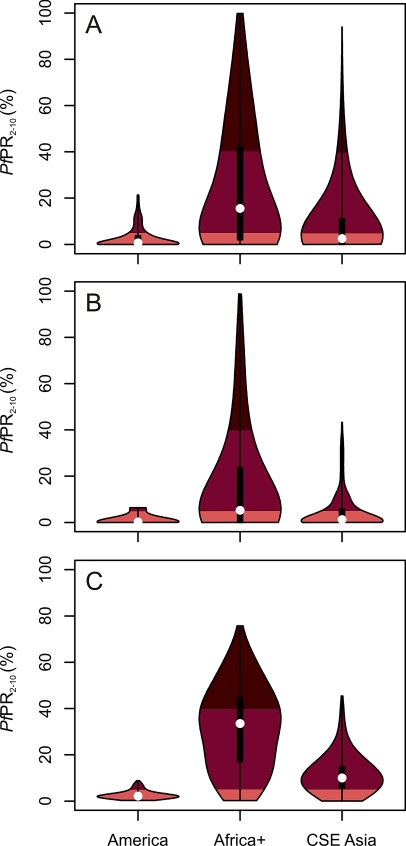
Violin Plots Showing for Each Region Frequency Distributions of *Pf*PR_2−10_ data (A) For all years, (B) for 2007, and (C) for the predicted 2007 surface. The width of each polygon illustrates the relative frequency of different *Pf*PR_2−10_ values within each region. The background is coloured to match the endemicity classes shown in Figure 4. The black central bar indicates the inter-quartile range and white circles indicate the median values (see text).

The stable P. falciparum transmission area of the Americas is characterised by a uniformly low endemicity (*Pf*PR_2−10_ ≤ 5%) (Figures 3 and 4). The total area at stable risk covers 6.03 million km^2^, mostly located in the Amazon basin (Figures 3 and 4). All the 40.64 million people in this region are exposed to this low risk. The median prevalence was 2.17% with the lowest and highest predicted *Pf*PR_2−10_ values 0.31% and 8.81%, respectively (Figure 8C). Examination of the frequency distributions for the region showed predicted values distributed approximately symmetrically around this median value (Figure 8C). The input data for 2007 (Figure 8B) showed a similar range but were positively skewed, whilst those for all years included values over a larger range (max = 21.30%) and displayed a pronounced positive skew (Figure 8A). The probability of correct endemicity class assignments was high in the Americas (Figure S4.1A in Protocol S4), due mainly to the relative uniformity of the low *Pf*PR_2−10_ value survey data [17,18], rather than any strong spatial structure (Figure S1.1 in Protocol S1). This result, combined with the relatively low population density of the region, led to the lowest values of the population weighted index of uncertainty (Figure 5B).

### Africa+

The stable P. falciparum transmission area in the Africa+ region covers 18.17 million km^2^, which contains 656.61 million people at risk and spans a wide range in transmission intensity. Over 4.03 million km^2^ (22.18%) of this area and 114.50 million people (17.44%) experience *Pf*PR_2−10_ ≤ 5%. These areas are located in the central and eastern extents of the southern and northern most latitudes (Figures 3 and 4). This endemicity class was relatively confidently predicted (Figure S4.1A in Protocol S4). The high transmission regions where *Pf*PR_2−10_ ≥ 40% dominate West Africa and large areas of Central Africa, covering 8.50 million km^2^, in which 345.28 million people are at risk. The probability of correct endemicity class prediction was high in West Africa and much lower in Central Africa (Figure S4.1C in Protocol S4), due to the relative abundance of contemporary *Pf*PR_2−10_ survey data in the former region and paucity in the latter (Figure 1). A significant area of the continent (5.63 million km^2^) has intermediate endemicity values, *Pf*PR_2−10_ > 5% to < 40%, and contains 196.83 million PAR. This endemicity class was predicted with the least confidence (Figure S4.1B in Protocol S4).

The median predicted prevalence for the stable endemicity area of the continent was 33.34%, with the lowest and highest predicted *Pf*PR_2−10_ values 0.20% and 75.40%, respectively (Figure 8C). The frequency distribution of predicted values (Figure 8) was centred on this median value, with a much less pronounced secondary mode centred at around 15% (Figure 8C). This distribution was very different to those of the all-year and 2007 input data, which were both positively skewed with maximum values of 99.78% and 98.70%, respectively (Figure 8A and 8B, respectively). The population weighted index of uncertainty shows a mixed picture for the region, with high values evident in Ethiopia for the low endemicity class and high values evident in Nigeria for the high endemicity class (Figure 5B), reflecting the co-occurrence of both low density of *Pf*PR_2−10_ surveys and large populations in each country.

### CSE Asia

The stable P. falciparum transmission area of the CSE Asia region is characterised by low malaria endemicity (*Pf*PR_2−10_ ≤ 5%), with geographically small but epidemiologically important patches of intermediate (*Pf*PR_2−10_ > 5 to < 40%) and high risk (*Pf*PR_2−10_ ≥ 40%) in for example, Orissa state, eastern India, western Myanmar, and the lowlands of New Guinea. The total area at stable risk covers 5.53 million km^2^, which contains 685.65 million PAR, mostly located in India and Indonesia (Figures 3 and 4). Over 4.72 million km^2^ (85.54%) of this area and 603.61 million (88.03%) people experience *Pf*PR_2−10_ ≤ 5%. The median predicted prevalence was 9.99%, with the lowest and highest predicted *Pf*PR_2−10_ values 0.006% and 45.40%, respectively. The frequency distribution of predicted *Pf*PR_2−10_ values was positively skewed (Figure 8C). The frequency distribution of the 2007 input data spanned a similar range of values, but displayed a more pronounced positive skew (Figure 8B). The plot for data from all years was also positively skewed but covered a much larger range of values, with a maximum of 93.91% (Figure 8A). The probability of correct endemicity class assignments was relatively high in the CSE Asia region, but with considerable uncertainty in the border areas between the low and intermediate endemicity classes (Figure S4.1A in Protocol S4). This result, combined with the high population density of the region, led to highest values of the population weighted index of uncertainty, notable particularly in India (Figure 5B).

## Discussion

We have to our knowledge, for the first time in 40 y provided a contemporary map of P. falciparum malaria endemicity at the global scale. The map addresses the key deficiencies of older maps of the global distribution of malaria risk outlined previously and therefore is unique in the following ways. First, it is based on a heavily documented and geographically extensive malariometric survey database (Protocol S1) [58] that will be released in the public domain (where permission has been granted for individual surveys) for all to use and evaluate in 2009 [1]. Second, the MBG methods (Protocol S3) and validation procedures (Protocol S4) have also been documented in exhaustive detail and the relevant code been made available in the public domain. The entire mapping process should therefore be reproducible by those with access to the requisite computing resources. Third, a rigorous assessment of the uncertainty associated with the mapped outputs has been undertaken so that the confidence in the results can be evaluated objectively (Figure 5).

### The World Malaria Situation in 2007

The world is substantially less malarious than would be predicted from the inspection of historical maps [5,14], both through a shrinking of the spatial limits and through a reduction in endemicity within this range. There is a striking global transition to a lower risk malaria ecology that will be explored in more detail in future work.

Of the 1.382 billion people exposed to stable malaria risk worldwide in 2007, 0.759 billion live in conditions of extremely low malaria endemicity with *Pf*PR_2−10_ ≤ 5% in the CSE Asia (0.604 billion, 79.55%), Africa+ (0.115 billion, 15.09%), and America (0.041 billion, 5.36%) regions (Figure 7; Table 4). These populations live under conditions where the biological prospects for sustained control at very low levels of malaria transmission is achievable and are ultimately compatible with a long-term movement toward elimination [19]. Specific subregional and national recommendations should of course, however, be shaped by a sober assessment of other environmental, logistical, financial, and political factors affecting the efficiency with which intervention plans might be implemented [73–75]. To a good approximation, the rest of the global population at stable malaria risk are Africans: 0.197 billion live under conditions of intermediate risk (*Pf*PR_2−10_ > 5 to < 40%) and 0.345 billion under conditions of high risk (*Pf*PR_2−10_ ≥ 40%) (Figure 7; Table 4). In the areas of intermediate risk, mathematical modelling suggests that by taking ITNs to scale, the interruption of P. falciparum malaria transmission might be achieved, whereas in the high transmission areas, malaria transmission will be more intractable and require aggressive control with suites of additional and complementary interventions [19,55].

### Statistical Implementation and Model Validation

The modelling procedure presented here represents a large scale implementation of modern Bayesian geostatistical techniques and incorporates a number of novel components. The incorporation of an age-standardization model has allowed the coherent assimilation of survey data obtained across a wide variety of surveyed age ranges whilst acknowledging the uncertainty introduced by this additional source of variation. Likewise, the use of a fully spatiotemporal random field has allowed surveys from as early as 1985 to be incorporated in the prediction of contemporary P. falciparum endemicity in a statistically and epidemiologically plausible framework.

MBG techniques are exceptionally computationally demanding even for small prediction problems. To our knowledge this is the first time these procedures have been applied to any disease at the global scale. This computational burden has also imposed a number of restrictions on the modelling procedure that may have improved predictive capability. In particular, the current model adopts a single mean and covariance function within each global region, representing an assumption of second-order stationarity within each. Approximations to nonstationary random fields adopted in smaller scale studies [32,76] represent possible refinements to the current model, but were considered computationally infeasible globally.

Assessment of the various validation statistics revealed that the model performed satisfactorily for each of the three performance aspects: predicting *Pf*PR_2−10_ point values and endemicity class, and providing realistic measures of prediction uncertainty. Given the highly variable nature of P. falciparum endemicity over even short distances, an overall correlation of 0.82 between the model predictions and validation data, and an average absolute error magnitude of 9.75% *Pf*PR_2−10_ represents an unexpected level of precision. Certain aspects of the uncertainty measures output by the model are suboptimal: in particular, the tendency to underestimate slightly the probability of *Pf*PR_2−10_ taking very low values. Nevertheless, given the multitude of sources of uncertainty that are captured and propagated though the modelling framework, the resulting uncertainty predictions represent a rich source of information in the generation of output products for decision makers.

The model was fitted using MCMC [77,78]. MCMC is an extremely powerful algorithm, and is the only general-purpose, computationally tractable algorithm available for many Bayesian problems. However, it is an approximate algorithm. No fail-proof method for estimating its error is available, but using a heuristic method (Protocol S1.3) we estimated that our “Monte Carlo error” is unimportant relative to the uncertainty in our actual posterior distributions.

The information contained in the maps presented here and the associated uncertainty varies across a range of geographical scales. The large-scale variation in endemicity described between regions and countries is unambiguous, robustly quantified, and of direct use to global planners. As progressively finer scales are considered, however, the utility of these maps for local malaria control managers diminishes although this is heavily dependent on the local availability and density of survey points. The appropriate threshold and metric of uncertainty will vary enormously for different end users and applications of the maps. As a rule-of-thumb, however, it is suggested that the differentiation in endemicity between areas smaller than the first administrative level would be inappropriate for most countries.

Examination of the frequency distributions for all-year and 2007 input *Pf*PR_2−10_ data, and for the predicted *Pf*PR_2−10_ surface, revealed a number of important features. Firstly, 2007 data from all three regions displayed substantially smaller median and maximum values and were more positively skewed than data from all years considered together (compare Figure 8A and 8B). Secondly, there were marked differences in all regions between the distribution of 2007 data values and the distribution of values from the predicted *Pf*PR_2−10_ surface (compare Figure 8B and 8C). Specifically, the latter distributions had larger medians, were less positively skewed, and for the Americas and Africa+ had substantially smaller maximum values. The overall shift towards higher *Pf*PR_2−10_ in the predicted surfaces can be attributed to the spatial clustering of the survey locations. It must always be remembered that the set of surveys collated represents an opportunistic sample driven by the motivations and constraints of a multitude of individuals, organizations, and governments. Visual examination of this set reveals a considerably larger proportion located in lower endemicity regions than would be the case in a spatially random sample and, as such, summary statistics of these raw data display a substantial bias. By predicting endemicity over a continuous surface, the MBG process compensated implicitly for this clustering in the output maps and the resulting frequency distribution was not biased in the same way.

The MBG process makes predictions at unsampled locations using linear combinations of survey data. For this reason, the resulting surfaces are inevitably smoother than the raw data from which they are predicted. One feature of this smoothing process is that the range of extreme high and low values in the predicted surface is likely to be smaller than that displayed by the input data. This explains why the frequency distributions for the predicted *Pf*PR_2−10_ surface cover substantially smaller ranges of values than those of the input data. An important implication of this smoothing effect is that the predicted surface provides a more robust prediction of endemicity at larger scales but is less able to represent faithfully the short-scale variations occurring over very short distances.

### Using Environmental Covariates to Make Continuous Maps

The extreme limiting effects of climate covariates have been incorporated comprehensively in the definition of the stable and unstable limits of P. falciparum malaria transmission described above [18]. There is an illusory attraction in the further use of environmental covariates to increase complexity and improve predictive accuracy in MBG endemicity mapping. This is because such analyses are based on the assumption that the contemporary distribution and endemicity of malaria approximates its fundamental niche [79,80]. This assumption is unfounded because the global distribution of malaria has contracted substantially [18] since its hypothesised maximum distribution circa 1900 [14]. Moreover, it is not known to what extent the environmental determinants of the remaining distribution reflect this fundamental niche, how these relationships might vary spatially, and therefore, what artefacts might be introduced by their inclusion in the analyses. In addition, it is not trivial to obtain “adequate” environmental covariates at a global level with the required spatial and temporal fidelity [63,81]. Finally, the degree to which these relations would be further obscured by ongoing and spatially variable intervention efforts is also unquantified. An increasing body of evidence points to these intervention effects being substantial, to have accelerated in the post 2000 period, and to represent a spatial mosaic of influence that would act to confound substantially any modelled relationships [82–90]. Unsurprisingly, no statistical support was found for the inclusion of a range of climate [62] and remotely sensed [63] environmental covariates (Protocol S1.7).

In eschewing the use of environmental covariates in this analysis framework, the output maps are determined only by the input survey data and the assumptions of the modelling. This choice ensures a maximally parsimonious baseline, against which future changes may be audited.

### Potential Geostatistical Improvements

In embracing the MBG approach, the rationale for excluding surveys with a sample size below 50 is diminished, as the uncertainty in relation to the population sampled is explicitly modelled by the technique (Protocol S3). This exclusion rule was devised at a time before MBG could be applied at a global scale and will be revised in future iterations of the map.

The spatial resolution with which these MBG techniques could be reasonably implemented on a computer cluster was on a 5 × 5 km grid. The entire process took an average of one month at this spatial resolution and has been estimated to take one year to run on a 1 × 1 km spatial grid. There are no plans to increase the spatial resolution of the output maps at the global scale because they are robust for the regional planning purposes for which they are intended. For smaller areas, such as *Pf*PR data rich countries where higher spatial resolution maps may be desirable to support national control plans, however, MBG outputs to 1 × 1 km grids can be considered [33]. Moreover, at these national scales, the fidelity of the geo-positioning of the input *Pf*PR survey data may have an important influence on the uncertainty of the predictions, so procedures that can help incorporate these effects into the modelling may also need to be investigated [91–93]. In this study, the uncertainty likely to be contributed by geo-positioning errors was thought to be trivial in relation to the scales of spatial variation in observed endemicity and given the global scale of model outputs.

We were not able to improve the age-correction model's predictive performance by modelling the age-dependent sensitivities of microscopy and rapid diagnostic tests separately or by modelling diagnostic specificity. The accuracy in the determination of *Pf*PR by microscopy or rapid diagnostic tests were assumed to be equivalent in these analyses, but the sensitivity of the diagnostic technique [94–98] could be included into a future iterations of this MBG framework.

No solution could be found to applying these MBG techniques across large tracts of ocean (for example in the Caribbean, Madagascar, and the Indonesian archipelago), given the global distribution of the *Pf*PR data and the lack of data in some regions (Figure 1). Potential biogeographical influences on malaria transmission on islands are ignored by these analyses. Future map iterations would ideally have sufficient data to treat islands separately or sufficient information on the distribution of *Anopheles* vectors to help inform the predictions [56].

We have incorporated the ability for the analyses to be cognisant of secular trends in the *Pf*PR data and of annual variations in transmission. This map does not provide a full description of seasonal malaria dynamics [99–101], however, and further information on the global variation of malaria seasonality might inform future map iterations.

### The Road Ahead: Public Domain and Dynamic Maps

These mapped surfaces are made available in the public domain with the publication of this article. The underlying data used in their predictions are due for public release in 2009 [1], and the online infrastructure to host this service is under development. The MAP team anticipate providing annual updates of this P. falciparum global malaria endemicity map and the accompanying *Pf*PR database. Annual updates will also be required to reflect the changing spatial limits of stable and unstable P. falciparum malaria transmission [18] in order to define accurately the limits within which endemicity predictions need to be made. If the international community is successful in rolling back malaria, informed decisions will need to be made about the temporal discontinuity between the spatial limits of P. falciparum malaria transmission (defined, where possible, by the average *Pf*API in the three most recently recorded years [18]) and the endemicity data (*Pf*PR collected since 1985).

It is obvious that the predicted map represents a snapshot of the year 2007 from a malaria endemicity that changes through time. No degree of statistical sophistication can circumvent the fact that additional data will increase the fidelity of the map, by either increasing the spatial resolution of the malariometric surveys or updating an existing survey location with more recent information. The methods have been devised specifically so that these surfaces can be updated rapidly. The predominantly univariate approach adopted also means changes in future maps' iterations can be attributed reliably to finding more data in areas of high uncertainty (changes in space) or to changes brought about through intervention success or disease recession (changes in time), rather than any temporal and spatial mix of the relationship of the *Pf*PR_2−10_ data and the environmental covariates.

We encourage the submission of additional existing data to improve the map in areas where we have least spatial accuracy, and new data to sustain future production of updated contemporary maps. Current areas of highest uncertainty are indicated to a good approximation by the inverse of the class prediction probability (Figure 5), although future work is aimed at refining this information. Therefore, an immediate priority is to generate regional maps showing the optimal location of new surveys that would need to be implemented to maximally reduce the variance in the existing endemicity surface for the minimum cost. These solutions are substantially more involved than the list of areas with highest variance provided here because (i) each new survey will change the structure of the spatial variance and affect the optimal location of the next survey; (ii) both the number and spatial distribution of surveys will affect the outcome and require multiple simulations to converge on optimal solutions; and (iii) potential survey locations will need to be weighted appropriately by the distribution of the human population.

### Immediate MAP Goals

The initial focus of the MAP has been P. falciparum [1] due to its global epidemiological significance [102] and its better prospects for control and local elimination [19]. We have not yet addressed the significant problem of P. vivax burden [103] despite its increasingly recognised clinical importance [104–106], but have archived over 2,500 P. vivax parasite rate surveys with which to start this process. Another immediate goal is in refining global burden of disease estimates for P. falciparum (both morbidity [102] and mortality [48,107,108]) to support global estimation of antimalarial intervention and commodity needs. The statistical methods used in this analysis will allow the next iteration of burden estimates to represent more holistically and robustly the uncertainty in predictions. In the medium term, combinations of these global endemicity maps with forthcoming maps of the distribution of the dominant *Anopheles* vectors of human malaria [56] should empower malaria control managers to make more informed decisions regarding interventions appropriate to the bionomics of their local suite of vectors. In the long term we hope to not only monitor and evaluate progress with these maps, but to increase our ability to model future malaria endemicity and support objective assessment of where in the world it might be possible to eliminate malaria.

### Conclusions

The state of the P. falciparum malaria world in 2007 represents an enormous opportunity for the international community to act [109,110], but these actions remain considerably under-resourced [111]. Regardless of whether nations champion sustained, intensive control or reach for the higher ambition of malaria elimination [2–4,74,112–114], the intermediate intervention paths are similar [19]. This cartographic resource will help countries determine their needs and serve as a baseline to monitor and evaluate progress towards interventional goals. We wish to continue to work alongside individuals, countries, and regions to improve future iterations of this map and document hopefully these intervention successes.

## Supporting Information

Alternative Language Text S1 Translation of the Article into French by Frédéric Piel and Stéphanie Loute (1.04 MB DOC)

Alternative Language Text S2 Translation of the Article into Chinese by Robert Li (438 KB DOC)

Alternative Language Text S3 Translation of the Article into Indonesian by Iqbal R.F. Elyazar and Siti Nurlela (1.08 MB DOC)

Alternative Language Text S4 Translation of the Article into Vietnamese by Bui H. Manh (549 KB DOC)

Alternative Language Text S5 Translation of the Article into Spanish by Carlos A. Guerra (796 KB DOC)

Protocol S1 The *Pf*PR Malariometric Survey Database S1.1 Summary of Data Search and Data Abstraction Procedures
S1.2 Data Exclusion Rules
S1.3 Age-Standardisation
S1.4 Semi-Variograms of *Pf*PR_2−10_ Data by Region
S1.5 Geostatistical Filter for the Detection of Extreme Outliers
S1.6 Malariometric Survey Data Summary and Descriptive Statistics
S1.7 Relationships with Environmental Covariates
(3.4 MB DOC)

Protocol S2 Demographic Databases and Procedures S2.1 Parasite Rate Survey Urban/Peri-Urban/Rural Classification Rules
S2.2 Urban/Peri-Urban/Rural Status and Prevalence
S2.3 GRUMP alpha Human Population Surface
S2.4 PAR Derivation
(2.5 MB DOC)

Protocol S3 Model Based Geostatistical Procedures S3.1 Overview of the Statistical Model
S3.2 Prior Specification
S3.3 Age-Standardization
S3.4 Implementation Details
S3.5 Overview of Map Generation
(23 MB DOC)

Protocol S4 Model Validation Procedures S4.1 Creation of the Validation Sets
S4.2 Procedures for Testing Model Performance
S4.3 Additional Results
(26 MB DOC)

## Abbreviations

Africa+: Africa, Yemen, and Saudi Arabia
AUC: area-under-curve
CSE Asia: Central and South and East Asia
GRUMP: Global Rural Urban Mapping Project
ITN: insecticide treated net
MAP: Malaria Atlas Project
MBG: model-based geostatistics
PA: per annum
PAR: population at risk
P*f*API: P. *falciparum* annual parasite incidence
P*f*PR: P. *falciparum* parasite rate
UE: urban extent
