# Correction: A World Malaria Map: Plasmodium falciparum Endemicity in 2007

**DOI:** 10.1371/annotation/a7ab5bb8-c3bb-4f01-aa34-65cc53af065d

**Published:** 2009-10-08

**Authors:** Simon I Hay, Carlos A Guerra, Peter W Gething, Anand P Patil, Andrew J Tatem, Abdisalan M Noor, Caroline W Kabaria, Bui H Manh, Iqbal R. F Elyazar, Simon Brooker, David L Smith, Rana A Moyeed, Robert W Snow

Reference 56 [Hay SI, Sinka ME, Tatem AJ, Patil AP, Guerra CA, et al. (2009) Developing global maps of the dominant Anopheles vectors of human malaria. PLoS Med. In press.] was erroneously listed as "In press." It was in preparation at the time but was not published.

