# Supplementary material for: A World Malaria Map: Plasmodium falciparum Endemicity in 2007
Source: PLoS Med. 2009 Mar 24;6(3):e1000048. doi: 10.1371/journal.pmed.1000048 (PMC2659708; doi:10.1371/journal.pmed.1000048)
Supplement: Supplementary file 1 [file pmed.1000048.sd001.doc]

**Carte mondiale du paludisme: l’endémicité de *Plasmodium falciparum* en 2007**

Simon I. Hay^1,2*^, Carlos A. Guerra^1,2^, Peter W. Gething^2,3^, Anand P. Patil^2^, Andrew J. Tatem^1,2,4,5^, Abdisalan M. Noor^1,6^, Caroline W. Kabaria^1^, Bui H. Manh^7^, Iqbal R. F. Elyazar^8^, Simon Brooker^1,9^, David L. Smith^5,10^, Rana A. Moyeed^11^ et Robert W. Snow^1,6^

1. Malaria Public Health and Epidemiology Group, Centre for Geographic Medicine, Kenya Medical Research Institute (KEMRI) – University of Oxford - Wellcome Trust Collaborative Programme, Nairobi, Kenya,

2. Spatial Ecology and Epidemiology Group, Department of Zoology, University of Oxford, Oxford, Royaume-Uni,

3. Centre for Geographical Health Research, School of Geography, University of Southampton, Highfield, Southampton, Royaume-Uni,

4. Department of Geography, University of Florida, Gainesville, Floride, Etats-Unis,

5. Emerging Pathogens Institute, University of Florida, Gainesville, Floride, Etats-Unis,

6. Centre for Tropical Medicine, Nuffield Department of Clinical Medicine, University of Oxford, Centre for Clinical Vaccinology and Tropical Medicine (CCVTM), Oxford, Royaume-Uni,

7. Oxford University Clinical Research Unit, Bach Mai Hospital, National Institute of Infectious and Tropical Diseases, Ha Noi, Vietnam,

8. Eijkman-Oxford Clinical Research Unit, Jakarta, Indonésie,

9. Department of Infectious and Tropical Diseases, London School of Hygiene and Tropical Medicine, Royaume-Uni,

10. Department of Zoology, University of Florida, Gainesville, Floride, Etats-Unis,

11. School of Mathematics and Statistics, University of Plymouth, Plymouth, Devon, Royaume-Uni

**Résumé**

Contexte

Une meilleure compréhension de la distribution spatiale actuelle des risques liés au paludisme est nécessaire afin de répartir efficacement les ressources pour la lutte contre cette maladie. La publication de la dernière carte mondiale de l’endémicité du paludisme remonte à il y a exactement quarante ans. Cet article décrit la création d’une nouvelle carte mondiale de l’endémicité du paludisme causé par *Plasmodium falciparum* en 2007.

Méthodologie et résultats

Au total, 8938 études sur le taux de parasitisme de *P. falciparum* (TP*Pf*) ont été identifiées grâce à différentes stratégies exhaustives de recherche. Parmi ces études, 7953 ont satisfait à des critères de sélection très stricts avant leur inclusion dans une base de données mondiale sur le TP*Pf*. Les données de notre carte d’endémicité ont été standardisées pour une tranche d’âge allant de 2 à 10 ans. Des méthodes géostatistiques de modélisation ont été utilisées pour créer une surface continue d’endémicité du paludisme au sein des limites spatiales définies préalablement comme stables pour la transmission de *P. falciparum*. Ces méthodes ont été intégrées dans un cadre de statistiques bayésiennes afin de prendre en compte, grâce à des méthodes robustes, les incertitudes des prévisions. Ces incertitudes sont représentées par la probabilité de prédire correctement l’une des trois classes d’endémicité, classes ayant été définies antérieurement afin de guider de manière informative la lutte contre le paludisme. Les estimations des populations à risque, ajustées afin de prendre en compte les effets de l’urbanisation sur la transmission du paludisme en Afrique, ont ensuite été calculées d’après les données de répartition des populations humaines en 2007.

Parmi le milliard trois cent quatre-vingt millions de personnes confrontées à un risque stable de paludisme causé par *P. falciparum*, 0,69 milliard se trouvent en Asie centrale et en Asie du Sud-Est (Asie CSE), 0,66 milliard en Afrique, au Yémen et en Arabie saoudite (Afrique+) et 0,04 milliard sur les continents américains. Tous les individus exposés à un risque stable en Amériques se situent dans la classe d’endémicité la plus basse (TP*Pf*_2-10_ ≤5%). La majeure partie (88%) des personnes vivant en Asie CSE dans des régions à risque stable se trouve également dans cette classe d’endémicité peu élevée; 11% se situent dans la classe d’endémicité intermédiaire (TP*Pf*_2-10_ >5 et <40%) et le pourcent restant (1%), dans des zones d’endémicité élevée (TP*Pf*_2-10_ ≥40%). Cette dernière catégorie prévaut dans la région Afrique+, où 0,35 milliard de personnes sont confrontées à un risque élevé. Le risque est intermédiaire pour le reste de la population vivant de cette région (0,20 milliard), excepté pour 0,11 milliard d’individus vivant dans des zones à risque stable même si peu élevé.

Conclusions

Les niveaux élevés d’endémicité du paludisme causé par *P. falciparum* sont courants en Afrique. Les niveaux d’endémicité sont par contre uniformément bas sur les continents américains. Une endémicité faible prévaut également en Asie CSE, mais des poches de transmission intermédiaire, voire élevée dans de rares régions, y persistent. Il existe dès lors des opportunités certaines pour contrôler le paludisme en Afrique et pour éliminer cette maladie partout ailleurs. Cette carte mondiale de l’endémicité du paludisme causé par *P. falciparum* en 2007 est la première d’une série qui permettra de suivre et d’évaluer les progrès de cette lutte contre le paludisme.

**Introduction**

Les cartes sont essentielles pour tous les aspects de la coordination de la lutte contre le paludisme [1]. Dans le contexte d'une politique internationale au sein de la communauté luttant contre le paludisme, considérant à nouveau la possibilité d'éradiquer cette maladie [2-4], une carte de la distribution du paludisme constitue un outil primordial pour la planification, la mise en œuvre et la mesure de l’impact des interventions contre la malaria à l’échelle mondiale. La dernière carte mondiale de l’endémicité de *Plasmodium falciparum* a été publiée en 1968 [5]. Comme toutes les cartes antérieures de la distribution mondiale du paludisme [6-10] et, dans une large mesure, celles qui lui ont succédé [11-16], cette carte (i) fournissait une description incomplète des données utilisées, (ii) délimitait les contours des régions à «risque» sur base d’avis peu détaillés et subjectifs d’experts et (iii) ne donnait aucun chiffre concernant l’incertitude des prévisions. Nous décrivons ici la production d’une nouvelle carte mondiale de l’endémicité du paludisme comblant ces lacunes importantes.

Cadre géographique de la modélisation

Les limites spatiales de la transmission du paludisme causé par *P. falciparum* dans le monde ont été récemment cartographiées en combinant des données sur l'incidence des cas de paludisme recensés dans chaque pays, des connaissances médicales complémentaires et les critères biologiques empêchant la transmission du paludisme [17,18]. Ces derniers sont basés sur la température et les limites de sécheresse tolérée biologiquement par les *Anophèles* vecteurs dominants localement. Cette méthode a permis de diviser le monde en trois classes: la représentation spatiale de l'absence de risque, celle d’un risque instable (Incidence Parasitaire Annuelle de *Plasmodium falciparum* (IPA*Pf*) inférieure à 0,1 pour 1000 individus par an) et celle d’un risque stable (IPA*Pf* supérieure ou égale à 0,1 pour 1000 individus par an) pour la transmission de *P. falciparum* en 2007. La distribution de ces trois classes est illustrée par la Figure 1. La classification stable vs. instable de l’IPA*Pf* est basée sur la compilation des raisons statistiques, logistiques, organisationnelles et pragmatiques qui influencent les niveaux d’IPA*Pf* utilisés pour définir les points d’action de la campagne mondiale d’éradication du paludisme [19-21].

L’exercice de cartographie décrit ici prolonge substantiellement ce travail. D’une part, il se base sur le plus grand ensemble d’études malariométriques jamais rassemblé à l’échelle mondiale. D’autre part, les valeurs de prévalence du paludisme causé par *P. falciparum* sont prédites pour chaque point situé au sein des limites spatiales de transmission stable, afin d’obtenir une surface continue de l’endémicité de *P. falciparum*. Pour faciliter cette étape, les limites spatiales ont été rééchantillonnées avec ArcView GIS 3.2 (ESRI, 1999) selon une grille de 5×5 km. Les méthodes de cartographie décrites ci-dessous ne pouvaient en effet pas être appliquées à une résolution spatiale de 1×1 km à l’échelle mondiale en raison des limites des performances des ordinateurs actuels.

Approches récentes dans la cartographie de l’endémicité du paludisme

Il existe de nombreuses méthodes pour la production de cartes continues de l’endémicité basées sur des données d’études malariométriques. Elles nécessitent toutes l’utilisation d’un modèle permettant de prédire des valeurs d’endémicité aux endroits pour lesquels aucune donnée n’est disponible [22-26]. Les cartes générées par ces modèles possèdent une incertitude inhérente, et la quantification de celle-ci est un problème important pour la cartographie de maladies.

Un certain nombre d’études récentes ont adopté un cadre prédictif connu sous le nom de «model-based geostatistics» (MBG) [27] pour la prévision spatiale de l’endémicité du paludisme [28-33] ou pour la prévalence d’autres maladies transmises par un vecteur ou par un hôte intermédiaire [34-38]. Les MBG fournissent une interprétation statistique explicite des outils géostatistiques classiques de prévision spatiale [39-41] et permettent d’incorporer les méthodes bayésiennes d’inférence statistique [42,43]. Le principal avantage des MBG pour la cartographie d’une maladie réside dans sa gestion rigoureuse de l’incertitude introduite durant les différentes étapes du processus de modélisation [27]. En modélisant l’interaction entre ces différentes sources d’incertitude, une distribution de probabilité est générée pour chaque valeur prédite. Celle-ci peut être synthétisée afin de fournir des mesures robustes de fiabilité pour les valeurs prédites. La carte résultante fournit dès lors un modèle de l’endémicité actuelle mondiale du paludisme, basé sur des données rigoureuses et utilisant les méthodes des MBG pour estimer la fiabilité des prévisions. Ses utilisateurs disposent ainsi d’une estimation claire de la fidélité de celles-ci [36].

Un principe sous-jacent aux géostatistiques implique qu’une prévision cartographiée devient de moins en moins fiable au fur et à mesure que la densité et la proximité des points de donnée avoisinants décroissent. Lorsque les données portent sur des périodes et des endroits différents, ce principe s’applique à travers le temps et à travers l’espace. Il y a peu d’exemples d’études épidémiologiques incorporant les géostatistiques spatiales dans un cadre temporel [44-47]. Dans cette étude, un cadre spatio-temporel complet de modélisation à l’aide de géostatistiques a été développé. L’inclusion de la dimension temporelle permet de comparer sans ambiguïté ce modèle avec de futures mises à jour de la carte. Celle-ci proposera ainsi un cadre géographiquement explicite pour le suivi et l’évaluation de l’impact des mesures de contrôle contre le paludisme causé par *P. falciparum* dans le monde entier.

**Méthodes**

Résumé des analyses

Ces analyses ont pour objectif d’utiliser une base de données contemporaine d’enquêtes sur le taux de parasitisme de *P. falciparum* (TP*Pf*) afin de créer une surface continue de l’endémicité du paludisme causé par *P. falciparum* à l’échelle mondiale pour 2007. Les méthodes sont transparentes et reproductibles, et l’incertitude associée aux prévisions est documentée de façon rigoureuse.

Les principales étapes suivies, dans notre cadre analytique, pour la conception de cette carte mondiale continue de la prévalence de *P. falciparum* sont exposées à la Figure 2. La recherche et le prétraitement des données de TP*Pf* étaient tout d’abord nécessaires afin de générer un ensemble géographiquement cohérent et parfaitement géo-référencé pour les cartographier les données des études malariométriques. Les covariables environnementales potentielles (Protocole S1) et l’influence de la structure des zones urbaines ont été évaluées [48-50] (Protocole S2). Après ces raffinements, la base de données des TP*Pf* a servi à créer une surface continue de la prévalence du paludisme, standardisée par âge et corrigée dans les zones urbaines, au moyen de MBG implémentées dans un cadre statistique bayésien (Protocole S3). Des méthodes poussées de validation ont ensuite été utilisées pour évaluer la précision des prévisions de l’endémicité et les indices d’incertitude (Protocole S4). Les estimations des populations à risque (PAR) pour le paludisme causé par *P. falciparum* ont finalement été calculées à l’échelle mondiale et présentées par classes d’âge et à l’échelle régionale.

Création d’une base de données mondiale de la prévalence parasitaire de *P. falciparum*

Parmi tous les indicateurs potentiellement disponibles pour mesurer l’endémicité du paludisme, le taux de parasitisme (c'est-à-dire la proportion des personnes échantillonnées pour laquelle les parasites sont détectables dans le sang périphérique) a été sélectionné pour servir de base à notre carte. Cet indicateur est en effet largement utilisé à l’échelle mondiale et il est sensible à une large gamme du spectre de transmission du paludisme causé par *P. falciparum*. Une classification du spectre de l’endémicité du paludisme au sein d’une classe d’âge allant de deux (2,00) à dix (9,99) ans a été suggérée [51]. Le choix de cette classe d’âge, informative d’un point de vue épidémiologique, est guidé par l’impact potentiel sur l’endémicité du paludisme grâce aux moustiquaires imprégnées d’insecticide (MII), qui constituent la méthode d’intervention la plus utilisée à l’heure actuelle [19]. La classe la plus basse du TP*Pf* dans le groupe d’âge de deux à dix ans (ci-après TP*Pf*_2-10_) correspond à un taux ≤5%. Cette valeur équivaut à la limite sous laquelle la taille de l’échantillon de population nécessaire pour mesurer précisément l’endémicité est trop importante d’un point de vue logistique. Dans ce cas, les mesures malariométriques basées sur la surveillance sont généralement privilégiées [52-54]. Nous avons considéré qu’une transmission intermédiaire stable correspond à un TP*Pf*_2-10_ >5% et <40%. Plusieurs modèles mathématiques prédisent en effet que l’interruption de la transmission du paludisme pourrait être atteinte grâce à une utilisation universelle de MII dans toutes les zones présentant un TP*Pf*_2-10_ <40% [19,55]. Malgré une incertitude relative concernant ce seuil liée au comportement et à la biologie des *Anophèles* vecteurs dominants localement [56], le seuil du TP*Pf*_2-10_ <40% est considéré comme relativement fiable étant donné que les MII sont rarement distribuées sans autres méthodes d’intervention visant également à réduire la transmission. Les zones de transmission élevée et stable sont définies par des prévalences correspondant systématiquement à des TP*Pf*_2-10_ ≥40%. Dans ces zones, la combinaison de diverses méthodes d’intervention doit être envisagée si on veut parvenir à interrompre la transmission du paludisme. Cette classification vise à guider l’interprétation de la surface d’endémicité prédite au sein de ces différentes classes. Elle se démarque des critères utilisés traditionnellement étant donné que ceux-ci ne se sont pas avérés correspondre, dans la plupart des modèles, avec les possibilités de contrôle et d’élimination [19,55].

Le processus d’identification, d’assemblage et de géoréférencement des études de la prévalence du parasite effectuées au sein de populations depuis 1985 a été décrite préalablement [58]. Bien que le «Malaria Atlas Project» (MAP, http://www.map.ox.ac.uk) continue en permanence à rechercher de nouvelles données du TP*Pf*, seules les données acquises avant le 31 juillet 2008 ont été utilisées pour cette itération de la carte d’endémicité mondiale en 2007 (Protocol S1.1). Au total, 8938 estimations du TP*Pf* provenant d’études transversales ont été assemblées. Ces données proviennent de 78 des 87 pays endémiques pour le paludisme causé par *P. falciparum* (PE*Pf*) [18]. Le Bangladesh, le Belize, le Bhoutan, Djibouti, la République dominicaine, la Guyane, l’Iran, le Kirghizistan et Panama ne sont pas représentés dans la base de données.

Après six niveaux d’exclusion de données (exclusion des études géoréférencées par des grands (>100 km2) ou petits (>25 km2) polygones [58]; exclusion des études ne pouvant pas être géoréférencées, ou pouvant l’être seulement de manière imprécise; et exclusion des études ne pouvant temporairement pas être subdivisées en études indépendantes, ou dont la date était inconnue), 7991 études du TP*Pf* ont été conservées dans la base de données (Figure S1.2 du Protocole S1).

Toutes ces données du TP*Pf* ont ensuite été standardisées pour la tranche d’âge compris entre 2 et 10 ans, puis cartographiées avec un algorithme basé sur des modèles de conversion catalytique, adapté au paludisme pour la première fois par Pull et Grab [59]. Cet algorithme s’est avéré fournir les meilleurs résultats parmi toutes les procédures de standardisation potentielles [51] (Protocole S1.3).

Le jeu de données final a alors été divisé en trois régions principales (Figure 1): les Amériques; l’Afrique, le Yémen et l’Arabie saoudite (Afrique+); l’Asie centrale et l’Asie du Sud-Est (Asie CSE) (Protocole S1.4). Cette division a permis d’examiner séparément ces régions distinctes d’un point de vue épidémiologique, entomologique et biogéographique, tout en conservant suffisamment de données dans chaque région pour permettre des analyses intéressantes. Cette division du globe a ensuite été confirmée par l’observation d’une structure spatiale distincte des données du TP*Pf*_2-10_ au sein de chaque région. Celle-ci est illustrée par un semi-variogramme (Figure S1.1 du Protocole S1).

Des approches spécifiques pour cartographier les zones urbaines, périurbaines et rurales en relation avec la transmission du paludisme ont été développées [50] (Protocole S2). En bref, toutes les zones urbaines définies par la version alpha du masque d’urbanisation (MU) du *Global Rural Urban Mapping Project* (GRUMP) [60,61] ont été identifiées à une résolution spatiale de 1x1 km (Protocole S2.1) [50]. Au sein de ces zones, les aires dans lesquelles la densité de population est supérieure à 1000 habitants par km^2^ d’après la surface de densité de population de la version 3 du «Gridded Population of the World» (GPW3) [60,61] ont été cartographiées [48]. Toutes les études du TP*Pf* ont alors été catégorisées soit en zone urbaine (Gridded Population of the World version 3 ≥1000 km^2^ dans le MU du GRUMP), soit en zone périurbaine (Gridded Population of the World version 3 <1000 km^2^ dans le MU du GRUMP) soit en zone rurale (hors du MU du GRUMP) (Protocole S2.2).

Les valeurs statistiques marginales dans les données de TP*Pf*_2-10_ classées en zone rurale ont ensuite été identifiées grâce à un filtre géostatistique (Protocole S1/5). Cette étape fait appel aux statistiques des semi-variogrammes pour estimer si chaque point diffère significativement des points voisins en tenant compte des distances les séparant et des profils régionaux de variation spatiale. Cette procédure a identifié 38 points de TP*Pf*_2-10_ en zone non urbaine. Ceux-ci ont été retirés du jeu de données avant la modélisation. Le détail de ces études est disponible sur demande.

Le jeu de données final du TP*Pf*_2-10_ (n= 7953) est présenté à la Figure 1. Les caractéristiques de cette base de données finalisées sur le TP*Pf*_2-10_.sont décrites en détails (Tableau S1.2 du Protocole S1). Un graphique du TP*Pf*_2-10_ médian par année durant la période d’observation est aussi inclus (Figure S1.3 du Protocole S1). Celui-ci illustre le fait que le temps constitue un paramètre de variation important à inclure dans les analyses basées sur les MBG. Des analyses préliminaires similaires pour les relations entre les données de TP*Pf*_2-10_ et diverses covariables climatiques et environnementales mesurées par télédétection n’ont montré aucune corrélation importante (Figure S1.5 et S1.6 du Protocole S1), ce qui confirme l’approche univariée pour ces analyses.

L’idée, largement répandue, selon laquelle les études malariométriques ne sont menées que dans les zones de prévalence élevée est erronée. En fait, la conduite d’études nationales destinées à être représentatives de toutes les régions d’un pays est de plus en plus fréquente. De plus, la confirmation de l’absence de transmission de *P. falciparum* lors d’échantillonnages portant sur *P. vivax* a pour résultat l’enregistrement de nombreuses données de prévalence nulle lors de ces enquêtes. Au total, 119 des 261 études effectuées en Amériques, 1010 des 5307 études réalisées en Afrique+ et 775 des 2385 études en Asie CSE rapportent des valeurs nulles (Figure 1).

Détails des méthodes statistiques

Les algorithmes géostatistiques génèrent des cartes continues en prédisant les valeurs aux endroits non échantillonnés grâce à des combinaisons linaires des valeurs disponibles. Dans le présent exercice cartographique, le fait que la fiabilité des prédictions de TP*Pf*_2-10_ à un lieu non échantillonné donné sera affectée par (i) la distribution des données autour de ce lieu (densité spatiale des données exploratoires), (ii) l’intensité des variations du TP*Pf*_2-10_ à travers l’espace (hétérogénéité spatiale des données exploratoires), et (iii) le nombre de personnes échantillonnées dans chaque étude (précision des études composant les données exploratoires) est intuitif. Une approche utilisant les MBG [27] a été intégrée dans un cadre statistique bayésien afin d’incorporer ces différents paramètres dans la génération de cartes continues du TP*Pf*_2-10_ (Protocole S3). Vu que les données ont été collectées au cours de différentes périodes, entre 1985 et 2008, il était important d’inclure également la dimension temporelle dans cette approche géostatistique purement spatiale, et ce afin de représenter à la fois la densité et l’hétérogénéité des données dans l’espace et dans le temps. L’algorithme de standardisation pour l’âge a été incorporé en tant que sous-modèle afin de permettre l’estimation des erreurs inhérentes à ce processus et leur propagation dans le reste les différentes étapes successives de modélisation (Protocole S3).

Dans chaque région, un modèle géostatistique bayésien a été élaboré. La valeur sous-jacente du TP*Pf*_2-10_ en 2007, , en chaque lieu y a été modélisée grâce à une transformation d’un champ spatialement et temporellement structuré, superposé à une variation déstructurée (aléatoire) . Le nombre de réponses positives à *P. falciparum* dans un échantillon total de en chaque lieu d’étude a été modélisé comme une variable binomiale conditionnelle indépendante prenant en compte la valeur sous-jacente non observée du TP*Pf*_2-10_ standardisé par âge [36]. La composante spatio-temporelle a été représentée par un processus gaussien stationnaire avec une moyenne *µ* et une covariance définies par une version spatialement anisotrope de la fonction de la covariance espace-temps proposée par Stein [64]. Une modification a cependant été apportée à cette dernière pour incorporer une composante périodique ayant une fréquence de 12 mois dans le modèle temporel et permettre à celui-ci d’incorporer les effets saisonniers dans la structure observée de la covariance temporelle. Ces effets apparaissent lorsque des études effectuées aux mêmes mois au cours de différentes années se ressemblent d’avantage que ne l’aurait prédit le modèle en l’absence de saisonnalité. La moyenne *µ* a été modélisée comme une fonction linéaire du temps et selon le fait que l’endroit de prévision se situait en zone urbaine ou périurbaine (respectivement défini par les variables 1*_u_*(*x*) et ) plutôt que rurale: . Chaque étude a été référencée temporellement en utilisant le point médian (en années décimales) entre les mois de départ et de fin enregistrés. Un statut urbain, périurbain ou rural a été attribué à chaque lieu prédit sur base de la surface du GRUMP modifiée dans les zones urbaines et décrite précédemment (Protocole S2.2). Cette surface a été rééchantillonnée selon une grille de 5×5 km. La composante non-structurée a été représentée comme une variable gaussienne ayant une moyenne nulle et une variance . L’inférence bayésienne a été appliquée avec les méthodes de Monte Carlo par chaînes de Markov (*Markov Chain Monte Carlo*, MCMC) pour générer des échantillons de la distribution *a posteriori* du champ gaussien en chaque lieu; les paramètres non-observés , *,* , et définis plus haut; et d’autres paramètres non observés définissant la structure et l’anisotropie de la fonction exponentielle de la covariance espace-temps (Protocole S3.4). Les distances entre points ont été mesurées par la distance orthodromique pour intégrer l’effet de la courbure terrestre, qui exerce une influence non négligeable à l’échelle régionale. Des échantillons ont été générés à partir de la moyenne annuelle de 2007 de la distribution *a posteriori* de en chaque lieu prédit. Pour chaque échantillon, ces prédictions ont été effectuées avec une simulation conditionnelle spatio-temporelle sur les 12 mois de l’année 2007 [44,65]. Ces prévisions ont été faites aux nœuds d’une grille spatiale régulière de 5×5 km au sein des zones de transmission stable de *P. falciparum*. Les résultats du modèle étaient donc constitués par des échantillons de la distribution prédite *a posteriori* de la moyenne annuelle du TP*Pf*_2-10_ pour 2007 en chaque point de la grille. Ces échantillons ont servi à produire des estimations ponctuelles (calculées comme la moyenne de chaque ensemble d’échantillons *a posteriori*), des probabilités d’appartenance à une classe d’endémicité et des estimations standards de variance (Protocole S3.4). Le Protocole S3.5 fournit une description plus détaillée de la manière dont ces résultats géostatistiques ont été utilisés pour générer les différentes cartes.

Validation du modèle

L’estimation de la vraisemblance de la carte générée par le modèle était essentielle. Plusieurs méthodes descriptives non triviales ont été utilisées (Protocole S4). La capacité du modèle de prévoir des valeurs ponctuelles du TP*Pf*_2-10_ et la classe d’endémicité la plus probable a été testée au moyen d’une procédure *holdout*. Un échantillon de validation a été généré en sélectionnant 10% des données (n=800) par un échantillonnage aléatoire stratifié et désagrégé spatialement. Cet échantillon a ensuite été retiré de l’ensemble de données (Protocole S4.1). Les 7153 points de donnée restants ont été utilisés pour toutes les étapes du modèle afin de générer des distributions prédictives *a posteriori* du TP*Pf*_2-10_. Les valeurs prédites ont alors pu être comparées aux valeurs connues pour les 800 données isolées préalablement. Contrairement à la simulation principale dans laquelle les moyennes ont été prédites pour l’année 2007, le test de validation a prédit le TP*Pf*_2-10_ pour le mois médian de chacune des 800 études isolées afin de fournir des données comparables d’un point de vue temporel. Vu la taille importante du jeu de données, un test de validation unique a été considéré comme suffisant pour générer des statistiques de validation selon le niveau de précision requis.

La capacité de prédire des valeurs connues du TP*Pf*_2-10_ est exprimée en utilisant l’erreur moyenne comme mesure de biais général, l’erreur moyenne absolue comme mesure de la précision générale et le coefficient de corrélation comme mesure d’association linéaire [44,66]. Les valeurs absolues de ces statistiques sont présentées, ainsi que leurs valeurs exprimées, pour chaque région, par une proportion de la moyenne du TP*Pf*_2-10_ telle qu’estimée au moyen des données de validation. La capacité de prédire l’appartenance à une classe d’endémicité a été testée en recourant à la méthode statistique de l’aire sous la courbe (AUC, *Area Under Curve*) dérivée des courbes ROC (*Receiver Operating Characteristic*). Ces dernières comparent la sensibilité à un moins la spécificité pour chaque classe d’endémicité [34,67]. Les valeurs de l’AUC supérieures à 0,9 indiquent une excellente concordance entre les valeurs réelles et prédites d’appartenance à une classe; celles supérieures à 0,7, une concordance modérément bonne; et des valeurs de 0,5 indiquent que le modèle n’obtient pas de meilleurs résultats que l’attribution aléatoire à une classe [34,67]. Une procédure a également été utilisée pour vérifier dans quelle mesure les distributions prédites *a posteriori* pour chaque lieu fournissaient une mesure appropriée de l’incertitude. Cette procédure a permis de comparer la probabilité attribuée aux valeurs prédites du TP*Pf*_2-10_ en chaque lieu avec les probabilités observées dans chaque région. Des précisions complémentaires concernant cette procédure sont fournies dans le Protocole S4.2.

Les distributions de fréquence du TP*Pf*_2-10_ sont illustrées à la fois pour les données originales et pour la surface prédite par le modèle au moyen de diagrammes en forme de violon (*violin plots*) [69]. Ceux-ci fournissent une approximation lissée de la fréquence de distribution (un diagramme de noyau de densité) du TP*Pf*_2-10_ pour chaque région, et illustrent les valeurs médianes et l’intervalle interquartiles. Des diagrammes ont été produits en utilisant les données du TP*Pf*_2-10_ standardisées par âge respectivement pour toutes les années comprises dans la base de données et pour 2007 uniquement. Un autre diagramme a été réalisé en utilisant les estimations ponctuelles pour chaque point de la surface du TP*Pf*_2-10_ prédite par le modèle pour 2007.

Estimer la densité de population humaine en 2007

La version alpha du GRUMP fournit des estimations du nombre de personnes par km^2^ et de la densité de population avec une résolution spatiale de 1×1 km pour les années 1990, 1995 et 2000. Ces données sont disponibles à la fois en version ajustée et non ajustée aux estimations de population nationale fournies par les Nations Unies [60,61] (Protocole S2.3). Les données de population ajustées pour l’année 2000 ont été projetées pour 2007 en appliquant le taux national de variance moyenne et d'accroissement intercensitaire pertinent pour chaque pays [70] grâce aux méthodes décrites précédemment [71] (Protocol S2.4). Ces chiffres ont ensuite été stratifiés par pays et par groupe d’âge sur la base des structures d’âge des populations définies par les Nations Unies pour l’année 2005 [72], et ceci afin d’obtenir des surfaces correspondant aux populations de 0 à 5 ans, de 5 à 14 ans et de 15 ans et plus.

Les frontières digitalisées des 87 pays endémiques pour le paludisme causé par *P. falciparum* ont été superposées aux classes d’endémicité ajustée dans les zones urbaines (et reprojetées avec une projection équivalente). Les zones de chaque classe d’endémicité ont été extraites dans ArcView GIS 3.2 (ESRI, 1999) (Protocole S2.4). Ces couches ont également été superposées aux données du GRUMP [60,61] afin d’extraire les estimations de population à risque pour *P. falciparum* ajustées dans les zones urbaines, par classes d’âge et d’endémicité (Protocole S2.4). Enfin, ces surfaces ont été combinées avec les cartes des incertitudes pour fournir un indice d’incertitude ajusté à la population (le produit du logarithme de la densité de population et de la réciproque de la probabilité d’une attribution correcte à une classe).

**Résultats**

Précision de la carte d’endémicité prédite du paludisme causé par *P. falciparum*

La surface continue d’endémicité du paludisme causé par *P. falciparum* prédite par le modèle est illustrée à la Figure 3. La classe d’endémicité la plus probable est représentée à la Figure 4. La probabilité effective de prédire correctement chaque classe est illustrée à la Figure 5A. Une description détaillée de la précision des prédictions, de la variation régionale de cette surface selon les différents niveaux de risque stable et la PAR associée est également fournie ci-dessous. D’autres indicateurs de l’incertitude des prédictions sont décrits dans le Protocole S4.3.

Prédiction de valeurs ponctuelles du TP*Pf*_2-10_

L’analyse de l’erreur moyenne lors de la génération de l’aire d’endémicité du paludisme causé par *P. falciparum* (Figure 3) révèle une erreur systématique minime dans les valeurs prédites du TP*Pf*_2-10_. La valeur mondiale de celle-ci est de 0,91, ce qui indique une tendance générale à surestimer le TP*Pf*_2-10_ de moins de un pourcent (Amériques = 0,63; Afrique+ = 0,80; Asie CSE = 1,18) (Tableau 1). L’analyse de l’erreur moyenne absolue montre que celle-ci est de l’ordre de 9,75 (Amériques = 3,52; Afrique+ = 11,02; Asie CSE = 7,71) pour les prévisions du TP*Pf*_2-10_ (Tableau 1). Le coefficient de corrélation entre les valeurs réelles et prédites est de 0,82 à l’échelle mondiale, ce qui témoigne d’une excellente concordance linéaire. Ceci est également illustré par un diagramme de dispersion (Figure 6A et Tableau 1). Les corrélations au niveau régional pour les continents américains et l’Asie CSE sont généralement plus faibles (Amériques = 0,03; Afrique+ = 0,82; Asie CSE = 0,70) (Tableau 1). Le semi-variogramme des résidus standardisés du modèle (Figure 6B) indique une autocorrélation spatiale très faible, jusqu’à des distances d’environ deux degrés décimaux. Une comparaison avec une enveloppe nulle simulée a montré que ce n’est pas statistiquement significatif.

Prédiction de la classe d’endémicité

Les courbes ROC (*Receiver-Operating-Characteristics*) et les statistiques de l’AUC pour chaque classe d’endémicité sont illustrées à la Figure 6C et dans le Tableau 2. Les valeurs de l’AUC mondiales pour les trois classes d’endémicité dépassaient le seuil de 0,7, indiquant une distinction allant d’assez bonne à bonne. Pour les classes de TP*Pf*_2-10_ ≤5% et de TP*Pf*_2-10_ ≥40%, ces valeurs dépassent le seuil de 0,9, correspondant à une excellente distinction. A l’échelle globale, 70,8% des points sont correctement classifiés (Amériques = 80,0%; Afrique+ = 70,6%; Asie CSE = 69,9%). Seul 1,1% des points sont attribués incorrectement dans une classe non adjacente (Amériques = 0,0%; Afrique+ = 0,6%; Asie CSE = 2,5%) (Tableau 2). Pour chaque classe, un tableau de contingence complet est fourni dans le Protocole S4.3

Mesures réalistes de l’incertitude pour chaque prévision

Un diagramme probabilité-probabilité, comparant les seuils de probabilité prédite avec les probabilités observées (Figure 6D), indique une bonne correspondance générale entre ces deux mesures, ce qui suggère que le modèle donne une représentation raisonnablement fidèle de l’incertitude des prédictions en chaque point. Cependant, graphiquement, la courbe apparaît légèrement au-dessus de la ligne 1:1 pour la plupart des valeurs-seuils, et particulièrement pour les seuils de probabilité compris entre 0,00 et 0,25 environ. Cela signifie qu’un seuil de probabilité prédit de 0,1, par exemple, correspond probablement à un «seuil de probabilité réel» d’environ 0,2. Autrement dit, le modèle a tendance à sous-estimer la probabilité du TP*Pf*_2-10_ pour les valeurs peu élevées (Figure S4.1A du Protocole S4). Cette tendance pourrait avoir entraîné, alternativement, des surestimations du TP*Pf*_2-10_ dans certaines zones de faible endémicité.

Carte mondiale de l’endémicité du paludisme causé par *P. falciparum*

En 2007, l’ensemble de la zone à risque stable de paludisme causé par *P. falciparum* couvrait 29,73 millions de km^2^ répartis entre les continents américains (6,03 millions de km^2^, 20,30%), l’Afrique+ (18,17 millions de km^2^, 61,10%), et les régions d’Asie CSE (5,53 millions de km^2^, 18.60%) (Tableau 3). Nous avons précédemment estimé que, à l’échelle mondiale, 2,37 milliards de personnes vivent dans des zones à risque pour la transmission du paludisme causé par *P. falciparum*. Parmi ceux-ci, 0,98 milliard vivent dans des zones à risque instable [17,18]. Les personnes exposées à un risque stable, 1,383 milliard, se trouvent en Amériques (0,041 milliard, 2.94%), en Afrique+ (0,657 milliard, 47,48%) et en Asie CSE (0,686 milliard, 49,58%) (Tableau 4 et Figure 7). La variation régionale du risque stable de transmission de *P. falciparum*, stratifiée selon les classes d’endémicité faible (TP*Pf*_2-10_ ≤5%), intermédiaire (TP*Pf*_2-10_ >5-<40%) et élevée (TP*Pf*_2-10_ ≥40%) est décrite ci-dessous. En Amériques et en Asie CSE, les enfants (groupes de 0 à 4 ans et de 5 à 14 ans) représentent presque un tiers (32% pour chaque groupe) de la population totale à risque. En Afrique, cette proportion atteint 43%.

Amériques

La zone de transmission stable de *P. falciparum* en Amériques se caractérise par une endémicité uniformément faible (TP*Pf*_2-10_ ≤5%) (Figure 3 et 4). L’ensemble de la zone à risque stable couvre 6,03 millions de km^2^ principalement situés dans le bassin amazonien (Figures 3 et 4). La totalité des 40,64 millions de personnes vivant dans cette région sont exposées à un faible risque. La prévalence médiane était de 2,17%, avec des valeurs prédites de TP*Pf*_2-10_ de 0,31% et 8,81% respectivement pour les valeurs les plus faibles et les plus élevées (Figure 8C). L’examen des distributions de fréquence pour la région a montré que les valeurs prédites se distribuaient de manière plus ou moins symétrique autour de cette valeur médiane (Figure 8C). L’intervalle de valeurs pour les données originales en 2007 (Figure 8B) était similaire mais présentait une asymétrie positive, tandis que les valeurs pour l’ensemble des années s’étalaient sur un intervalle plus large (max = 21,30%) et affichaient une asymétrie positive plus marquée (Figure 8A). La probabilité d’une attribution correcte à une classe d’endémicité était élevée dans cette région (Figure S4.1A du Protocole S4), et ce principalement en raison de la relative uniformité des valeurs peu élevées du TP*Pf*_2-10_ dans les données [17,18], plutôt qu’à une quelconque structure spatiale marquée (Figure S1.1 du Protocole S1). En raison des densités de population relativement faibles dans cette région, ce résultat correspond aux valeurs les plus basses de l’indice de fiabilité pondéré par la population (Figure 5B).

Afrique, Yémen et Arabie saoudite (Afrique+)

La zone de transmission stable de *P. falciparum* en Afrique+ couvre 18,17 millions de km^2^. 656,61 millions de personnes se trouvent dans cette zone à risque qui couvre un large éventail d’intensités de transmission. Dans cette région, 114,50 millions de personnes (17,44%) font face à un TP*Pf*_2-10_ ≤5%. Ces intensités de transmission faibles couvrent plus de 4,03 millions de km^2^ et se situent dans les zones centrales et orientales des latitudes les plus méridionales et septentrionales (Figure 3 et 4). Cette classe d’endémicité a été prédite avec une relativement bonne certitude (Figure S4.1A du Protocole S4). Les régions à fort taux de transmission avec un TP*Pf*_2-10_ ≥40% couvrent la majeure partie de l’Afrique de l’Ouest et de larges zones d’Afrique centrale, soit 8,5 millions de km^2^. 345,28 millions de personnes y sont à risque. La probabilité de prédiction d’une classe d’endémicité correcte était élevée en Afrique de l’Ouest mais beaucoup plus faible en Afrique centrale (Figure S4.1C du Protocole S4) en raison de l’abondance relative des données d’étude du TP*Pf*_2-10_ dans la première et de leur rareté dans la seconde (Figure 1). Une partie non négligeable du continent (5,63 millions de km^2^) présente des valeurs d’endémicité intermédiaires, TP*Pf*_2-10_ >5% et <40%. La PAR est de 196,83 millions d’individus. C’est cette classe d’endémicité qui a été prédite avec fiabilité la plus faible (Figure S4.1B du Protocole S4).

Au sein de la zone d’endémicité stable sur ce continent, la prévalence médiane prédite était de 33,34%. Les valeurs prédites du TP*Pf*_2-10_ les étaient de 0,20% et de 75,40% respectivement pour les valeurs les plus faibles et les plus élevées (Figure 8C). La distribution de fréquence des valeurs prédites (Figure 8) était centrée sur cette valeur médiane, avec un mode secondaire nettement moins prononcé, centré aux alentours de 15% (Figure 8C). Cette distribution était très différente de celle observée pour toutes les années et pour les données de 2007, celles-ci étant toutes deux positivement asymétriques avec des valeurs maximales respectives de 99,78% et 98,70% (Figure 8A et 8B, respectivement). L’indice d’incertitude, pondéré par la population, indique des valeurs particulièrement élevées d’une part en Éthiopie pour la classe d’endémicité la plus basse et d’autre part au Nigeria pour la classe d’endémicité la plus élevée (Figure 5B). Cela reflète la coexistence dans les données de faibles densités du TP*Pf*_2-10_ et d’une population importante dans chacun de ces pays.

Asie centrale et du Sud-Est (Asie CSE)

La zone de transmission stable de *P. falciparum* dans la région de l’Asie CSE se caractérise par une faible endémicité du paludisme (TP*Pf*_2-10_ ≤5%), avec des régions, limitées spatialement mais importantes sur le plan épidémiologique, dans lesquelles le risque est intermédiaire (TP*Pf*_2-10_ >5 et <40%) ou élevé (TP*Pf*_2-10_ ≥40%). Ces régions se situent par exemple dans l’état d’Orissa, l’Est de l’Inde, l’Ouest du Myanmar et les zones de faible altitude en Nouvelle-Guinée. La zone totale à risque stable couvre 5,53 millions de km^2^. La PAR s’élève à 685,65 millions de personnes, vivant principalement en Inde et en Indonésie (Figures 3 et 4). Dans cette région, 603,61 millions de personnes (88,03%), reparties sur plus de 4,72 millions de km^2^ (85,84%), ont un TP*Pf*_2-10_ ≤5%. La prévalence médiane prédite était de 9,99%, avec les valeurs de TP*Pf*_2-10_ de 0,006% et de 45,40% respectivement pour les valeurs les plus faibles et les plus élevées. La fréquence de distribution des valeurs prédites de TP*Pf*_2-10_ était positivement asymétrique (Figure 8C). La distribution de fréquence des données de 2007 s’étendait sur une gamme de valeurs similaire mais présentait une asymétrie positive plus marquée (Figure 8B). Le graphique des données sur l’ensemble des années présentait également une asymétrie positive mais couvrait une gamme de valeurs plus large, avec un maximum de 93,91% (Figure 8A). La probabilité d’attribution correcte à une classe d’endémicité était relativement élevée en Asie CSE mais l’incertitude était très élevée dans les zones situées entre les limites des classes d’endémicité peu élevée et intermédiaire (Figure S4.1A du Protocole S4). Ce facteur, combiné à la forte densité de population dans cette région, a induit les valeurs les plus élevées de l’indice d’incertitude pondéré par la population, et ce, particulièrement en Inde (Figure 5B).

**Discussion**

A notre connaissance, c’est la première fois depuis 40 ans qu’une carte contemporaine de l’endémicité du paludisme causé par *P. falciparum* à l’échelle mondiale est élaborée. Cette carte comble les principales faiblesses des cartes plus anciennes de la distribution mondiale du risque palustre décrites précédemment. Elle est unique en son genre, pour les raisons suivantes: premièrement, elle se base sur une base de données d’études malariométriques largement documentée et géographiquement extensive (protocole S1). Les données utilisées seront rendues publiques en 2009 (pour les études individuelles pour lesquelles les autorisations de diffusion ont été accordées) afin que chacun puisse les utiliser et les évaluer [1]; deuxièmement, les méthodes des MBG (Protocole S3) et les procédures de validation (Protocole S4) ont également été documentées de manière très exhaustive et le code correspondant en a été rendu public. L’intégralité du processus de cartographie peut dès lors être reproduite par ceux qui ont accès à des ressources informatiques nécessaires; troisièmement, une estimation rigoureuse de l’incertitude associée aux différents produits cartographiques a été entreprise afin que la fiabilité des résultats puisse être objectivement évaluée (Figure 5).

La situation du paludisme dans le monde en 2007

Le monde est considérablement moins impaludé que ne le laisse supposer l’examen des cartes historiques [5,14]. Cela est dû à la fois à un rétrécissement des limites spatiales de l’endémicité et à la réduction de cette dernière au sein de ces limites. Il y a en effet une transition globale frappante vers une écologie du paludisme à risque plus faible, qui sera étudiée plus en détails dans de futurs travaux.

A l’échelle mondiale, en 2007, sur le milliard trois cent quatre vingt millions de personnes exposées à un risque de paludisme stable, 0,759 milliard d’individus sont confrontés à un risque extrêmement bas de paludisme, avec un TP*Pf*_2-10_ ≤5%, dont 0,604 milliard en Asie CSE (79,55%), 0,115 milliard en Afrique+ (15,09%) et 0,041 milliard en Amériques (5,36%) (Tableau 4 et Figure 7). Ces populations vivent dans des conditions où les perspectives biologiques d’un maintien contrôlé du paludisme à des niveaux de transmission très bas sont réalistes. La perspective de l’élimination du paludisme, à long terme, y est donc envisageable [19]. Il va de soi que, par ailleurs, des recommandations spécifiques aux niveaux nationaux et sous-régionaux doivent être élaborées par une prise en compte sérieuse des facteurs environnementaux, financiers et politiques, qui ont un impact sur l’efficacité avec laquelle les plans d’intervention peuvent être mis en place [73-75]. Selon une bonne approximation, le reste de la population mondiale soumise à un risque paludéen stable est africaine: 0,197 milliard de personnes vivent dans des conditions de risque intermédiaire (TP*Pf*_2-10_ >5 et <40%) et 0,345 millions, dans des conditions de risque élevé (TP*Pf*_2-10_ ≥40%) (Tableau 4 et Figure 7). Dans les zones de risque intermédiaire, notre modèle mathématique suggère qu’une utilisation plus importante de MII pourrait permettre d’interrompre la transmission du paludisme causé par *P. falciparum*. Par contre, dans les zones de transmission élevée, la transmission du paludisme pourrait s’avérer moins vulnérable et requérir des méthodes de lutte plus agressive, basées sur la combinaison de diverses méthodes d’interventions supplémentaires et complémentaires [19,55].

Implémentation des statistiques et validation du modèle

La procédure de modélisation décrite ici constitue une application à grande échelle de techniques géostatistiques bayésiennes modernes et inclut un certain nombre de nouvelles composantes. L’intégration d’un modèle de standardisation pour l’âge a permis une assimilation cohérente de données d’étude collectées pour une large gamme d’âges, tout en prenant en compte l’incertitude introduite par cette source de variation supplémentaire. De la même manière, l’utilisation d’un cadre aléatoire entièrement spatio-temporel a permis d’intégrer des études remontant à 1985 pour prédire l’endémicité actuelle de *P. falciparum* de manière statistiquement et épidémiologiquement plausible.

La technique des MBG est extrêmement consommatrices en termes de ressources informatiques, même pour de petites prévisions. À notre connaissance, c’est la première fois que ces procédures ont été appliquées pour une maladie à l’échelle mondiale. Cette contrainte informatique a également imposé un certain nombre de limitations pour la procédure de modélisation, sans lesquelles la capacité prédictive du modèle aurait sans doute pu être améliorée. En particulier, le modèle actuel adopte une fonction de covariance et de moyenne uniques dans chaque région du monde, ce qui correspond à une hypothèse de stationnarité de second ordre au sein de chacune d’elles. Les approximations basées sur des champs aléatoires non stationnaires adoptées dans des études réalisées dans des zones plus limitées constituent des raffinements potentiels de ce modèle, mais l’application de telles méthodes à l’échelle globale est infaisable d’un point de vue informatique.

La vérification des différentes statistiques de validation a révélé que le modèle obtenait des résultats satisfaisants pour chacun des trois aspects suivants: la prédiction de valeurs ponctuelles du TP*Pf*_2-10_, la prévision de la classe d’endémicité et la vraisemblance des mesures de l’incertitude des prévisions. Compte tenu de la nature hautement versatile de l’endémicité du paludisme causé par *P. falciparum*, même sur de courtes distances, une corrélation absolue de 0,82 entre les prévisions du modèle et les données de validation, et une ampleur moyenne de l’erreur absolue de 9,75% du TP*Pf*_2-10_ fournissent des résultats d’une précision remarquable. Certains aspects des mesures de l’incertitude obtenues par le modèle ne sont pas optimaux: en particulier la tendance du modèle à sous-estimer légèrement la probabilité de TP*Pf*_2-10_ pour les valeurs très faibles. Malgré tout, étant donné la multitude de sources d’incertitude introduites et propagées dans notre cadre de modélisation, les prévisions obtenues pour cette incertitude constitue une source d’informations importante pour la création de produits finis à destination des décideurs.

Le modèle a utilisé les processus de Monte Carlo par Chaînes de Markov (MCMC) [77,78]. L’algorithme de MCMC est très puissant et constitue le seul algorithme couramment utilisé partout dans le monde et suffisamment souple sur le plan informatique pour de nombreux problèmes bayésiens. Il s’agit cependant d’un algorithme approximatif. Aucune méthode sûre n’existe pour estimer les erreurs de celui-ci. L’utilisation d’une méthode heuristique (Protocole S1.3) nous a cependant permis de déterminer que notre «erreur de Monte Carlo» était faible comparée à l’incertitude de distributions réelles obtenues *a posteriori*.

Les informations contenues dans les cartes présentées ici et l’incertitude qui leur est associée varient au sein d’une large gamme d’échelles géographiques. La variation à grande échelle de l’endémicité décrite entre régions et pays est sans équivoque et quantifiée de manière robuste. Ces cartes peuvent être utilisées directement pour la planification de la lutte contre le paludisme dans le monde entier. Néanmoins, l’utilité de ces cartes pour les gestionnaires locaux de la lutte contre le paludisme diminue au fur et à mesure que la zone d’intérêt considérée se réduit. Cela dépend cependant fortement de la disponibilité et de la densité locale des points de données. Le seuil et la mesure de l’incertitude adéquats varieront énormément en fonction des besoins de l’utilisateur final et des applications pour lesquelles ces cartes seront utilisées. Néanmoins, en règle générale, il semble que la catégorisation de l’endémicité au sein de zones inferieures aux limites administratives de premier niveau ne serait pas appropriée dans la plupart des pays.

L’étude des distributions de fréquence des données encodées pour toutes les années et pour 2007 et de l’aire prédite du TP*Pf*_2-10_ a révélé plusieurs particularités importantes. Premièrement, les données de 2007 pour chacune de nos trois régions ont montré des valeurs médianes et maximales considérablement moins élevées et une asymétrie positive plus marquée que les données pour l’ensemble de la période (comme illustré par la comparaison des Figures 8A et 8B). Deuxièmement, des différences marquées entre la distribution des valeurs de 2007 et celle des valeurs calculées à partir de l’aire du TP*Pf*_2-10_ prédite ont été observées dans chacune des régions (comme illustré par la comparaison des Figures 8B et 8C). En particulier, les distributions de ces dernières présentaient des médianes plus larges, une asymétrie positive moins marquée et des valeurs maximales nettement moins élevées en Amériques et en Afrique+. Cette tendance générale vers un TP*Pf*_2-10_ plus élevé dans les surfaces prédites peut être attribuée à l’agrégation des données d’étude dans l’espace. Cet ensemble d’études rassemblées ne constitue en effet qu’un échantillon opportuniste guidé par les motivations et les contraintes subies par une multitude d’individus, organisations et gouvernements. L’examen visuel de ces données révèle une proportion beaucoup plus importante de points dans les régions d’endémicité peu élevée que ce que l’on obtiendrait avec un échantillonnage spatial aléatoire. Par conséquent, les statistiques résumant ces données brutes présentent un biais important. En effectuant des prédictions de l’endémicité pour une surface continue, le processus des MBG a implicitement compensé cette agrégation lors de la production des cartes finales, et la distribution de fréquence résultante n’était pas biaisée de la même manière.

Le processus des MBG permet d’effectuer des prédictions pour des lieux n’ayant pas été échantillonnés en utilisant des combinaisons linéaires des données disponibles. Pour cette raison, les surfaces prédites sont inévitablement plus lisses que les données brutes à partir desquelles les prédictions sont effectuées. Une particularité de ce processus de lissage est que la gamme de valeurs extrêmes de la surface prédite est potentiellement moindre que celle des données originales. Cela explique pourquoi les distributions de fréquence pour la surface du TP*Pf*_2-10_ prédite couvrent des éventails de valeurs plus limités que ceux des données originales. Cet effet implique également que la surface prédite fournit une prédiction plus fiable de l’endémicité à l’échelle mondiale mais qu’elle représente moins fidèlement les petites variations survenant sur de petites distances.

Utilisation de covariables environnementales pour la création de cartes continues

Les effets extrêmement limitants des covariables climatiques ont été intégrés de manière exhaustive dans la définition des limites stables et instables de la transmission du paludisme causé par *P. falciparum* décrites ci-dessus [18]. La prise en compte additionnelle de covariables environnementales afin d’augmenter la complexité et d’améliorer la capacité prédictive de la cartographie de l’endémicité par les MBG est faussement attractive. En effet, cette idée se base sur le principe selon lequel la distribution et l’endémicité actuelles du paludisme correspondent plus ou moins à sa niche fondamentale [79,80]. Cette supposition n’est pas fondée du fait que la distribution mondiale du paludisme s’est singulièrement réduite [18] depuis son pic de distribution hypothétique aux environs de 1900 [14]. En outre, la mesure selon laquelle des déterminants environnementaux de la distribution actuelle reflètent cette niche fondamentale est inconnue, de même que la manière dont ces relations varient spatialement, ou par conséquent, les artefacts éventuels que leur introduction dans le modèle pourrait induire. De plus, à l'échelle mondiale, il n’est pas évident d’obtenir des covariables environnementales «adéquates» ayant la fidélité spatiale et temporelle requise [63,81]. Enfin, le degré selon lequel ces relations pourraient être biaisées par les efforts d’intervention spatialement hétérogènes est également non quantifié. Un nombre croissant de recherches indique que les effets de ces interventions sont non négligeables, qu’ils se sont accélérés depuis l’an 2000 et que leur hétérogénéité spatiale perturberait de manière substantielle toutes relations mises en évidence par un modèle [82-90]. C'est donc sans surprise qu'aucun support statistique n’a été observé pour l’incorporation de covariables climatiques [62] et environnementales basée sur la télédétection [63] (Protocole S1.7).

De part la non-inclusion de covariables environnementales dans notre cadre analytique, les cartes produites qui en dérivent sont uniquement déterminées par les données d’étude et les hypothèses du modèle. Ce choix garantit une ligne de référence parcimonieuse optimale par rapport à laquelle de futurs changements pourront être comparés.

Améliorations géostatistiques potentielles

De part l’adoption de l’approche des MBG, les motivations pour l’exclusion des études dont la taille de l’échantillon était inférieure à 50 individus se sont réduites. L’incertitude liée à la taille de l'échantillon est en effet explicitement prise en compte par le modèle (Protocole S3). Cette règle d’exclusion a été définie avant que les MBG puissent être appliquées à l'échelle mondiale. Elle sera corrigée dans les futures itérations de cette carte. Un tel exercice d’extraction de ces données était cependant logistiquement irréalisable pour la carte présentée ici.

La résolution spatiale à laquelle les techniques des MBG pourraient être raisonnablement appliquées en utilisant une grappe de serveurs était de 5×5 km. La totalité du processus a duré en moyenne un mois pour cette résolution. Nous l’avons estimée à trois mois pour une résolution spatiale de 1×1 km. Nous ne prévoyons pas d’augmenter la résolution spatiale des cartes produites à l’échelle mondiale car elles sont suffisamment robustes pour les programmes régionaux auxquels elles sont destinées. Cependant, l’obtention de résultats des MBG basés sur une résolution de 1×1 km pourraient être envisagée localement [33], par exemple pour des pays disposant de nombreuses données sur le TP*Pf* et dans lesquels des cartes basées sur une résolution spatiale plus fine pourraient être utiles pour soutenir les programmes nationaux de lutte. De plus, à l'échelle nationale, la précision du géo-positionnement des données du TP*Pf* pourrait potentiellement avoir un impact important sur l’incertitude des prévisions. Les procédures permettant d'intégrer ce paramètre mériteraient probablement d'être investiguées [91-93]. Dans la présente étude, l’incertitude potentiellement causée par des erreurs de géo-positionnement a été considérée comme insignifiante en raison de l'échelle des variations spatiales de l’endémicité observée et de l’échelle mondiale des produits du modèle obtenus.

Nous n’avons pas pu améliorer la performance prédictive du modèle de correction de l’âge ni en modélisant séparément la sensibilité des méthodes microscopiques et des tests de diagnostic rapide en fonction de l'âge, ni en modélisant la spécificité des méthodes de diagnostic. La précision de la détermination du TP*Pf* par microscopie ou des tests de diagnostic rapide a été considérée équivalente dans ces analyses, mais la sensibilité de la technique de diagnostic [94-98] pourrait être incluse dans de futures itérations utilisant ce cadre basé sur les MBG.

Aucune solution n’a pu être trouvée pour appliquer la technique des MBG à travers de larges étendues océaniques (par exemple aux Caraïbes, à Madagascar ou dans l’archipel indonésien), en raison de la distribution globale des données du TP*Pf* et de leur absence dans certaines régions (Figure 1). Les influences biogéographiques potentielles sur la transmission du paludisme dans les îles ont été ignorées pour ces analyses. Les futures itérations de notre carte devraient idéalement inclure suffisamment de données pour pouvoir traiter les îles séparément, ou incorporer suffisamment d’informations sur la distribution des *Anopheles* vecteurs pour améliorer les prédictions [56].

Nous avons intégré dans nos analyses leur capacité à intégrer d’une part, des tendances à long terme dans les données du TP*Pf* et d’autre part, des variations annuelles de transmission. Cependant, cette carte ne fournit pas une description complète des dynamiques saisonnières du paludisme [99-101], et des informations complémentaires sur les variations mondiales de la saisonnalité du paludisme pourraient améliorer les futures itérations de cette carte.

La voie à suivre: le domaine public et les cartes dynamiques

Par la publication de cet article, ces surfaces cartographiées sont mises à la disposition de tout un chacun. Les données sous-jacentes utilisées pour les prédictions seront rendues publiques dans le courant de l'année 2009 [1] et l’infrastructure en ligne qui hébergera ces données est en cours de développement. L’équipe du MAP prévoit de fournir des mises à jour annuelles de cette carte d’endémicité mondiale du paludisme causé par *P. falciparum* et de la base de données des TP*Pf* de laquelle cette carte découle. Ces mises à jour annuelles seront également nécessaires pour refléter les changements des limites spatiales, stables et instables, de la transmission palustre de *P. falciparum* [18], et ce afin de définir précisément les limites au sein desquelles les prévisions de l’endémicité doivent être réalisées. Si la communauté internationale parvient à faire reculer le paludisme, des décisions basées sur des données concrètes devront être prises concernant la discontinuité temporelle entre les limites spatiales de transmission du paludisme causé par *P. falciparum* (définies, là où c’est possible, par la moyenne de l’IPA*Pf* sur les trois années les plus récentes pour lesquelles des données sont disponibles [18]) et les données d’endémicité (TP*Pf* collectés depuis 1985).

Il est évident que la carte de prévision est un instantané de l’endémicité du paludisme en 2007 et que celle-ci change au cours du temps. Aucun degré de sophistication statistique ne peut compenser le fait que des données additionnelles augmenteraient la fiabilité de la carte produite, soit en affinant la résolution spatiale des études malariométriques, soit en actualisant les données existantes en un lieu grâce à des données plus récentes. Les méthodes utilisées ont été spécialement conçues dans le but de pouvoir actualiser rapidement ces surfaces. L’approche adoptée, principalement univariée, implique également que les modifications dans les futures itérations de cette carte pourront être basées de manière fiable sur la recherche de données additionnelles dans les zones où l’incertitude est élevée (changements dans l’espace), ou sur des changements induits par le succès de méthodes d'interventions ou le recul de la maladie (changements dans le temps), plutôt que par n’importe quel mélange spatial et temporel de la relation entre les données du TP*Pf*_2-10_ et des covariables environnementales.

Nous encourageons vivement la soumission de données supplémentaires existantes dans le but d’améliorer cette carte, particulièrement dans les zones où nous ne possédons pas ou peu d'informations, ainsi que de données récentes afin de permettre la production de futures cartes mises à jour. Les zones dans lesquelles l’incertitude est actuellement la plus élevée sont indiquées, avec une bonne approximation, par l’inverse de la probabilité de la classe prédite (Figure 5). Nous travaillons cependant à améliorer cette approximation. Par conséquent, une priorité immédiate consiste à générer des cartes régionales indiquant les sites d'échantillonnage les plus utiles pour de réduire au maximum la variance de l’aire d’endémicité actuelle et ce, avec un coût minimum. Cette approche a bien plus d'implications que la liste des zones où la variance est la plus élevée du fait que (i) chaque nouvelle étude influence la structure de la variance spatiale et a un impact sur l’emplacement idéal de l’étude suivante; (ii) le nombre et la distribution spatiale des études influencent les résultats et nécessitent de multiples simulations pour converger vers des solutions optimales et (iii) l'emplacement d’études potentielles doivent être judicieusement pondérés par la distribution de la population humaine.

Les objectifs immédiats du Malaria Atlas Project

Le MAP s'est initialement focalisé sur *P. falciparum* [1] en raison de son impact épidémiologique au niveau mondial [102] et de meilleures perspectives de contrôle ou, localement, d’élimination pour cette espèce [19]. Nous n’avons pas encore traité le problème important de l'impact de *P. vivax* [103] malgré son importance clinique de plus en plus largement reconnue [104-106]. Nous avons cependant déjà rassemblé plus de 2500 études du taux de parasitisme de *P. vivax* (TP*Pv)* pour commencer ces analyses. Un autre objectif à court terme vise à raffiner les estimations de l'impact du paludisme causé par *P. falciparum* au niveau mondial (à la fois en terme de morbidité [102] et de mortalité [48,107,108]) afin de soutenir l’estimation globale des progrès de la lutte contre la paludisme et des besoins en moyens. Les méthodes statistiques utilisées dans cette analyse permettront de représenter l’incertitude des prévisions de manière plus holistique et plus robuste dans la prochaine itération des estimations de l'impact du paludisme. À moyen terme, la combinaison des cartes mondiales de l’endémicité avec les cartes en cours d'élaboration de la distribution des *Anophèles* vecteurs dominants du paludisme humain [56], devrait permettre aux gestionnaires de la lutte contre la maladie de prendre leurs décisions sur des bases plus solides en ce qui concerne les interventions adaptées à la biologie des vecteurs locaux. À long terme, nous espérons que ces cartes ne serviront pas seulement à surveiller et à évaluer les progrès mais aussi à accroître notre capacité à modéliser l'endémicité future du paludisme et à contribuer à déterminer objectivement où il serait possible d’éliminer la maladie.

Conclusions

La situation du paludisme causé par *P. falciparum* dans le monde en 2007 constitue un défi important pour le plan d’actions de la communauté internationale [109,110], mais ces actions manquent toujours considérablement de moyens [111]. Que les pays maintiennent une lutte intensive contre le paludisme ou visent plus haut encore, c’est-à-dire son élimination [2-4,74,112-114], les moyens d’intervention sont similaires [19]. Cette ressource cartographique aidera ces pays à déterminer leurs besoins et leur servira de ligne directrice pour suivre et évaluer leurs progrès pour atteindre les objectifs internationaux. Nous souhaitons continuer à travailler en collaboration avec des individus, des pays et des régions afin d’améliorer les itérations futures de cette carte et, nous l’espérons, illustrer les succès de ces interventions.

**References**

1. Hay SI, Snow RW (2006) The Malaria Atlas Project: developing global maps of malaria risk. PLoS Med 3: e473.

2. Horton (2007) Is malaria eradication possible? Lancet 370: 1459.

3. Roberts L, Enserink M (2007) Did they really say … eradication? Science 318: 1544-1545.

4. Feachem R, Sabot O (2008) A new global malaria eradication strategy. Lancet 10: 1633-1635.

5. Lysenko AJ, Semashko IN (1968) Geography of malaria. A medico-geographic profile of an ancient disease [in Russian]. In: Lebedew AW, editor. Itogi Nauki: Medicinskaja Geografija. Moscow: Academy of Sciences, USSR. pp. 25-146.

6. Boyd MF (1930) An introduction to malariology. Cambridge, Massachusetts: Harvard University Press.

7. Pampana EJ, Russell PF (1955) Le Paludisme: problème mondial. Geneva: World Health Organization. pp. 317-321.

8. Macdonald G (1957) Local features of malaria. The epidemiology and control of malaria. London: Oxford University Press. pp. 63-99.

9. W.H.O. (1966) Malaria eradication in 1965. World Health Organ Chron 20: 286-300.

10. Dutta HM, Dutt AK (1978) Malarial ecology: a global perspective. Soc Sci Med 12: 69-84.

11. W.H.O. (1977) Information on the world malaria situation. Wkly Epidemiol Rec 52.

12. W.H.O. (1994) World malaria situation in 1992. Part II. Wkly Epidemiol Rec 69: 317-324.

13. W.H.O. (1997) World malaria situation in 1994. Part I. Wkly Epidemiol Rec 72: 269-274.

14. Hay SI, Guerra CA, Tatem AJ, Noor AM, Snow RW (2004) The global distribution and population at risk of malaria: past, present, and future. Lancet Infect Dis 4: 327-336.

15. Kiszewski A, Mellinger A, Spielman A, Malaney P, Sachs SE, et al. (2004) A global index representing the stability of malaria transmission. Am J Trop Med Hyg 70: 486-498.

16. Mouchet J, Carnevale P, Coosemans M, Julvez J, Manguin S, et al. (2004) Paludisme et grandes régions biogéographiques. Biodiversité du paludisme dans le monde. Montrouge, France: John Libbey Eurotext.

17. Guerra CA (2007) Mapping the contemporary global distribution limits of malaria using empirical data and expert opinion [Doctoral thesis]. Oxford: University of Oxford. 258 p.

18. Guerra CA, Gikandi PW, Tatem AJ, Noor AM, Smith DL, et al. (2008) The limits and intensity of *Plasmodium falciparum* transmission: implications for malaria control and elimination worldwide. PLoS Med 5: e38.

19. Hay SI, Smith DL, Snow RW (2008) Measuring malaria endemicity from intense to interrupted transmission. Lancet Infect Dis 8: 369-378.

20. Yekutiel P (1980) III The Global Malaria Eradication Campaign. In: Klingberg MA, editor. Eradication of infectious diseases: a critical study. Basel, Switzerland: Karger. pp. 34-88.

21. Pampana E (1969) A textbook of malaria eradication. London: Oxford University Press.

22. Kleinschmidt I, Bagayoko M, Clarke GPY, Craig M, Le Sueur D (2000) A spatial statistical approach to malaria mapping. Int J Epidemiol 29: 355-361.

23. Kleinschmidt I, Omumbo J, Briet O, van de Giesen N, Sogoba N, et al. (2001) An empirical malaria distribution map for West Africa. Trop Med Int Health 6: 779-786.

24. Rogers DJ, Randolph SE, Snow RW, Hay SI (2002) Satellite imagery in the study and forecast of malaria. Nature 415: 710-715.

25. Omumbo JA, Hay SI, Snow RW, Tatem AJ, Rogers DJ (2005) Modelling malaria risk in East Africa at high-spatial resolution. Trop Med Int Health 10: 557-566.

26. Rogers DJ (2006) Models for vectors and vector-borne diseases. Adv Parasitol 62: 1-35.

27. Diggle PJ, Tawn JA, Moyeed RA (1998) Model-based geostatistics. J Roy Stat Soc C-App 47: 299-326.

28. Diggle P, Moyeed R, Rowlingson B, Thomson M (2002) Childhood malaria in The Gambia: a case-study in model-based geostatistics. J Roy Stat Soc C-App 51: 493-506.

29. Gemperli A, Vounatsou P, Kleinschmidt I, Bagayoko M, Lengeler C, et al. (2004) Spatial patterns of infant mortality in Mali: the effect of malaria endemicity. Am J Epidemiol 159: 64-72.

30. Gemperli A, Vounatsou P, Sogoba N, Smith T (2006) Malaria mapping using transmission models: application to survey data from Mali. Am J Epidemiol 163: 289-297.

31. Rattanasiri S, Bohning D, Rojanavipart P, Athipanyakom S (2004) A mixture model application in disease mapping of malaria. Southeast Asian J Trop Med Public Health 35: 38-47.

32. Gosoniu L, Vounatsou P, Sogoba N, Smith T (2006) Bayesian modelling of geostatistical malaria risk data. Geospat Health 1: 127-139.

33. Noor AM, Clements ACA, Gething PW, Moloney G, Borle M, et al. (2008) Spatial prediction of *Plasmodium falciparum* prevalence in Somalia. Malar J 7: 159.

34. Clements ACA, Lwambo NJS, Blair L, Nyandindi U, Kaatano G, et al. (2006) Bayesian spatial analysis and disease mapping: tools to enhance planning and implementation of a schistosomiasis control programme in Tanzania. Trop Med Int Health 11: 490-503.

35. Clements ACA, Moyeed R, Brooker S (2006) Bayesian geostatistical prediction of the intensity of infection with *Schistosoma mansoni* in East Africa. Parasitology 133: 711-719.

36. Diggle PJ, Thomson MC, Christensen OF, Rowlingson B, Obsomer V, et al. (2007) Spatial modelling and the prediction of *Loa loa* risk: decision making under uncertainty. Ann Trop Med Parasitol 101: 499-509.

37. Beck-Worner C, Raso G, Vounatsou P, N'Goran EK, Rigo G, et al. (2007) Bayesian spatial risk prediction of *Schistosoma mansoni* infection in western Cote d'Ivoire using a remotely-sensed digital elevation model. Am J Trop Med Hyg 76: 956-963.

38. Raso G, Matthys B, N'Goran EK, Tanner M, Vounatsou P, et al. (2005) Spatial risk prediction and mapping of *Schistosoma mansoni* infections among schoolchildren living in western Cote d'Ivoire. Parasitology 131: 97-108.

39. Chilès J-P, Delfiner P (1999) Geostatistics: modeling spatial uncertainty. Toronto: John Wiley and Sons. 720 p.

40. Goovaerts P (1997) Geostatistics for natural resource evaluation. New York, U.S.A.: Oxford University Press. 483 p.

41. Matheron G (1971) The theory of regionalized variables and its applications. Fontainebleau, France: Ecole Nationale Supérieure des Mines de Paris. 211 p.

42. Lawson AB (2001) Statistical methods in spatial epidemiology. Chichester: John Wiley and Sons. 277 p.

43. Best N, Richardson S, Thomson A (2005) A comparison of Bayesian spatial models for disease mapping. Stat Methods Med Res 14: 35-59.

44. Gething PW, Noor AM, Gikandi PW, Hay SI, Nixon MS, et al. (2008) Developing geostatistical space-time models to predict outpatient treatment burdens from incomplete national data. Geogr Anal 40: 167-188.

45. Gething PW, Noor AM, Goodman CA, Gikandi PW, Hay SI, et al. (2007) Information for decision making from imperfect national data: tracking major changes in health care use in Kenya using geostatistics. BMC Med 5: 37.

46. Wang XH, Zhou XN, Vounatsou P, Chen Z, Utzinger J, et al. (2008) Bayesian spatio-temporal modeling of *Schistosoma japonicum* prevalence data in the absence of a diagnostic 'gold' standard. PLoS Negl Trop Dis 2: e250.

47. Yang GJ, Vounatsou P, Zhou XN, Tanner M, Utzinger J (2005) A Bayesian-based approach for spatio-temporal modeling of county level prevalence of *Schistosoma japonicum* infection in Jiangsu province, China. Int J Parasitol 35: 155-162.

48. Hay SI, Guerra CA, Tatem AJ, Atkinson PM, Snow RW (2005) Urbanization, malaria transmission and disease burden in Africa. Nat Rev Microbiol 3: 81-90.

49. Robert V, MacIntyre K, Keating J, Trape JF, Duchemin JB, et al. (2003) Malaria transmission in urban sub-Saharan Africa. Am J Trop Med Hyg 68: 169-176.

50. Tatem AJ, Guerra CA, Kabaria CW, Noor AM, Hay SI (2008) Human population, urban settlement patterns and their impact *Plasmodium falciparum* malaria endemicity. Malar J 7: 218.

51. Smith DL, Guerra CA, Snow RW, Hay SI (2007) Standardizing estimates of the *Plasmodium falciparum* parasite rate. Malar J 6: 131.

52. Swaroop S (1959) Statistical considerations and methodology in malaria eradication. Part I. Statistical considerations. WHO/Mal/240. Geneva: World Health Organization.

53. Swaroop S (1959) Statistical considerations and methodology in malaria eradication. Part II. Statistical methodology. WHO/Mal/240. Geneva: World Health Organization.

54. Swaroop S, Gilroy AB, Uemura K (1966) Statistical methods in malaria eradication. Geneva: World Health Organization. 164 p.

55. Smith DL, Smith T, Hay SI (2009) Measuring malaria for elimination. Chapter seven. Shrinking the malaria map: a prospectus on malaria elimination. California: Malaria Elimination Group, University of California Santa Cruz. In press.

56. Hay SI, Sinka ME, Tatem AJ, Patil AP, Guerra CA, et al. (2009) Developing global maps of the dominant *Anopheles* vectors of human malaria. PLoS Med. In press..

57. Metselaar D, Van Thiel PH (1959) Classification of malaria. Trop Geogr Med 11: 157-161.

58. Guerra CA, Hay SI, Lucioparedes LS, Gikandi PW, Tatem AJ, et al. (2007) Assembling a global database of malaria parasite prevalence for the Malaria Atlas Project. Malar J 6: 17.

59. Pull JH, Grab B (1974) Simple epidemiological model for evaluating malaria inoculation rate and risk of infection in infants. Bull World Health Organ 51: 507-516.

60. Balk DL, Deichmann U, Yetman G, Pozzi F, Hay SI, et al. (2006) Determining global population distribution: methods, applications and data. Adv Parasitol 62: 119-156.

61. CIESIN/IFPRI/WB/CIAT (2007) Global Rural Urban Mapping Project (GRUMP) alpha: Gridded Population of the World, version 2, with urban reallocation (GPW-UR). Available at http://sedac.ciesin.columbia.edu/gpw. Palisades, New York, USA: Center for International Earth Science Information Network, Columbia University / International Food Policy Research Institute / The World Bank / and Centro Internacional de Agricultura Tropical.

62. New M, Lister D, Hulme M, Makin I (2002) A high-resolution data set of surface climate over global land areas. Clim Res 21: 1-25.

63. Scharlemann JPW, Benz D, Hay SI, Purse BV, Tatem AJ, et al. (2008) Global data for ecology and epidemiology: a novel algorithm for temporal Fourier processing MODIS data. PLoS One 3: e1408.

64. Stein ML (2005) Space-time covariance functions. J Am Stat Assoc 100: 310-321.

65. Gething PW, Noor AM, Gikandi PW, Ogara EAA, Hay SI, et al. (2006) Improving imperfect data from health management information systems in Africa using space-time geostatistics. PLoS Med 3: e271.

66. Saito H, Goovaerts P (2000) Geostatistical interpolation of positively skewed and censored data in a dioxin-contaminated site. Environ Sci Technol 34: 4228-4235.

67. Brooker S, Hay SI, Bundy DA (2002) Tools from ecology: useful for evaluating infection risk models? Trends Parasitol 18: 70-74.

68. Moyeed RA, Papritz A (2002) An empirical comparison of kriging methods for nonlinear spatial point prediction. Math Geol 34: 365-386.

69. Hintze JL, Nelson RD (1998) Violin plots: a box plot-density trace synergism. Am Stat 52: 181-184.

70. U.N.P.D. (2005) World urbanization prospects: population database. http://esa.un.org/unup/. New York: United Nations Population Division.

71. Hay SI, Noor AM, Nelson A, Tatem AJ (2005) The accuracy of human population maps for public health application. Trop Med Int Health 10: 1073-1086.

72. U.N.P.D. (2006) World population prospects: the 2006 revision population database. http://esa.un.org/unpp/. New York: United Nations Population Division.

73. W.H.O. (2007) Malaria elimination: a field manual for low and moderate endemic countries. Geneva: World Health Organization. 85 p.

74. Lines J, Whitty CJM, Hanson K (2007) Prospects for eradication and elimination of malaria: a technical briefing for DFID. London: London School of Hygiene & Tropical Medicine.

75. W.H.O. (2006) Informal consultation on malaria elimination: setting up the WHO agenda. WHO/HTM/MAL/2006.1114. Geneva: World Health Organization. 68 p.

76. Raso G, Vounatsou P, Gosoniu L, Tanner M, N'Goran EK, et al. (2006) Risk factors and spatial patterns of hookworm infection among schoolchildren in a rural area of western Côte d'Ivoire. Int J Parasitol 36: 201-210.

77. Gelman A, Carlin JB, Stern HS (2003) Bayesian Data Analysis. Texts in Statistical Science. Boca Raton, Florida, U.S.A.: Chapman & Hall / CRC Press LLC. 696 p.

78. Gilks WR, Spiegelhalter DJ (1999) Markov Chain Monte Carlo in Practice. Interdisciplinary statisics. Boca Raton, Florida, U.S.A.: Chapman & Hall / CRC Press LLC.

79. Hutchinson GE (1957) Concluding remarks. Cold Spring Harbor Symp Quant Biol 22: 415-427.

80. Southwood TRE (1977) Habitat, templet for ecological strategies? Presidential address to British Ecological Society, 5 January 1977. J Anim Ecol 46: 337-365.

81. Hay SI, Tatem AJ, Graham AJ, Goetz SJ, Rogers DJ (2006) Global environmental data for mapping infectious disease distribution. Adv Parasitol 62: 37-77.

82. Barat LM (2006) Four malaria success stories: how malaria burden was successfully reduced in Brazil, Eritrea, India, and Vietnam. Am J Trop Med Hyg 74: 12-16.

83. Barnes KI, Watkins WM, White NJ (2008) Antimalarial dosing regimens and drug resistance. Trends Parasitol 24: 127-134.

84. Bhattarai A, Ali AS, Kachur SP, Martensson A, Abbas AK, et al. (2007) Impact of artemisinin-based combination therapy and insecticide-treated nets on malaria burden in Zanzibar. PLoS Med 4: e309.

85. Fegan GW, Noor AM, Akhwale WS, Cousens S, Snow RW (2007) Effect of expanded insecticide-treated bednet coverage on child survival in rural Kenya: a longitudinal study. Lancet 370: 1035-1039.

86. Noor AM, Amin AA, Akhwale WS, Snow RW (2007) Increasing coverage and decreasing inequity in insecticide-treated bed net use among rural Kenyan children. PLoS Med 4: e255.

87. Nyarango PM, Gebremeskel T, Mebrahtu G, Mufunda J, Abdulmumini U, et al. (2006) A steep decline of malaria morbidity and mortality trends in Eritrea between 2000 and 2004: the effect of combination of control methods. Malar J 5: 33.

88. Okiro EA, Hay SI, Gikandi PW, Sharif SK, Noor AM, et al. (2007) The decline in paediatric malaria admissions on the coast of Kenya. Malar J 6: 151.

89. Schellenberg D, Menendez C, Aponte J, Guinovart C, Mshinda H, et al. (2004) The changing epidemiology of malaria in Ifakara Town, southern Tanzania. Trop Med Int Health 9: 68-76.

90. Noor AM, Moloney G, Borle M, Fegan GW, Shewchuk T, et al. (2008) The use of mosquito nets and the prevalence of *Plasmodium falciparum* infection in rural South Central Somalia. PLoS One 3: e2081.

91. Chapman AD, Wieczorek J (2006) Guide to best practices for georeferencing. Copenhagen: Global Biodiversity Information Facility.

92. Guralnick RP, Wieczorek J, Beaman R, Hijmans RJ (2006) BioGeomancer: automated georeferencing to map the world's biodiversity data. PLoS Biol 4: e381.

93. Wieczorek J, Guo Q, Hijmans RJ (2004) The point-radius method for georeferencing locality descriptions and calculating associated uncertainty. Int J Geogr Inf Sci 18: 745-767.

94. O'Meara WP, Collins WE, McKenzie FE (2007) Parasite prevalence: a static measure of dynamic infections. Am J Trop Med Hyg 77: 246-249.

95. Wongsrichanalai C, Barcus MJ, Muth S, Sutamihardja A, Wernsdorfer WH (2007) A review of malaria diagnostic tools: microscopy and rapid diagnostic test (RDT). Am J Trop Med Hyg 77: 119-127.

96. Zurovac D, Midia B, Ochola SA, English M, Snow RW (2006) Microscopy and outpatient malaria case management among older children and adults in Kenya. Trop Med Int Health 11: 432-440.

97. Fogg C, Twesigye R, Batwala V, Piola P, Nabasumba C, et al. (2008) Assessment of three new parasite lactate dehydrogenase (pan-pLDH) tests for diagnosis of uncomplicated malaria. Trans R Soc Trop Med Hyg 102: 25-31.

98. Metzger WG, Vivas-Martinez S, Rodriguez I, Goncalves J, Bongard E, et al. (2008) Malaria diagnosis under field conditions in the Venezuelan Amazon. Trans R Soc Trop Med Hyg 102: 20-24.

99. Hay SI, Omumbo JA, Craig MH, Snow RW (2000) Earth observation, geographic information systems and *Plasmodium falciparum* malaria in sub-Saharan Africa. Adv Parasitol 47: 173-215.

100. Hay SI, Snow RW, Rogers DJ (1998) Predicting malaria seasons in Kenya using multitemporal meteorological satellite sensor data. Trans R Soc Trop Med Hyg 92: 12-20.

101. Hay SI, Snow RW, Rogers DJ (1998) From predicting mosquito habitat to malaria seasons using remotely sensed data: Practice, problems and perspectives. Parasitol Today 14: 306-313.

102. Snow RW, Guerra CA, Noor AM, Myint HY, Hay SI (2005) The global distribution of clinical episodes of *Plasmodium falciparum* malaria. Nature 434: 214-217.

103. Price RN, Tjitra E, Guerra CA, Yeung S, White NJ, et al. (2007) Vivax malaria: neglected and not benign. Am J Trop Med Hyg 77: 79-87.

104. Tjitra E, Anstey NM, Sugiarto P, Warikar N, Kenangalem E, et al. (2008) Multidrug-resistant *Plasmodium vivax* associated with severe and fatal malaria: a prospective study in Papua, Indonesia. PLoS Med 5: e128.

105. Rogerson SJ, Carter R (2008) Severe vivax malaria: newly recognised or rediscovered. PLoS Med 5: e136.

106. Genton B, D'Acremont V, Rare L, Baea K, Reeder JC, et al. (2008) *Plasmodium vivax* and mixed infections are associated with severe malaria in children: a prospective cohort study from Papua New Guinea. PLoS Med 5: e127.

107. Rowe AK, Rowe SY, Snow RW, Korenromp EL, Schellenberg JRA, et al. (2006) The burden of malaria mortality among African children in the year 2000. Int J Epidemiol 35: 691-704.

108. Snow RW, Craig M, Deichmann U, Marsh K (1999) Estimating mortality, morbidity and disability due to malaria among Africa's non-pregnant population. Bull World Health Organ 77: 624-640.

109. G.F.T.A.M. (2007) The Global Fund. Who we are what we do. Geneva: The Global Fund to Fight AIDS, Tuberculosis and Malaria (GFTAM). 36 p.

110. Grabowsky M (2008) The billion-dollar malaria moment. Nature 451: 1051-1052.

111. Snow RW, Guerra CA, Mutheu JJ, Hay SI (2008) International funding for malaria control in relation to populations at risk of stable *Plasmodium falciparum* transmission. PLoS Med 5: e142.

112. Tanner M, de Savigny D (2008) Malaria eradication back on the table. Bull World Health Organ 86: 82.

113. Greenwood BM, Fidock DA, Kyle DE, Kappe SH, Alonso PL, et al. (2008) Malaria: progress, perils, and prospects for eradication. J Clin Investig 118: 1266-1276.

114. R.B.M.P. (2008) The global malaria action plan for a malaria free world. Geneva, Switzerland: Roll Back Malaria Partnership (R.B.M.P), World Health Organization. 274 p.

**Remerciements**

Ce rassemblement d’études de prévalence parasitaire à l’échelle mondiale dépendait grandement des contributions de chercheurs et de personnes luttant le paludisme. Toutes ces personnes sont mentionnées sur le site web du MAP (http://www.map.ac.uk/acknowledgements.html). Nous remercions également Archie Clements pour ses commentaires sur ce manuscrit. Les auteurs remercient le *Kenyan Medical Research Institute* (KEMRI) pour son aide. Cet article est publié avec la permission du directeur du KEMRI.

**Financement**

SIH est financé par un *Senior Research Fellowship* du Wellcome Trust (#079091) qui soutient également CAG, AJT et PWG. AMN est soutenu par le Wellcome Trust en tant que *Research Training Fellow* (#081829). BHM et IRFE remercient la Li Ka Shing Foundation pour son soutien. SB est financé par le Wellcome Trust en tant que *Career Development Fellow* (#081673). RWS est un Wellcome Trust *Principal Research Fellow* (#079080). Cette subvention aide également APP. Ce travail fait partie des résultats du Malaria Atlas Project (MAP, http://www.map.ox.ac.uk), financé principalement par le Wellcome Trust (Royaume-Uni). Les donateurs n’ont joué aucun rôle dans la conception de cette étude, ni dans la collecte ou l’analyse des données, ni dans la décision de publication ou la préparation du manuscrit.

**Contributions par auteur**

SIH a écrit l’avant-projet de cet article. RWS, CAG, AMN, CWK, BHM, IRFE et SIH ont compilé et cartographié les données du TP*Pf*. SIH et RWS ont conçu les expériences. AJT, AP ET PWG ont affiné et mis en œuvre les protocoles expérimentaux. DS a conçu les procédures de standardisation de l’âge. SB et RAM ont joué le rôle de conseillers en statistiques. Tous les auteurs ont commenté la version finale du manuscrit.

**Intérêts conflictuels**

Aucun intérêt conflictuel n’a été déclaré par les auteurs.

**Abréviations**

Afrique+	Afrique, Yémen et Arabie saoudite

Asie CSE	Asie centrale et du Sud-Est

AUC	Area Under Curve

GRUMP	Global Rural-Urban Mapping Project

MII	Moustiquaire imprégnée d’insecticide

MAP	Malaria Atlas Project

MBG	Model-Based Geostatistics

MU	Masque d’urbanisation

PAR	Population à risque

IPA*Pf*	Incidence Parasitaire Annuelle de *Plasmodium falciparum*

TP*Pf*	Taux de parasitisme de *P. falciparum*

UE	Urban Extent

**Tableau 1**. Résumé des statistiques de validation utilisées pour prédire le TP*Pf*_2-10_ par région

La moyenne de chaque distribution prédite *a posteriori* a été utilisée comme estimation ponctuelle du TP*Pf*_2-10_ en comparaison avec les valeurs observées. Les valeurs entre parenthèses indiquent le pourcentage de la moyenne régionale représenté par la valeur de l’erreur correspondante. Consulter le corps du texte pour une explication complète de la dérivation de ces statistiques et l’interprétation des résultats.

doi:10.1371/journal.pmed.1000048.t001

| **Mesure de validation** | **Amériques** | **Afrique+** | **Asie CSE** | **Monde** |
| --- | --- | --- | --- | --- |
|  |  |  |  |  |
| Erreur moyenne | 0.627 (20.401) | 0.805 (3.070) | 1.184 (9.203) | 0.912 (4.267) |
| Erreur absolue moyenne | 3.522 (114.639) | 11.023 (42.024) | 7.705 (59.860) | 9.750 (45.625) |
| Corrélation | 0.032 | 0.822 | 0.699 | 0.823 |

**Tableau 2**. Résumé des statistiques de validation pour la prédiction de la classe d’endémicité du TP*Pf*_2-10_ par région

Consulter le corps du texte pour une explication complète de la dérivation de ces statistiques et l’interprétation des résultats.

doi:10.1371/journal.pmed.1000048.t002

| **Mesure de validation** | **Amériques** | **Afrique+** | **Asie CSE** | **Monde** |
| --- | --- | --- | --- | --- |
|  |  |  |  |  |
| AUC (≤5%) | 0.452 | 0.927 | 0.885 | 0.915 |
| AUC (5%-<40%) | 0.452 | 0.758 | 0.813 | 0.779 |
| AUC (≥40%) | - | 0.921 | 0.842 | 0.927 |
| % total correct | 80.000 | 70.621 | 69.874 | 70.750 |
| ≤5% classé comme ≥40% (%) | 0.000 | 0.188 | 0.418 | 0.250 |
| ≥40% classé comme ≤5% (%) | 0.000 | 0.377 | 2.092 | 0.875 |

**Tableau 3**. Zone à risque pour le paludisme causé par *P. falciparum* en 2007

Les zones sont exprimées en millions de km^2^. Risque instable (incidence parasitaire annuelle par *P. falciparum* (IPA*Pf*) <0,1 pour 1000 individus par an) et risque stable (IPA*Pf* ≥0,1 pour 1000 individus par an). Le risque stable est subdivisé en trois classes d’endémicité du TP*Pf*_2-10_ standardisées pour l’âge [51] et liées à la lutte contre le paludisme [19].

doi:10.1371/journal.pmed.1000048.t003

| **Région** | **Risque instable** | **Risque stable** | **TP*Pf*_2-10_ ≤5%** | **TP*Pf*_2-10_ >5 - <40%** | **TP*Pf*_2-10_ ≥40%** | **Total** |
| --- | --- | --- | --- | --- | --- | --- |
|  |  |  |  |  |  |  |
| Amériques | 2.13 | 6.03 | 6.03 | 0.00 | 0.00 | 8.17 |
| Afrique+ | 4.21 | 18.17 | 4.03 | 5.63 | 8.50 | 22.37 |
| Asie CSE | 3.40 | 5.53 | 4.72 | 0.69 | 0.11 | 8.93 |
| Monde | 9.74 | 29.72 | 14.79 | 6.32 | 8.61 | 39.46 |

**Tableau 4**. Population à risque (PAR) pour le paludisme causé par *P. falciparum* en 2007

Les populations sont exprimées en millions de personnes. Risque instable (incidence parasitaire annuelle par *P. falciparum* (IPA*Pf*) <0,1 pour 1000 individus par an) et risque stable (IPA*Pf* ≥0,1 pour 1000 individus par an). Le risque stable est subdivisé en trois classes d’endémicité du TP*Pf*_2-10_ standardisées pour l’âge [51] et liées à la lutte contre le paludisme [19]. Afrique, Yémen et Arabie saoudite (Afrique+) et Asie centrale et du Sud-Est (Asie CSE). Pour chaque région, la PAR est ensuite subdivisée par tranches d’âge de 0 à 4 ans, de 5 à 14 ans et de 15 ans et plus. **_†_** A noter que de très légères modifications de la population mondiale à risque, estimée auparavant à une résolution de 1×1 km, sont inévitablement générées par les contraintes informatiques, qui ont impliqué un rééchantillonnage à une résolution spatiale de 5×5 km des limites spatiales [18].

doi:10.1371/journal.pmed.1000048.t004

| **Région** | **Classe d’âge** | **Risque instable** | **Risque  stable** | **TPPf2-10 ≤5%** | **TP*Pf*_2-10_ >5 et <40%** | **TP*Pf*_2-10_ ≥40%** | **Total** |
| --- | --- | --- | --- | --- | --- | --- | --- |
|  |  |  |  |  |  |  |  |
| Amériques | 0-4 | 5.62 | 4.51 | 4.51 | 0.00 | 0.00 | 10.14 |
|  | 5-14 | 10.66 | 8.66 | 8.66 | 0.00 | 0.00 | 19.32 |
|  | 15+ | 33.78 | 27.47 | 27.47 | 0.00 | 0.00 | 61.25 |
|  | Total | 50.06 | 40.64 | 40.64 | 0.00 | 0.00 | 90.71 |
|  |  |  |  |  |  |  |  |
| Afrique+ | 0-4 | 3.29 | 108.64 | 15.48 | 32.90 | 60.26 | 111.93 |
|  | 5-14 | 5.64 | 170.80 | 25.35 | 51.48 | 93.98 | 176.44 |
|  | 15+ | 12.95 | 377.17 | 73.67 | 112.45 | 191.05 | 390.12 |
|  | Total | 21.88 | 656.61 | 114.50 | 196.83 | 345.28 | 678.49 |
|  |  |  |  |  |  |  |  |
| Asie CSE | 0-4 | 99.69 | 73.70 | 64.98 | 8.10 | 0.62 | 173.38 |
|  | 5-14 | 195.40 | 143.20 | 126.26 | 15.67 | 1.27 | 338.60 |
|  | 15+ | 616.17 | 468.75 | 412.37 | 51.52 | 4.86 | 1084.93 |
|  | Total | 911.26 | 685.65 | 603.61 | 75.29 | 6.75 | 1596.91 |
|  |  |  |  |  |  |  |  |
| Monde | 0-4 | 108.60 | 186.85 | 84.97 | 41.00 | 60.88 | 301.31 |
|  | 5-14 | 211.70 | 322.66 | 160.26 | 67.15 | 95.25 | 560.40 |
|  | 15+ | 662.90 | 873.40 | 513.51 | 163.97 | 195.91 | 1504.41 |
|  | Total | 983.20 | 1382.91 | 758.75 | 272.13 | 352.04 | 2366.11 |

**Figure 1.** Limites spatiales du risque lié au paludisme causé par *Plasmodium falciparum*. Ces limites sont définies par l’incidence parasitaire annuelle de *P. falciparum* (IPA*Pf*) combinée à des filtres d’intelligence médicale, de température et d’aridité

Les zones ont été définies comme étant à risque stable (zones en gris foncé, IPA*Pf* ≥0,1 pour 1000 individus par an), instable (zones en gris moyen, IPA*Pf* <0,1 pour 1000 individus par an) ou sans risque (zones en gris clair, IPA*Pf* =0 pour 1000 individus par an) [17-19]. Les études de prévalence de *P. falciparum* au sein des populations, menées entre le 1^er^ janvier 1985 et le 31 juillet 2008, sont illustrées. Sur les 8938 études collectées, 7953 ont satisfaits à nos critères d’inclusion pour la modélisation (voir les Méthodes et protocole S1.2) et sont représentées sur cette figure. Les données d’étude présentées sont standardisées pour l’âge [51] (TP*Pf*_2-10_) et illustrées par un gradient continu allant du jaune (0%) au rouge (100%) (voir la légende de la carte). Les lignes pointillées séparent les Amériques; l’Afrique, le Yémen et l’Arabie saoudite (Afrique+); et les régions d’Asie centrale et du Sud-Est (Asie CSE).

doi:10.1371/journal.pmed.1000048.g001

**Figure 2.** Vue d’ensemble schématique des procédures de cartographie

Les losanges bleus décrivent les données originales. Les rectangles oranges représentent les modèles et les procédures expérimentaux; S1, Protocole S1; S2, Protocole S2; S3, Protocole S3 et S4, Protocole S4. Les ellipses vertes indiquent les résultats; les lignes pointillées, des produits intermédiaires, et les lignes continues, les résultats finaux. U, zones urbaines; PU, zones périurbaines; R, zones rurales.

doi:10.1371/journal.pmed.1000048.g002

**Figure 3.** Distribution spatiale de l’endémicité du paludisme causé par *Plasmodium falciparum*

Les données correspondent aux estimations ponctuelles de la moyenne annuelle du TP*Pf*_2-10_ pour 2007, prédites par un modèle basé sur des géostatistiques (MBG), à l’intérieur des limites spatiales stables de la transmission palustre due à *P. falciparum*. Elles sont illustrées sous forme d’un gradient continu allant du jaune (0%) au rouge (100 %) (voir la légende de la carte). Le reste des continents a été défini comme étant à risque instable (zones en gris moyen, IPA*Pf* <0,1 pour 1000 individus par an), ou sans risque (zones en gris clair, IPA*Pf* =0 pour 1000 individus par an) [17-19].

doi:10.1371/journal.pmed.1000048.g003

**Figure 4.** Distribution spatiale des prédictions du TP*Pf*_2-10_ stratifiées par classe d’endémicité.

Elles sont classées selon: un risque faible (TP*Pf*_2-10_ ≤5%, rouge clair); un risque intermédiaire (TP*Pf*_2-10_ >5% et <40%, rouge moyen) et un risque élevé (TP*Pf*_2-10_ ≥40%, rouge foncé). La carte illustre la classe à laquelle le TP*Pf*_2-10_ a la plus forte probabilité prédite d’appartenir. Le reste des continents a été défini comme étant à risque instable (zones en gris moyen, IPA*Pf* <0,1 pour 1000 individus par an), ou sans risque (zones en gris clair) [17-19].

doi:10.1371/journal.pmed.1000048.g004

**Figure 5.** Cartes de l’incertitude du modèle

(A) Probabilité que le TP*Pf*_2-10_ se situe dans la classe d’endémicité à laquelle il a été assigné, cartographiée et illustrée par un gradient continu allant du jaune (0,3) au bleu (1). Toute valeur supérieure à 0,3 constitue un meilleur résultat qu’une attribution aléatoire. Le reste des continents a été défini comme étant à risque instable (zones en gris moyen, IPA*Pf* <0,1 pour 1000 individus par an), ou sans risque (zones en gris clair) [17-19].

(B) Indice d’incertitude pondéré par la population. Cet indice illustre l’importance vraisemblable de l’incertitude obtenue par le produit du logarithme de la densité de population (Protocole S2.3) et la réciproque de la probabilité d’une attribution correcte à une classe, rééchelonné de 0 à 1. L’indice est présenté pour la classe d’endémicité du TP*Pf*_2-10_ la plus probable. Voir A. pour les zones à risque instable et sans risque.

doi:10.1371/journal.pmed.1000048.g005

**Figure 6.** Diagramme de validation du modèle

(A) Diagramme de dispersion des valeurs ponctuelles du TP*Pf*_2-10_ réelles et prédites.

(B) Exemple du semi-variogramme des résidus standardisés du modèle de Pearson, estimé à des intervalles discrets (cercles) et comparé à une enveloppe de Monte Carlo (lignes pointillées) représentant l’éventail des valeurs attendues aléatoirement en l’absence d’autocorrélation spatiale.

(C) Courbes *Receiver-Operating-Characteristic* pour chaque classe d’endémicité du TP*Pf*_2-10_ (ligne noire: TP*Pf*_2-10_ ≤5%, ligne rouge: TP*Pf*_2-10_ >5% et <40%, ligne verte: TP*Pf*_2-10_ ≥40%) et statistiques associées à l’aire sous la courbe.

(D) Diagramme de probabilité-probabilité comparant les seuils de probabilité prédite avec le pourcentage réel des valeurs dépassant réellement ces seuils. Dans les diagrammes A, B et D, la ligne 1:1 (ligne pointillée) est également présentée pour référence. Voir le corps du texte pour l’explication complète des procédures de validation et l’interprétation des résultats.

doi:10.1371/journal.pmed.1000048.g006

**Figure 7.** Graphiques circulaires représentant la population à risque (PAR) pour le paludisme causé par *P. falciparum* en 2007

Les graphiques illustrent la proportion de la population vivant dans chacune des classes d’endémicité du TP*Pf*_2-10_ prédites pour les Amériques, l’Afrique+, les régions d’Asie CSE et le monde. Les graphiques sont proportionnels à la population totale dans chaque région et les segments sont colorés de manière à correspondre aux classes d’endémicité de la Figure 4.

doi:10.1371/journal.pmed.1000048.g007

**Figure 8.** Diagrammes en violon représentant les distributions de fréquence des données du TP*Pf*_2-10_ pour chaque région

(A) Pour toutes les années, (B) pour 2007, et (C) pour la surface prédite pour 2007. La largeur de chaque diagramme illustre la fréquence relative de différentes valeurs du TP*Pf*_2-10_ dans chaque région. L’arrière-plan est coloré de manière à correspondre aux classes d’endémicité de la Figure 4. La barre noire centrale indique l’écart interquartile et les cercles blancs, les valeurs médianes (voir texte).

doi:10.1371/journal.pmed.1000048.g008

**Informations complémentaires**

**Version alternative du texte - S1**.

Traduction de l’article en français par Frédéric Piel et Stéphanie Loute

Disponible à: doi:10.1371/journal.pmed.1000048.sd001 (1.04 MB DOC).

**Version alternative du texte – S2**.

Traduction de l’article en chinois par Robert Li

Disponible à: doi:10.1371/journal.pmed.1000048.sd002 (437 KB DOC).

**Version alternative du texte – S3.**

Traduction de l’article en indonésien par Iqbal R. F. Elyazar et Siti Nurlela

Disponible à: doi:10.1371/journal.pmed.1000048.sd003 (1.07 MB DOC).

**Version alternative du texte – S4**.

Traduction de l’article en vietnamien par Bui H. Manh

Disponible à: doi:10.1371/journal.pmed.1000048.sd004 (574 KB DOC).

**Version alternative du texte – S5**.

Traduction de l’article en espagnol par Carlos A. Guerra

Disponible à: doi:10.1371/journal.pmed.1000048.sd005 (774 KB DOC).

**Protocole S1**. Base de données des études malariométriques sur le taux de parasite de *Plasmodium falciparum*

S1.1 Résumé de la recherche de données et des procédures d’extraction des données

S1.2 Règles d’exclusion des données

S1.3 Standardisation pour l’âge

S1.4 Semi-variogrammes des données du TP*Pf*_2-10_ par région

S1.5 Filtre géostatistique pour la détection des observations extrêmes

S1.6 Résumé des données d’études malariométriques et statistiques descriptives

S1.7 Relations avec les covariables environnementales

**Protocole S2**. Bases de données et procédures démographiques

S2.1 Règles de classification des études sur le taux de parasitisme en zones urbaines, périurbaines et rurales

S2.2 Statut et prévalence des zones urbaines, périurbaines et rurales

S2.3 Version alpha de la surface de population du GRUMP

S2.4 Calcul des populations à risque

**Protocole S3**. Procédures pour les géostatistiques basées sur un modèle (MBG)

S3.1 Vue générale du modèle statistique

S3.2 Spécifications préliminaires

S3.3 Standardisation pour l’âge

S3.4 Détails de la mise en œuvre

S3.5 Vue générale de la création de la carte

**Protocole S4**. Procédures de validation du modèle

S4.1 Création d’un jeu de données de validation

S4.2 Procédures de test des performances du modèle

S4.3 Résultats supplémentaires
