# Supplementary material for: A World Malaria Map: Plasmodium falciparum Endemicity in 2007
Source: PLoS Med. 2009 Mar 24;6(3):e1000048. doi: 10.1371/journal.pmed.1000048 (PMC2659708; doi:10.1371/journal.pmed.1000048)
Supplement: Supplementary file 2 [file pmed.1000048.sd002.doc]

世界疟疾地图**: *2007年恶性疟原虫流行***

Simon I. Hay^1,2^*, Carlos A. Guerra^1,2^, Peter W. Gething^2,3^, Anand P. Patil^2^, Andrew J. Tatem^1,2,4,5^, Abdisalan M. Noor^1,6^, Caroline W. Kabaria^1^, Bui H. Manh^7^, Iqbal R.F. Elyazar^8^, Simon Brooker^1,9^, David L. Smith^5,10^, Rana A. Moyeed^11^, Robert W. Snow^1,6^

1 Malaria Public Health and Epidemiology Group, Centre for Geographic Medicine, Kenya Medical Research Institute (KEMRI) - University of Oxford - Wellcome Trust Collaborative Programme, Nairobi, Kenya,

2 Spatial Ecology and Epidemiology Group, Department of Zoology, University of Oxford, Oxford, United Kingdom,

3 Centre for Geographical Health Research, School of Geography, University of Southampton, Highfield, Southampton, United Kingdom,

4 Department of Geography, University of Florida, Gainesville, Florida, United States of America,

5 Emerging Pathogens Institute, University of Florida, Gainesville, Florida, United States of America,

6 Centre for Tropical Medicine, Nuffield Department of Clinical Medicine, University of Oxford, Centre for Clinical Vaccinology and Tropical Medicine (CCVTM), Oxford, United Kingdom,

7 Oxford University Clinical Research Unit, Bach Mai Hospital, National Institute of Infectious and Tropical Diseases, Ha Noi, Vietnam,

8 Eijkman-Oxford Clinical Research Unit, Jakarta, Indonesia,

9 Department of Infectious and Tropical Diseases, London School of Hygiene and Tropical Medicine, United Kingdom,

10 Department of Zoology, University of Florida, Gainesville, Florida, United States of America,

11 School of Mathematics and Statistics, University of Plymouth, Plymouth, Devon, United Kingdom

背景

有效的资源分配用来防治疟疾, 需要详细了解疟疾危害的空间分布。这正好是四十年了自从上次的全球流行疟疾地图刊登以来。这篇论文介绍了2007年世界恶性疟原虫的地理地图。

方法和结果

运用多种的搜索方法,一共得到了8938份疟原虫寄生率(PfPR)的调查。其中, 7953份通过了严格的数据逼真测试,进入了全球PfPR数据库的数据,用流行程度变量及年龄标准在2-10岁制图。用地质统计学的程序来创建一个在先前确定的恶性疟原虫传播的地理范围内疟疾流行分布范围。这些程序在贝叶斯统计框架下执行，以便对这些预测的不确定性做粗略评估。用来表述正确预测三个流行类别的概率;先前分层作为一个疟疾控制的指导。人口危险性估计，调整传播模型非洲城市传播影响, 然后推导出相关的2007年人口。

全球13.8亿人处于疟疾的危险之中。 6.9亿的人在亚洲中部和东南亚(CSE 亚洲), 6.6亿人在非洲、也门、沙特阿拉伯(非洲+) 以及0.4亿人在美洲。所有这些流行区中，美洲属于最低流行类(*Pf*PR_2-10_ ≤5%)。)亚洲中部和东南亚地区绝大多数(占88%)也属于低流行类; 11%属于中级流行类(*Pf*PR_2-10_ >5-<40%), 其余部分(1%)属于高度流行地区 (*Pf*PR_2-10_ ≥40%)。非洲高流行地区有 3.5亿人生活在高流行区。2亿人住在中等风险地区()和1.1亿人住在较低的风险地区。

结论和重要性

高强度的恶性疟原虫流行在非洲，低流行等级的均在美洲。低流行等级也存在亚洲中部和东南部，但局部地区有中度和非常罕见的高传播区。因此，控制非洲的疟疾与消灭其他地区的疟疾均有同等重大意义。2007年全球恶性疟原虫流行疟疾图是系列丛书中第一本可用监测干预过程和评估。

引言

地图是协调控制疟疾等方面必不可少的[1]。在国际防治策略中，疟疾流行的地区已考虑从疟疾的防治到疟疾的消除[2,3,4]，因此疟疾地图学将成为越来越重要的工具,它能用于计划制定、项目实施和评估干预疟疾防治措施。上一本恶性疟原虫流行全球地图发表在1968年[5]。以前的疟疾全球分布图[6,7,8,9,10]在很大程度上[11,12,13,14,15,16],都有(i)资料不完整的描述;(2) “风险”的定义有主观且缺乏专家意见(3)不确定性预测不能量化。本文所描述的新的疟疾全球分布图均克服这些缺陷。

模型的地理通用范围

全球的疟原虫传播图是通过各国报告病例数据, 以及其他医学情报，以及当地的温度和按蚊情况制作的[17,18]。研究结果将2007年全球范围恶性疟原虫传播分成了三种类型: 无风险的地理分布,不稳定风险(疟原虫寄生年发生率< 0.1(PfAPI))和稳定的风险地区(PfAPI≥0.1)。如图1所示。这个稳定-不稳定的PfAP分类是在基于统计、供应，纲领性的规划，以及PfAPI水平用来定义在全球疟疾消除行动中的活动要点[19,20,21]。

全球最大规模的疟原虫调查是用来预测疟疾流行范围内的传播。为了改进这一地理的分布，大多数取样5×5 km 范围利用ArcView GIS 3.2 (ESRI,1999)。更准确在1x1公里范围的在全球分布，有一些技术上的困难和难度。

制作疟疾流行图的方法

流行图是采用全球疟原虫的调查数据，利用一个模型来预测没有调查的区域流行值 [22,23,24,25,26]。这种模型产生的图有一个不确定性，而且量化也是疾病分布图的关键。

一些最近的研究采用了一种地理统计预测模型框架,称之为model-based geostatistics (MBG)[27]，为预测疟疾流行[28,29,30,31,32,33]其他媒介和中间宿主 [34,35,36,37,38]。MBG为预测提供了一份正式的地质统计学工具的统计解释[39,40,41]，并且允许结合贝叶斯方法的统计推断[42,43]。MBG的主要优点是严格处理塑模过程中在不同阶段的不确定[27]. 通过塑造这些不同来源的不确定的相互作用, 为每个预测位置产生概率分布, 为在预期值周围提供强劲的信区间。这样，地图将为全球疟疾流行提供一个当代标准，采用MBG技术来评估预测的区间，同时提供一个估计预测真实度 [36]。

地质统计学的一个基本原则是, 因为密度和临近的数据点的减小，预测图变得越来越不确定。当数据在不同时间, 以及不同的位置采集, 它就产生了连续的地理范围。 流行病学研究实例将地理地质统计学与时间合并不多见[44,45,46,47], 但是在该项研究中，完整的地理地质统计模型框架已发展。采用时间尺度和明确的基准并可与未来地图反复的对比。因此，地图将会提供一个明确的地理框架,用于监测和评估疟原虫疟疾。

方法

分析要点

利用现代数据库的恶性疟原虫寄生率(PfPR)调查做出一个2007年全球性的疟原虫疟疾流行，用可转换和可重复的方法粗略实现了不确定性和相关资料的预测。

其主要步骤用来定义的疟原虫全球地图在框架图2中列出。第一, 必须寻找和预处理PfPR数据以产生一个粗略的地理位置和地理数据, 以便绘图和检查潜在环境变化(协议 S1)和人类定居模式[48,49,50](协议S2)。第二，精确的PfPR数据库用来制作一个不间断、年龄标准的和城市的贝叶斯统计框架疟疾流行(S3)。第三, 广泛的验证程序用来评估流行预测和不确定指标的精确性(S4)。 最后, 产生全球性高危的疟原虫疟疾人群（PAR）估计并且可根据区域级别,依年龄档次分层。

全球疟原虫发病率数据库的组合

在所有可测量的度量标准中,疟疾寄生虫流行率(外周血寄生虫阳性比例/人口数)为绘图基础, 其敏感性包括疟原虫疟疾传播的各种程度。分层的疟疾流行频率分布建议取2(2.00)至10(9.99)岁年龄组[51], 它可指导疾病防治和用药处理的防蚊蚊帐(ITNs) [19]。最低流行程度的PfPR在2至10岁年龄组(以下简称*Pf*PR_2-10_), 相当于≤5%。这是PfPR调查要求的人口样本, 可作为准确的测量和监督，因此被受青睐 [52,53,54]。我们把中度的, 稳定传播的表示为*Pf*PR_2-10_ >5%-<40%, 因为在数学模型范围，疟疾传播的中断可以用药处理的蚊帐全面覆盖*Pf*PR_2-10_ <40%的地区[19,55]。尽管由于当地疟蚊的行为和生态而有一些不确定性[56], *Pf*PR_2-10_ <40%等级被认为是保守的基准, 因为很少仅用药蚊帐的单一措施，其他的干预措施可进一步降低传播。在高度传播区, 综合干预措施可用于流行水平在*Pf*PR_2-10_ ≥40%以上。这是用疟疾流行分类用来指导预测流行率的解释。在大多数模型中，传统流行基准不是按规定比例来考虑控制和根除措施的[19,55]。

自1985年起已开展鉴别，收集和地理定位的社区流行性寄生虫估计调查[58]。建立PfPR数据是疟疾地图项目(MAP http://www.map.ox.ac.uk) 的一个首要项目，并且在2008年7月31日完成了2007全球流行地图(协议S1.1)。在87个恶性疟疾流行国家中，78个(PfMECs)国家中已收集于8938个横向调查资料。没有纳入的国家有孟加拉国、伯利兹城、不丹、吉布提、多米尼加、圭亚那、伊朗、吉尔吉斯斯坦、巴拿马。

经过六个层次的排除(去除调查位置最大(> 100平方公里)和最小(> 25平方公里) [58]; 删除有些调查质量差, 或者不精确的地理位置,; 以及日期不详的)、仍然保留了7,991份 PfPR调查 (协议S1.2和图S1(S1.2))。

使用Pull和Grab催化转化模型的运算法则，在绘图之前，将所有疟疾的PfPR转换在2至10岁范围定上年龄标准[59]。该算法认为是候选标准程序中挑选出最好的，另处详细说明[51](协议S1.3)。

全球最后的数据集分为三个主要的地区(图1): 美洲、非洲，也门、沙特(非洲+); 以及亚洲中部和东南亚(CSE Asia) (协议S1.4)。这样的分区便于从生物地理， 昆虫学，流行病学等分别考虑[8,16], 同时保留3个地区有足够意义的分析数据。每个区域的*Pf*PR_2-10_结构数据进一步支持全球地区的划分，不完全变量图将加以说明(协议S1.4,图S1(S1.1)。

已发展绘制城市，郊区和农村的疟疾传播方式，其基本原理将在另处详细说明[50](协议S2)。总之, 所有城市范围按全球农村城市测绘工程(GRUMP)α版alpha version urban extent mask (GRUMP UE)定为 1x1公里地理块。在这些城市范围内, 依照世界人口网格第三版，那些人口密度大于1000人/平方公里的 [60,61]就绘图[48]。随后调查将城市在≥1000平方公里within GRUMP UE, 郊区在< 1000平方公里within GRUMP UE,或者乡村(GRUMP UE以外)(协议S2.2)。

利用地质统计过筛来处理农村*Pf*PR_2-10_数据 (协议。1.5)。采用不完全变量统计图来评估是否每点与附近点有显著不同，并给出他们之间的距离,提供地理变异的区域图案。这个程序鉴别了38项非城市 *Pf*PR_2-10_记录, 这些记录在进一步模拟以前被移除。这些调查的细节可向我们咨询。

最后的*Pf*PR_2-10_数据(n = 7,953)如图1所示。表格S1.2描述最后*Pf*PR_2-10_数据库的属性, 包括一个每年平均*Pf*PR_2-10_的观察期的图表(协议S1.6, 图S1(S1.3)), 提供时间是MBG模型中一种重要的变化来源。 类似关于这些数据间各种气候已初步探查[62)和遥感[63]环境变量没有直接的关系(协议S1.7, 图 S1(1.5)和图S1(S1.6))。

有一种普遍的误解, 疟疾统计调查只在高发病率区域性进行。越来越倾向于进行全国性的调查作为全国代表性的地区, 当采样是调查间日疟时，确认没有疟原虫传播, 结果调查中记录了许多零流行值。总的来说, 261份调查中119份报告美洲为零值，5307份调查中1010份报告非洲为零值，以及2385份调查中775份报告亚洲中部和东南部为零值(图1)。

统计的细节

地质统计学运算法则产生的绘图是在未抽样的地点使用线性组合可供选择的抽样中预测。在测绘图上是直观的看，把可信区间放在一个预测*Pf*PR_2-10_在未抽样位置会受到影响(i)区域调查点的分布, (2)*Pf*PR_2-10_平稳穿越空间变化的宽度 (培训数据的地理分布不均),(3) 每一个调查中的取样人数(训练数据的组成调查精确度)。MBG方法[27]使用贝叶斯统计框架将这些*Pf*PR_2-10_连续绘图所产生的因素合并[协议S3)。因为数据是在1985-2008期间的不同时期采集, 至关重要是了地理地质统计近似于框架, 同时传播和时间上数据的密度一致。年龄标准化准则在框架中合并成一个子模型, 允许其固有的误差能被估计和并适合MGB方法中应用(Protocol S3)。

2007年*Pf*PR_2-10_数据建立了各地区的贝叶斯地质统计模型, , 在每个地区被建模作。如果没有年龄标化的*Pf*PR_2-10_ 的值，亦也可在任一调查总数的疟原虫阳性值建模。这些效应可在不同年份中产生,但在同一年中比较类似而缺乏季节性。平均数,并作为一个线性函数的时间,地点,以及是否是城市,或郊区,而不是农村:。 继而利用每一个调查的中间值（记录开始和结束数月）。将城市、郊区,或农村数据分配到每个预测地点并使用改性的GRUMP (协议S2.2 resampled),代表5×5公里。无结构变量用高斯零均值和方差表示。贝叶斯推理是通过马尔科夫链蒙特卡罗生成样本的后概率的位置及在高斯领域每个数据。地点间的距离将影响圆曲率并共同对全球的曲线产生影响。样本是采用2007年平均分布在每个预测的后部的位置。每个样本的连节后,预测了地图范围使用的2007年12个月内44,65][。这些预测表示在5×5公里范围内的疟原虫传播。因此模型产出与样本分布的预测在每一个格子位置均被用于点估计,流行程度概率和标准方差估计(协议S3.4)。地理统计结果的输出如何产生不同图像也进一步提供描述方法。

模型的验证

图的适用性评估是关键，而采用一些non – trivial 描述方法(协议S4)。利用一个hold-out程序来测试模型对*Pf*PR_2-10_点值的预测能力和最有可能的流行类型。验证是通过选择分层随机抽样的百分之十的数据(n = 800), 然后从数据中取出 (协议S4.1)。模型随后利用了其余7153数据生成预测后的*Pf*PR_2-10_分布与位置并与800 取出数据的已知值做对比。与主要模型运转相比, 在2007年的年度预测, 每月预测确认运行*Pf*PR_2-10_值与每个取出调查的点相对应，以提供暂时的可比值。大量数据和单一的验证后可认为足以产生验证统计及所需的精确水平。

预测*Pf*PR_2-10_能力可使用平均误差作为测量的总偏差, 绝对平均误差作为衡量全面准确性和相关系数的线性关系[44,66]。这些统计采纳每个区域绝对值和平均*Pf*PR_2-10_的比例从验证组中计算出来。用曲线下面积(AUC)来测试预测流行类别的人数，每一个流行类型用receiver-operating-characteristic(ROC), 敏感度与特异性表示[34,67]。AUC值高于0.9表示实际和预测的成员人数完全吻合, 值高于0.7表明良好吻合，值等于0.5表示模拟操作比不上一个随机分配的好[34,67]。用一个程序 (44,68]测试在每个预测后的分布并提供了合适的测量位置不确定性。这个程序概率在每个预测点的*Pf*PR_2-10_ 的预测值与相应的每个地区的观测概率做对比。程序详情请参阅(协议S4.2)。

*Pf*PR_2-10_的频率分布需要为数据输入和预测表输出[69]。 这些图形显示平滑的*Pf*PR_2-10_频率分布近似值 (一个kernel密度图), 每个地区覆盖的中心显示着中位值和内四分位数值。用年龄标准化数据计算不同的点图*Pf*PR_2-10_,分布，在预测图上*Pf*PR_2-10_,的每个地方，用点值来估标2007年的*Pf*PR_2-10_,

2007年的人口密度估计

用调整和未调整的联合国人口估计GRUMPα版本提供了在1990，1995和2000年，1x1km地理分辨率的人口计数网格和人口密度估计。[60,61](协议S2.3)。调整后2000年的人口计数拟合到2007年，使用先前描述的方法[71](协议S2.4)。通过应用国家人口普查的增长率[70]，。这些人口计数通过全国性年龄群分层，使用联合国定义[72]的人口年龄结构： 5岁以下,5 – 14岁和15岁以上人口普查的表面。

87个恶性疟疾流行国家分布重叠在调整过的城市流行类上，使用ArcView GIS 3.2来提取每个流行类的面积(ESRI,1999) (协议S2.4)。这些分层也覆盖在GRUMP 数据中[60,61]，通过流行和年龄类来获取危险疟原虫城市调整估计人口相一致(协议S2.4)。最后这些数据与分类性地图相结合, 提供一个粗略的人口权重指数。

结果

预测疟疾流行地图的准确性

预测的疟疾流行情况如图3所示。 疟疾的流行类别的显示在图4。各类精确的实际的概率预测在图5A。不同风险水平地区的详细介绍，以及PAR精度预测描述。同时提供了预测的非量化的测量(协议S4.3)。

*Pf*PR_2-10_的点值预测

疟疾流行程度的平均误差显示，PfPR2-10的预测值与全球实际值趋向0.91比列，低估PfPR2-10的值不到1%的范围内(美洲=0.63,非洲+ = 0.80;CSE亚洲= 1.18)(表1)。绝对误差的检查显示*Pf*PR_2-10_平均误差预测为9.75(美洲=3.52, 非洲+ =11.02, CSE亚洲=7.71) (表1)。预测值与实际中全球相关系数在是0.82, 显示有良好的线性关系 (图6和表1)。就地区而言,美洲和亚洲之间相互关系较弱 (美洲= 0.03、非洲+ = 0.82、CSE亚洲= 0.70)(表1)。

流行类别的预测

各种流行类别的ROC曲线和AUC统计已在 (图6和表2)中显示。全球AUC值在三类流行类中超过0.7界限均为良好，同时满足*Pf*PR_2-10_ ≤5%和*Pf*PR_2-10_≥40%类。总的来说, 70.8%的散点可正确分类(美洲=80.0%, 非洲+ = 70.6%, 亚洲= 69.9%)，重要的是，只有1.1%的点在非临近类(美洲=0.0%, 非洲+ =0.6%, CSE亚洲= 2.5%) (表2)。一张完整的各类流行程度的列联表已被提供(协议S4.3)。

为预测提供非量化的实际措施

概率散点图将预测概率界限与观测范围概率做比较(图6)，表明相应的两个测量之间大体接近，建议模型为不确定的点预测提供一个合理的解释。 但是, 散点图在高于1:1的阈值时, 最大幅度的概率阈值为0.00至0.25之间。这意味着有一个预测概率阈值,例如,0.1,有可能涉及到一个“真实的概率阈值”0.2。换句话说, 这个模型就会倾向于较低低估*Pf*PR_2-10_的概率值(协议S4.3 图S4(S4.1A))。这种趋势可能会导致过多的估计一些低流行地区的*Pf*PR_2-10_值。

全球疟疾流行地图

2007年全球有风险的疟疾流行面积为2973万平方公里的区域, 分布在美洲(603万平方公里,占20.30%),非洲+(1817万平方公里, 占61.10%)，(CSE亚洲地区553万平方公里,18.60%)(表3)。我们曾经估计全世界有23.7亿人生活在疟疾流行区, 其中9.8亿人居住在不稳定风险地区[17,18]。 暴露在稳定风险地区人口有13.83亿人，分布在(美洲0.41亿,2.94%), 非洲+(6.57亿,47.48%)和CSE亚洲(6.86亿,49.58%)(表4和图7)。在稳定的恶性疟原虫风险变化区域内, 分层为低(*Pf*PR_2-10_≤5%),中等(*Pf*PR_2-10_> 5 - < 40%)和高(*Pf*PR_2-10_≥40%) 流行类别，这些分析如下所述。在美洲和CSE亚洲， 儿童(0-4和5-14年龄组)接近PAR总数的32%。在非洲则上升到43%。

美洲

美洲的恶性疟原虫传播的特点是低流行性(*Pf*PR_2-10_≤5%)(图3和4)。在总面积为603万平方公里的稳定风险区域, 主要分布在亚马逊河流域(图3、4)。所有4064万人在这个地区暴露于低风险。流行中位值是2.17%和最高与最低的*Pf*PR_2-10_预测值分别是0.31% 和8.81% (图8)。区域的频率分布表现出预测值分布近似均值(图8)。2007年的输入数据(图8B)显示，流行分布呈正偏态,历史资料 (最大=21.30%)并展示了显著正偏态分布(图8A)。较高正确概率的流行类分布在美洲(协议S4.3,图S4(S4.1A)), 主要由于低*Pf*PR_2-10_调查数据值 [17,18],。结合较低的地区人口密度，导致人口的加权指数的不确定性(图5B)。

非洲,也门和沙特阿拉伯(非洲+)。

非洲的疟原虫传播的区域有1817万平方公里, 其中6。5661亿人处于危险中。403万平方公里(22.18%)以上的面积和1。1450亿人(17.44%)在*Pf*PR_2-10_≤5%。这些地区位于中部和东部地区的南部与北部的大部分范围(图3、4)。流行类别能进行准确的预测(协议S4.3图(S4) S4.1A)。高传输地区*Pf*PR_2-10_≥40%主要在西非和大面积的中非，有850万平方公里, 345.28百万人处于危险之中。在西非流行类预测的正确概率明显低于中非 (协议S4.3 图S4 (S4.1C)), 是由于前者地区有相对丰富的近期*Pf*PR_2-10_调查数据而后者地区的则缺少这些数据 (图1)。 明显的内陆地区(563万平方公里)呈中级流行值*Pf*PR_2-10_>5% - <40%, 包含196.83百万的PAR。流行类别用最小的可信度预测(协议S4.3,图(S4) S4.1B)。

内陆区域的预测中值为33.34%, 其中最高和最低*Pf*PR_2-10_预期值分别为0.20%和75.40%(图8C)。预测值的频率分布(图8)集中在这个中间值, 和大约15%的次级中心(图8C)。这种分布与2007年全年的输入数据非常不同, 正面倾斜值和最大值分别为98.70% 和99.78%(图8A,和8B)。人口加权指数的不确定性展现一张复合的区域图片, 低流行类的高显值在唉索比亚和高流行类的高显值在尼日利亚(图5B), 反映了低密度的*Pf*PR_2-10_结果和各个国家人口较多。

中部, 东南亚-亚洲(CSE亚洲)

亚洲的疟原虫传播地区是属于低疟疾流行区(*Pf*PR_2-10_≤5%), 地理面积小但流行病的程度呈中(*Pf*PR_2-10_ >5-< 40%) 和高风险性(*Pf*PR_2-10_≥40%),例如, 奥里萨邦沿海国家，印度东部, 缅甸西部和低地新几内亚。危险地区覆盖了553万平方公里， 其中包括6.8565亿人口的PAR，多数位于印度和印度尼西亚(图3、4)。超过472万平方公里(85.54%)的地区和6。0363亿人口位于*Pf*PR_2-10_ ≤5%。平均流行预测为9.99%, 最高和最低的*Pf*PR_2-10_预测值分别为0.006%和45.40%。 *Pf*PR_2-10_预测值的频率分布模型呈正倾斜,(图8C)。2007年的频率分布展示了一种更为显著的正面倾斜(图8B)。对数全年数据绘图同样呈正面偏态，但是覆盖着较大范围的值，最高值达到93.91%(图8A)。正确的流行类概率较高分布在CSE 亚洲地区，但在边缘地区，介于低和中等流行类之间伴随着相当大的不确定性 (协议S4.3，图S4 (S4.1A))。 这结合高密度人口的区域, 导致人口加权指数不确定性的最高值, 特别是在印度(图5B)。

讨论

我们在过去的40年里第一次为全球范围内提供了恶性疟疾流行地图。本地图指出了先前全球疟疾危险分布地图的不足。首先, 它是基于一个大量的资料和地理学的广泛疟疾统计调查数据库(协议S1) [58], 并将发布在公众(许可个人的调查和评估), 可在2009年使用[1]。第二, MBG和方法(协议S3) 验证程序(协议S4)也被详尽的记录，相关代码提供给公众。整个绘图过程在那些有权使用必备的计算资源中间可重复。第三,对相关的不确定和绘图输出进行慎密的评估，因此对结果的可信度可以客观的评估出(图5)。

2007年世界疟疾的情况

全球已大幅度减少了疾病的传播，与历史地图[5,14]相比,同时通过压缩的地理分布,这样降低了流行程度。未来的工作应探讨将全球过渡到一个较低的风险的疟疾生态学环境中。

2007年13亿人的暴露于疾病传播区，其中 7.59亿位于低流行区 -亚洲(6.04亿)、非洲、1.1亿，美洲0.4亿。 (表4)地区和图7)。这些低流行程度的国家已有一个清除疟疾的长期目标。有些特殊地区和国家,由于后勤保障、财力环境与政治的原因影响了疟疾的控制与消除干预措施。1.9亿人位于中等风险的非洲和3.45亿人口位于高危险(PfPR2≥10 - 40%)(表4和图7)。在该地区的中度流行区、数学模型建议采用药蚊帐干预措施来中断疟疾传播,而在较高的流行地区,疟疾的传播则需要加大防治力度和相关干预措施[19,55额外补充)。

统计实施和模型验证

介绍的建模过程需要大量的贝叶斯地理统计技术,并结合新颖的方法。年龄标准化模型的合并可以得到一致的调查数据,在各式各样的调查中的年龄均可用统一标准。另外,使用一个完全的地理随机调查早在1985年纳入了预测的当代疟原虫流行程度统计学的框架。

MBG技术要求非常精确预测计算问题。据我们所知,这是第一次将这些程序应用在全球范围内的疾病。这个计算负荷也有数量的限制,可以提高预测能力。在特殊情况下,一个全球性的地区模型采用单一的均值和方差函数。采用非随机领域的小范围研究[32,76]可能代表某一部分，但作为全球性是不容易施行的。

多次模型验证评价显示三个满意方面。*Pf*PR_2−10_年龄组的预测值,流行类别和提供现实的不确定性预测。自然性可变的疟原虫流行程度可发生在很短的距离内。预测和验证的相关系数为0.82在*Pf*PR_2−10_的9.75%的绝对误差代表着未期待的水平。非确定性模型是不容乐观的。在实际情况下,用下限值来低估PfPR2-10的概率。

该模型利用马尔可夫链蒙特卡罗拟合[77,78]。是一个非常强大的算法公式,是唯一通用、计算量以及平衡算法用于许多贝叶斯问题。然而,它是一种近似算法。可用fail-proof方法计算误差。

地图上所包含的信息以及相关的不确定性变异可出现在不同的地域。流行程度范围的不同可出现在不同的地区和国家。适当的阈值和不确定测量可根据用户和应用者的目的而不同。然而,在行政级别低的地区（小面积范围内）采用流行类别或程度是不合适的。

2007年全年的录入数据的频率分布和2-10岁年龄组的资料预测能揭示总体PfPR的重要特征。首先,2007年的数据从三个区域中显示较小的中位值和极端值，与其他年份相比显示正偏态分布。其次各区域2007年的实测和预测2-10岁年龄组的值都不同。特别是2-10岁的预测值有较大的中位值和非呈正偏态，美洲和非洲的资料中位值较小。需要记住的是，应用的一组资料代表一个样本，这个资料是由个人，组织和政府提供的，这组资料的目测检查，发现相当大比例是位于低流行率的地区，样本不是随机样本，原始资料有较大的偏差。MBG改进了聚类结果地图,用同样的方法减少了频率分布的偏差。

采用线性组合调查的数据，MBG能对未抽样调查的地区进行了预测。因此预测散点线比原始散点线平滑。主要是预测值的极端值小于录入的数据。这就解释了预测的频率分布范围小于的输入数据。

使用环境变量来改进地图

环境因素对疾病传播的影响也在前面描述了。这样可望便用环境因素变量后可增强和改进MBG流行图的预测能力。尚不知环境变化的何种程度能反映传播的分布。另外，这些关系的程度也不清楚，因此干预措施无法定量。越来越多的证据支持这些干预措施有效,必须持之以恒。在使用环境因素变量的分析框架中,所获得的图形主要取决于录入的调查数据和建模的假设。这提供了最简洁的基线资料,今后流行情况的变化也可便于比较。

地理统计学上的改进

运用MBG的方法,对于调查样本小于50给予删除。这种排除规则可在MBG应用之前，而且在今后应用中要加以修改。庞大的集合数据提取在现有的模型条件下是不容许的。

实施MBG技术空间分辨率在计算机集点是5×5公里。整个过程要一个月的时间完成。如果计算机采用一个1×1公里的空间网格则需要3个月完成。目前还没有计划来提高全球的空间分辨率。在较小的区域,如PfPR数据国家可望提高空间分辨率来支持国家防治计划。然而,MBG输出可考虑到1×1公里的网格。准确性的录入PfPR 地理位置和调查数据对预测的不确定度有较大影响。他们这些影响的建模程序可能需要进一步调查[91,92,93]。本研究中,地理位置的不准确性可能被认为是应用比例的不同而造成的。

我们不能改善年龄校正模型的预测性。PfPR的准确性和快速诊断测试或通过显微镜的识别是有关的,但是这些诊断技术的灵敏度可以包括[94,95,96,97,98]在今后的MBG框架。

如果全球数据或某地区数据缺乏，则没能找到解决MBG技术能应用在大片的海洋的困难(例如在加勒比地区,马达加斯加和印度尼西亚群岛)。分析中已发现，忽视了岛屿生态地质上对疟疾传播的影响，今后的图形应有足够的资料来预测岛屿的疾病传播。

我们已经分析长期趋势变化的数据, 但图形没有提供季节性动态关系，今后应注意全球疟疾季节性变化。

前进的方向:公众和动态的地图

预测图和本文均可得到查阅。用于预测的基本数据将于2009年得到。MAP团队预期能提供全球疾病流行的最新进展和数据，数据的定期更新需要反映疟疾传播的动态，以便准确的提供预测界值。如果国际地区能够成功的防治疟疾，就要求做出临时中断疟疾传播的空间界值（在近三年的平均PfAPI值）。

很明显,预测图代表snap-shot 2007年度的疟疾动态。要提高统计上的可靠性可以增加疾病的其他调查或可提供新的测量数据。设计的方法,已有建议以便更新加速。采用单变量的方法也可改变今后的图形。

我们鼓励提交其他现有的数据来改善地图。新数据将用来改进最新的地图。最高的不确定性可用近似预测概率(图5),尽管将来的工作要进一步在这方面改进。一个首要任务是要产生区域地图并要有新调查，要最大限度地降低流行程度的差异和应用最低成本。这些方案都已涉及到一些高差异的地区，因为(1)每次新调查将改变空间方差的结构，影响下次调查的定位。（2）调查的空间分布和数量会影响图形的产生（3）调查的地方需要根据人口分布而权重。

　　疟疾地图集目标。

　　当初MAP项目的目的是针对恶性疟疾，这是因为全球流行病学的重要性以及可供恶性疟疾的防治和根除。尽管间日疟已日益增加了临床上的重要性，我们还没有重视间日疟的问题。已有2500次以上的PvPR调查，也开始了建模过程。另一个目的是在确定全球疟疾疾病负担的估计， (发病率和死亡率(102]],[48,107,108)用来支持全球科学家在这方面的需求。本分析中所用的统计方法有助于今后疾病负担估计的修正。应用于该分析将允许更新负担的评估,更能代表估计可靠性与整体的不确定性预测。从中期规划而言,流行程度地图与疟疾传播按蚊的分布有助于疟疾工作者做出更明智的决策。从长远看来,我们希望不仅是用这些地图集来监测和评估防治进展,更重要的是增加我们今后建模的能力和支持全球某地区有可能根除疟疾的客观性评价。

　　结论

2007年全球疟疾形势显示需求进一步的国际共同努力。但是这些活动的资源任然相当缺乏。是否全民行动，还是加大防治力度，或力争消除疟疾，防治途径都是相同。地图的资源有助于确定需要，作为检测的基线，防治目标的进展评估。我们希望与各国家同仁共同努力，进一步改进本图集，促进防治目标的成功。

**参考文献**

1. Hay SI, Snow RW (2006) The Malaria Atlas Project: developing global maps of malaria risk. PLoS Med 3: e473. doi:10.1371/journal.pmed.0030473

2. Horton (2007) Is malaria eradication possible? Lancet 370: 1459.

3. Roberts L, Enserink M (2007) Did they really say … eradication? Science 318: 1544-1545.

4. Feachem R, Sabot O (2008) A new global malaria eradication strategy. Lancet 10: 1633-1635.

5. Lysenko AJ, Semashko IN (1968) Geography of malaria. A medico-geographic profile of an ancient disease [in Russian]. Lebedew AW, editor. Itogi Nauki: Medicinskaja Geografija. Moscow: Academy of Sciences, USSR. pp. 25-146.

6. Boyd MF (1930) An introduction to malariology. Cambridge (Massachusetts): Harvard University Press.

7. Pampana EJ, Russell PF (1955) Le paludisme: problème mondial. Geneva: World Health Organization. pp. 317-321.

8. Macdonald G (1957) Local features of malaria. The epidemiology and control of malaria. London: Oxford University Press. pp. 63-99.

9. WHO (1966) Malaria eradication in 1965. World Health Organ Chron 20: 286-300.

10. Dutta HM, Dutt AK (1978) Malarial ecology: a global perspective. Soc Sci Med 12: 69-84.

11. WHO (1977) Information on the world malaria situation. Wkly Epidemiol Rec 52.

12. WHO (1994) World malaria situation in 1992. Part II. Wkly Epidemiol Rec 69: 317-324.

13. WHO (1997) World malaria situation in 1994. Part I. Wkly Epidemiol Rec 72: 269-274.

14. Hay SI, Guerra CA, Tatem AJ, Noor AM, Snow RW (2004) The global distribution and population at risk of malaria: past, present, and future. Lancet Infect Dis 4: 327-336.

15. Kiszewski A, Mellinger A, Spielman A, Malaney P, Sachs SE, et al. (2004) A global index representing the stability of malaria transmission. Am J Trop Med Hyg 70: 486-498.

16. Mouchet J, Carnevale P, Coosemans M, Julvez J, Manguin S, et al. (2004) Paludisme et grandes régions biogéographiques. Biodiversité du paludisme dans le monde. Montrouge (France): John Libbey Eurotext.

17. Guerra CA (2007) Mapping the contemporary global distribution limits of malaria using empirical data and expert opinion [Doctoral thesis]. Oxford: University of Oxford. 258 p.

18. Guerra CA, Gikandi PW, Tatem AJ, Noor AM, Smith DL, et al. (2008) The limits and intensity of *Plasmodium falciparum* transmission: implications for malaria control and elimination worldwide. PLoS Med 5: e38. doi:10.1371/journal.pmed.0050038

19. Hay SI, Smith DL, Snow RW (2008) Measuring malaria endemicity from intense to interrupted transmission. Lancet Infect Dis 8: 369-378.

20. Yekutiel P (1980) III The Global Malaria Eradication Campaign. Klingberg MA, editor. Eradication of infectious diseases: a critical study. Basel: Karger. pp. 34-88.

21. Pampana E (1969) A textbook of malaria eradication. London: Oxford University Press.

22. Kleinschmidt I, Bagayoko M, Clarke GPY, Craig M, Le Sueur D (2000) A spatial statistical approach to malaria mapping. Int J Epidemiol 29: 355-361.

23. Kleinschmidt I, Omumbo J, Briet O, van de Giesen N, Sogoba N, et al. (2001) An empirical malaria distribution map for West Africa. Trop Med Int Health 6: 779-786.

24. Rogers DJ, Randolph SE, Snow RW, Hay SI (2002) Satellite imagery in the study and forecast of malaria. Nature 415: 710-715.

25. Omumbo JA, Hay SI, Snow RW, Tatem AJ, Rogers DJ (2005) Modelling malaria risk in East Africa at high-spatial resolution. Trop Med Int Health 10: 557-566.

26. Rogers DJ (2006) Models for vectors and vector-borne diseases. Adv Parasitol 62: 1-35.

27. Diggle PJ, Tawn JA, Moyeed RA (1998) Model-based geostatistics. J Roy Stat Soc C-App 47: 299-326.

28. Diggle P, Moyeed R, Rowlingson B, Thomson M (2002) Childhood malaria in The Gambia: a case-study in model-based geostatistics. J Roy Stat Soc C-App 51: 493-506.

29. Gemperli A, Vounatsou P, Kleinschmidt I, Bagayoko M, Lengeler C, et al. (2004) Spatial patterns of infant mortality in Mali: the effect of malaria endemicity. Am J Epidemiol 159: 64-72.

30. Gemperli A, Vounatsou P, Sogoba N, Smith T (2006) Malaria mapping using transmission models: application to survey data from Mali. Am J Epidemiol 163: 289-297.

31. Rattanasiri S, Bohning D, Rojanavipart P, Athipanyakom S (2004) A mixture model application in disease mapping of malaria. Southeast Asian J Trop Med Public Health 35: 38-47.

32. Gosoniu L, Vounatsou P, Sogoba N, Smith T (2006) Bayesian modelling of geostatistical malaria risk data. Geospat Health 1: 127-139.

33. Noor AM, Clements ACA, Gething PW, Moloney G, Borle M, et al. (2008) Spatial prediction of *Plasmodium falciparum* prevalence in Somalia. Malar J 7: 159.

34. Clements ACA, Lwambo NJS, Blair L, Nyandindi U, Kaatano G, et al. (2006) Bayesian spatial analysis and disease mapping: tools to enhance planning and implementation of a schistosomiasis control programme in Tanzania. Trop Med Int Health 11: 490-503.

35. Clements ACA, Moyeed R, Brooker S (2006) Bayesian geostatistical prediction of the intensity of infection with *Schistosoma mansoni* in East Africa. Parasitology 133: 711-719.

36. Diggle PJ, Thomson MC, Christensen OF, Rowlingson B, Obsomer V, et al. (2007) Spatial modelling and the prediction of *Loa loa* risk: decision making under uncertainty. Ann Trop Med Parasitol 101: 499-509.

37. Beck-Worner C, Raso G, Vounatsou P, N’Goran EK, Rigo G, et al. (2007) Bayesian spatial risk prediction of *Schistosoma mansoni* infection in western Cote d’Ivoire using a remotely-sensed digital elevation model. Am J Trop Med Hyg 76: 956-963.

38. Raso G, Matthys B, N’Goran EK, Tanner M, Vounatsou P, et al. (2005) Spatial risk prediction and mapping of *Schistosoma mansoni* infections among schoolchildren living in western Cote d’Ivoire. Parasitology 131: 97-108.

39. Chilès J-P, Delfiner P (1999) Geostatistics: modeling spatial uncertainty. Toronto: John Wiley and Sons. 720 p.

40. Goovaerts P (1997) Geostatistics for natural resource evaluation. New York: Oxford University Press. 483 p.

41. Matheron G (1971) The theory of regionalized variables and its applications. Fontainebleau (France): Ecole Nationale Supérieure des Mines de Paris. 211 p.

42. Lawson AB (2001) Statistical methods in spatial epidemiology. Chichester: John Wiley and Sons. 277 p.

43. Best N, Richardson S, Thomson A (2005) A comparison of Bayesian spatial models for disease mapping. Stat Methods Med Res 14: 35-59.

44. Gething PW, Noor AM, Gikandi PW, Hay SI, Nixon MS, et al. (2008) Developing geostatistical space-time models to predict outpatient treatment burdens from incomplete national data. Geogr Anal 40: 167-188.

45. Gething PW, Noor AM, Goodman CA, Gikandi PW, Hay SI, et al. (2007) Information for decision making from imperfect national data: tracking major changes in health care use in Kenya using geostatistics. BMC Med 5: 37.

46. Wang XH, Zhou XN, Vounatsou P, Chen Z, Utzinger J, et al. (2008) Bayesian spatio-temporal modeling of *Schistosoma japonicum* prevalence data in the absence of a diagnostic ‘gold’ standard. PLoS Negl Trop Dis 2: e250. doi:10.1371/journal.pntd.0000250

47. Yang GJ, Vounatsou P, Zhou XN, Tanner M, Utzinger J (2005) A Bayesian-based approach for spatio-temporal modeling of county level prevalence of *Schistosoma japonicum* infection in Jiangsu province, China. Int J Parasitol 35: 155-162.

48. Hay SI, Guerra CA, Tatem AJ, Atkinson PM, Snow RW (2005) Urbanization, malaria transmission and disease burden in Africa. Nat Rev Microbiol 3: 81-90.

49. Robert V, MacIntyre K, Keating J, Trape JF, Duchemin JB, et al. (2003) Malaria transmission in urban sub-Saharan Africa. Am J Trop Med Hyg 68: 169-176.

50. Tatem AJ, Guerra CA, Kabaria CW, Noor AM, Hay SI (2008) Human population, urban settlement patterns and their impact *Plasmodium falciparum* malaria endemicity. Malar J 7: 218.

51. Smith DL, Guerra CA, Snow RW, Hay SI (2007) Standardizing estimates of the *Plasmodium falciparum* parasite rate. Malar J 6: 131.

52. Swaroop S (1959) Statistical considerations and methodology in malaria eradication. Part I. Statistical considerations. WHO/Mal/240. Geneva: World Health Organization.

53. Swaroop S (1959) Statistical considerations and methodology in malaria eradication. Part II. Statistical methodology. WHO/Mal/240. Geneva: World Health Organization.

54. Swaroop S, Gilroy AB, Uemura K (1966) Statistical methods in malaria eradication. Geneva: World Health Organization. 164 p.

55. Smith DL, Smith T, Hay SI (2009) Measuring malaria for elimination. Chapter 7. Shrinking the malaria map: a prospectus on malaria elimination. Santa Cruz (California): Malaria Elimination Group, University of California Santa Cruz. In press.

56. Hay SI, Sinka ME, Tatem AJ, Patil AP, Guerra CA, et al. (2009) Developing global maps of the dominant *Anopheles* vectors of human malaria. PLoS Med. In press.

57. Metselaar D, Van Thiel PH (1959) Classification of malaria. Trop Geogr Med 11: 157-161.

58. Guerra CA, Hay SI, Lucioparedes LS, Gikandi PW, Tatem AJ, et al. (2007) Assembling a global database of malaria parasite prevalence for the Malaria Atlas Project. Malar J 6: 17.

59. Pull JH, Grab B (1974) Simple epidemiological model for evaluating malaria inoculation rate and risk of infection in infants. Bull World Health Organ 51: 507-516.

60. Balk DL, Deichmann U, Yetman G, Pozzi F, Hay SI, et al. (2006) Determining global population distribution: methods, applications and data. Adv Parasitol 62: 119-156.

61. Center for International Earth Science Information Network, Columbia University/International Food Policy Research Institute/The World Bank/and Centro Internacional de Agricultura Tropical (2007) Global Rural Urban Mapping Project (GRUMP) alpha: Gridded Population of the World, version 2, with urban reallocation (GPW-UR). Available: http://sedac.ciesin.columbia.edu/gpw. Palisades (New York): Center for International Earth Science Information Network, Columbia University/International Food Policy Research Institute/The World Bank/and Centro Internacional de Agricultura Tropical.

62. New M, Lister D, Hulme M, Makin I (2002) A high-resolution data set of surface climate over global land areas. Clim Res 21: 1-25.

63. Scharlemann JPW, Benz D, Hay SI, Purse BV, Tatem AJ, et al. (2008) Global data for ecology and epidemiology: a novel algorithm for temporal Fourier processing MODIS data. PLoS One 3: e1408. doi:10.1371/journal.pone.0001408

64. Stein ML (2005) Space-time covariance functions. J Am Stat Assoc 100: 310-321.

65. Gething PW, Noor AM, Gikandi PW, Ogara EAA, Hay SI, et al. (2006) Improving imperfect data from health management information systems in Africa using space-time geostatistics. PLoS Med 3: e271. doi:10.1371/journal.pmed.0030271

66. Saito H, Goovaerts P (2000) Geostatistical interpolation of positively skewed and censored data in a dioxin-contaminated site. Environ Sci Technol 34: 4228-4235.

67. Brooker S, Hay SI, Bundy DA (2002) Tools from ecology: useful for evaluating infection risk models? Trends Parasitol 18: 70-74.

68. Moyeed RA, Papritz A (2002) An empirical comparison of kriging methods for nonlinear spatial point prediction. Math Geol 34: 365-386.

69. Hintze JL, Nelson RD (1998) Violin plots: a box plot-density trace synergism. Am Stat 52: 181-184.

70. U.N.P.D) (2005) World urbanization prospects: population database. http://esa.un.org/unup/. New York: United Nations Population Division.

71. Hay SI, Noor AM, Nelson A, Tatem AJ (2005) The accuracy of human population maps for public health application. Trop Med Int Health 10: 1073-1086.

72. U.N.P.D. (2006) World population prospects: the 2006 revision population database. http://esa.un.org/unpp/. New York: United Nations Population Division.

73. WHO (2007) Malaria elimination: a field manual for low and moderate endemic countries. Geneva: World Health Organization. 85 p.

74. Lines J, Whitty CJM, Hanson K (2007) Prospects for eradication and elimination of malaria: a technical briefing for DFID. London: London School of Hygiene & Tropical Medicine.

75. WHO. (2006) Informal consultation on malaria elimination: setting up the WHO agenda. WHO/HTM/MAL/2006.1114. Geneva: World Health Organization. 68 p.

76. Raso G, Vounatsou P, Gosoniu L, Tanner M, N’Goran EK, et al. (2006) Risk factors and spatial patterns of hookworm infection among schoolchildren in a rural area of western Côte d’Ivoire. Int J Parasitol 36: 201-210.

77. Gelman A, Carlin JB, Stern HS (2003) Bayesian Data Analysis. Texts in Statistical Science. Boca Raton (Florida): Chapman & Hall/CRC Press LLC. 696 p.

78. Gilks WR, Spiegelhalter DJ (1999) Markov Chain Monte Carlo in Practice. Interdisciplinary statistics. Boca Raton (Florida): Chapman & Hall/CRC Press LLC.

79. Hutchinson GE (1957) Concluding remarks. Cold Spring Harbor Symp Quant Biol 22: 415-427.

80. Southwood TRE (1977) Habitat, templet for ecological strategies? Presidential address to British Ecological Society, 5 January 1977. J Anim Ecol 46: 337-365.

81. Hay SI, Tatem AJ, Graham AJ, Goetz SJ, Rogers DJ (2006) Global environmental data for mapping infectious disease distribution. Adv Parasitol 62: 37-77.

82. Barat LM (2006) Four malaria success stories: how malaria burden was successfully reduced in Brazil, Eritrea, India, and Vietnam. Am J Trop Med Hyg 74: 12-16.

83. Barnes KI, Watkins WM, White NJ (2008) Antimalarial dosing regimens and drug resistance. Trends Parasitol 24: 127-134.

84. Bhattarai A, Ali AS, Kachur SP, Martensson A, Abbas AK, et al. (2007) Impact of artemisinin-based combination therapy and insecticide-treated nets on malaria burden in Zanzibar. PLoS Med 4: e309. doi:10.1371/journal.pmed.0040309

85. Fegan GW, Noor AM, Akhwale WS, Cousens S, Snow RW (2007) Effect of expanded insecticide-treated bednet coverage on child survival in rural Kenya: a longitudinal study. Lancet 370: 1035-1039.

86. Noor AM, Amin AA, Akhwale WS, Snow RW (2007) Increasing coverage and decreasing inequity in insecticide-treated bed net use among rural Kenyan children. PLoS Med 4: e255. doi:10.1371/journal.pmed.0040255

87. Nyarango PM, Gebremeskel T, Mebrahtu G, Mufunda J, Abdulmumini U, et al. (2006) A steep decline of malaria morbidity and mortality trends in Eritrea between 2000 and 2004: the effect of combination of control methods. Malar J 5: 33.

88. Okiro EA, Hay SI, Gikandi PW, Sharif SK, Noor AM, et al. (2007) The decline in paediatric malaria admissions on the coast of Kenya. Malar J 6: 151.

89. Schellenberg D, Menendez C, Aponte J, Guinovart C, Mshinda H, et al. (2004) The changing epidemiology of malaria in Ifakara Town, southern Tanzania. Trop Med Int Health 9: 68-76.

90. Noor AM, Moloney G, Borle M, Fegan GW, Shewchuk T, et al. (2008) The use of mosquito nets and the prevalence of *Plasmodium falciparum* infection in rural South Central Somalia. PLoS One 3: e2081. doi:10.1371/journal.pone.0002081

91. Chapman AD, Wieczorek J (2006) Guide to best practices for georeferencing. Copenhagen: Global Biodiversity Information Facility.

92. Guralnick RP, Wieczorek J, Beaman R, Hijmans RJ (2006) BioGeomancer: automated georeferencing to map the world’s biodiversity data. PLoS Biol 4: e381. doi:10.1371/journal.pbio.0040381

93. Wieczorek J, Guo Q, Hijmans RJ (2004) The point-radius method for georeferencing locality descriptions and calculating associated uncertainty. Int J Geogr Inf Sci 18: 745-767.

94. O’Meara WP, Collins WE, McKenzie FE (2007) Parasite prevalence: a static measure of dynamic infections. Am J Trop Med Hyg 77: 246-249.

95. Wongsrichanalai C, Barcus MJ, Muth S, Sutamihardja A, Wernsdorfer WH (2007) A review of malaria diagnostic tools: microscopy and rapid diagnostic test (RDT). Am J Trop Med Hyg 77: 119-127.

96. Zurovac D, Midia B, Ochola SA, English M, Snow RW (2006) Microscopy and outpatient malaria case management among older children and adults in Kenya. Trop Med Int Health 11: 432-440.

97. Fogg C, Twesigye R, Batwala V, Piola P, Nabasumba C, et al. (2008) Assessment of three new parasite lactate dehydrogenase (pan-pLDH) tests for diagnosis of uncomplicated malaria. Trans R Soc Trop Med Hyg 102: 25-31.

98. Metzger WG, Vivas-Martinez S, Rodriguez I, Goncalves J, Bongard E, et al. (2008) Malaria diagnosis under field conditions in the Venezuelan Amazon. Trans R Soc Trop Med Hyg 102: 20-24.

99. Hay SI, Omumbo JA, Craig MH, Snow RW (2000) Earth observation, geographic information systems and *Plasmodium falciparum* malaria in sub-Saharan Africa. Adv Parasitol 47: 173-215.

100. Hay SI, Snow RW, Rogers DJ (1998) Predicting malaria seasons in Kenya using multitemporal meteorological satellite sensor data. Trans R Soc Trop Med Hyg 92: 12-20.

101. Hay SI, Snow RW, Rogers DJ (1998) From predicting mosquito habitat to malaria seasons using remotely sensed data: Practice, problems and perspectives. Parasitol Today 14: 306-313.

102. Snow RW, Guerra CA, Noor AM, Myint HY, Hay SI (2005) The global distribution of clinical episodes of *Plasmodium falciparum* malaria. Nature 434: 214-217.

103. Price RN, Tjitra E, Guerra CA, Yeung S, White NJ, et al. (2007) Vivax malaria: neglected and not benign. Am J Trop Med Hyg 77: 79-87.

104. Tjitra E, Anstey NM, Sugiarto P, Warikar N, Kenangalem E, et al. (2008) Multidrug-resistant *Plasmodium vivax* associated with severe and fatal malaria: a prospective study in Papua, Indonesia. PLoS Med 5: e128. doi:10.1371/journal.pmed.0050128

105. Rogerson SJ, Carter R (2008) Severe vivax malaria: newly recognised or rediscovered. PLoS Med 5: e136. doi:10.1371/journal.pmed.0050136

106. Genton B, D’Acremont V, Rare L, Baea K, Reeder JC, et al. (2008) *Plasmodium vivax* and mixed infections are associated with severe malaria in children: a prospective cohort study from Papua New Guinea. PLoS Med 5: e127. doi:10.1371/journal.pmed.0050127

107. Rowe AK, Rowe SY, Snow RW, Korenromp EL, Schellenberg JRA, et al. (2006) The burden of malaria mortality among African children in the year 2000. Int J Epidemiol 35: 691-704.

108. Snow RW, Craig M, Deichmann U, Marsh K (1999) Estimating mortality, morbidity and disability due to malaria among Africa’s non-pregnant population. Bull World Health Organ 77: 624-640.

109. The Global Fund to Fight AIDS, Tuberculosis and Malaria (GFTAM) (2007) The Global Fund. Who we are what we do. Geneva: The Global Fund to Fight AIDS, Tuberculosis and Malaria (GFTAM). 36 p.

110. Grabowsky M (2008) The billion-dollar malaria moment. Nature 451: 1051-1052.

111. Snow RW, Guerra CA, Mutheu JJ, Hay SI (2008) International funding for malaria control in relation to populations at risk of stable *Plasmodium falciparum* transmission. PLoS Med 5: e142. doi:10.1371/journal.pmed.0050142

112. Tanner M, de Savigny D (2008) Malaria eradication back on the table. Bull World Health Organ 86: 82.

113. Greenwood BM, Fidock DA, Kyle DE, Kappe SH, Alonso PL, et al. (2008) Malaria: progress, perils, and prospects for eradication. J Clin Investig 118: 1266-1276.

114. Roll Back Malaria Partnership (R.B.M.P) (2008) The global malaria action plan for a malaria free world. Geneva: Roll Back Malaria Partnership (R.B.M.P), World Health Organization. 274 p.

致谢

各地区疟疾的防治与研究者提供疟原虫调查和数据，这些分别在MAP网站上列出 (http://www.map.ac.uk/acknowledgements.html). 我们也要感谢Archie Clements 在原稿上的注释。作者感谢肯尼亚医学研究学院的支持(KEMRI)，这篇论文已获得KEMRI院长的许可并且发布。

资金

SIH 是由同时支持CAG, AJT, PWG的Wellcome Trust 的高级研究奖学金资助的。AMN 是Wellcome Trust 支持的研究训练伙伴。 BHM和IRFE 感谢李嘉诚基金会的支持。 SB是Wellcome Trust 资助的职业培训伙伴(#081673)。RWS Wellcome Trust 主要研究伙伴。这个资助金也是由AP支持。这个工作是疟疾地图项目结果的一部分，主要是由Wellcome Trust U.K.所资助。资金资助者没有参与任何学习设计，数据收集和分析，选择出版或者手稿准备。

作者贡献

SIH起草了原稿。RWS、CWK BHM IRFE和SIH 编辑和绘制PfPR数据。SIH和RWS构思了实验。AJT, APP和PWG 负责提炼和执行实验协议。DS 设计了年龄标准化程序。SB和RAM 扮演了的统计学顾问的角色。所有作者在最终手稿上添加了评论。

表1。

统计验证和各地区*Pf*PR_2-10_的预测

| **Validation measure** | **America** | **Africa+** | **CSE Asia** | **World** |
| --- | --- | --- | --- | --- |
|  |  |  |  |  |
| Mean error | 0.627 (20.401) | 0.805 (3.070) | 1.184 (9.203) | 0.912 (4.267) |
| Mean absolute error | 3.522 (114.639) | 11.023 (42.024) | 7.705 (59.860) | 9.750 (45.625) |
| Correlation | 0.032 | 0.822 | 0.699 | 0.823 |

表2。各地区预测*Pf*PR_2-10_流行类别的验证统计数据。

| **Validation measure** | **America** | **Africa+** | **CSE Asia** | **World** |
| --- | --- | --- | --- | --- |
|  |  |  |  |  |
| AUC (≤5%) | 0.452 | 0.927 | 0.885 | 0.915 |
| AUC (5%-<40%) | 0.452 | 0.758 | 0.813 | 0.779 |
| AUC (≥40%) | - | 0.921 | 0.842 | 0.927 |
| Overall % correct | 80.000 | 70.621 | 69.874 | 70.750 |
| ≤5% classed as ≥40% (%) | 0.000 | 0.188 | 0.418 | 0.250 |
| ≥40% classed as ≤5% (%) | 0.000 | 0.377 | 2.092 | 0.875 |

表3。2007年各地区恶性疟原虫疟疾的危害指数

| **Region** | **Unstable risk** | **Stable risk** | ***Pf*PR_2-10_** ≤**5%** | ***Pf*PR_2-10_ >5 - <40%** | ***Pf*PR_2-10_** ≥**40%** | **Total** |
| --- | --- | --- | --- | --- | --- | --- |
|  |  |  |  |  |  |  |
| America | 2.13 | 6.03 | 6.03 | 0.00 | 0.00 | 8.17 |
| Africa+ | 4.21 | 18.17 | 4.03 | 5.63 | 8.50 | 22.37 |
| CSE Asia | 3.40 | 5.53 | 4.72 | 0.69 | 0.11 | 8.93 |
| World | 9.74 | 29.72 | 14.79 | 6.32 | 8.61 | 39.46 |

表4。2007年恶性疟原虫疟疾的PAR。

| **Region** | **Unstable risk_†_** | **Stable risk_†_** | ***Pf*PR_2-10_** ≤**5%** | ***Pf*PR_2-10_ >5 - <40%** | ***Pf*PR_2-10_** ≥**40%** | **Total** |
| --- | --- | --- | --- | --- | --- | --- |
|  |  |  |  |  |  |  |
| America |  |  |  |  |  |  |
| 0-4 | 5.62 | 4.51 | 4.51 | 0.00 | 0.00 | 10.14 |
| 5-14 | 10.66 | 8.66 | 8.66 | 0.00 | 0.00 | 19.32 |
| 15+ | 33.78 | 27.47 | 27.47 | 0.00 | 0.00 | 61.25 |
| Total | 50.06 | 40.64 | 40.64 | 0.00 | 0.00 | 90.71 |
|  |  |  |  |  |  |  |
| Africa+ |  |  |  |  |  |  |
| 0-4 | 3.29 | 108.64 | 15.48 | 32.90 | 60.26 | 111.93 |
| 5-14 | 5.64 | 170.80 | 25.35 | 51.48 | 93.98 | 176.44 |
| 15+ | 12.95 | 377.17 | 73.67 | 112.45 | 191.05 | 390.12 |
| Total | 21.88 | 656.61 | 114.50 | 196.83 | 345.28 | 678.49 |
|  |  |  |  |  |  |  |
| CSE Asia |  |  |  |  |  |  |
| 0-4 | 99.69 | 73.70 | 64.98 | 8.10 | 0.62 | 173.38 |
| 5-14 | 195.40 | 143.20 | 126.26 | 15.67 | 1.27 | 338.60 |
| 15+ | 616.17 | 468.75 | 412.37 | 51.52 | 4.86 | 1084.93 |
| Total | 911.26 | 685.65 | 603.61 | 75.29 | 6.75 | 1596.91 |
|  |  |  |  |  |  |  |
| World |  |  |  |  |  |  |
| 0-4 | 108.60 | 186.85 | 84.97 | 41.00 | 60.88 | 301.31 |
| 5-14 | 211.70 | 322.66 | 160.26 | 67.15 | 95.25 | 560.40 |
| 15+ | 662.90 | 873.40 | 513.51 | 163.97 | 195.91 | 1504.41 |
| Total | 983.20 | 1382.91 | 758.75 | 272.13 | 352.04 | 2366.11 |

图1

按PfAPI确定的恶性疟疾地理分布界限。地区定义为稳定(深灰色区域, PfAPI≥0.1‰每千pa), 非稳定(中度灰色地区, PfAPI < 0.1‰每千pa)或人无风险(浅灰色, PfAPI = 0‰每千)[17,18,19]。

图2

绘图原理的步骤和方法。蓝钻石表示输入数据。橙色盒指示模型与实验程序; S1,协议S1; S2,协议S2; S3协议S3和S4,协议S4)。

图3

恶性疟疾的地理流行。这个数据是基于对2007年平均*Pf*PR_2-10_在稳定地理传播的地理统计学点的估计, 显示成为一个黄色到红色 (见图释), 剩余的面积定义为不稳定的风险(中等灰色地带, PfAPI < 0.1‰每千pa)或无风险(浅灰色,PfAPI = 0‰每千pa)[17,18,19]。

图4

按流行类别分层的恶性疟疾*Pf*PR_2-10_的地理分布[19]。低风险*Pf*PR_2-10_≤ 5%的浅红; 中度风险*Pf*PR_2-10_ > 5% - < 40%的中红和高风险*Pf*PR_2-10_≥40%的深红色。

图5

*Pf*PR_2-10_的概率分布图用黄色到蓝色从的连续表示。值高于为稳定危险区。其余的面积被定义为不稳定的风险(中等灰色地带, PfAPI < 0.1‰每千pa)或无风险(浅灰色)[17,18,19]。

图6

验证散点图。每个*Pf*PR_2-10_类的Receiver-Operating特性曲线(ROC) (黑线*Pf*PR_2-10_≤5%, 红线*Pf*PR_2-10_>5% - <40%, 绿线*Pf*PR_2-10_≥40%)和相关曲线下面积(AUC)统计。

图7

2007年恶性疟原虫疟疾危险中的人口图。图表显示了居住在美洲，非洲和CSE亚洲每个预期*Pf*PR_2-10_流行类比例的百分比。图表在每个区域与总人口成适当比例，同时用颜色分割出相符合的流行类，如图4所示。

图8

每个地区*Pf*PR_2-10_数据的频谱分布。A为全年，B为2007年，C为2007年预期表面。每一个标绘图的宽度表示每个区域内不同*Pf*PR_2-10_值的相关频率。背景用颜色来流行类相配，如图4所示。黑色中央杆表明内四分位数的范围以及白圈表示中值。

信息支持

Alternative Language Text S1.

法语翻译 -- Frédéric Piel， Stéphanie Loute

Found at doi:10.1371/journal.pmed.1000048.sd001 (1.04 MB DOC).

Alternative Language Text S2.

中文翻译 -- Robert Li

Found at doi:10.1371/journal.pmed.1000048.sd002 (437 KB DOC).

Alternative Language Text S3.

印度尼西亚语翻译 -- Iqbal R.F. Elyazar and Siti Nurlela

Found at doi:10.1371/journal.pmed.1000048.sd003 (1.07 MB DOC).

Alternative Language Text S4.

越南语翻译 -- Bui H. Manh

Found at doi:10.1371/journal.pmed.1000048.sd004 (574 KB DOC).

Alternative Language Text S5.

西班牙语翻译 -- Carlos A. Guerra

Found at doi:10.1371/journal.pmed.1000048.sd005 (774 KB DOC).

协议S1.

The *Pf*PR Malariometric Survey Database

S1.1 Summary of Data Search and Data Abstraction Procedures

S1.2 Data Exclusion Rules

S1.3 Age-Standardisation

S1.4 Semi-Variograms of *Pf*PR_2−10_ Data by Region

S1.5 Geostatistical Filter for the Detection of Extreme Outliers

S1.6 Malariometric Survey Data Summary and Descriptive Statistics

S1.7 Relationships with Environmental Covariates

Found at doi:10.1371/journal.pmed.1000048.sd005 (3.4 MB DOC).

协议S2.

Demographic Databases and Procedures

S2.1 Parasite Rate Survey Urban/Peri-Urban/Rural Classification Rules

S2.2 Urban/Peri-Urban/Rural Status and Prevalence

S2.3 GRUMP alpha Human Population Surface

S2.4 PAR Derivation

Found at doi:10.1371/journal.pmed.1000048.sd006 (2.5 MB DOC).

协议S3.

Model Based Geostatistical Procedures

S3.1 Overview of the Statistical Model

S3.2 Prior Specification

S3.3 Age-Standardization

S3.4 Implementation Details

S3.5 Overview of Map Generation

Found at doi:10.1371/journal.pmed.1000048.sd007 (23 MB DOC).

协议S4.

Model Validation Procedures

S4.1 Creation of the Validation Sets

S4.2 Procedures for Testing Model Performance

S4.3 Additional Results

Found at doi:10.1371/journal.pmed.1000048.sd008 (26 MB DOC).
