# Supplementary material for: A World Malaria Map: Plasmodium falciparum Endemicity in 2007
Source: PLoS Med. 2009 Mar 24;6(3):e1000048. doi: 10.1371/journal.pmed.1000048 (PMC2659708; doi:10.1371/journal.pmed.1000048)
Supplement: Supplementary file 3 [file pmed.1000048.sd003.doc]

**Peta Malaria Dunia: Endemisitas *Plasmodium falsiparum* tahun 2007**

Simon I. Hay^1,2*^, Carlos A. Guerra^1,2^, Peter W. Gething^2,3^, Anand P. Patil^2^, Andrew J. Tatem^1,2,4,5^, Abdisalan M. Noor^1,6^, Caroline W. Kabaria^1^, Bui H. Manh^7^, Iqbal R.F. Elyazar^8^, Simon Brooker^1,9^, David L. Smith^5,10^, Rana A. Moyeed^11^, Robert W. Snow^1,6^

**1** Malaria Public Health and Epidemiology Group, Centre for Geographic Medicine, Kenya Medical Research Institute (KEMRI) - University of Oxford - Wellcome Trust Collaborative Programme, Nairobi, Kenya,

**2** Spatial Ecology and Epidemiology Group, Department of Zoology, University of Oxford, Oxford, United Kingdom,

**3** Centre for Geographical Health Research, School of Geography, University of Southampton, Highfield, Southampton, United Kingdom,

**4** Department of Geography, University of Florida, Gainesville, Florida, United States of America,

**5** Emerging Pathogens Institute, University of Florida, Gainesville, Florida, United States of America,

**6** Centre for Tropical Medicine, Nuffield Department of Clinical Medicine, University of Oxford, Centre for Clinical Vaccinology and Tropical Medicine (CCVTM), Oxford, United Kingdom,

**7** Oxford University Clinical Research Unit, Bach Mai Hospital, National Institute of Infectious and Tropical Diseases, Ha Noi, Vietnam,

**8** Eijkman-Oxford Clinical Research Unit, Jakarta, Indonesia,

**9** Department of Infectious and Tropical Diseases, London School of Hygiene and Tropical Medicine, United Kingdom,

**10** Department of Zoology, University of Florida, Gainesville, Florida, United States of America,

**11** School of Mathematics and Statistics, University of Plymouth, Plymouth, Devon, United Kingdom

**Ringkasan**

Latar Belakang

Alokasi sumber daya secara efisien untuk mengintervensi malaria membutuhkan pemahaman rinci tentang distribusi spasial resiko malaria terbaru. Tepat empat puluh tahun sejak terakhir kalinya peta global endemisitas malaria diterbitkan. Tulisan ini menjelaskan pembuatan peta global endemisitas *Plasmodium falsiparum* yang terbaru untuk tahun 2007.

Metodologi dan Temuan

Secara keseluruhan terdapat 8,938 survey prevalensi parasit *P. falsiparum* (*Pf*PR) yang ditemukan dengan berbagai strategi pencarian yang menyeluruh. Diantaranya, 7,953 telah melewati uji keterandalan data yang ketat untuk dimasukkan ke dalam basisdata global, standarisasi umur 2-10 tahun untuk pemetaan endemisitas. Sebuah model berbasis prosedur geostatistika digunakan untuk membuat permukaan kontinu endemisitas malaria dalam batas-batas spasial transmisi stabil *P.falsiparum*. Prosedur yang digunakan adalah teknik statistika Bayesian sehingga ketidakpastian pendugaan bisa dievaluasi dengan jelas. Ketidakpastian ini dinyatakan sebagai besaran peluang menduga secara tepat satu dari tiga kelas endemisitas, yang sebelumnya distratifikasi sebagai panduan yang informatif untuk kegiatan pengendalian malaria. Dugaan terhadap jumlah penduduk beresiko, setelah disesuaikan dengan akibat urbanisasi di Afrika terhadap transmisi, diperoleh dengan merujuk kepada jumlah penduduk di tahun 2007.

Dari 1,38 milyar orang yang beresiko stabil terhadap malaria *P. falsiparum*, 0,69 milyar berada di Asia Tengah dan Tenggara (CSE Asia), 0,66 milyar di Afrika, Yaman, dan Saudi Arabia (Afrika+) dan 0,04 milyar di Amerika. Semua yang terpapar resiko stabil di Amerika tinggal di kelas endemisitas yang paling rendah (*Pf*PR_2-10_ ≤5%). Mayoritas (88%) orang yang tinggal di wilayah beresiko stabil CSE Asia juga tinggal di kelas endemisitas rendah; sebagian kecil (11%) di kelas endemisitas menengah (*Pf*PR_2-10_ >5 sampai <40%) dan sisanya (1%) di kelas endemisitas tinggi (*Pf*PR_2-10_ ≥40%). Endemisitas tinggi menyebar di wilayah Afrika+, dimana 0,35 milyar orang tinggal pada tingkat resiko tersebut. Sebagian besar sisanya berada di kelas resiko menengah (0,20 milyar), dan lebih kecil lagi (0,11 milyar) di resiko stabil rendah.

Kesimpulan

Endemisitas malaria *P.falsiparum* yang tinggi merupakan hal yang umum di Afrika. Endemisitas rendah ditemukan secara merata di Amerika. Endemisitas rendah juga menyebar di CSE Asia, namun masih terdapat kantung-kantung transmisi sedang dan transmisi tinggi walaupun sangat jarang. Oleh sebab itu, ada kesempatan penting untuk mengendalikan malaria di Afrika dan mengeliminasi malaria di kawasan lainnya. Peta global endemisitas malaria *P. falsiparum* tahun 2007 ini merupakan yang pertama dari terbitan tahunan yang dengannya bisa dimungkinkan untuk memantau dan mengevaluasi kemajuan dari proses intervensi.

Pendahuluan

Peta-peta merupakan hal yang mendasar artinya bagi seluruh aspek koordinasi pengendalian malaria [1]. Di dalam lingkungan kebijakan internasional dimana komunitas pengendalian malaria telah ditantang untuk memikirkan kembali kemungkinan eliminasi [2-4], ilmu kartografi malaria akan menjadi alat yang semakin penting untuk perencanaan, implementasi dan pengukuran dampak intervensi malaria di seluruh dunia. Peta global endemisitas untuk *Plasmodium falsiparum* yang terakhir dipublikasikan tahun 1968 [5]. Sama halnya dengan peta distribusi malaria sebelumnya [6-10], dan banyak lagi yang sesudahnya [11-16], peta tersebut (i) memiliki kelemahan dimana tidak lengkapnya penjelasan tentang masukan data yang dipergunakan; (ii) menetapkan garis “resiko” secara subjektif dan aturan pendapat pakar kurang dijelaskan; dan (iii) tidak menghitung ketidakpastian terhadap pendugaan. Pada tulisan ini kami menjelaskan pembuatan peta baru global endemisitas malaria untuk mengatasi kelemahan-kelemahan tersebut di atas.

Lingkup Geografis dalam Pemodelan

Batas-batas spasial global transmisi malaria *P. falsiparum* telah dipetakan baru-baru ini dengan menggabungkan data pelaporan insidens kasus nasional, intelijen medis lainnya, dan aturan biologis untuk menentukan dimana transmisi itu tidak mungkin ada, diturunkan dari batasan suhu dan tingkat kekeringan terhadap bionomik vektor dominan lokal *Anopheles* [17,18]. Hasil kegiatan ini kemudian membagi dunia ke dalam tiga kelas transmisi *P.falsiparum* untuk tahun 2007: wilayah tidak ada beresiko, resiko tidak stabil (Insidens Parasit Tahunan *P. falsiparum* (*Pf*API) <0,1 per 1.000 orang per tahun (pa)) dan resiko stabil (*Pf*API ≥0,1 per 1.000 orang pa). Kelas-kelas ini disajikan dalam Gambar 1. Klasifikasi stabil–tidak stabil *Pf*API didasarkan kepada kajian secara statistika, logistik, program dan alasan pragmatis untuk mendukung tingkat *Pf*API yang digunakan dalam menentukan tindakan-tindakan yang diambil selama kampanye global eradikasi malaria [19-21].

Pemetaan yang dijelaskan disini sesungguhnya memperluas pekerjaan ini. Survey-survey malariometrik secara global yang terbesar yang pernah dikumpulkan digunakan untuk menduga nilai prevalensi malaria *P. falsiparum* pada semua titik yang berada di dalam batas spasial transmisi stabil sehingga diperoleh permukaan kontinu endemisitas *P. falsiparum*. Untuk memfasilitasi proses ini batasan spasial memerlukan penarikan sampel ulang sampai grid 5×5 km dengan menggunakan ArcView GIS 3.2 (ESRI, 1999) sebab teknik pemetaan komputasi yang secara intensif dipergunakan ini, dan akan dijelaskan nanti, tidak dapat dilakukan untuk resolusi spasial 1×1 km pada skala global.

Pendekatan Baru Untuk Pemetaan Endemisitas Malaria

Banyak pendekatan yang tersedia untuk menghasilkan peta endemisitas kontinu dengan menggunakan data dari survei-survei malariometrik, dimana kesemuanya memerlukan model untuk memprediksi nilai endemisitas pada wilayah-wilayah dimana data survei tak tersedia [22-26]. Peta-peta yang dihasilkan dari model seperti ini memiliki suatu ketidakpastian di dalam pendugaannya dan perhitungan tentang ketidakpastian ini sendiri menjadi perhatian utama di dalam pemetaan penyakit.

Sejumlah studi terbaru telah mengadopsi kerangka kerja dengan kemampuan memprediksi dikenal sebagai model berbasis geostatistika (MBG) [27] untuk menduga pendugaan spasial endemisitas malaria [28-33] dan prevalensi dari penyakit bersumber binatang dan penyakit dengan penjamu sementara lainnya [34-38]. MBG memberikan penafsiran statistik formal terhadap perangkat geostatistika klasik untuk menarik suatu kesimpulan secara statistik [39-41] dan memungkinkan penggunaan metode Bayesian dalam pengambilan kesimpulan tersebut [42,43]. Keuntungan utama MBG dalam pemetaan penyakit adalah ditanganinya secara tepat ketidakpastian yang terjadi pada setiap tahapan di dalam proses pemodelan [27]. Dengan memodelkan interaksi dari sumber-sumber ketidakpastian ini, maka suatu sebaran peluang dapat dihasilkan dari masing-masing lokasi pendugaan, yang kemudian dapat diringkaskan ke dalam suatu ukuran kepercayaan di sekitar nilai dugaan. Oleh sebab itu, peta yang dihasilkan memiliki bukti acuan yang terbaru dalam menentukan endemisitas global malaria, menggunakan teknik MBG untuk menilai tingkat kepercayaan dalam pendugaan, dan menyediakan kepada pemakai peta ini suatu dugaan yang jelas tentang ketepatan dari pendugaan-pendugaan tersebut [36].

Prinsip dasar geostatistika adalah bahwa prediksi yang dipetakan tersebut menjadi semakin tidak pasti ketika kepadatan dan kedekatan titik-titik data itu semakin berkurang. Ketika data dikumpulkan dalam waktu yang berbeda-beda, seperti halnya lokasi yang berbeda-berbeda, maka prinsip ini juga berlaku untuk lintas waktu dan lintas ruang. Contoh-contoh dari studi-studi epidemiologis yang mengembangkan teknik spasial geostatistika yang menyertakan lintas waktu memang masih jarang digunakan [44-47], tetapi di dalam penelitian ini kerangka kerja pemodelan spasial-temporal telah dikembangkan secara utuh. Dengan melibatkan dimensi waktu maka dimungkinkan untuk membandingkan patok duga ini dengan iterasi peta-peta di masa depan. Nantinya peta ini menyediakan kerangka kerja geografis yang jelas untuk memantau dan mengevaluasi dampak komunitas pengendalian malaria terhadap malaria *P.falsiparum* di seluruh dunia.

Metode

Garis Besar Analisis

Tujuan analisis ini adalah menggunakan basisdata survey parasit *Plasmodium falsiparum* (*Pf*PR) yang terbaru untuk membuat permukaan kontinu endemisitas global malaria *P. falsiparum* tahun 2007, dibuat dengan metode yang transparan dan bisa diproduksi ulang dan ketidakpastian pendugaannya didokumentasikan dengan baik.

Langkah-langkah utama untuk membuat peta global kontinu prevalensi *P. falsiparum* sesuai dengan kerangka analitis kami disajikan di dalam Gambar 2. Pertama, diperlukan pencarian dan pengolahan pendahuluan terhadap data *Pf*PR untuk menghasilkan basisdata yang memiliki geo-lokasi dari survey-survey malariometrik dan memeriksa peubah-perubah lingkungan yang potensial (Protokol S1) dan pengaruh dari pola pemukiman penduduk [48-50] (Protokol S2). Kedua, basis data *Pf*PR yang telah dibersihkan ini kemudian digunakan dalam MBG dengan teknik statistika Bayesian untuk membuat suatu permukaan kontinu prevalensi malaria dengan memperhatikan standarisasi-umur dan koreksi-urban (Protokol S3). Ketiga, prosedur validasi secara luas digunakan untuk menilai akurasi pendugaan endemisitas dan ukuran ketidakpastian (Protokol S4). Terakhir, dugaan jumlah penduduk beresiko (PAR) terhadap malaria *P. falsiparum* dihitung secara global dan ditampilkan untuk skala kawasan, dikelompokkan berdasarkan kelas umur.

Pengumpulan Basisdata Global Prevalensi Parasit *P. falsiparum*

Dari semua ukuran potensial yang tersedia untuk mengukur endemisitas malaria, tingkat parasit (proporsi sampel yang bisa dideteksi keberadaan parasit di dalam darah perifer) lebih diminati sebagai dasar pemetaan, karena secara global dipergunakan dimana-mana [18] dan memiliki sensitifitas terhadap berbagai variasi transmisi malaria *P. falsiparum* [19]. Pengelompokan jangkauan endemisitas malaria yang informatif secara epidemiologis seperti yang telah disarankan adalah kelompok umur 2 (2.00) sampai 10 (9.99) tahun [51], dipandu oleh adanya dampak potensial terhadap endemisitas malaria dengan menggunakan intervensi malaria yang secara luas dipergunakan saat ini - kelambu celup berinsektisida (ITN) [19]. Kelas terendah *Pf*PR pada kelompok umur 2 sampai 10 tahun (dinamakan *Pf*PR_2-10_), berada pada nilai ≤5%. Dibawah nilai inilah survey-survey *Pf*PR mengambil sampel populasi menjadi terkendala secara logistik untuk mengukur endemisitas secara akurat dan oleh sebab itu malariometrik berbasis surveilans lebih disukai [52-54]. Kami mempertimbangkan transmisi stabil sedang dapat diwakilkan oleh *Pf*PR_2-10_ >5% sampai <40%, semenjak berbagai model matematis menduga bahwa transmisi malaria dapat diputus dengan cakupan universal ITN untuk semua wilayah dengan *Pf*PR_2-10_ <40% [19,55]. Meskipun adanya ketidakpastian karena perilaku dan bionomik vektor dominan lokal *Anopheles* [56], *Pf*PR_2-10_ <40% dipertimbangkan sebagai patokan konservatif, semenjak ITN jarang digunakan sendiri tanpa intervensi lain sehingga nantinya transmisi berkurang lebih besar lagi. Wilayah dengan transmisi stabil tinggi, dimana intervensi campuran perlu dipertimbangkan jika pemutusan transmisi mungkin dilakukan, ditentukan apabila prevalensi malaria berada di atas nilai: *Pf*PR_2-10_ ≥40%. Klasifikasi malaria ini digunakan sebagai acuan dalam menginterpretasikan dugaan endemisitas dan merupakan pendekatan berbeda dibandingkan acuan endemisitas tradisional [57] yang telah diperlihatkan dalam kebanyakan model tidak memberikan dorongan yang berarti terhadap kesempatan untuk mengendalikan dan mengeliminasi malaria [19,55].

Proses identifikasi, pengumpulan dan geo-lokasi survey prevalensi malaria berbasis komunitas dilakukan sejak tahun 1985 dan telah dijelaskan [58]. Pencarian data *Pf*PR merupakan kegiatan Malaria Atlas Project yang sedang berjalan (MAP, http://www.map.ox.ac.uk) dan diselesaikan tanggal 31 Juli 2008 untuk iterasi peta global endemisitas malaria tahun 2007 (Protokol S1.1). Terdapat total 8,938 survey penampang-melintang dugaan *Pf*PR yang bersumber dari 78 diantara 87 negara endemis *P. falsiparum* (*Pf*MECs) [18]. Negara-negara yang tidak ada di dalam basisdata adalah Bangladesh, Belize, Bhutan, Djibouti, Republik Dominika, Guyana, Iran, Kyrgyzstan dan Panama.

Setelah enam tahap proses pemisahan (mengeluarkan survey-survey yang hanya berada pada poligon besar (>100 km^2^) dan kecil (>25 km^2^) [58]; mengeluarkan survey-survey yang tidak bisa digeo-lokasi, atau tidak bisa digeo-lokasi secara tepat; dan mengeluarkan survey-survey yang lokasinya tidak bisa dipisahkan atau survey-survey yang tanggalnya tidak diketahui), maka diperoleh 7,991 survey *Pf*PR (Gambar S1.2 dalam Protokol S1)

Sebelum dipetakan semua data *Pf*PR umurnya distandarisasi menjadi kelompok umur 2 sampai 10 tahun dengan menggunakan algoritma berbasis model konversi katalitis yang pertama kali dipergunakan oleh Pull and Grab [59]. Algoritma ini tampil baik dari berbagai pilihan prosedur standarisasi lainya dan telah dijelaskan di tempat yang lain (Protokol S1.3) [51].

Basisdata terakhir kemudian dikelompokkan ke dalam tiga kawasan utama (Gambar 1): Amerika; Afrika, Yaman dan Saudi Arabia (Afrika+); and Asia Tengah dan Tenggara (CSE Asia) (Protokol S1.4). Pembagian seperti ini memandang kawasan-kawasan tersebut berbeda secara biogeografis, entomologis dan epidemiologis [8,16], sejalan dengan upaya mempertahankan sejumlah data yang cukup memadai supaya analisisnya berarti. Pembagian secara global ini didukung oleh berbedanya struktur spasial *Pf*PR_2-10_ pada setiap kawasan, yang digambarkan oleh semi-variograms (Gambar S1.1 dalam Protokol S1).

Pendekatan-pendekatan khusus transmisi untuk memetakan batasan urban, peri-urban dan rural juga dikembangkan, dan latar belakang pengembangan ini dijelaskan secara detail pada bagian yang lain (Protokol S2) [50]. Singkatnya, semua batasan urban (UE) ditentukan dengan Global Rural Urban Mapping Project (GRUMP) alpha version UE mask (GRUMP UE) [60,61] pada resolusi spasial 1x1 km (Protokol S2.1) [50]. Untuk urban, wilayah-wilayah dengan kepadatan populasi diatas 1000 orang setiap km^2^ menurut densitas populasi dari Gridded Population of the World version 3 [60,61] kemudian dipetakan [48]. Semua survey kemudian ditetapkan baik sebagai urban (Gridded Population of the World version 3 ≥1.000 km^2^ dalam GRUMP UE), peri-urban (Gridded Population of the World version 3 <1.000 km^2^ dalam GRUMP UE), atau rural (diluar GRUMP UE) (Protocol S2.2).

Pencilan ekstrim pada data rural *Pf*PR_2-10_ kemudian diidentifikasi menggunakan suatu saringan geostatistis (Protokol S1.5). Proses ini menggunakan statistik semi-variogram untuk menilai apakah setiap titik berbeda secara nyata dari titik-titik yang berdekatan dengan sebelumnya diketahui selisih jarak dan pola regional dari variasi spasial. Prosedur ini mengidentifikasi 38 baris data non-urban *Pf*PR_2-10_, yang kemudian dikeluarkan dari basis data sebelum pemodelan berikutnya. Perincian dari survey tersebut dapat disediakan berdasarkan permintaan.

Koleksi data final *Pf*PR_2-10_ (n=7.953) yang digunakan ditampilkan di Gambar 1. Atribut basis data final *Pf*PR_2-10_ juga dijelaskan (Tabel S1.2 dalam protokol S1), bersama dengan plot median *Pf*PR_2-10_ berdasarkan periode tahun pengamatan (Gambar S1.3 dalam Protokol S1), mengindikasikan bahwa waktu merupakan sumber keragaman penting dalam model MBG. Eksplorasi pendahuluan yang serupa terhadap hubungan data tersebut dengan berbagai variasi iklim [62] dan peubah lingkungan dengan citra jarak jauh [63] menunjukkan bahwa tidak terdapat hubungan yang kuat (Gambar S1.5 and S1.6 dalam Protokol S1), sehingga analisis lebih banyak dilakukan dengan pendekatan peubah tunggal.

Ada pandangan umum yang salah bahwa survey malariometrik hanya dilakukan di wilayah-wilayah dengan prevalensi tinggi. Kenyataannya, terdapat peningkatan kecenderungan melakukan survey nasional untuk memperkuat keterwakilan semua wilayah di dalam suatu negara, dan konfirmasi ketika tidak ditemukannya transmisi *P. falsiparum* ketika sampel diambil untuk mendapatkan *P. vivax,* menghasilkan banyak nilai prevalensi nol pada survey-survey tersebut. Secara keseluruhan, 119 dari 261 survey melaporkan nilai nol di Amerika, 1010 dari 5307 survey melaporkan nilai nol di Afrika+ dan 775 dari 2385 survey melaporkan nilai nol di wilayah CSE Asia (Gambar 1).

Detail Statistika

Algoritma geostatistika menghasilkan peta-peta kontinu melalui pendugaan nilai lokasi-lokasi yang tidak disampel menggunakan kombinasi linier dari data sampel yang ada. Dalam upaya pemetaan seperti yang dijelaskan di dalam penelitian ini, secara intuisi bahwa kepercayaan dalam menduga *Pf*PR_2-10_ di lokasi-lokasi yang tidak disampel akan dipengaruhi oleh (i) sebaran titik-titik survey diseputar lokasi tersebut (kepadatan spasial dalam data latihan), (ii) kondisi dimana *Pf*PR_2-10_ bervariasi secara mulus sepanjang ruang (heterogenitas dalam data latihan), dan (iii) jumlah orang yang disampel dari masing-masing survey (ketelitian komponen survey dalam data latihan). Pendekatan MBG [27] digunakan dalam kerangka kerja Bayesian yang menggabungkan faktor-faktor ini untuk menghasilkan peta kontinu *Pf*PR_2-10_ (Protokol S3). Karena data dikumpulkan pada waktu yang berbeda sepanjang periode penelitian 1985-2008, maka merupakan suatu hal yang penting untuk mengembangkan pendekatan geostatistika ruang semata menjadi pendekatan ruang-wilayah yang menghitung secara bersama-sama kepadatan dan heterogenitas data ruang dan waktu tersebut. Algoritma standarisasi-umur dimasukkan sebagai bagian dari model sehingga kesalahan yang ada di dalam proses ini dapat diduga dan dimasukkan ke dalam tahapan MBG (Protokol S3).

Untuk setiap kawasan, suatu model geostatistika Bayesian untuk *Pf*PR_2-10_ tahun 2007, , dibuat untuk setiap lokasi yang dimodelkan sebagai transformasi dari wilayah terstruktur spasiotemporal ditambah dengan variasi tidak terstruktur (acak) . Jumlah respon positif *P. falsiparum* dari total sampel pada setiap lokasi survey dimodelkan sebagai variabel binomial saling bebas bersyarat dengan diketahui sebelumnya nilai *Pf*PR_2-10_ standarisasi-umur yang tidak diamati [36]. Komponen spatiotemporal ditampilkan melalui proses Gausian stasioner dengan nilai tengah dan kovarians yang diperoleh melalui versi anisotropis spasial dari fungsi kovarians ruang-waktu yang diusulkan oleh Stein [64]. Modifikasi fungsi kovarians Stein dibuat untuk memungkinkan model marginal-waktu memasukkan komponen periodik dengan panjang gelombang 12 bulan, sehingga memberikan kemampuan untuk memodelkan efek musiman di dalam struktur kovarians temporal teramati. Efek-efek tersebut muncul ketika penelitian dilakukan dalam tahun yang berbeda tapi masih dalam bulan kalender yang sama sehingga memiliki kecenderungan untuk lebih mirip antara satu dengan yang lainnya dibandingkan dengan yang diperkirakan ketika tidak adanya pola musiman. Komponen nilai tengah dimodelkan sebagai fungsi linier dari waktu, , dan prediksi lokasi apakah urban, atau peri-urban (ditunjukkan oleh variabel indikator dan ) atau rural: . Setiap survey dirujuk secara temporal menggunakan titik tengah (desimal untuk tahun) antara bulan mulai survey dan bulan berakhirnya survey. Status urban, peri-urban, atau rural ditetapkan untuk setiap lokasi pendugaan menggunakan permukaan wilayah urban GRUMP modifikasi yang telah dijelaskan sebelumnya (Protokol S2.2), dilakukan pengambilan sampel ulang sampai grid 5×5 km. Komponen tidak terstruktur dijelaskan sebagai Gaussian dengan nilai tengah nol dan ragam . Penarikan kesimpulan Bayesian menggunakan Markov Chain Monte Carlo (MCMC) untuk memperoleh sampel-sampel dari sebaran posterior: wilayah Gaussian untuk setiap lokasi data; parameter tidak teramati , *,* , dan serta parameter-parameter tidak teramati lainnya yang digunakan untuk mendefinisikan struktur dan anisotropi dari fungsi kovarians ruang-waktu eksponensial (Protokol S3.4). Jarak antar lokasi dihitung dalam jarak lingkaran-besar untuk menampung efek lengkungan bumi, menjadi suatu hal penting dalam skala regional. Sampel-sampel kemudian dibangkitkan dari nilai tengah tahun 2007 dari sebaran posterior pada setiap lokasi pendugaan. Untuk masing-masing sampel dari posterior gabungan, pendugaan dibuat menggunakan simulasi bersyarat ruang-waktu selama 12 bulan untuk tahun 2007 [44,65]. Pendugaan ini dibuat pada titik-titik di atas grid spasial 5×5 km dalam batas spasial transmisi stabil *P. falsiparum*. Oleh sebab itu keluaran model terdiri atas sampel-sampel dari sebaran posterior dugaan untuk nilai tengah *Pf*PR_2-10_ tahun 2007 pada masing-masing lokasi grid, yang mana digunakan untuk membangkitkan titik-titik pendugaan (dihitung sebagai nilai tengah dari masing-masing bagian sampel posterior), peluang keanggotaan kelas endemisitas dan dugaan ragam standar (Protokol S3.4). Penjelasan yang mendalam tentang bagaimana keluaran geostatistika digunakan untuk membuat berbagai peta tersebut juga diberikan (Protokol S3.5).

Validasi Model

Penilaian kelayakan permukaan untuk dipetakan merupakan hal yang penting dan beberapa metode deskriptif yang penting telah digunakan (Protokol S4). Kemampuan model untuk menduga nilai titik *Pf*PR_2-10_ dan kemungkinan kelas endemisitas diuji menggunakan prosedur *hold-out*. Data untuk validasi dipilih sebanyak sepuluh persen (n=800) menggunakan penarikan sampel acak yang tidak tergerombol secara spasial, kemudian data ini dikeluarkan dari basisdata (Protokol S4.1). Model kemudian dijalankan menggunakan 7.153 titik data yang tersisa untuk menghasilkan sebaran posterior dugaan *Pf*PR_2-10_ yang nantinya digunakan untuk diperbandingkan dengan nilai-nilai dari 800 lokasi yang dikeluarkan tadi. Untuk dibandingkan dengan model utama tadi, yang nilai tengah tahunannya diprediksi untuk tahun 2007, proses validasi menduga nilai *Pf*PR_2-10_ untuk bulan yang sepadan dengan titik-tengah dari masing-masing survey yang dikeluarkan tadi, sehingga diperoleh nilai serupa waktunya. Dengan besarnya basis data, satu kali validasi dipandang memadai untuk mendapatkan statistik validasi dengan tingkat ketelitian yang diperlukan.

Kemampuan untuk memprediksi nilai *Pf*PR_2-10_ yang telah diketahui sebelumnya diringkaskan menggunakan nilai tengah galat sebagai suatu ukuran bias secara keseluruhan, nilai tengah galat sebagai ukuran akurasi secara keseluruhan, koefisien korelasi sebagai ukuran hubungan linier [44,66]. Statistik ini disajikan baik sebagai nilai mutlak dan sebagai proporsi nilai tengah *Pf*PR_2-10_ pada setiap wilayah seperti yang dihitung dari data validasi. Kemampuan menduga keanggotaan kelas endemisitas diuji menggunakan statistik *area-under-curve* (AUC) yang diperoleh dari kurva *receiver-operating-characteristic* (ROC), dengan plot sensitifitas terhadap 1-spesifisitas untuk masing-masing kelas endemisitas [34,67]. Nilai AUC di atas 0,9 mengindikasikan kesepakatan yang sangat baik antara kelas sesungguhnya dengan kelas dugaan, nilai di atas 0,7 mengindikasikan kesepakatan yang cukup baik, dan nilai 0,5 mengindikasikan bahwa model ini tidak lebih baik dari ditentukan kelas keanggotaan secara acak saja [34,67]. Sebuah prosedur juga digunakan [44,68] untuk menguji kondisi sebaran posterior dugaan pada setiap lokasi pendugaan apakah mampu menghasilkan ukuran ketidakpastian yang sesuai. Prosedur ini memungkinkan peluang terhadap nilai dugaan *Pf*PR_2-10_ untuk setiap lokasi pendugaan bisa diperbandingkan dengan peluang amatan yang sepadan di dalam setiap kawasan. Penjelasan lebih lanjut dari prosedur ini juga disediakan (Protokol S4.2).

Distribusi frekuensi *Pf*PR_2-10_ divisualisasikan juga untuk data masukan dan data keluaran dari permukaan pendugaan menggunakan plot violin [69]. Plot-plot ini menampilkan pendekatan yang dipermulus untuk distribusi frekuensi (plot densitas kernel) *Pf*PR_2-10_ untuk setiap kawasan dengan dilengkapi dengan batang yang memperlihatkan nilai median dan nilai antar-kuartil. Plot terpisah juga dihitung menggunakan *Pf*PR_2-10_ dari data standarisasi-umur untuk semua tahun yang ada di dalam basisdata dan untuk data tahun 2007 saja, dan sebuah plot lainnya dihitung menggunakan pendugaan titik untuk setiap lokasi dari permukaan pendugaan *Pf*PR_2-10_ tahun 2007.

Menduga Kepadatan Populasi Penduduk Tahun 2007

Versi alpha GRUMP menyediakan jumlah populasi dan kepadatan populasi pada resolusi spasial 1×1 km untuk tahun 1990, 1995 dan 2000, baik yang telah mengalami penyesuaian dan yang belum terhadap dugaan populasi nasional PBB (Protokol S2.3) [60,61]. Jumlah penduduk yang telah mengalami penyesuaian untuk tahun 2000 kemudian diproyeksikan untuk tahun 2007 dengan menggunakan laju pertumbuhan antar sensus nasional, variasi medium [70] menggunakan metode-metode yang telah dijelaskan sebelumnya [71] (Protokol S2.4). Jumlah populasi ini kemudian dikelompokkan berdasarkan kelompok umur sesuai dengan definisi PBB [72] tentang struktur umur populasi pada tahun 2005 untuk mendapatkan jumlah penduduk pada kelompok umur dibawah 5 tahun, 5-14 tahun dan ≥ 15 tahun.

Batas-batas digital dari 87 negara-negara endemis malaria *P. falsiparum* ditumpuk di atas permukaan kelas endemisitas yang telah mengalami penyesuaian terhadap urban (diproyeksikan kembali pada proyeksi wilayah yang sama) dan wilayah-wilayah kelas endemisitas menggunakan ArcView GIS 3.2 (ESRI, 1999) (Protokol S2.4). Lapisan-lapisan tersebut kemudian ditumpuk lagi di atas data GRUMP data [60,61] untuk memperoleh dugaan jumlah penduduk yang memiliki resiko terhadap *P. falsiparum* berdasarkan endemisitas dan kelompok umur (Protokol S2.4). Akhirnya permukaan-permukaan ini kemudian digabungkan dengan peta ketidakpastian untuk mendapatkan indek ketidakpastian populasi-terboboti (perkalian dari log kepadatan penduduk dengan kebalikan dari peluang penentuan kelas yang benar).

Hasil

Akurasi Pendugaan Peta Endemisitas Malaria *P. falsiparum*

Permukaan pendugaan kontinu untuk endemisitas malaria *P. falsiparum* ditampilkan pada Gambar 3. Pengendalian yang berhubungan dengan penentuan yang paling memungkinkan dari keanggotaan kelas endemisitas juga ditampilkan pada Gambar 4. Peluang aktual dari pendugaan setiap kelas secara benar disajikan pada Gambar 5A. Penjelasan rinci tentang keragaman regional pada berbagai tingkatan resiko stabil dan PAR yang berhubungan, setelah penjelasan akurasi pendugaan di dalam teks. Ukuran-ukuran alternatif untuk ketidakpastian pendugaan juga disajikan (Protokol S4.3).

Pendugaan Nilai-Titik *Pf*PR_2-10_

Pemeriksaan nilai tengah galat dalam permukaan endemisitas malaria *P. falsiparum* (Gambar 3) menghasilkan bias minimal terhadap nilai dugaan *Pf*PR_2-10_ keseluruhan yaitu 0,91 yang artinya ada kecendrungan untuk menduga-lebih *Pf*PR_2-10_ kurang dari satu persen (Amerika = 0,63, Afrika+ = 0,80, CSE Asia = 1,18) (Tabel 1). Pemeriksaan nilai tengah galat mutlak menghasilkan rata-rata kesalahan dalam pendugaan *Pf*PR_2-10_ sebesar 9,75 (Amerika = 3,52, Afrika+ = 11,02, CSE Asia = 7,71) (Tabel 1). Koefisien korelasi global antara nilai aktual dengan nilai dugaan sebesar 0,82, yang mengindikasikan adanya kesepakatan linier yang sangat kuat untuk skala global and hal ini kemudian diilustrasikan lebih lanjut di dalam plot pencar (Gambar 6A dan Tabel 1). Korelasi pada kawasan regional untuk Amerika dan CSE Asia pada umumnya lebih lemah (Amerika = 0,03, Afrika+ = 0,82, CSE Asia = 0,70) (Tabel 1). Suatu semi-variogram dari sisaan model yang dibakukan (Gambar 6B) memperlihatkan beberapa bukti autokorelasi spasial yang sangat lemah, sampai ke selisih dua derajat desimal, meskipun perbandingan dengan simulasi amplop-kosong menunjukkan bahwa hasilnya tidak nyata secara statistik (Protokol S4.2).

Menduga Kelas Endemisitas

Kurva *receiver-operating-characteristics* dan statistik AUC untuk masing-masing kelas endemisitas juga ditampilkan (Gambar 6C dan Tabel 2). Nilai AUC secara global untuk ketiga kelas endemisitas melampui nilai batas 0,7 yang artinya kemampuan untuk membedakan berkisar antara sedang sampai ke baik. Sedangkan untuk kelas *Pf*PR_2-10_ ≤5% dan *Pf*PR_2-10_ ≥40% nilai AUC secara global melampui nilai ambang batas 0,9 yang artinya kemampuan untuk membedakan sangat bagus. Secara keseluruhan, 70,8% dari titik-titik tersebut diklasifikasikan dengan benar (Amerika = 80,0%, Afrika+ = 70,6%, CSE Asia = 69,9%) dan yang penting, hanya 1,1% dari titik-titik tersebut salah klasifikasi ke dalam kelas yang tidak berbatasan (Amerika = 0,0%, Afrika+ = 0,6%, CSE Asia = 2,5%) (Tabel 2). Tabel kontingensi untuk masing-masing kelas juga ditampilkan (Protokol S4.3).

Ukuran Ketidakpastian yang Realistis untuk Setiap Pendugaan

Plot Peluang-Peluang membandingkan ambang batas peluang pendugaan dengan peluang yang diamati (Gambar 6D) memperlihatkan bahwa secara umum terdapat hubungan dekat antara kedua pengukuran tersebut, yang artinya bahwa model menyediakan gambaran yang meyakinkan tentang ketidakpastian pendugaan titik. Namun, garis plot jatuh sedikit di atas garis 1:1 melintasi sebagian besar nilai ambang batas, paling banyak pada ambang batas peluang dugaan antara 0,00 dan sekitar 0,25. Hal ini berarti ambang batas peluang dugaan, misalnya 0,1, berkemungkinan berhubungan dengan “nilai ambang peluang aktual” disekitar nilai 0,2. Dengan kata lain, model ini memiliki tendensi untuk menduga lebih rendah besaran peluang *Pf*PR_2-10_ untuk mendapatkan nilai-nilai yang kecil (Gambar S4.1A dalam Protokol S4). Kecenderungan ini mungkin menyebabkan, untuk berikutnya, menduga lebih tinggi nilai *Pf*PR_2-10_ pada beberapa daerah dengan endemisitas rendah.

Peta Global Endemisitas malaria *P. falsiparum*

Untuk tahun 2007 luasan wilayah dengan resiko stabil malaria *P. falsiparum* sebesar 29,73 juta km^2^, tersebar di wilayah Amerika (6,03 juta km^2^, 20,30%), Afrika+ (18,17 juta km^2^, 61,10%), dan CSE Asia (5,53 juta km^2^, 18,60%) (Tabel 3). Kami menduga sebelumnya bahwa terdapat 2,37 milyar orang yang beresiko terhadap transmisi malaria *P. falsiparum* di seluruh dunia dan sekitar 0,98 milyar diantaranya hidup di wilayah dengan resiko tidak stabil [17,18]. Untuk mereka yang terpapar terhadap resiko stabil, 1,383 milyar, tersebar di Amerika (0,041 milyar, 2,94%), Afrika+ (0,657 milyar, 47,48%) dan CSE Asia (0,686 milyar, 49,58%) (Tabel 4 dan Gambar 7). Variasi di wilayah dengan resiko stabil *P. falsiparum*, dikelompokkan ke dalam kelas-kelas endemisitas yaitu rendah (*Pf*PR_2-10_ ≤5%), sedang (*Pf*PR_2-10_ >5-<40%) dan tinggi (*Pf*PR_2-10_ ≥40%) seperti yang dijelaskan di bawah ini. Di Amerika and CSE Asia, anak-anak (kelompok umur 0-4 dan 5-14) mendekati sepertiga (masing-masingnya 32%) dari total PAR. Di Afrika proporsi ini meningkat sampai 43%.

Amerika

Kawasan transmisi stabil *P. falsiparum* di Amerika memiliki sifat endemisitas rendah yang seragam (*Pf*PR_2-10_ ≤5%) (Gambar 3 dan 4). Total wilayah resiko stabil meliputi 6,3 juta km^2^, sebagian besar berada di lembah sungai Amazon (Gambar 3 dan 4). Seluruh 40,64 juta penduduk di wilayah ini terpapar dengan resiko rendah. Median prevalensi sebesar 2,17% dengan nilai dugaan *Pf*PR_2-10_ terendah dan tertinggi sebesar 0,31% dan 8,81%, masing-masingnya (Gambar 8C). Pemeriksaan distribusi frekuensi untuk kawasan ini memperlihatkan nilai dugaannya tersebar secara simetris terhadap nilai mediannya (Gambar 8C). Data yang dimasukkan untuk tahun 2007 (Gambar 8B) memperlihatkan kisaran nilai yang serupa tapi memiliki kecondongan positif, sedangkan data untuk seluruh tahun meliputi kisaran yang lebih lebar (maksimum = 21,30%) dan terlihat jelas adanya kecondongan positif (Gambar 8A). Peluang untuk menentukan kelas endemisitas secara benar besarannya relatif tinggi di Amerika (Gambar S4.1A dalam Protokol S4), utamanya disebabkan oleh nilai *Pf*PR_2-10_ rendah yang relatif tersebar seragam [17,18], daripada adanya struktur spasial yang kuat lainnya (Gambar S1.1 dalam Protokol S1). Hasil ini, digabungkan dengan kepadatan populasi yang relatif rendah di kawasan tersebut, mengarah kepada nilai-nilai terendah dari indek ketidakpastian populasi-terboboti (Gambar 5B).

Afrika+

Kawasan transmisi stabil *P. falsiparum* di Africa+ meliputi 18,17 juta km^2^, yang meliputi 656,61 juta orang yang beresiko dan tersebar pada kisaran intensitas transmisi yang lebar. Lebih dari 4,03 juta km^2^ (22,18%) luasan di kawasan ini dan 114,50 juta jiwa (17,44%) tinggal pada kelas *Pf*PR_2-10_ ≤5%. Daerah-daerah ini berlokasi di tengah dan sebelah timur dari lintang selatan dan lintang utara (Gambar 3 dan 4). Kelas endemisitas ini secara relatif bisa diduga dengan baik (Gambar S4.1A dalam Protokol S4) Wilayah-wilayah transmisi tinggi dimana *Pf*PR_2-10_ ≥40% mendominasi wilayah Afrika Barat dan sebagian besar Afrika Tengah, meliputi 8,50 juta km^2^, dimana 345,28 juta penduduk beresiko terhadap malaria. Peluang untuk menduga kelas endemisitas secara benar nilainya tinggi di Afrika Barat dan lebih rendah di Afrika Tengah (Gambar S4.1C dalam Protokol S4), disebabkan oleh melimpahnya data survey *Pf*PR_2-10_ yang terbaru di wilayah yang disebutkan pertama dan kurangnya banyaknya data di wilayah yang disebutkan kemudian (Gambar 1). Bagian penting dari benua ini (5,63 juta km^2^) memiliki kelas endemisitas sedang, *Pf*PR_2-10_ >5% sampai <40%, dan meliputi 196,83 juta PAR. Kelas endemisitas ini diduga dengan tingkat keyakinan yang paling rendah (Gambar S4.1B dalam Protokol S4.3).

Prevalensi median dugaan untuk wilayah endemisitas stabil di benua ini sebesar 33,34%, dengan nilai dugaan *Pf*PR_2-10_ terendah dan tertinggi sebesar 0,20% dan 75,40%, masing-masingnya (Gambar 8C). Distribusi frekuensi dari nilai dugaan (Gambar 8) terpusat di nilai median, dengan adanya modus kedua di sekitar 15% (Gambar 8C). Sebaran ini sangat berbeda dibandingkan dengan data seluruh tahun dan data masukan tahun 2007, yang keduanya memiliki kecondongan positif dengan nilai maksimum 99,78% dan 98,70%, masing-masingnya (Gambar 8A dan 8B). Indek ketidakpastian populasi-terboboti memperlihatkan gambaran campuran untuk kawasan ini, dimana nilai tinggi di Ethiopia untuk kelas endemisitas rendah dan nilai tinggi di Nigeria untuk kelas endemisitas tinggi (Gambar 5B), mencerminkan adanya densitas rendah survey-survey *Pf*PR_2-10_ dan jumlah penduduk yang besar di masing-masing negara.

CSE Asia

Wilayah stabil transmisi *P. falsiparum* di kawasan CSE Asia memiliki sifat endemisitas malaria rendah (*Pf*PR_2-10_ ≤5%), endemisitas sedang walaupun secara geografis kecil namun penting secara epidemiologis (*Pf*PR_2-10_ >5 sampai <40%) dan resiko tinggi (*Pf*PR_2-10_ ≥40%) seperti contoh, Orissa state, India bagian timur, Myanmar bagian barat dan daratan rendah New Guinea. Luas wilayah dengan resiko stabil ini meliputi 5,53 juta km^2^, dengan PAR 685,65 juta, sebagian besar berada di India dan Indonesia (Gambar 3 dan 4). Lebih dari 4.72 juta km^2^ (85.54%) dari wilayah ini dan 603,61 juta (88,03%) penduduk tinggal pada *Pf*PR_2-10_ ≤5%. Median prevalensi dugaan sebesar 9,99%, with dimana nilai dugaan *Pf*PR_2-10_ terendah dan tertinggi sebesar 0,006% dan 45,40% masing-masingnya. Sebaran frekuensi nilai dugaan *Pf*PR_2-10_ memiliki kecondongan positif (Gambar 8C). Sebaran frekuensi data input 2007 berada pada kisaran nilai yang serupa, tapi terlihat jelas lebih condong positif (Gambar 8B). Plot data untuk seluruh tahun juga condong positif tapi mencakup kisaran nilai yang jauh lebih besar, maksimum 93.91% (Gambar 8A). Peluang untuk menentukan kelas endemisitas secara benar nilainya relatif tinggi di kawasan CSE Asia, tapi terdapat ketidakpastian yang cukup besar untuk wilayah perbatasan antara kelas endemisitas rendah dan sedang (Gambar S4.1 dalam Protokol S4). Hasil ini, digabungkan dengan kepadatan populasi yang tinggi di kawasan itu, mengarah kepada indek ketidakpastian populasi-terboboti yang paling tinggi, ditemukan terutama di India (Gambar 5B).

Pembahasan

Sepanjang pengetahuan kami, untuk pertama kalinya dalam kurun waktu 40 tahun kami menyediakan sebuah peta terbaru endemisitas malaria *P. falsiparum* untuk skala global. Peta ini mengatasi kelemahan-kelemahan dari peta-peta distribusi global resiko malaria sebelumnya oleh sebab itu peta ini memiliki kekhususan sebagai berikut. Pertama, peta didasarkan kepada basisdata survey malariometrik yang terdokumentasi dan tersebar luas secara geografis (Protokol S1) [58] yang nantinya ditampilkan untuk domain publik (ketika ijin telah diperoleh untuk masing-masing survey) untuk digunakan dan dievaluasi pada tahun 2009. Kedua, metode MBG (Protokol S3) dan prosedur validasi (Protokol S4) juga telah didokumentasikan secara rinci dan kode yang relevan telah tersedia dalam domain publik. Oleh sebab itu, seluruh proses pemetaan ini seharusnya dapat dibuat ulang oleh mereka yang memiliki akses terhadap sumber daya komputasi yang memenuhi syarat. Ketiga, penilaian yang akurat terhadap ketidakpastian dari hasil pemetaan ini telah dilakukan sehingga keyakinan terhadap hasilnya bisa dievaluasi secara obyektif (Gambar 5).

Situasi Malaria Dunia Tahun 2007

Malaria di dunia berkurang daripada yang diperkirakan dari pemeriksaan peta-peta historis [5,14], baik dengan menyempitnya batas spasial dan menurunnya endemisitas. Ada suatu transisi global yang menjanjikan untuk menuju ekologi resiko malaria yang lebih rendah, yang nantinya akan digali lebih dalam dimasa mendatang.

Dari 1,382 milyar orang yang terpapar resiko malaria secara terus-menerus di seluruh dunia pada tahun 2007, 0,759 milyar diantaranya hidup pada endemisitas malaria yang sangat rendah dengan *Pf*PR_2-10_ ≤5% di CSE Asia (0,604 milyar, 79,55%), Afrika+ (0.115 milyar, 15,09%) dan Amerika (0,041 milyar, 5,36%) (Gambar 7; Tabel 4). Populasi ini hidup pada kondisi dimana harapan biologis untuk mengendalikan transmisi malaria pada tingkatan yang sangat rendah secara terus-menerus dapat dilakukan dan pada akhirnya sangat sesuai dengan kemajuan jangka panjang yang mengarah kepada eliminasi [19]. Namun demikian, rekomendasi khusus untuk sub-kawasan dan nasional seharusnya dihasilkan dari kajian yang serius terhadap faktor-faktor lingkungan, logistik, keuangan dan politik yang mempengaruhi efisiensi rencana pelaksanaan intervensi [73-75]. Sisa populasi global pada wilayah dengan resiko malaria stabil adalah Afrika: 0,197 milyar tinggal di kondisi resiko sedang (*Pf*PR_2-10_ >5 sampai <40%) dan 0,345 milyar di kondisi resiko tinggi (*Pf*PR_2-10_ ≥40%) (Gambar 7; Tabel 4). Di wilayah dengan resiko sedang, pemodelan matematis menyarankan bahwa dengan menggunakan ITN, pemutusan transmisi malaria *P. falsiparum* dapat dilakukan, sedangkan di wilayah transmisi tinggi, transmisi malaria lebih sulit ditangani dan memerlukan pengendalian yang agresif dengan melakukan penggabungan antara intervensi tambahan dan pelengkap lainnya [19,55].

Penggunaan Statistik dan Model Validasi

Prosedur pemodelan yang disajikan di sini menampilkan suatu penggunaan dalam skala besar dari tehnik geostatistika Bayesian modern dan memasukkan sejumlah komponen baru. Dimasukkannya model standarisasi-umur memungkinkan asimilasi yang masuk akal dari data survey dengan kisaran umur yang luas, bersamaan dengan diketahuinya ketidakpastian yang diakibatkan dari berbagai sumber keragaman tambahan ini. Serupa dengan itu, penggunaan lapangan acak spatiotemporal secara utuh memungkinkan survey dari mulai tahun 1985 dapat dimasukkan ke dalam dugaan endemisitas *P. falsiparum* terbaru melalui kerangka kerja statistika dan epidemiologi.

Teknik MBG sangat membutuhkan komputasi yang luar biasa meskipun untuk masalah pendugaan yang kecil. Sepengetahuan kami, ini adalah pertama kalinya prosedur ini digunakan pada skala global. Beban komputasi ini juga menyebabkan sejumlah keterbatasan dalam prosedur pemodelan yang mungkin bisa memperbaiki kemampuan prediksi. Secara khusus, model ini mengadopsi satu nilai tengah dan fungsi kovarian untuk setiap kawasan, dengan menggunakan asumsi stasioneritas turunan-kedua untuk masing-masingnya. Pendekatan lapangan acak non-stasioner yang digunakan dalam skala penelitian yang lebih kecil [32,76] menunjukkan kemungkinan perbaikan bagi model ini, tetapi komputasinya dianggap tidak memungkinkan secara global.

Penilaian berbagai statistik validasi memperlihatkan bahwa kinerja model ini memuaskan untuk setiap dari tiga aspek kinerja: memprediksi nilai *Pf*PR_2-10_ dan kelas endemisitas, dan memberikan ukuran yang realistis terhadap prediksi ketidakpastian. Dengan diketahui bahwa sifat endemisitas *P. falsiparum* sangat bervariasi meskipun dalam waktu yang pendek, korelasi keseluruhan antara prediksi model dan data validasi sebesar 0,82, dan rata-rata galat absolut sebesar 9,75%, *Pf*PR_2-10_ menunjukkan tingkat ketelitian yang tidak terduga. Aspek-aspek tertentu dari ukuran ketidakpastian dari keluaran model adalah sub-optimal: khususnya, kecenderungan untuk menduga sedikit lebih rendah peluang *Pf*PR_2-10_ untuk mendapatkan nilai yang sangat rendah. Meskipun demikian, banyaknya sumber-sumber ketidakpastian yang diperoleh dan tersebar dalam kerangka kerja pemodelan ini, prediksi ketidakpastian yang dihasilkan memperlihatkan sumber informasi yang sangat kaya bagi pembuatan berbagai produk keluaran bagi para pembuat keputusan.

Model ini dipaskan menggunakan MCMC [77,78]. MCMC merupakan algoritma yang sangat kuat, dan hanya untuk tujuan yang umum, algoritma komputasi yang bagus untuk berbagai masalah Bayesian. Namun, algoritma ini merupakan sebuah algoritma pendekatan. Tidak tersedia metode bukti-gagal untuk menduga kesalahan ini, tetapi dengan menggunakan metode heuristik (Protokol S1.3) kami menduga bahwa “kesalahan Monte Carlo” tidak begitu penting terhadap ketidakpastian dalam sebaran posterior aktual kami.

Informasi di dalam peta yang ditampilkan di sini dan ketidakpastiannya bervariasi sepanjang kisaran geografis. Variasi skala-besar dalam endemisitas antara kawasan dan negara terlihat jelas, sangat terkuantifikasi, dan dapat langsung digunakan oleh para perencana global. Namun, sejalan dengan penggunaan skala yang lebih kecil, kegunaan peta ini bagi para pengelola pengendalian malaria di tingkat lokal menjadi menurun meskipun hal ini sangat tergantung kepada ketersediaan dan kepadatan titik-titik survey di lokasi setempat. Ambang batas yang sesuai dan ukuran ketidakpastian menjadi sangat bervariasi bagi para pengguna peta. Namun, sebagai sebuah petunjuk praktis, disarankan bahwa membedakan endemisitas antara daerah yang lebih kecil dengan daerah tingkat administratif pertama mungkin tidak sesuai bagi sebagian besar negara.

Pemeriksaan frekuensi distribusi data *Pf*PR_2-10_ untuk seluruh tahun dan data masukan tahun 2007, dan untuk prediksi permukaan *Pf*PR_2-10_, memperlihatkan sejumlah temuan penting. Pertama, data tahun 2007 dari semua kawasan menunjukkan nilai median dan nilai maksimum yang secara nyata lebih kecil dan lebih condong positif dibandingkan data untuk seluruh tahun (bandingkan Gambar 8A dan 8B). Kedua, terdapat perbedaan yang jelas pada semua kawasan antara distribusi nilai tahun 2007 dan distribusi prediksi permukaan *Pf*PR_2-10_ (bandingkan Gambar 8B dan 8C). Secara khusus, distribusi yang disebutkan terakhir memiliki median yang lebih besar, kurang condong positif, dan untuk Amerika dan Afrika+ memiliki nilai maksimum yang secara nyata lebih kecil. Pergeseran yang mengarah kepada nilai dugaan permukaan *Pf*PR_2-10_ yang lebih tinggi dapat dijelaskan dengan pengelompokan spasial dari lokasi-lokasi survey. Harus selalu diingat bahwa sebagian data survey yang dikumpulkan mewakili sampel yang tersedia karena didorong oleh berbagai motivasi dan keterbatasan individu, organisasi dan pemerintah. Pemeriksaan secara visual terhadap bagian data tersebut menemukan bahwa sebagian besar survey itu dilakukan di wilayah dengan endemisitas yang lebih rendah dibandingkan dengan penarikan sampel acak secara spasial dan ringkasan statistik dari data mentah ini memperlihatkan adanya suatu bias yang besar. Dengan menduga endemisitas untuk permukaan kontinu, proses MBG memberikan kompensasi secara implisit untuk menggerombolkan kelompok ini pada peta keluaran dan dalam jalan yang sama distribusi frekuensi yang dihasilkannya menjadi tidak bias.

Proses MBG membuat pendugaan untuk lokasi-lokasi yang tidak diambil sampelnya menggunakan kombinasi linear dari data survey. Untuk alasan ini, permukaan yang dihasilkan menjadi lebih mulus daripada data mentah yang digunakan untuk pendugaan tersebut. Satu sifat dari proses pemulusan ini adalah kisaran nilai yang paling tinggi dan yang paling rendah pada permukaan dugaan tampak lebih kecil dibanding dengan yang ditampilkan oleh data input. Hal ini menjelaskan mengapa distribusi frekuensi pendugaan permukaan *Pf*PR_2-10_ mencakup kisaran nilai yang lebih kecil dibanding dengan data input. Implikasi penting dari efek pemulusan ini adalah bahwa pendugaan permukaan memberikan dugaan endemisitas yang lebih baik pada skala yang lebih besar tetapi kurang mampu untuk menampilkan perbedaan pada skala-kecil yang terjadi dalam jarak yang sangat dekat.

Penggunaan Peubah Lingkungan untuk Membuat Peta yang Berkesinambungan

Peubah-peubah iklim yang penting telah dimasukkan secara utuh dalam definisi batasan stabil dan tidak stabil untuk transmisi malaria *P. falsiparum* seperti yang tersebut di atas [18]. Ada ketertarikan menggunakan peubah-peubah lingkungan secara lebih jauh untuk meningkatkan kompleksitas dan meningkatkan akurasi pendugaan dalam pemetaan endemisitas MBG.

Hal ini disebabkan karena analisis-analisis seperti itu didasarkan pada asumsi bahwa distribusi dan endemisitas malaria saat ini sedang mendekati peranan pentingnya [79,80]. Asumsi ini tidak berdasar karena distribusi malaria global telah berkurang sangat banyak [18] sejak distribusi maksimumnya diperkirakan sekitar tahun 1900 [14]. Lebih jauh lagi, tidak diketahui dalam kondisi apa faktor-faktor penentu lingkungan dari distribusi yang masih ada ini menjelaskan seberapa penting peranan tersebut, bagaimana hubungan tersebut bervariasi secara spasial, dan artefak apa saja yang mungkin dimasukkan ke dalam analisis ini. Sebagai tambahan, merupakan suatu hal yang krusial untuk mendapatkan peubah-peubah lingkungan yang “memadai” pada skala global dengan tingkat keterandalan ruang dan waktu yang diperlukan [63,81]. Akhirnya, tingkatan ini semakin menjadi tidak jelas dengan adanya upaya-upaya intervensi yang masih berlangsung dan hubungannya dengan ruang menjadi tidak bisa dihitung. Semakin banyaknya bukti mengarah kepada fakta bahwa efek-efek intervensi ini memang banyak artinya, meningkat setelah periode 2000 dan menunjukkan suatu pengaruh spasial yang saling berkaitan itu akan banyak mengganggu hubungan apa saja yang dimodelkan [82-90]. Tak mengejutkan, tidak ada dukungan secara statistik yang dapat ditemukan untuk memasukkan kisaran iklim [62] dan peubah-peubah lingkungan dari pencitraan jarak jauh [63] (Protokol S1.7).

Untuk menghindari penggunaan peubah-peubah lingkungan dalam kerangka kerja analisis, hasil peta yang diperoleh hanya ditentukan oleh masukan data survey dan asumsi pemodelan. Pilihan ini menjamin upaya memaksimalkan prinsip parsimoni, dihadapkan dengan kemungkinan perubahan di masa datang.

Potensi Perbaikan Secara Geostatistik

Untuk menerima pendekatan MBG, alasan untuk mengeluarkan survey dengan jumlah sampel dibawah 50 menjadi berkurang, karena ketidakpastian dengan sampel populasi yang dimodelkan oleh tehnik ini (Protokol S3). Aturan pengeluaran ini direncanakan sebelum MBG dapat diaplikasikan pada skala global dan akan diperbaiki pada iterasi peta di masa mendatang. Penggunaan ekstrasi data yang sangat besar ini secara logistik tidak bisa dilakukan untuk pemetaan yang telah ada.

Resolusi spasial dimana tehnik MBG ini dapat diimplementasikan secara rasional untuk sekelompok komputer adalah grid 5x5 km. Keseluruhan proses memakan waktu rata-rata satu bulan untuk resolusi spasial ini dan diperkirakan akan memakan waktu sekitar 3 bulan untuk dijalankan pada resolusi spasial grid 1x1 km. Tidak ada rencana untuk meningkatkan resolusi spasial hasil peta pada skala global karena peta ini sangat mampu digunakan untuk tujuan perencanaan regional seperti yang dimaksud. Namun, untuk daerah yang lebih kecil, misalnya negara-negara dengan data *Pf*PR yang banyak dimana peta dengan resolusi spasial yang lebih tinggi diperlukan untuk mendukung rencana pengendalian secara nasional, keluaran MBG sampai grid 1x1 km dapat dipertimbangkan [33]. Lebih lanjut, pada skala nasional, keterandalan penentuan posisi geografis dari masukan data survey *Pf*PR mungkin memberi pengaruh penting terhadap ketidakpastian pendugaan, sehingga prosedur yang dapat menggabungkan efek-efek tersebut ke dalam model mungkin perlu juga untuk diselidiki [91-93]. Dalam penelitian ini, ketidakpastian mungkin disebabkan oleh kesalahan penentuan posisi geografis yang diperkirakan dulu tidak penting artinya dalam hubungannya dengan skala variasi spasial pada endemisitas yang teramati dan skala global dari keluaran model.

Kami tidak bisa meningkatkan kinerja prediksi model koreksi-umur dengan memodelkan sensitifitas yang tergantung kepada umur dari mikroskop dan uji diagnostik cepat secara terpisah atau dengan memodelkan spesifisitas diagnostik. Akurasi penentuan *Pf*PR secara mikroskopis atau dengan uji diagnostik cepat diasumsikan sama dalam analisa ini, tetapi sensitivitas tehnik diagnostik [94-98] dapat dimasukkan ke dalam interasi kerangka kerja MBG di masa mendatang.

Tidak ada solusi yang dapat dilakukan untuk mengaplikasikan tehnik MBG ini untuk wilayah dengan lautan yang besar (misalnya Karibia, Madagaskar dan kepulauan Indonesia), dengan adanya distribusi global data *Pf*PR dan kurangnya data di beberapa wilayah (Gambar 1). Pengaruh bio-geografis potensial terhadap transmisi malaria di pulau-pulau diabaikan dalam analisa ini. Idealnya, iterasi peta di masa mendatang akan memiliki data yang cukup untuk memperlakukan pulau-pulau tersebut secara terpisah atau informasi yang cukup tentang vektor *Anopheles* untuk membantu pendugaan tersebut [56].

Kami telah memasukkan kemampuan analisis ini untuk menyadari adanya pola sekuler dalam data *Pf*PR dan variasi transmisi tahunan. Meskipun demikian, peta ini tidak menyediakan gambaran lengkap dinamika malaria musiman [99-101], dan informasi lebih jauh tentang variasi global malaria musim mungkin dapat dimasukkan untuk iterasi peta di masa mendatang.

Rencana ke Depan: Domain Publik dan Peta Dinamis

Permukaan yang dipetakan ini akan tersedia untuk domain publik bersamaan dengan dipublikasikannya artikel ini. Data yang digunakan dalam prediksi ini akan ditampilkan untuk umum pada tahun 2009 [1] dan infrastruktur online untuk menampung layanan ini masih dalam tahap pengembangan. Tim MAP mengantisipasi untuk memperbarui setiap tahun peta global endemisitas malaria *P. falsiparum* dan basisdata *Pf*PR terkait. Pembaruan setiap tahun juga diperlukan untuk menggambarkan perubahan batas spasial transmisi stabil dan tidak stabil malaria *P. falsiparum* [18], untuk menentukan secara akurat batas-batas dimana prediksi endemisitas perlu dibuat. Jika komunitas internasional sukses menurunkan malaria, maka akan dibuat keputusan tentang terpisahnya antara batas spasial transmisi malaria *P. falsiparum* (ditentukan, bila memungkinkan, dengan nilai rata-rata dari *Pf*API dari catatan tiga tahun terakhir [18]) dan data endemisitas (*Pf*PR yang dikumpulkan sejak tahun 1985).

Merupakan hal yang jelas bahwa peta dugaan ini menampilkan gambaran endemisitas malaria tahun 2007 yang akan terus berubah seiring waktu. Tidak ada statistika canggih yang bisa menghindari fakta bahwa tambahan data akan meningkatkan reliabilitas peta ini, baik dengan meningkatkan resolusi spasial survey malariometrik ataupun dengan memperbaharui lokasi survey yang telah ada dengan informasi terbaru. Metode ini telah dikembangkan sedemikian rupa sehingga permukaan ini dapat diperbaharui dengan cepat. Dengan dipakainya lebih banyak pendekatan peubah tunggal maka berarti bahwa perubahan dalam iterasi peta di masa mendatang dapat dilakukan secara konsisten dengan memperoleh lebih banyak data di daerah dengan ketidakpastian tinggi (perubahan dalam tempat) atau perubahan akibat suksesnya intervensi atau penurunan penyakit (perubahan dalam waktu), dibandingkan dengan hubungan campuran spasial dan temporal dari data *Pf*PR_2-10_ dan peubah-peubah lingkungan.

Kami menganjurkan masuknya data tambahan pada data yang telah ada untuk memperbaiki peta pada wilayah-wilayah di mana akurasi spasial kami paling sedikit, dan data yang baru untuk terus melanjutkan menghasilkan peta yang selalu diperbaharui. Wilayah-wilayah dengan ketidakpastian paling tinggi saat ini diindikasikan sebagai pendekatan yang baik melalui kebalikan peluang prediksi kelas (Gambar 5), meskipun tugas di masa mendatang diarahkan untuk memperbaiki informasi ini. Oleh sebab itu, prioritas dalam waktu dekat adalah membuat peta kawasan yang menunjukkan lokasi optimal dari survey baru untuk memaksimalkan pengurangan ragam dalam permukaan endemisitas yang telah ada dengan biaya seminimum mungkin. Solusi ini lebih rumit dibandingkan dengan daftar wilayah dengan ragam tertinggi yang ditampilkan di sini sebab (i) setiap survey baru akan merubah struktur ragam spasial dan mempengaruhi lokasi optimal dari survey berikutnya; (ii) jumlah dan distribusi spasial survey akan mempengaruhi hasil dan membutuhkan simulasi berkali-kali untuk mengerucut pada solusi yang optimal dan (iii) lokasi survei yang potensial perlu diboboti dengan benar menggunakan distribusi populasi manusia.

Tujuan Malaria Atlas Project Dalam Waktu Dekat

Fokus awal MAP adalah *P. falsiparum* [1] karena pentingnya nilai epidemiologis secara global [102] dan karena prospek yang lebih baik dalam pengendalian dan eliminasi secara lokal [19]. Kami belum menyentuh sama sekali masalah yang penting tentang beban parasit *P. vivax* [103] meskipun diketahui beban klinisnya semakin meningkat [104-106], tetapi kami telah menyimpan lebih dari 2500 survey *P.vivax* parasite rate (*Pv*PR) sebagai langkah awal untuk memulai proses ini. Tujuan lainnya adalah untuk memperbaiki perkiraan beban global penyakit *P. falsiparum* (baik morbiditas [102] dan mortalitas [48,107,108]) untuk mendukung perkiraan kebutuhan komoditas dan intervensi antimalaria. Metode statistik dalam analisis ini memungkinkan iterasi lanjutan untuk memperkirakan dugaan beban secara menyeluruh dan dugaan ketidakpastian menjadi lebih baik. Untuk jangka menengah, kombinasi peta endemisitas global ini dengan peta distribusi dominan vektor malaria pada manusia *Anopheles* [56] seharusnya memberi kemampuan bagi para manajer pengendalian malaria untuk membuat keputusan yang dilandasi dengan informasi menyangkut intervensi yang sesuai dengan bionomik vektor lokal mereka sendiri. Untuk jangka panjang, kami berharap untuk tidak hanya memantau dan mengevaluasi kemajuan dengan peta ini, tetapi juga meningkatkan kemampuan kami untuk membuat model endemisitas malaria di masa mendatang dan mendukung penilaian obyektif di wilayah manapun di dunia yang memungkinkan untuk mengeliminasi malaria.

Kesimpulan

Situasi malaria *Plasmodium falsiparum* di dunia pada tahun 2007 memperlihatkan begitu banyaknya kesempatan bagi komunitas internasional untuk melakukan tindakan [109,110], tetapi tindakan-tindakan ini masih kurang sumber daya [111]. Terlepas dari bangsa mana yang akan menjadi juara, pengendalian secara intensif atau meraih impian yang lebih tinggi untuk menghapuskan malaria [2-4,74,112-114], jalur intervensi jangka menengah tampaknya serupa [19]. Sumber kartografik ini akan membantu banyak negara untuk menentukan kebutuhannya dan berperan sebagai dasar untuk memantau dan mengevaluasi kemajuan yang mengarah kepada tujuan intervensi. Kami berharap untuk terus bekerja sama dengan berbagai pihak, baik perorangan, negara dan wilayah untuk meningkatkan iterasi di masa datang dan berharap mendokumentasikan kesuksesan intervensi tersebut.

**Daftar Pustaka**

1. Hay SI, Snow RW (2006) The Malaria Atlas Project: developing global maps of malaria risk. PLoS Med 3: e473. doi:10.1371/journal.pmed.0030473

2. Horton (2007) Is malaria eradication possible? Lancet 370: 1459.

3. Roberts L, Enserink M (2007) Did they really say … eradication? Science 318: 1544-1545.

4. Feachem R, Sabot O (2008) A new global malaria eradication strategy. Lancet 10: 1633-1635.

5. Lysenko AJ, Semashko IN (1968) Geography of malaria. A medico-geographic profile of an ancient disease [in Russian]. Lebedew AW, editor. Itogi Nauki: Medicinskaja Geografija. Moscow: Academy of Sciences, USSR. pp. 25-146.

6. Boyd MF (1930) An introduction to malariology. Cambridge (Massachusetts): Harvard University Press.

7. Pampana EJ, Russell PF (1955) Le paludisme: problème mondial. Geneva: World Health Organization. pp. 317-321.

8. Macdonald G (1957) Local features of malaria. The epidemiology and control of malaria. London: Oxford University Press. pp. 63-99.

9. WHO (1966) Malaria eradication in 1965. World Health Organ Chron 20: 286-300.

10. Dutta HM, Dutt AK (1978) Malarial ecology: a global perspective. Soc Sci Med 12: 69-84.

11. WHO (1977) Information on the world malaria situation. Wkly Epidemiol Rec 52.

12. WHO (1994) World malaria situation in 1992. Part II. Wkly Epidemiol Rec 69: 317-324.

13. WHO (1997) World malaria situation in 1994. Part I. Wkly Epidemiol Rec 72: 269-274.

14. Hay SI, Guerra CA, Tatem AJ, Noor AM, Snow RW (2004) The global distribution and population at risk of malaria: past, present, and future. Lancet Infect Dis 4: 327-336.

15. Kiszewski A, Mellinger A, Spielman A, Malaney P, Sachs SE, et al. (2004) A global index representing the stability of malaria transmission. Am J Trop Med Hyg 70: 486-498.

16. Mouchet J, Carnevale P, Coosemans M, Julvez J, Manguin S, et al. (2004) Paludisme et grandes régions biogéographiques. Biodiversité du paludisme dans le monde. Montrouge (France): John Libbey Eurotext.

17. Guerra CA (2007) Mapping the contemporary global distribution limits of malaria using empirical data and expert opinion [Doctoral thesis]. Oxford: University of Oxford. 258 p.

18. Guerra CA, Gikandi PW, Tatem AJ, Noor AM, Smith DL, et al. (2008) The limits and intensity of *Plasmodium falciparum* transmission: implications for malaria control and elimination worldwide. PLoS Med 5: e38. doi:10.1371/journal.pmed.0050038

19. Hay SI, Smith DL, Snow RW (2008) Measuring malaria endemicity from intense to interrupted transmission. Lancet Infect Dis 8: 369-378.

20. Yekutiel P (1980) III The Global Malaria Eradication Campaign. Klingberg MA, editor. Eradication of infectious diseases: a critical study. Basel: Karger. pp. 34-88.

21. Pampana E (1969) A textbook of malaria eradication. London: Oxford University Press.

22. Kleinschmidt I, Bagayoko M, Clarke GPY, Craig M, Le Sueur D (2000) A spatial statistical approach to malaria mapping. Int J Epidemiol 29: 355-361.

23. Kleinschmidt I, Omumbo J, Briet O, van de Giesen N, Sogoba N, et al. (2001) An empirical malaria distribution map for West Africa. Trop Med Int Health 6: 779-786.

24. Rogers DJ, Randolph SE, Snow RW, Hay SI (2002) Satellite imagery in the study and forecast of malaria. Nature 415: 710-715.

25. Omumbo JA, Hay SI, Snow RW, Tatem AJ, Rogers DJ (2005) Modelling malaria risk in East Africa at high-spatial resolution. Trop Med Int Health 10: 557-566.

26. Rogers DJ (2006) Models for vectors and vector-borne diseases. Adv Parasitol 62: 1-35.

27. Diggle PJ, Tawn JA, Moyeed RA (1998) Model-based geostatistics. J Roy Stat Soc C-App 47: 299-326.

28. Diggle P, Moyeed R, Rowlingson B, Thomson M (2002) Childhood malaria in The Gambia: a case-study in model-based geostatistics. J Roy Stat Soc C-App 51: 493-506.

29. Gemperli A, Vounatsou P, Kleinschmidt I, Bagayoko M, Lengeler C, et al. (2004) Spatial patterns of infant mortality in Mali: the effect of malaria endemicity. Am J Epidemiol 159: 64-72.

30. Gemperli A, Vounatsou P, Sogoba N, Smith T (2006) Malaria mapping using transmission models: application to survey data from Mali. Am J Epidemiol 163: 289-297.

31. Rattanasiri S, Bohning D, Rojanavipart P, Athipanyakom S (2004) A mixture model application in disease mapping of malaria. Southeast Asian J Trop Med Public Health 35: 38-47.

32. Gosoniu L, Vounatsou P, Sogoba N, Smith T (2006) Bayesian modelling of geostatistical malaria risk data. Geospat Health 1: 127-139.

33. Noor AM, Clements ACA, Gething PW, Moloney G, Borle M, et al. (2008) Spatial prediction of *Plasmodium falciparum* prevalence in Somalia. Malar J 7: 159.

34. Clements ACA, Lwambo NJS, Blair L, Nyandindi U, Kaatano G, et al. (2006) Bayesian spatial analysis and disease mapping: tools to enhance planning and implementation of a schistosomiasis control programme in Tanzania. Trop Med Int Health 11: 490-503.

35. Clements ACA, Moyeed R, Brooker S (2006) Bayesian geostatistical prediction of the intensity of infection with *Schistosoma mansoni* in East Africa. Parasitology 133: 711-719.

36. Diggle PJ, Thomson MC, Christensen OF, Rowlingson B, Obsomer V, et al. (2007) Spatial modelling and the prediction of *Loa loa* risk: decision making under uncertainty. Ann Trop Med Parasitol 101: 499-509.

37. Beck-Worner C, Raso G, Vounatsou P, N’Goran EK, Rigo G, et al. (2007) Bayesian spatial risk prediction of *Schistosoma mansoni* infection in western Cote d’Ivoire using a remotely-sensed digital elevation model. Am J Trop Med Hyg 76: 956-963.

38. Raso G, Matthys B, N’Goran EK, Tanner M, Vounatsou P, et al. (2005) Spatial risk prediction and mapping of *Schistosoma mansoni* infections among schoolchildren living in western Cote d’Ivoire. Parasitology 131: 97-108.

39. Chilès J-P, Delfiner P (1999) Geostatistics: modeling spatial uncertainty. Toronto: John Wiley and Sons. 720 p.

40. Goovaerts P (1997) Geostatistics for natural resource evaluation. New York: Oxford University Press. 483 p.

41. Matheron G (1971) The theory of regionalized variables and its applications. Fontainebleau (France): Ecole Nationale Supérieure des Mines de Paris. 211 p.

42. Lawson AB (2001) Statistical methods in spatial epidemiology. Chichester: John Wiley and Sons. 277 p.

43. Best N, Richardson S, Thomson A (2005) A comparison of Bayesian spatial models for disease mapping. Stat Methods Med Res 14: 35-59.

44. Gething PW, Noor AM, Gikandi PW, Hay SI, Nixon MS, et al. (2008) Developing geostatistical space-time models to predict outpatient treatment burdens from incomplete national data. Geogr Anal 40: 167-188.

45. Gething PW, Noor AM, Goodman CA, Gikandi PW, Hay SI, et al. (2007) Information for decision making from imperfect national data: tracking major changes in health care use in Kenya using geostatistics. BMC Med 5: 37.

46. Wang XH, Zhou XN, Vounatsou P, Chen Z, Utzinger J, et al. (2008) Bayesian spatio-temporal modeling of *Schistosoma japonicum* prevalence data in the absence of a diagnostic ‘gold’ standard. PLoS Negl Trop Dis 2: e250. doi:10.1371/journal.pntd.0000250

47. Yang GJ, Vounatsou P, Zhou XN, Tanner M, Utzinger J (2005) A Bayesian-based approach for spatio-temporal modeling of county level prevalence of *Schistosoma japonicum* infection in Jiangsu province, China. Int J Parasitol 35: 155-162.

48. Hay SI, Guerra CA, Tatem AJ, Atkinson PM, Snow RW (2005) Urbanization, malaria transmission and disease burden in Africa. Nat Rev Microbiol 3: 81-90.

49. Robert V, MacIntyre K, Keating J, Trape JF, Duchemin JB, et al. (2003) Malaria transmission in urban sub-Saharan Africa. Am J Trop Med Hyg 68: 169-176.

50. Tatem AJ, Guerra CA, Kabaria CW, Noor AM, Hay SI (2008) Human population, urban settlement patterns and their impact *Plasmodium falciparum* malaria endemicity. Malar J 7: 218.

51. Smith DL, Guerra CA, Snow RW, Hay SI (2007) Standardizing estimates of the *Plasmodium falciparum* parasite rate. Malar J 6: 131.

52. Swaroop S (1959) Statistical considerations and methodology in malaria eradication. Part I. Statistical considerations. WHO/Mal/240. Geneva: World Health Organization.

53. Swaroop S (1959) Statistical considerations and methodology in malaria eradication. Part II. Statistical methodology. WHO/Mal/240. Geneva: World Health Organization.

54. Swaroop S, Gilroy AB, Uemura K (1966) Statistical methods in malaria eradication. Geneva: World Health Organization. 164 p.

55. Smith DL, Smith T, Hay SI (2009) Measuring malaria for elimination. Chapter 7. Shrinking the malaria map: a prospectus on malaria elimination. Santa Cruz (California): Malaria Elimination Group, University of California Santa Cruz. In press.

56. Hay SI, Sinka ME, Tatem AJ, Patil AP, Guerra CA, et al. (2009) Developing global maps of the dominant *Anopheles* vectors of human malaria. PLoS Med. In press.

57. Metselaar D, Van Thiel PH (1959) Classification of malaria. Trop Geogr Med 11: 157-161.

58. Guerra CA, Hay SI, Lucioparedes LS, Gikandi PW, Tatem AJ, et al. (2007) Assembling a global database of malaria parasite prevalence for the Malaria Atlas Project. Malar J 6: 17.

59. Pull JH, Grab B (1974) Simple epidemiological model for evaluating malaria inoculation rate and risk of infection in infants. Bull World Health Organ 51: 507-516.

60. Balk DL, Deichmann U, Yetman G, Pozzi F, Hay SI, et al. (2006) Determining global population distribution: methods, applications and data. Adv Parasitol 62: 119-156.

61. Center for International Earth Science Information Network, Columbia University/International Food Policy Research Institute/The World Bank/and Centro Internacional de Agricultura Tropical (2007) Global Rural Urban Mapping Project (GRUMP) alpha: Gridded Population of the World, version 2, with urban reallocation (GPW-UR). Available: http://sedac.ciesin.columbia.edu/gpw. Palisades (New York): Center for International Earth Science Information Network, Columbia University/International Food Policy Research Institute/The World Bank/and Centro Internacional de Agricultura Tropical.

62. New M, Lister D, Hulme M, Makin I (2002) A high-resolution data set of surface climate over global land areas. Clim Res 21: 1-25.

63. Scharlemann JPW, Benz D, Hay SI, Purse BV, Tatem AJ, et al. (2008) Global data for ecology and epidemiology: a novel algorithm for temporal Fourier processing MODIS data. PLoS One 3: e1408. doi:10.1371/journal.pone.0001408

64. Stein ML (2005) Space-time covariance functions. J Am Stat Assoc 100: 310-321.

65. Gething PW, Noor AM, Gikandi PW, Ogara EAA, Hay SI, et al. (2006) Improving imperfect data from health management information systems in Africa using space-time geostatistics. PLoS Med 3: e271. doi:10.1371/journal.pmed.0030271

66. Saito H, Goovaerts P (2000) Geostatistical interpolation of positively skewed and censored data in a dioxin-contaminated site. Environ Sci Technol 34: 4228-4235.

67. Brooker S, Hay SI, Bundy DA (2002) Tools from ecology: useful for evaluating infection risk models? Trends Parasitol 18: 70-74.

68. Moyeed RA, Papritz A (2002) An empirical comparison of kriging methods for nonlinear spatial point prediction. Math Geol 34: 365-386.

69. Hintze JL, Nelson RD (1998) Violin plots: a box plot-density trace synergism. Am Stat 52: 181-184.

70. U.N.P.D) (2005) World urbanization prospects: population database. http://esa.un.org/unup/. New York: United Nations Population Division.

71. Hay SI, Noor AM, Nelson A, Tatem AJ (2005) The accuracy of human population maps for public health application. Trop Med Int Health 10: 1073-1086.

72. U.N.P.D. (2006) World population prospects: the 2006 revision population database. http://esa.un.org/unpp/. New York: United Nations Population Division.

73. WHO (2007) Malaria elimination: a field manual for low and moderate endemic countries. Geneva: World Health Organization. 85 p.

74. Lines J, Whitty CJM, Hanson K (2007) Prospects for eradication and elimination of malaria: a technical briefing for DFID. London: London School of Hygiene & Tropical Medicine.

75. WHO. (2006) Informal consultation on malaria elimination: setting up the WHO agenda. WHO/HTM/MAL/2006.1114. Geneva: World Health Organization. 68 p.

76. Raso G, Vounatsou P, Gosoniu L, Tanner M, N’Goran EK, et al. (2006) Risk factors and spatial patterns of hookworm infection among schoolchildren in a rural area of western Côte d’Ivoire. Int J Parasitol 36: 201-210.

77. Gelman A, Carlin JB, Stern HS (2003) Bayesian Data Analysis. Texts in Statistical Science. Boca Raton (Florida): Chapman & Hall/CRC Press LLC. 696 p.

78. Gilks WR, Spiegelhalter DJ (1999) Markov Chain Monte Carlo in Practice. Interdisciplinary statistics. Boca Raton (Florida): Chapman & Hall/CRC Press LLC.

79. Hutchinson GE (1957) Concluding remarks. Cold Spring Harbor Symp Quant Biol 22: 415-427.

80. Southwood TRE (1977) Habitat, templet for ecological strategies? Presidential address to British Ecological Society, 5 January 1977. J Anim Ecol 46: 337-365.

81. Hay SI, Tatem AJ, Graham AJ, Goetz SJ, Rogers DJ (2006) Global environmental data for mapping infectious disease distribution. Adv Parasitol 62: 37-77.

82. Barat LM (2006) Four malaria success stories: how malaria burden was successfully reduced in Brazil, Eritrea, India, and Vietnam. Am J Trop Med Hyg 74: 12-16.

83. Barnes KI, Watkins WM, White NJ (2008) Antimalarial dosing regimens and drug resistance. Trends Parasitol 24: 127-134.

84. Bhattarai A, Ali AS, Kachur SP, Martensson A, Abbas AK, et al. (2007) Impact of artemisinin-based combination therapy and insecticide-treated nets on malaria burden in Zanzibar. PLoS Med 4: e309. doi:10.1371/journal.pmed.0040309

85. Fegan GW, Noor AM, Akhwale WS, Cousens S, Snow RW (2007) Effect of expanded insecticide-treated bednet coverage on child survival in rural Kenya: a longitudinal study. Lancet 370: 1035-1039.

86. Noor AM, Amin AA, Akhwale WS, Snow RW (2007) Increasing coverage and decreasing inequity in insecticide-treated bed net use among rural Kenyan children. PLoS Med 4: e255. doi:10.1371/journal.pmed.0040255

87. Nyarango PM, Gebremeskel T, Mebrahtu G, Mufunda J, Abdulmumini U, et al. (2006) A steep decline of malaria morbidity and mortality trends in Eritrea between 2000 and 2004: the effect of combination of control methods. Malar J 5: 33.

88. Okiro EA, Hay SI, Gikandi PW, Sharif SK, Noor AM, et al. (2007) The decline in paediatric malaria admissions on the coast of Kenya. Malar J 6: 151.

89. Schellenberg D, Menendez C, Aponte J, Guinovart C, Mshinda H, et al. (2004) The changing epidemiology of malaria in Ifakara Town, southern Tanzania. Trop Med Int Health 9: 68-76.

90. Noor AM, Moloney G, Borle M, Fegan GW, Shewchuk T, et al. (2008) The use of mosquito nets and the prevalence of *Plasmodium falciparum* infection in rural South Central Somalia. PLoS One 3: e2081. doi:10.1371/journal.pone.0002081

91. Chapman AD, Wieczorek J (2006) Guide to best practices for georeferencing. Copenhagen: Global Biodiversity Information Facility.

92. Guralnick RP, Wieczorek J, Beaman R, Hijmans RJ (2006) BioGeomancer: automated georeferencing to map the world’s biodiversity data. PLoS Biol 4: e381. doi:10.1371/journal.pbio.0040381

93. Wieczorek J, Guo Q, Hijmans RJ (2004) The point-radius method for georeferencing locality descriptions and calculating associated uncertainty. Int J Geogr Inf Sci 18: 745-767.

94. O’Meara WP, Collins WE, McKenzie FE (2007) Parasite prevalence: a static measure of dynamic infections. Am J Trop Med Hyg 77: 246-249.

95. Wongsrichanalai C, Barcus MJ, Muth S, Sutamihardja A, Wernsdorfer WH (2007) A review of malaria diagnostic tools: microscopy and rapid diagnostic test (RDT). Am J Trop Med Hyg 77: 119-127.

96. Zurovac D, Midia B, Ochola SA, English M, Snow RW (2006) Microscopy and outpatient malaria case management among older children and adults in Kenya. Trop Med Int Health 11: 432-440.

97. Fogg C, Twesigye R, Batwala V, Piola P, Nabasumba C, et al. (2008) Assessment of three new parasite lactate dehydrogenase (pan-pLDH) tests for diagnosis of uncomplicated malaria. Trans R Soc Trop Med Hyg 102: 25-31.

98. Metzger WG, Vivas-Martinez S, Rodriguez I, Goncalves J, Bongard E, et al. (2008) Malaria diagnosis under field conditions in the Venezuelan Amazon. Trans R Soc Trop Med Hyg 102: 20-24.

99. Hay SI, Omumbo JA, Craig MH, Snow RW (2000) Earth observation, geographic information systems and *Plasmodium falciparum* malaria in sub-Saharan Africa. Adv Parasitol 47: 173-215.

100. Hay SI, Snow RW, Rogers DJ (1998) Predicting malaria seasons in Kenya using multitemporal meteorological satellite sensor data. Trans R Soc Trop Med Hyg 92: 12-20.

101. Hay SI, Snow RW, Rogers DJ (1998) From predicting mosquito habitat to malaria seasons using remotely sensed data: Practice, problems and perspectives. Parasitol Today 14: 306-313.

102. Snow RW, Guerra CA, Noor AM, Myint HY, Hay SI (2005) The global distribution of clinical episodes of *Plasmodium falciparum* malaria. Nature 434: 214-217.

103. Price RN, Tjitra E, Guerra CA, Yeung S, White NJ, et al. (2007) Vivax malaria: neglected and not benign. Am J Trop Med Hyg 77: 79-87.

104. Tjitra E, Anstey NM, Sugiarto P, Warikar N, Kenangalem E, et al. (2008) Multidrug-resistant *Plasmodium vivax* associated with severe and fatal malaria: a prospective study in Papua, Indonesia. PLoS Med 5: e128. doi:10.1371/journal.pmed.0050128

105. Rogerson SJ, Carter R (2008) Severe vivax malaria: newly recognised or rediscovered. PLoS Med 5: e136. doi:10.1371/journal.pmed.0050136

106. Genton B, D’Acremont V, Rare L, Baea K, Reeder JC, et al. (2008) *Plasmodium vivax* and mixed infections are associated with severe malaria in children: a prospective cohort study from Papua New Guinea. PLoS Med 5: e127. doi:10.1371/journal.pmed.0050127

107. Rowe AK, Rowe SY, Snow RW, Korenromp EL, Schellenberg JRA, et al. (2006) The burden of malaria mortality among African children in the year 2000. Int J Epidemiol 35: 691-704.

108. Snow RW, Craig M, Deichmann U, Marsh K (1999) Estimating mortality, morbidity and disability due to malaria among Africa’s non-pregnant population. Bull World Health Organ 77: 624-640.

109. The Global Fund to Fight AIDS, Tuberculosis and Malaria (GFTAM) (2007) The Global Fund. Who we are what we do. Geneva: The Global Fund to Fight AIDS, Tuberculosis and Malaria (GFTAM). 36 p.

110. Grabowsky M (2008) The billion-dollar malaria moment. Nature 451: 1051-1052.

111. Snow RW, Guerra CA, Mutheu JJ, Hay SI (2008) International funding for malaria control in relation to populations at risk of stable *Plasmodium falciparum* transmission. PLoS Med 5: e142. doi:10.1371/journal.pmed.0050142

112. Tanner M, de Savigny D (2008) Malaria eradication back on the table. Bull World Health Organ 86: 82.

113. Greenwood BM, Fidock DA, Kyle DE, Kappe SH, Alonso PL, et al. (2008) Malaria: progress, perils, and prospects for eradication. J Clin Investig 118: 1266-1276.

114. Roll Back Malaria Partnership (R.B.M.P) (2008) The global malaria action plan for a malaria free world. Geneva: Roll Back Malaria Partnership (R.B.M.P), World Health Organization. 274 p.

**Penghargaan**

Pengumpulan data survey prevalensi parasit secara global sangat tergantung kepada kemurahan hati dari banyak orang dalam komunitas penelitian dan pengendalian malaria dan orang-orang ini terdaftar dalam situs MAP (http://www.map.ac.uk/acknowledgements.html). Kami juga berterima kasih kepada Archie Clements atas komentarnya atas manuskrip ini. Para penulis memberikan penghargaan atas dukungan Kenyan Medical Research Institute (KEMRI) dan tulisan ini dipublikasikan dengan ijin dari direktur KEMRI.

**Pendanaan**

SIH didanai oleh Senior Research Fellowship dari Wellcome Trust (Nomor 079091) yang juga mendukung CAG, AJT, dan PWG. AMN didukung oleh Wellcome Trust sebagai Research Training Fellow (Nomor 081829). BHM dan IRFE mendapatkan dukungan dari yayasan Li Ka Shing. SB didanai oleh Wellcome Trust sebagai Career Development Fellow (Nomor 081673). RWS adalah Wellcome Trust Principal Research Fellow (Nomor 079080). Bantuan ini juga mendukung AP. Pekerjaan ini merupakan bagian dari hasil Malaria Atlas Project (MAP, http://www.map.ox.ac.uk), utamanya didanai oleh Wellcome Trust, U.K. Pemberi dana tidak mempunyai peranan dalam merancang penelitian, pengumpulan data dan analisis, keputusan publikasi, atau persiapan manuskrip.

**Kontribusi Penulis**

SIH dan RWS menyusun penelitian ini. SB dan RAM memiliki peran sebagai penasihat dalam bidang statistika secara keseluruhan. PWG, APP, dan AJT memperhalus dan menjalankan protokol penelitian. AJT, AP dan PWG memperbaiki dan menjalankan protokol penelitian ini. DS mengembangkan prosedur standarisasi-umur. SIH, CAG, AMN, CWK, BHM, IRFE dan RWS mengkompilasi dan memetakan data *Pf*PR. SIH menulis draft pertama dari manuskrip ini. SIH, CAG, AMN, PWG, APP, AJT, AMN, CWK, BHM, IRFE, SB, DLS, RAM dan RWS memberikan komentar atas draft akhir manuskrip.

**Benturan Kepentingan**

Tidaka ada benturan kepentingan dari para penulis.

**Singkatan**

Afrika+	Africa, Yemen and Saudi Arabia

API	Annual Parasite Incidence

AUC	Area-Under-Curve

CSE Asia	Central and South East Asia

GPW	Gridded Population of the World

GRUMP	Global Rural-Urban Mapping Project

ITN	Insecticide Treated Net

MAP	Malaria Atlas Project

MBG	Model Based Geostatistics

PAR	Population At Risk

*Pf*API	*P. falciparum* Annual Parasite Incidence

*Pf*PR	*P. falciparum* Parasite Rate

*Pv*PR	*P. vivax* Parasite Rate

UE	Urban Extent

**Tabel 1**. Ringkasan statistik validasi untuk menduga *Pf*PR_2-10_ berdasarkan kawasan.

Nilai tengah setiap sebaran posterior dugaan digunakan sebagai titik dugaan *Pf*PR_2-10_ untuk dibandingkan dengan nilai amatan. Nilai di dalam tanda kurung menunjukkan persentase nilai tengah kawasan yang ditunjukkan oleh nilai galat yang bersangkutan. Lihat teks untuk penjelasan detail tentang penurunan statistik ini dan interpretasi hasilnya.

doi:10.1371/journal.pmed.1000048.t001

| **Ukuran validasi** | **Amerika** | **Afrika+** | **CSE Asia** | **Dunia** |
| --- | --- | --- | --- | --- |
|  |  |  |  |  |
| Galat nilai tengah | 0.627 (20.401) | 0.805 (3.070) | 1.184 (9.203) | 0.912 (4.267) |
| Galat nilai tengah | 3.522 (114.639) | 11.023 (42.024) | 7.705 (59.860) | 9.750 (45.625) |
| mutlak |  |  |  |  |
| Korelasi | 0.032 | 0.822 | 0.699 | 0.823 |

**Tabel 2**. Ringkasan statistik validasi untuk menduga kelas endemisitas *Pf*PR_2-10_ berdasarkan kawasan.

Lihat teks untuk penjelasan detail tentang penurunan statistik ini dan interpretasi hasilnya.

doi:10.1371/journal.pmed.1000048.t002

| **Ukuran validasi** | **Amerika** | **Afrika+** | **CSE Asia** | **Dunia** |
| --- | --- | --- | --- | --- |
|  |  |  |  |  |
| AUC (≤5%) | 0.452 | 0.927 | 0.885 | 0.915 |
| AUC (5%-<40%) | 0.452 | 0.758 | 0.813 | 0.779 |
| AUC (≥40%) | - | 0.921 | 0.842 | 0.927 |
| % benar secara keseluruhan | 80.000 | 70.621 | 69.874 | 70.750 |
| ≤5% dimasukkan sebagai ≥40% (%) | 0.000 | 0.188 | 0.418 | 0.250 |
| ≥40% dimasukkan sebagai ≤5% (%) | 0.000 | 0.377 | 2.092 | 0.875 |

**Tabel 3**. Wilayah dengan resiko malaria *Plasmodium falsiparum* tahun 2007.

Luas wilayah dinyatakan dalam jutaan km^2^. Resiko tidak stabil (*Pf*API <0.1 per 1.000 orang pa) dan resiko stabil (*Pf*API ≥0.1 per 1.000 orang pa). Resiko stabil dibagi ke dalam tiga standarisasi-umur [51] dan pengendalian yang berhubungan dengan kelas endemisitas *Pf*PR_2-10_ [19].

doi:10.1371/journal.pmed.1000048.t003

| **Kawasan** | **Resiko tidak stabil** | **Resiko**  **stabil** | ***Pf*PR_2-10_** ≤**5%** | ***Pf*PR_2-10_ >5 sampai <40%** | ***Pf*PR_2-10_** ≥**40%** | **Total** |
| --- | --- | --- | --- | --- | --- | --- |
|  |  |  |  |  |  |  |
| Amerika | 2.13 | 6.03 | 6.03 | 0.00 | 0.00 | 8.17 |
| Afrika+ | 4.21 | 18.17 | 4.03 | 5.63 | 8.50 | 22.37 |
| CSE Asia | 3.40 | 5.53 | 4.72 | 0.69 | 0.11 | 8.93 |
| Dunia | 9.74 | 29.72 | 14.79 | 6.32 | 8.61 | 39.46 |

**Tabel 4**. PAR malaria *P. falsiparum* tahun 2007.

Jumlah populasi dinyatakan dalam jutaan. Resiko tidak stabil (*Pf*API <0.1 per 1.000 orang pa) dan resiko stabil (*Pf*API ≥0.1 per 1.000 orang pa). Resiko stabil dibagi ke dalam tiga standarisasi-umur [51] dan pengendalian yang berhubungan dengan kelas endemisitas *Pf*PR_2-10_ [19]. Untuk setiap kawasan PAR dibagi lagi atas kelompok umur 0-4 tahun, 5-14 tahun dan 15+ tahun.

**_†_**Perlu dicatat bahwa kendala komputasi yang memerlukan penarikan sampel ulang batas spasial sampai resolusi spasial 5×5 km, pada akhirnya menghasilkan sedikit perubahan terhadap total populasi global yang beresiko, sebelumnya diduga pada 1×1 km [18].

doi:10.1371/journal.pmed.1000048.t004

| Kawasan | Kelompok Umur | Resiko Tidak Stabil**^a^** | Resiko Stabil**^a^** | *Pf*PR_2−10_  ≤ 5% | *Pf*PR_2−10_  > 5 sampai < 40% | *Pf*PR_2−10_  ≥ 40% | Total |
| --- | --- | --- | --- | --- | --- | --- | --- |
| Amerika | 0–4 | 5.62 | 4.51 | 4.51 | 0.00 | 0.00 | 10.14 |
|  | 5–14 | 10.66 | 8.66 | 8.66 | 0.00 | 0.00 | 19.32 |
|  | 15+ | 33.78 | 27.47 | 27.47 | 0.00 | 0.00 | 61.25 |
|  | Total | 50.06 | 40.64 | 40.64 | 0.00 | 0.00 | 90.71 |
| Afrika+ | 0–4 | 3.29 | 108.64 | 15.48 | 32.90 | 60.26 | 111.93 |
|  | 5–14 | 5.64 | 170.80 | 25.35 | 51.48 | 93.98 | 176.44 |
|  | 15+ | 12.95 | 377.17 | 73.67 | 112.45 | 191.05 | 390.12 |
|  | Total | 21.88 | 656.61 | 114.50 | 196.83 | 345.28 | 678.49 |
| CSE Asia | 0–4 | 99.69 | 73.70 | 64.98 | 8.10 | 0.62 | 173.38 |
|  | 5–14 | 195.40 | 143.20 | 126.26 | 15.67 | 1.27 | 338.60 |
|  | 15+ | 616.17 | 468.75 | 412.37 | 51.52 | 4.86 | 1,084.93 |
|  | Total | 911.26 | 685.65 | 603.61 | 75.29 | 6.75 | 1,596.91 |
| Dunia | 0–4 | 108.60 | 186.85 | 84.97 | 41.00 | 60.88 | 301.31 |
|  | 5–14 | 211.70 | 322.66 | 160.26 | 67.15 | 95.25 | 560.40 |
|  | 15+ | 662.90 | 873.40 | 513.51 | 163.97 | 195.91 | 1,504.41 |
|  | Total | 983.20 | 1,382.91 | 758.75 | 272.13 | 352.04 | 2,366.11 |

**Gambar 1.** Batasan Spasial Resiko Malaria *Plasmodium falsiparum* Ditentukan Melalui *Pf*API Dengan Intelijen Medis, Suhu dan Tingkat Kekeringan.

Wilayah-wilayah ditetapkan sebagai stabil (wilayah dengan abu-abu tua, dimana *Pf*API ≥0.1 per 1.000 pa), tidak stabil (wilayah dengan abu-abu sedang, dimana *Pf*API <0.1 per 1.000 pa) atau tidak ada resiko (abu-abu muda, dimana *Pf*API = 0 per 1.000 pa) [17-19]. Survey prevalensi komunitas *P. falsiparum* dilakukan antara 1 Januari 1985 dan 31 Juli 2008. Dari 8,938 survey yang terkumpul, 7,953 memenuhi kriteria untuk dimasukkan kedalam pemodelan (Lihat Metode dan Protokol S1.2). Data survey yang ditampilkan adalah (*Pf*PR_2-10_) dengan standarisasi-umur [51] dan ditampilkan sebagai rangkaian mulai dari warna kuning sampai merah dari 0-100% (lihat legenda peta). Garis-garis putus memisahkan Amerika; Afrika+; dan CSE Asia.

doi:10.1371/journal.pmed.1000048.g001

**Gambar 2.** Tinjauan Sistematis Prosedur dan Metode Pemetaan.

Wajik biru menjelaskan data input. Kotak orange menunjukkan model dan prosedur penelitian; S1, Protokol S1; S2, Protokol S2; S3, Protokol S3; dan S4, Protokol S4. Kotak hijau menunjukkan data keluaran; garis putus keluaran antara, garis penuh keluaran final. U, urban; PU, peri-urban; and R, rural.

doi:10.1371/journal.pmed.1000048.g002

**Gambar 3.** Distribusi Spasial Endemisitas Malaria *Plasmodium falsiparum*.

Data tersebut merupakan titik dugaan model-berbasis geostatistika dari nilai tengah tahunan *Pf*PR_2-10_ untuk tahun 2007 di dalam batas spasial transmisi stabil malaria *P. falsiparum*, diperlihatkan sebagai perubahan warna kuning ke merah dari 0-100% (lihat legenda peta). Sisa dari wilayah daratan tersebut ditetapkan sebagai resiko tidak stabil (wilayah dengan abu-abu sedang, dimana *Pf*API <0.1 per 1,000 pa) atau tidak ada resiko (abu-abu muda, dimana *Pf*API = 0 per 1.000 pa) [17-19].

doi:10.1371/journal.pmed.1000048.g003

**Gambar 4.** Distribusi Spasial Prediksi Malaria *Plasmodium falsiparum* *Pf*PR_2-10_ Berdasarkan Kelas Endemisitas

Mereka dikelompokkan ke dalam resiko rendah *Pf*PR_2-10_ ≤5%, merah muda; resiko sedang *Pf*PR_2-10_ >5% sampai <40%, merah sedang; dan resiko tinggi *Pf*PR_2-10_ ≥40% merah tua. Peta ini memperlihatkan kelas dimana *Pf*PR_2-10_ memilki peluang dugaan keanggotaan tertinggi. Sisa dari wilayah daratan tersebut ditetapkan sebagai resiko tidak stabil (wilayah dengan abu-abu sedang, dimana *Pf*API <0.1‰ pa) atau tidak ada resiko (abu-abu muda, dimana *Pf*API = 0 per 1.000 pa) [17-19].

doi:10.1371/journal.pmed.1000048.g004

**Gambar 5.** Peta Ketidakpastian Model

(A). Peluang *Pf*PR_2-10_ masuk ke dalam kelas yang telah ditentukan kemudian dipetakan dan diperlihatkan sebagai rangkain dari warna kuning ke biru dari . Nilai yang berada diatas lebih baik daripada dilakukan pengalokasian secara acak. Sisa dari wilayah daratan tersebut ditetapkan sebagai resiko tidak stabil (wilayah dengan abu-abu sedang, dimana *Pf*API <0.1 per seribu pa) atau tidak ada resiko (abu-abu muda, dimana *Pf*API = 0 per 1.000 pa) [17,18,19].

(B) Indeks ketidakpastian populasi-terboboti. Indeks memperlihatkan seberapa penting ketidakpastian dinilai dari perkalian antara log densitas populasi (Protokol S2.3) dan kebalikan dari peluang penentuan kelas secara benar, memiliki skala dari 0-1. Indeks ini disajikan untuk kelas endemisitas *Pf*PR_2-10_ yang paling mungkin. Resiko tidak stabil dan tidak ada resiko diperlihatkan di (A).

doi:10.1371/journal.pmed.1000048.g005

**Gambar 6.** Plot-Plot Validasi Model

(A) Plot pencar nilai aktual terhadap nilai-nilai dugaan titik *Pf*PR_2-10_.

(B) Semi-variogram sampel dari sisaan model Pearson dibakukan diduga pada selisih diskret (lingkaran) dan dibandingkan dengan amplop Monte Carlo (garis putus-putus) memperlihatkan kisaran nilai yang diharapkan terjadi secara acak dalam kondisi tanpa autokorelasi spasial.

(C) Kurva *Receiver-Operating Characteristic* untuk masing-masing kelas endemisitas *Pf*PR_2-10_ (garis hitam *Pf*PR_2-10_ ≤5%, garis merah *Pf*PR_2-10_ >5% sampai <40%, garis hijau *Pf*PR_2-10_ ≥40%) dan AUC yang berhubungan.

(D) Plot Peluang-Peluang membandingkan ambang batas peluang dugaan dengan persentase aktual dari nilai sebenarnya yang melampaui nilai ambang batas ini. Dalam plot atas dan kanan bawah, garis 1:1 juga ditampilkan (garis putus-putus) sebagai acuan. Lihat teks untuk penjelasan detail tentang prosedur validasi dan interpretasi hasilnya.

doi:10.1371/journal.pmed.1000048.g006

**Gambar 7.** Diagram Kue Memperlihatkan Populasi Resiko Malaria (PAR) *P. falsiparum* Tahun 2007

Diagram memperlihatkan proporsi populasi yang tinggal di setiap kelas endemisitas dugaan *Pf*PR_2-10_ untuk kawasan Amerika, Afrika+, CSE Asia dan seluruh dunia. Diagram-diagram ini diatur besarnya secara proporsional terhadap populasi total di setiap kawasan dan setiap bagian diwarnai sesuai dengan kelas endemisitas seperti yang terlihat di Gambar 4.

doi:10.1371/journal.pmed.1000048.g007

**Gambar 8.** Plot Violin Memperlihatkan Distribusi Frekuensi Data *Pf*PR_2-10_ Untuk Setiap Kawasan

(A) untuk semua tahun, (B) untuk tahun 2007, dan (C) untuk permukaan pendugaan tahun 2007. Lebar dari setiap plot poligon menggambarkan frekuensi relatif berbagai nilai *Pf*PR_2-10_ untuk setiap kawasan. Latar belakang diberi warna disesuaikan dengan kelas endemisitas seperti yang terlihat dalam Gambar 4. Batang tengah hitam mengindikasikan kisaran antar-kuartal dan lingkaran putih menunjukkan nilai median (lihat teks).

doi:10.1371/journal.pmed.1000048.g008

**Informasi Pendukung**

**Bahasa Alternatif Teks S1**.

Terjemahan artikel ke dalam bahasa Perancis oleh Frédéric Piel and Stéphanie Loute

Tersedia di doi:10.1371/journal.pmed.1000048.sd001 (1.04 MB DOC).

**Bahasa Alternatif Teks S2.**

Terjemahan artikel ke dalam bahasa China oleh Robert Li

Tersedia di doi:10.1371/journal.pmed.1000048.sd002 (437 KB DOC).

**Bahasa Alternatif Teks S3.**

Terjemahan artikel ke dalam bahasa Indonesia oleh Iqbal R.F. Elyazar dan Siti Nurlela

Tersedia di doi:10.1371/journal.pmed.1000048.sd003 (1.07 MB DOC).

**Bahasa Alternatif Teks S4.**

Terjemahan artikel ke dalam bahasa Vietnam oleh Bui H. Manh

Tersedia di doi:10.1371/journal.pmed.1000048.sd004 (574 KB DOC).

**Bahasa Alternatif Teks S5.**

Terjemahan artikel ke dalam bahasa Spanyol oleh Carlos A. Guerra

Tersedia di doi:10.1371/journal.pmed.1000048.sd005 (774 KB DOC).

**Protokol S1**. Basisdata Survey Malariometrik *PfPR*

S1.1 Ringkasan prosedur pencarian data dan pengambilan intisari data

S1.2 Aturan Eklusi Data

S1.3 Standarisasi Umur

S1.4 Semi-Variogram data *Pf*PR_2-10_ berdasarkan Kawasan

S1.5 Penyaringan secara Geostatistika untuk Mendeteksi Pencilan Jauh

S1.6 Ringkasan Data Survey Malariometrik dan Statistika Deskriptif

S1.7 Hubungan dengan Peubah-Peubah Lingkungan

Tersedia doi:10.1371/journal.pmed.1000048.sd005 (3.4 MB DOC).

**Protokol S2**. Basisdata Demografis dan Prosedur

S2.1 Aturan Klasifikasi Urban/Peri-Urban/Rural Survey Parasit

S2.2 Status Urban/Peri-Urban/Rural dan Prevalensi

S2.3 Populasi Penduduk GRUMP alpha

S2.4 Cara Mendapatkan PAR

Tersedia di doi:10.1371/journal.pmed.1000048.sd006 (2.5 MB DOC).

**Protokol S3**. Prosedur Model berbasis Geostatistik

S3.1 Tinjauan Model Statistika

S3.2 Spesifikasi Pendahuluan

S3.3 Standarisasi Umur

S3.4 Detail Implementasi

S3.5 Tinjauan Pembuatan Peta

Tersedia di doi:10.1371/journal.pmed.1000048.sd007 (23 MB DOC).

**Protokol S4**. Prosedur Validasi Model

S4.1 Membuat Data untuk Validasi

S4.2 Prosedur untuk Menguji Kinerja Model

S4.3 Hasil-hasil Tambahan

Tersedia di doi:10.1371/journal.pmed.1000048.sd008 (26 MB DOC).
