# Supplementary material for: A World Malaria Map: Plasmodium falciparum Endemicity in 2007
Source: PLoS Med. 2009 Mar 24;6(3):e1000048. doi: 10.1371/journal.pmed.1000048 (PMC2659708; doi:10.1371/journal.pmed.1000048)
Supplement: Supplementary file 4 [file pmed.1000048.sd004.doc]

**Bản đồ sốt rét thế giới: Tỷ lệ lưu hành *Plasmodium falciparum* năm 2007**

Simon I. Hay^1,2*^, Carlos A. Guerra^1,2^, Peter W. Gething^2,3^, Anand P. Patil^2^, Andrew J. Tatem^1,2,4, 5^, Abdisalan M. Noor^1,6^, Caroline W. Kabaria^1^, Bui H. Manh^7^, Iqbal R. F. Elyazar^8^, Simon Brooker^1,9^, David L. Smith^5,10^, Rana A. Moyeed^11^ and Robert W. Snow^1,6^

1. Nhóm dịch tễ học và sức khỏe công cộng về sốt rét, Trung tâm Y học Địa lý, Viện Nghiên cứu Y học Kenya (KEMRI) – Đại học Oxford – Chương trình hợp tác Wellcome Trust, Nairobi, Kenya

2. Nhóm Dịch tễ học và Sinh thái học không gian, tòa nhà Tinbergen, Khoa Động vật học, Đại học Oxford, Đường South Parks, Oxford, Anh Quốc

3. Trung tâm Nghiên cứu sức khỏe Địa lý, Trường Địa lý, Đại học Southampton, Highfield, Southampton, Anh Quốc

4. Khoa Địa lý, Đại học Florida, Gainsville, Florida, Hoa Kỳ

5. Viện Nghiên cứu sự bùng nổ các tác nhân gây bệnh, Đại học Florida, Gainsville, Florida, Hoa Kỳ

6. Trung tâm Y khoa Nhiệt đới, Khoa Y học lâm sàng Nuffield, Đại học Oxford, Trung tâm vacxin học lâm sàng và Y học Nhiệt đới (CCVTM), Oxford, Anh quốc

7. Đơn vị Nghiên cứu Lâm sàng trường Đại học Oxford, Bệnh viện Bạch Mai, Viện các bệnh Truyền nhiễm và bệnh Nhiệt đới quốc gia, 78 Giải Phóng – Hà Nội, Việt Nam

8. Đơn vị Nghiên cứu Lâm sàng Eijkman-Oxford, Jalan Diponegoro No. 69, Jakarta 10430, Indonesia

9. Khoa các bệnh Truyền nhiễm và bệnh Nhiệt đới, Department of Infectious and Tropical Diseases, Trường Đại học Vệ sinh và Y học Nhiệt đới London, Anh Quốc

10. Khoa Động vật, Đại học Florida, Gainsville, Florida, Hoa Kỳ

11. Trường toán học và Thống kê, Đại học Plymouth, Drake Circus, Plymouth, Devon, Anh Quốc

Mức độ lưu hành sốt rét toàn cầu

**Tài trợ**: SIH được tài trợ bởi Quỹ học bổng nghiên cứu cao cấp của Wellcome Trust (#079091), cũng là học bổng hỗ trợ cho CAG, AJT và PWG. AMN được Wellcome Trust hỗ trợ dưới dạng Nghiên cứu sinh (#081829). BHM và IRFE cám ơn hỗ trợ của quỹ Li Ka Shing. SB được Wellcome Trust tài trợ dưới dạng Nghiên cứu sinh phát triển nghề (#081673). RWS là Nghiên cứu sinh chính của Wellcome Trust (#079080). Ngân sách tài trợ này cũng hỗ trợ cho AP. Nghiên cứu này là một phần của kết quả của Dự án Atlas bệnh sốt rét (MAP, [http://www.map.ox.ac.uk](http://www.map.ox.ac.uk/)), chủ yếu do Wellcome Trust, Anh Quốc tài trợ. Các nhà tài trợ hoàn toàn không đóng vai trò gì trong thiết kế nghiên cứu, thu thập dữ liệu và phân tích, quyết định xuất bản, hoặc chuẩn bị bản thảo.

**Cạnh tranh**: Không có cạnh tranh nào do bất kỳ tác giả nào tuyên bố.

**Biên tập học thuật**: Ivo Mueller, Viện Nghiên cứu Y học Papua New Guinea, Papua New Guinea

**Trích dẫn**: Hay SI, Guerra CA, Gething PW, Patil AP, Tatem AJ, et al. (2009) A world malaria map: *Plasmodium falciparum* endemicity in 2007. PLoS Med 6(3): e1000048. doi:10.1371/journal.pmed.1000048

**Nhận**: ngày 10 tháng Mười năm 2008

**Chấp nhận**: ngày 2 tháng Hai năm 2009

**Xuất bản**:

**Bản quyền**: © 2009 Hay và ctv. Đây là tài liệu mở được phân phối theo Giấy phép Creative Commons Attribution, cho phép sử dụng, phân phối và sao chép không giới hạn dưới bất kỳ hình thức nào, với điều kiện tác giả gốc và nguồn tài liệu được trích dẫn rõ.

**Các chữ viết tắt:** Châu Phi+, Châu Phi, Yemen và A-rập Xê-út; AUC, Area-Under-Curve (Diện tích dưới đường cong biểu diễn); TDNA, Trung, Đông và Nam Á; GRUMP, Global Rural-Urban Mapping Project (Dự án lập bản đồ đô thị-nông thôn toàn cầu); ITN, Insecticide Treated Net (Màn tẩm hóa chất); MAP, Malaria Atlas Project (Dự án Atlas bệnh sốt rét); MBG, Model Based Geostatistics (Địa thống kê dựa trên cơ sở mô hình hóa); PA, Per Annum (trên năm); PAR; Population At Risk (Dân số trong vùng có nguy cơ); *Pf*API; *P. falciparum* Annual Parasite Incidence (Tỷ lệ nhiễm ký sinh trùng *P. falciparum* hàng năm); PfPR, *P. falciparum* parasite index (Tỷ lệ nhiễm ký sinh trùng *P. falciparum*); *Pv*API; *P.vivax* Annual Parasite Incidence (Tỷ lệ lệ nhiễm ký sinh trùng *P. vivax* hàng năm); UE, Urban Extent (Phạm vi của vùng đô thị)**.**

**Tóm tắt**

Việc phân bổ hiệu quả tài nguyên để ngăn chặn sốt rét đòi hỏi hiểu biết chi tiết về phân bố không gian hiện thời của nguy cơ sốt rét. Đã trải qua đúng bốn mươi năm kể từ khi bản đồ toàn cầu về tỷ lệ lưu hành của sốt rét được công bố. Bài viết này mô tả phương pháp tạo bản đồ mức độ lưu hành sốt rét *Plasmodium falciparum* toàn cầu cho năm 2007.

**Phương pháp luận và các kết quả**

Tổng cộng 8.938 cuộc điều tra tỷ lệ ký sinh trùng *P.falciparum* (*Pf*PR) đã được thu thập bằng nhiều phương thức tìm kiếm chi tiết. Trong số này, 7.953 điều tra thỏa mãn các yêu cầu tiêu chuẩn chặt chẽ để đưa vào cơ sở dữ liệu *Pf*PR toàn cầu, được chuẩn hóa tuổi trong khoảng 2-10 năm tuổi để dùng trong công tác lập bản đồ mức độ lưu hành. Quy trình thống kê địa lý dựa trên mô hình được dùng để tạo ra bề mặt liên tục thể hiện tỷ lệ lưu hành của sốt rét trong vùng giới hạn không gian có sự lan truyền *P. falciparum* ổn định đã được xác định trước đây. Những quy trình này được thực thi trong môi trường thống kê kiểu Bayes, nhờ đó mức độ không chắc chắn của các dự báo có thể được đánh giá một cách chặt chẽ. Điều này được biểu diễn thành xác suất dự báo đúng của một trong ba cấp lưu hành; đã được phân loại trước đây dùng làm gợi ý thông tin cho công tác kiểm soát sốt rét. Các ước lượng dân số trong vùng có nguy cơ mắc bệnh, được hiệu chỉnh theo các tác động làm thay đổi mức độ truyền bệnh của quá trình đô thị hóa ở châu Phi, được tính ra sau khi có tham chiếu với các bề mặt dân số năm 2007.

Trong tổng số 1,38 tỉ người thuộc vùng có nguy cơ sốr rét *P. falciparum* ổn định, 0,69 tỉ người thuộc khu vực Trung, Đông và Nam Á (TDNA), 0,66 tỉ người thuộc châu Phi, Yemen và Arập Xê-út (châu Phi+) và 0,04 tỉ ở các nước châu Mỹ. Tất cả những người nằm trong vùng nguy cơ ổn định ở các nước châu Mỹ đều thuộc nhóm tỷ lệ lưu hành thấp nhất (*Pf*PR_2-10_ ≤5%). Một phần rất lớn (88%) những người sống trong vùng có nguy cơ ổn định ở TDNA cũng thuộc nhóm lưu hành thấp này; một phần nhỏ còn lại (11%) ở trong vùng thuộc nhóm lưu hành trung bình (*Pf*PR_2-10_ từ >5 đến <40%) và phần còn lại (1%) ở trong vùng lưu hành cao (*Pf*PR_2-10_ ≥40%). Mức độ lưu hành cao phân bố rộng rãi ở khu vực châu Phi+, nơi có đến 0,35 tỉ người sống trong vùng có nguy cơ cao này. Hầu hết phần còn lại ở trong vùng có nguy cơ trung bình (0,20 tỉ), với một phần nhỏ hơn (0,11 tỉ) ở trong vùng có nguy cơ thấp.

**Các kết luận và ý nghĩa**

Mức độ lưu hành cao của sốt rét *P.falciparum* phổ biến ở châu Phi. Mức độ lưu hành thấp khá đồng nhất được ghi nhận ở các vùng thuộc châu Mỹ. Mức độ lưu hành thấp cũng phân bố rộng rãi ở khu vực TDNA, nhưng có những khu vực nhỏ với mức độ nguy cơ trung bình và rất hiếm thấy là những khu vực có nguy cơ truyền bệnh cao. Do vậy đây là cơ sở quan trọng cho việc kiểm soát sốt rét ở châu Phi và loại bỏ sốt rét ở những vùng khác. Bản đồ toàn cầu về mức độ lưu hành của sốt rét *P.falciparum* này là kết quả đầu tiên của một chuỗi các bản đồ khác mà nhờ đó có thể giám sát và đánh giá được tiến độ của công tác phòng chống sốt rét.

**Giới thiệu**

Bản đồ rất cần thiết trong mọi phương diện của công tác điều phối hoạt động kiểm soát sốt rét [1]. Trong bối cảnh chính sách quốc tế khi mà cộng đồng phòng chống sốt rét đang bị thử thách bởi việc phải xem xét lại tính hợp lý của việc loại bỏ sốt rét [2-4], công tác lập bản đồ sốt rét sẽ trở thành một công cụ ngày càng quan trọng cho việc lập kế hoạch, thực thi và đánh giá tác động của công tác phòng chống sốt rét trên toàn thế giới. Bản đồ toàn cầu gần đây nhất của về mức độ lưu hành của *Plasmodium falciparum* được công bố năm 1968 [5]. Một đặc điểm chung giống với tất cả các bản đồ trước đó về sự phân bố trên toàn cầu của sốt rét , và giống với phần lớn các bản đồ sau đó , là bản đồ này (i) có dữ liệu đầu vào không được mô tả chi tiết; (ii) đường cong “nguy cơ” xác định sử dụng các quy tắc dựa trên ý kiến chuyên gia một cách chủ quan và được diễn giải kém và (iii) không cung cấp định lượng mức độ không chắc chắn của các dự báo. Ở đây chúng tôi mô tả việc thành lập một bản đồ mới về mức độ lưu hành sốt rét toàn cầu khắc phục những nhược điểm chính này.

Phạm vi địa lý của việc mô hình hóa

Giới hạn không gian trên toàn cầu của sự lan truyền sốt rét *P.falciparum* vừa được lập bản đồ bằng cách dùng ba nguồn thông tin là dữ liệu về tỷ lệ mắc bệnh đã được báo cáo ở cấp quốc gia, các nguồn thông tin y học khác và các quy tắc sinh học về việc loại bỏ sự lan truyền bệnh, xuất xứ từ giới hạn về nhiệt độ và độ khô hạn đối với sinh thái học của các vectơ *Anopheles* chính [17,18]. Kết quả của nó là phân chia thế giới thành ba nhóm: biểu thị về mặt không gian vùng không có nguy cơ, nguy cơ không ổn định (Chỉ số mắc bệnh sốt rét *Plasmodium falciparum* hàng năm (PfAPI) < 0.1 trên 1.000 người trên năm) và nguy cơ ổn định (*Pf*API ≥0.1 trên 1.000 người trên năm) về sự lan truyền *Plasmodium falciparum* cho năm 2007. Những nhóm này được trình bày trên Hình 1. Việc phân chia mức ổn định-không ổn định của *Pf*API dựa vào các lý do về thống kê, hậu cần, chương trình và thực tiễn, những yếu tố là cơ sở của các cấp *Pf*API dùng để xác định các điểm hành động trong chương trình loại trừ sốt rét toàn cầu [19-21].

Công tác lập bản đồ được mô tả trong tài liệu này mở rộng hoạt động nói trên một cách đáng kể. Việc tổng hợp các điều tra sốt rét ở mức toàn cầu lớn nhất từ trước đến này đã được dùng để tiên đoán các giá trị về tỷ lệ mắc sốt rét *P. falciparum* tại tất cả các điểm nằm trong giới hạn không gian có sự lan truyền ổn định để tạo ra một bề mặt liên tục về mức độ lưu hành của *P. falciparum*. Để hỗ trợ cho công tác này, các giới hạn không gian đòi hỏi việc thu mẫu lại ở độ phân giải 5x5km bằng ArcView 3.2 (ESRI, 1999) do việc sử dụng các kỹ thuật lập bản đồ nặng nề trên máy tính, sẽ được mô tả trong phần tiếp theo, không thể chạy được ở độ phân giản không gian 1x1km trên mức toàn cầu.

Các phương pháp tiếp cận gần đây trong việc lập bản đồ mức độ lưu hành sốt rét

Nhiều phương pháp tiếp cận khác nhau hiện có để tạo ra các bản đồ mức độ lưu hành liên tục bằng cách dùng số liệu điều tra sốt rét, tất cả các phương pháp đó đều đòi hỏi việc sử dụng một mô hình để tiên đoán các giá trị về mức độ lưu hành tại những vị trí không có số liệu điều tra [22-26]. Những bản đồ tạo thành từ các mô hình này có tính chất không chắc chắn cố hữu và việc định lượng của nó là mối quan ngại chủ yếu trong lập bản đồ bệnh.

Một số nghiên cứu gần đây đã chấp nhận việc dùng một môi trường tiên đoán được gọi là địa lý thống kê dựa trên mô hình (MBG) [27] dùng để dự báo về mặt không gian mức độ lưu hành của sốt rét [28-33] và tỷ lệ nhiễm của các loại bệnh có nguồn gốc từ vectơ và ký chủ trung gian khác [34-38]. MBG cung cấp một cách diễn giải thống kê chính quy các công cụ địa lý thống kê cổ điển dùng trong tiên đoán không gian và cho phép đưa vào các phương pháp kiểu Bayes dùng trong suy luận thống kê [42,43]. Ưu điểm chính của MBG trong việc lập bản đồ bệnh là xử lý chặt chẽ mức độ không chắc chắn sinh ra trong các giai đoạn khác nhau của tiến trình mô hình hóa [27]. Bằng cách mô hình hóa sự tương tác giữa mức độ không chắc chắn từ các nguồn khác nhau, phân bố xác suất được sinh ra cho mỗi vị trí tiên đoán, có thể được tóm tắt lại để cho ra các số đo xác thực về độ tin cậy của các giá trị tiên đoán. Do vậy bản đồ tạo thành sẽ cung cấp một thước đo hiện tại về mức độ lưu hành sốt rét trên toàn cầu, dùng các kỹ thuật MBG để đánh giá độ tin cậy trong các tiên đoán, và cung cấp cho người dùng bản đồ ước lượng rõ ràng về tính xác thực của các tiên đoán [36].

Một nguyên tắc nền tảng của địa thống kê là dự báo được lập bản đồ gia tăng mức độ không chắc chắn khi mật độ và mức độ lân cận của các điểm dữ liệu cạnh nhau giảm xuống. Khi dữ liệu được thu thập ở các thời điểm khác nhau, cũng như ở các địa điểm khác nhau, nguyên tắc này được áp dụng cho thời gian cũng như không gian. Ví dụ về các nghiên cứu dịch tễ mở rộng địa thống kê không gian cho cả thời gian không nhiều [44-47], nhưng trong nghiên cứu này một hệ thống mô hình hóa địa thống kê hoàn chỉnh theo không gian và thời gian đã được phát triển. Việc đưa vào thời gian cho phép so sánh rõ ràng thước đo này với các lần chạy lặp lại bản đồ trong tương lai. Do vậy bản đồ sẽ cung cấp cho ta một hệ thống địa lý rõ rệt để giám sát và đánh giá tác động của các cơ quan phòng chống sốt rét *P. falciparum* trên toàn thế giới.

**Phương pháp**

Tổng quan phân tích

Mục tiêu của các phân tích này là dùng một cơ sở dữ liệu hiện có về các điều tra tỷ lệ mắc ký sinh trùng *Plasmodium falciparum* (*Pf*PR) để tạo một bề mặt liên tục, toàn cầu về mức độ lưu hành của sốt rét *P. falciparum* cho năm 2007, được thực hiện bằng các phương pháp rõ ràng và có thể lặp lại được và ghi nhận chi tiết mức độ không chắc chắn liên quan đến các dự báo của phương pháp này.

Các bước quan trọng được dùng để xác định bản đồ toàn cầu liên tục về tỷ lệ nhiễm *P. falciparum* trong hệ thống phân tích của chúng tôi được khái quát trong Hình 2. Trước tiên, cần phải tìm kiếm và xử lý trước dữ liệu *Pf*PR để tạo ra một bộ dữ liệu được xác định tọa độ, phân bố rộng về mặt địa lý của các điều tra sốt rét dùng cho việc lập bản đồ và khảo sát các đồng biến số môi trường (Nghi thức S1) và tác động của các khuôn mẫu phân bố của dân cư [48-50] (Nghi thức S2). Thứ hai, cơ sở dữ liệu *Pf*PR đã tinh chỉnh được dùng để tạo ra bề mặt tỷ lệ nhiểm sốt rét liên tục, chuẩn hóa theo tuổi và được hiệu chỉnh theo đô thị bằng các phương pháp MBG trong hệ thống thống kê kiểu Bayes (Nghi thức S3). Thứ ba, các quy trình chi tiết kiểm tra tính xác thực được thực hiện để đánh giá độ chính xác của các dự báo về mức độ lưu hành và các số đo về độ không chắc chắn (Nghi thức S4). Sau cùng, các ước lượng về dân số trong vùng có nguy cơ (PAR) bị sốt rét *P. falciparum* được trích ra ở mức toàn cầu và được biểu diễn ở mức khu vực, phân chia theo nhóm tuổi.

Thu thập cơ sở dữ liệu toàn cầu về tỷ lệ nhiễm *P. falciparum*

Trong tất cả các số đo có thể sử dụng để đo mức độ lưu hành sốt rét, tỷ lệ nhiễm ký sinh trùng (tỷ lệ số người được lấy mẫu có ký sinh trùng phát hiện được trong máu) được chọn dùng để lập bản đồ, do tính phổ biến ở mức toàn cầu của nó [18] và độ nhạy của nó trên toàn vùng có lan truyền sốt rét *P. falciparum* [19]. Việc phân loại mức độ lưu hành sốt rét đã được đề xuất dùng nhóm tuổi có ý nghĩa về mặt dịch tễ học 2 (2,00) đến 10 (9,99) [51], do ảnh hưởng của khả năng kiểm soát mức độ lưu hành sốt rét bằng phương pháp phòng chống sử dụng rộng rãi nhất hiện nay – màn tẩm (ITN) – lên nhóm tuổi này [19]. Lớp *Pf*PR thấp nhất trong nhóm tuổi từ 2 đến 10 (từ đây được gọi là *Pf*PR_2-10_), tương ứng với ≤5%. Đây là điểm mà với giá trị thấp hơn thì các điều tra *Pf*PR đòi hỏi phải có kích thước mẫu của số người điều tra khó có thể đạt được để đo được chính xác mức độ lưu hành và do vậy các số đo về sốt rét dựa trên cơ sở điều tra thường được dùng [52–54]. Chúng tôi coi mức độ lan truyền ổn định, ở mức trung bình được biểu thị bởi *Pf*PR_2-10_ từ >5% đến <40%, vì phạm vi mà các mô hình toán học tiên đoán rằng việc cắt đứt lan truyền có thể đạt được với mức độ dùng ITN trên toàn khu vực khi *Pf*PR_2-10_ <40% . Bất kể một số hạn chế liên quan đến tập tính và sinh thái của các vector *Anopheles* địa phương [56], mức *Pf*PR_2-10_ <40% được coi là mốc đo an toàn, vì việc dùng ITN ít khi nào tách biệt với các biện pháp phòng chống khác giúp giảm mức độ lan truyền hơn nữa. Các vùng có mức độ lan truyền cao và ổn định, nơi mà các biện pháp phòng chống tổng hợp cần được nghiên cứu áp dụng nếu muốn cắt đứt được sự lan truyền, được nhận diện khi tỷ lệ nhiễm ở trên mức này: *Pf*PR_2-10_ ≥40%. Phân loại sốt rét này được dùng để hướng dẫn cho việc diễn giải bề mặt mức độ lưu hành dự báo trong suốt quá trình phân tích và khác với các thước đo mức độ lưu hành truyền thống [57] đã được chứng minh là không đánh giá một cách có ý nghĩa khi xem xét đến khả năng kiểm soát và loại bỏ được sốt rét trong hầu hết các mô hình [19,55].

Tiến trình nhận diện, thu thập và định vị các điều tra cộng đồng về tỷ lệ nhiễm ký sinh trùng từ năm 1985 đã được mô tả [58]. Việc tìm kiếm dữ liệu *Pf*PR là hoạt động đang diễn ra trong khuôn khổ Dự án Atlas Sốt rét (MAP, http://www.map.ox.ac.uk) và được hoàn tất vào ngày 31 tháng Bảy năm 2008 cho lần chạy năm 2007 của bản đồ mức độ lưu hành sốt rét toàn cầu (Nghi thức S1). Tổng cộng 8.938 điều tra cắt ngang ước lượng *Pf*PR đã được tập hợp từ 78 trong số 87 quốc gia có lưu hành sốt rét *P. falciparum* (*Pf*MECs) . Những quốc gia không có thông tin trong cơ sở dữ liệu là Bangladesh, Belize, Bhutan, Djibouti, Cộng hòa Dominic, Guyana, Iran, Kyrgyzstan và Panama.

Sau sáu cấp độ lọc bỏ dữ liệu (loại bỏ các điều tra giới hạn trong vùng lớn (> 100 km^2^) và nhỏ (> 25 km^2^) [58]; loại bỏ các điều tra không thể định vị được tọa độ hoặc định vị không chính xác; loại bỏ những điều tra không thể tách biệt được về mặt thời gian thành từng điều tra riêng hoặc những điều tra không biết thời gian), còn lại 7.991 điều tra trong cơ sở dữ liệu (Hình S1.2 trong Nghi thức S1).

Sau đó dữ liệu *Pf*PR được chuẩn hóa theo tuổi thành nhóm tuổi 2-10 trước khi lập bản đồ bằng cách dùng một thuật toán dựa trên các mô hình chuyển đổi xúc tác được điều chỉnh cho sốt rét của Pull và Grab [59]. Thuật toán này được thấy ra phù hợp nhất trong số các quy trình chuẩn hóa và được mô tả chi tiết trong tài liệu khác [51] (Nghi thức S1.3).

Bộ dữ liệu sau cùng được phân chia ra thành ba vùng chính trên toàn cầu (Hình 1): châu Mỹ; châu Phi, Yemen và Ả-rập Xê-út (châu Phi+); và khu vực Trung, Đông và Nam Á (TDNA) (Nghi thức S1.4). Việc phân chia này cho phép các khu vực tách biệt về mặt địa sinh học, côn trùng học và dịch tễ học này được xử lý một cách riêng biệt, trong khi vẫn có đủ dữ liệu trong từng vùng để thực hiện được các phân tích có ý nghĩa. Sự phân chia ở mức toàn cầu này được hỗ trợ bằng cách quan sát cấu trúc không gian tách biệt của dữ liệu *Pf*PR_2-10_ trong từng vùng, minh họa bởi các biến semi-variogram của chúng (Hình S1.1 trong Nghi thức S1).

Các phương pháp tiếp cận đặc thù với sự lan truyền sốt rét dùng để lập bản đồ các khu vực đô thị, gần đô thị và nông thôn đã được phát triển, ý nghĩa của chúng được mô tả chi tiết trong một tài liệu khác [50] (Nghi thức S2). Tóm tắt là tất cả các vùng đô thị (các vùng UE) được xác định theo Dự án lập bản đồ Nông thôn Đô thị Toàn cầu (GRUMP), mặt nạ UE phiên bản alpha (GRUMP UE) [60,61] được xác định với độ phân giải không gian là 1x1 km (Nghi thức S2.1) [50]. Trong các phạm vi này, những vùng nào có mật độ dân số lớn hơn 1000 người/km^2^ dựa theo bản đồ dân số thế giới phân lưới phiên bản 3 (GPW3), bề mặt mật độ dân số [60,61] được lập bản đồ [48]. Tất cả các điều tra được gán giá trị đô thị (Dân số Thế giới dạng Phân lưới phiên bản 3 ≥1000 km^2^ ở trong GRUMP UE), gần đô thị (Dân số Thế giới dạng Phân lưới phiên bản 3 <1000 km^2^ ở trong GRUMP UE) hoặc nông thôn (ngoài GRUMP UE) (Nghi thức S2.2).

Sau đó các giá trị ngoại biên về mặt thống kê trong dữ liệu *Pf*PR_2-10_ được nhận diện bằng một bộ lọc địa lý thống kê (Nghi thức S1.5). Tiến trình này dùng thống kê semi-variogram để đánh giá xem một điểm có khác biệt quan trọng với các điểm lân cận hay không dựa trên khoảng cách và sự biến động về mặt không gian trong vùng. Quy trình này đã nhận diện được 38 bản ghi không phải là đô thị của *Pf*PR_2-10_, được loại bỏ khỏi bộ dữ liệu trước khi thực hiện mô hình hóa tiếp theo. Chi tiết của các điều tra này được cung cấp nếu có yêu cầu.

Bộ dữ liệu *Pf*PR_2-10_ (n=7.953) sau cùng được trình bày trên Hình 1. Tính chất của cơ sở dữ liệu *Pf*PR_2-10_ sau cùng này được mô tả (Bảng S1.2 trong Nghi thức S1) cùng với một đồ thị về giá trị giữa của *Pf*PR_2-10_ theo năm ghi nhận dữ liệu (Hình S1.3 trpmg Nghi thức S1), cho thấy thời gian là nguồn biến động quan trọng được đưa vào mô hình MBG. Các nghiên cứu sơ bộ tương tự về mối quan hệ của những dữ liệu này với giới hạn về khí hậu [62] và các đồng biến số môi trường từ ảnh viễn thám [63] không cho thấy mối quan hệ chặt chẽ (Hình S1.5 và S1.6 trong Nghi thức S1), củng cố cho phương pháp chủ đạo một biến của các phân tích này.

Có một quan niệm không đúng cho rằng các điều tra sốt rét chỉ được thực hiện ở các vùng có tỷ lệ nhiễm cao. Thực ra, sự gia tăng về xu hướng thực hiện các điều tra cấp quốc gia có tính chất đại diện cho tất cả các vùng trong một quốc gia, và việc xác nhận sự vắng mặt của lan truyền *P. falciparum* khi thu mẫu *P. vivax* đã dẫn đến nhiều giá trị tỷ lệ nhiễm là không ghi nhận được trong các điều tra. Tổng cộng có 119 trong số 261 ghi nhận giá trị không ở châu Mỹ, 1010 trong số 5307 ghi nhận giá trị không ở châu Phi+ và 775 trong số 2385 ghi nhận giá trị không trong khu vực TDNA (Hình 1).

Chi tiết về thống kê

Các thuật toán về địa lý thống kê tạo ra các bản đồ liên tục bằng cách tiên đoán các giá trị tại các vị trí không thu mẫu bằng các phương pháp tổng hợp tuyến tính từ dữ liệu mẫu có được. Đối với việc lập bản đồ được mô tả trong nghiên cứu này, rõ ràng là độ tin cậy đi kèm với một giá trị tiên đoán *Pf*PR_2-10_ tại một vị trí không thu mẫu sẽ chịu ảnh hưởng của (i) phân bố của các điểm điều tra quanh vị trí đó (mật độ không gian của dữ liệu huấn luyện), (ii) phạm vi mà giá trị *Pf*PR_2-10_ thay đổi một cách trơn tru trong không gian (tính không đồng nhất về mặt không gian của dữ liệu huấn luyện), và (iii) số người được lấy mẫu trong từng khảo sát (độ chính xác của các điều tra thành phần trong dữ liệu huấn luyện). Phương pháp MBG [27] được áp dụng trong hệ thống thống kê kiểu Bayes để đưa các yếu tố này vào khi tạo ra các bản đồ liên tục về *Pf*PR_2-10_ (Nghi thức S3). Vì dữ liệu được thu thập tại các thời điểm khác nhau trong giai đoạn nghiên cứu 1985-2008, điều quan trọng là cần mở rộng phương pháp địa lý thống kê chỉ theo không gian ra một hệ thống theo không gian-thời gian để xem xét đồng thời mật độ và đồ không đồng nhất của dữ liệu theo cả thời gian lẫn không gian. Thuật toán chuẩn hóa tuổi được đưa vào thành một mô hình phụ trong hệ thống này để cho phép ước lượng các sai số vốn có trong tiến trình này và đưa chúng vào giai đoạn MBG (Nghi thức S3).

Đối với mỗi vùng, một mô hình địa thống kê kiểu Bayes được xây dựng, trong đó có giá trị *Pf*PR_2-10_ cho năm 2007, , tại mỗi vị trí được mô hình hóa như là sự chuyển đổi của một trường được cấu trúc theo không-thời gian chồng lên biến động không theo cấu trúc (ngẫu nhiên) . Số lần dương tính với *P. falciparum* từ một mẫu tổng tại mỗi vị trí khảo sát được mô hình hóa như là một biến nhị thức độc lập có điều kiện theo giá trị *Pf*PR_2-10_ đã chuẩn hóa theo tuổi tại điểm không quan sát được [36]. Phần không-thời gian được biểu thị bằng phương pháp Gauss tĩnh với trị trung bình và hiệp phương sai được xác định theo phiên bản không đẳng hướng về mặt không gian của hàm hiệp phương sai không-thời gian do Stein [64] đề nghị. Hàm hiệp phương sai Stein được điều chỉnh để cho phép mô hình cận biên thời gian đưa vào được thành phần chu kỳ có bước sóng 12 tháng, cho phép có khả năng mô hình hóa các tác động theo mùa trong cấu trúc hiệp phương sai theo thời gian quan sát được. Các tác động này phát sinh khi các nghiên cứu được thực hiện ở các năm khác nhau nhưng trong các tháng như nhau trong năm có xu hướng giống nhau hơn so với khi không có tính chất theo mùa. Thành phần trung bình được mô hình hóa như một hàm tuyến tính của thời gian, , và theo vị trí tiên đoán là đô thị, hay gần đô thị (được xác định tương ứng bởi các biến chỉ định và ) chứ không phải nông thôn: . Mỗi điều tra được tham chiếu về mặt không gian bằng cách dùng điểm giữa (tính theo năm thập phân) giữa tháng bắt đầu và kết thúc ghi nhận số liệu. Trạng thái đô thị, gần đô thị, hay nông thôn được gán cho mỗi vị trí tiên đoán bằng cách dùng bề mặt phạm vi đô thị GRUMP có chỉnh sửa đã mô tả ở trên (Nghi thức S2.2), lấy mẫu lại ở độ phân giải lưới 5x5 km. Thành phần không cấu trúc được biểu diễn ở dạng Gauss có trị trung bình là không và phương sai . Phép suy luận kiểu Bayes được áp dụng bằng cách dùng phương pháp Markov Chain Monte Carlo (MCMC) để tạo ra các mẫu từ các phân bố sau của: trường Gauss tại mỗi vị trí dữ liệu; các tham số không quan sát được , *,* , và như đã trình bày ở trên và các tham số không quan sát được khác xác định cấu trúc và tính không đẳng hướng của hàm hiệp phương sai không-thời gian dạng mũ (Nghi thức S3.4). Khoảng cách giữa các vị trí được tính toán theo khoảng cách vòng tròn lớn để đưa vào tác động của mặt cong trái đất, yếu tố trở nên quan trọng ở tỷ lệ cấp vùng. Các mẫu được tạo ra từ trị trung bình năm 2007 của phân bố sau tại mỗi vị trí tiên đoán. Đối với mỗi mẫu của hậu nghiệm kết hợp, các tiên đoán được thực hiện bằng cách dùng phép giả lập có điều kiện theo không-thời gian trong 12 tháng của năm 2007 . Những tiên đoán này được thực hiện tại các điểm trên lưới không gian 5x5 km thông thường trong giới hạn không gian có sự lan truyền ổn định của P. falciparum. Do vậy đầu ra của mô hình bao gồm các mẫu từ phân bố sau được tiên đoán của giá trị *Pf*PR_2-10_ trung bình của năm 2007 tại mỗi vị trí trên lưới, sau đó nó được dùng để tạo ra các ước lượng điểm (được tính là trị trung bình của mỗi tập hợp mẫu hậu nghiệm), các xác suất thuộc nhóm lưu hành nào và các ước lượng phương sai chuẩn (Nghi thức S3.4). Mô tả chi tiết hơn về phương pháp các đầu ra địa lý thống kê được dùng để tạo các bản đồ khác nhau như thế nào được cung cấp (Nghi thức S3.5).

Kiểm chứng mô hình

Một đánh giá tính xác thực của bề mặt được lập bản đồ là điều cần thiết và một vài phương pháp mô tả đặc biệt được áp dụng (Nghi thức S4). Khả năng của mô hình trong tiên đoán giá trị điểm của *Pf*PR_2-10_ và nhóm mức độ lưu hành có khả năng cao nhất được kiểm tra bằng cách dùng quy trình “lấy ra”. Một tập hợp kiểm chứng được tạo ra bằng cách chọn *theo* cách thu mẫu ngẫu nhiên phân cấp khử nhóm về mặt không gian mười phần trăm dữ liệu (n=800), sau đó tập hợp này được loại bỏ khỏi bộ dữ liệu (Nghi thức S4.1). Tiếp theo mô hình được chạy đầy đủ với 7.153 điểm dữ liệu còn lại để tạo ra các phân bố hậu nghiệm tiên đoán của *Pf*PR_2-10_ để so sánh với các giá trị đã biết tại các vị trí của dữ liệu 800 điểm lấy ra. Khác với việc chạy mô hình chính, trong đó giá trị trung bình được tiên đoán cho năm 2007, giá trị *Pf*PR_2-10_ tiên đoán từ chạy kiểm chứng cho tháng tương ứng với điểm giữa của mỗi điều tra bị lấy ra, để cung cấp các giá trị so sánh tạm thời. Với bộ dữ liệu có kích thước lớn này, một tập hợp kiểm chứng được coi là đủ để tạo ra thống kê kiểm chứng với cấp độ chính xác cần có.

Khả năng tiên đoán các giá trị đã biết của *Pf*PR_2-10_ được tóm tắt bằng cách dùng sai số trung bình làm số đo độ lệch tổng thể, sai số tuyệt đối trung bình làm độ chính xác tổng thể và hệ số tương quan làm số đo mức độ liên hệ tuyến tính [44,66]. Các phép thống kê này được trình bày ở cả dạng giá trị tuyệt đối lẫn dạng tỷ lệ của trị trung bình *Pf*PR_2-10_ trong mỗi vùng khi được tính ra từ tập hợp kiểm chứng. Khả năng tiên đoán nhóm mức độ lưu hành được kiểm tra bằng cách dùng thống kê diện-tích-dưới-đường-biểu-diễn (AUC) dẫn xuất từ đường cong đặc trưng hoạt động của bộ thu (ROC), là đường cong vẽ đồ thị độ nhạy theo 1 – mức độ đặc thù của mỗi nhóm mức độ lưu hành [34,67]. Các giá trị AUC trên 0,9 cho thấy sự phù hợp rất cao giữa giá trị thực và nhóm lớp được tiên đoán, các giá trị trên 0,7 cho thấy sự phù hợp tương đối tốt và các giá trị ở mức 0,5 cho thấy mô hình chạy không tốt hơn sự phân phối ngẫu nhiên thành phần của lớp [34,67]. Một quy trình [44,68] cũng được thực hiện để kiểm tra phạm vi các phân bố sau tiên đoán tại mỗi vị trí tiên đoán cung cấp một số đo hợp lý về độ không chắc chắn đến mức nào. Quy trình này cho phép xác suất được gán vào các giá trị *Pf*PR_2-10_ tiên đoán tại mỗi vị trí tiên đoán được so sánh với các xác suất tương ứng quan sát được trong từng vùng. Chi tiết hơn về quy trình này cũng được cung cấp (Nghi thức S4.2).

Các phân bố tần số của *Pf*PR_2-10_ được hiển thị cho cả dữ liệu đầu vào và bề mặt tiên đoán đầu ra bằng cách dùng các đồ thị violin [69]. Các đồ thị này trình bày phép xấp xỉ đã làm trơn của phân bố tần số (một đồ thị mật độ nhân) của *Pf*PR_2-10_ cho từng vùng chồng lên một thanh trung tâm thể hiện các giá trị giữa và giá trị khoảng tứ phân vị. Các đồ thị riêng biệt được tính toán bằng cách dùng dữ liệu *Pf*PR_2-10_ đã chuẩn hóa theo tuổi từ tất cả các năm trong cơ sở dữ liệu và chỉ từ năm 2007, và một đồ thị bổ sung được tính toán bằng cách dùng các ước lượng điểm cho mỗi vị trí trên bề mặt *Pf*PR_2-10_ đầu ra được tiên đoán của năm 2007.

Ước lượng mật độ dân số năm 2007

Phiên bản GRUMP alpha cung cấp các tính toán dân số dạng phân lưới và ước lượng mật độ dân số ở độ phân giải không gian của các năm 1990, 1995 và 2000, cả dạng đã điều chỉnh lẫn không điều chỉnh theo ước lượng dân số quốc gia của Liên hiệp quốc [60,61] (Nghi thức S2.3). Các tính toán dân số đã điều chỉnh cho năm 2000 được quy chiếu đến năm 2007 bằng cách áp dụng tốc độ tăng dân ở mức quốc gia, phương sai trung bình, mức tăng giữa các đợt điều tra theo từng quốc gia [70] theo các phương pháp đã mô tả trước đây [71] (Nghi thức S2.4). Các tính toán dân số này sau đó được phân chia ở mức quốc gia theo nhóm tuổi dùng cấu trúc tuổi dân số theo định nghĩa của Liên hiệp Quốc [72] dùng cho năm 2005 để thu được các bề mặt tính toán dân số ở mức dưới 0-5 tuổi, 5-14 tuổi và từ 15 tuổi trở lên.

Ranh giới số của 87 quốc gia có lưu hành *P. falciparum* được chồng lên bề mặt nhóm mức độ lưu hành đã được điều chỉnh về thuộc tính đô thị (được quy chiếu theo phép chiếu đồng diện tích) và các khu vực của mỗi cấp lưu hành được trích ra bằng cách dùng ArcView GIS 3.2 (ESRI, 1999) (Nghi thức S2.4). Các lớp này cũng được chồng lên dữ liệu GRUMP [60,61] để trích xuất các ước lượng vùng đô thị có điều chỉnh của dân số trong vùng có nguy cơ *P. falciparum* theo mức độ lưu hành và theo nhóm tuổi (Nghi thức S2.4). Sau cùng các bề mặt này được phối hợp với các bản đồ về mức độ không chắc chắn để cung cấp một chỉ mục về độ không chắc chắn lấy trọng số theo dân số (tạo logarit của mật độ dân số và nghịch đảo của xác suất gán đúng nhóm).

**Kết quả**

Độ chính xác của bản đồ mức độ lưu hành sốt rét *P. falciparum* tiên đoán

Bề mặt tiên đoán liên tục về mức độ lưu hành sốt rét *P. falciparum* được trình bày trên Hình 3. Việc kiểm soát nhóm mức độ lưu hành liên quan mà thành viên là có xác suất cao nhất được trình bày trên hình 4. Xác suất thực của việc tiên đoán đúng từng nhóm được trình bày trong Hình 5A. Mô tả chi tiết biến động theo vùng của khu vực tại các cấp nguy cơ ổn định khác nhau này và PAR liên quan đi kèm với mô tả về độ chính xác của các tiên đoán. Các số đo khác về độ không chắc chắn của các tiên đoán được cung cấp trong phần này (Nghi thức S4.3).

Tiên đoán các giá trị điểm *Pf*PR_2-10_

Việc khảo sát sai số trung bình khi tạo ra bề mặt mức độ lưu hành sốt rét *P. falciparum* (hình 3) cho thấy độ lệch tổng tối thiểu trong các giá trị *Pf*PR_2-10_ tiên đoán được với giá trị ở mức toàn cầu là 0,91 cho thấy xu hướng ước lượng quá mức *Pf*PR_2-10_ là dưới 1 phần trăm (châu Mỹ = 0.63, châu Phi+ = 0.80, TDNA = 1.18) (Bảng 1). Xem xét sai số tuyệt đối trung bình cho thấy độ lớn trung bình của sai số trong các tiên đoán *Pf*PR_2-10_ là 9,75 (châu Mỹ = 3,52, châu Phi+ = 11.=,02, TDNA = 7,71) (Bảng 1). Hệ số tương quan toàn cầu giữa giá trị thực và giá trị tiên đoán là 0,82, cho thấy sự phù hợp tuyến tính rất tốt ở mức toàn cầu và điều này được minh họa rõ hơn bởi biểu đồ phân tán (Hình 6, phía trên trái và Bảng 1). Các tương quan ở cấp khu vực đối với các nước châu Mỹ và TDNA nhìn chung là yếu hơn (các nước châu Mỹ = 0,03, châu Phi + = 0,82, TDNA = 0,70) (Bảng 1). Một biểu đồ semi-variogram các phần dư của mô hình chuẩn hóa (Hình 6B) cho thấy bằng chứng về tự tương quan không gian vào, lên đến độ trễ khoảng hai độ thập phân, mặc dù phép so sánh với “phong bì rỗng” giả lập cho thấy điều này không có ý nghĩa về mặt thống kê (Nghi thức S4.2).

Tiên đoán nhóm mức độ lưu hành

Các đường cong ROC và thống kê AUC cho mỗi nhóm lưu hành được trình bày (Hình 6, dưới cùng bên trái và Bảng 2). Các giá trị AUC toàn cầu cho ba nhóm mức độ lưu hành vượt quá ngưỡng 0,7 cho mức phân biệt từ khá đến tốt, trong khi các giá trị cho cả nhóm *Pf*PR_2-10_ ≤5% và *Pf*PR_2-10_ ≥40% vượt quá ngưỡng 0,9 cho mức phân biệt rất tốt. Tổng thể là 70,8% các điểm được phân loại đúng (các nước châu Mỹ = 80,0%, châu Phi+ = 70,6%, TDNA= 69,9%) và quan trọng là chỉ có 1,1% các điểm bị phân loại sai sang nhóm không kề cận (các nước châu Mỹ = 0,0%, châu Phi+ = 0,6%, TDNA = 2,5%) (Bảng 2). Một bảng tiếp liên đầy đủ cho từng nhóm được cung cấp (Nghi thức S4.3).

Cung cấp các số đo thực tiễn về mức độ không chắc chắn cho từng tiên đoán

Đồ thị xác suất-xác suất so sánh các ngưỡng xác suất tiên đoán được với các xác suất vùng bao trùm quan sát được (Hình 6D) cho thấy tương quan gần gũi giữa hai giá trị này, thể hiện việc mô hình này cung cấp sự biểu diễn tương đối đáng tin cậy về mức độ không chắc chắc của các tiên đoán điểm. Tuy nhiên đường đồ thị được vẽ hơi lệch lên trên đường 1:1 chạy qua hầu hết các giá trị ngưỡng, chủ yếu là với các ngưỡng xác suất trong khoảng 0,00 và quanh giá trị 0,25. Điều này có nghĩa là ngưỡng xác suất tiên đoán được của, ví dụ như 0,1, có thể liên quan đến “ngưỡng xác suất thực” ở vào khoảng 0,2. Nói cách khác, mô hình này có xu hướng ước lượng thấp xác suất của *Pf*PR_2-10_ lấy giá trị dưới (Hình S4.1A trong Nghi thức S4). Đổi lại, xu hướng này dẫn đến việc ước lượng quá *Pf*PR_2-10_ ở một số khu vực có mức độ lưu hành thấp.

Bản đồ mức độ lưu hành sốt rét *P. falciparum* toàn cầu

Trong năm 2007, diện tích có nguy cơ sốt rét *P. falciparum* ổn định trên toàn cầu là 29,73 triệu km^2^, phân bố ở các nước châu Mỹ (6,03 triệu km^2^, 20,30%), châu Phi + (18,17 triệu km^2^, 61,10%), và TDNA (5,53 triệu km^2^, 18,60%) (Bảng 3). Trước đây chúng tôi đã ước lượng rằng trên toàn cầu có 2,37 tỷ người ở trong vùng có nguy cơ lan truyền *P. falciparum* và có 0,98 tỷ người ở trong vùng có nguy cơ không ổ định [17,18]. Số người thuộc vùng nguy cơ ổn định, 1,383 tỷ, phân bố ở các nước châu Mỹ (0,041 tỷ, 2,94%), châu Phi+ (0,657 tỷ, 47,48%) và TDNA (0,686 tỷ, 49,58%) (Bảng 4 và Hình 7). Biến động trong khu vực thuộc vùng có nguy cơ *P. falciparum* ổn định, được phân loại thành các cấp độ lưu hành thấp (*Pf*PR_2-10_ ≤5%), trung bình (*Pf*PR_2-10_ >5-<40%) và cao (*Pf*PR_2-10_ ≥40%) do các phân tích này hỗ trợ được trình bày dưới đây. Ở các nước châu Mỹ và TDNA, trẻ em (thuộc các nhóm tuổi 0-4 và 5-14) chiếm gần một phần ba (32% mỗi nhóm) tổng PAR. Ở châu Phi tỷ lệ này lên tới 43%.

Các nước châu Mỹ

Khu vực có sự lan truyền *P. falciparum* ổn định ở các nước châu Mỹ đặc trưng bởi mức độ lưu hành thấp đồng nhất (*Pf*PR_2-10_ ≤5%) (Hình 3 và 4). Tổng diện tích khu vực có nguy cơ ổn định bao trùm một khu vực rộng 6,03 triệu km^2^, hầu hết tập trung ở lưu vực sông Amazon (Hình 3 và 4). Tổng cộng có 40,64 triệu người trong khu vực này sống trong vùng có nguy cơ thấp này. Giá trị giữa của tỷ lệ nhiễm là 2,17% với *Pf*PR_2-10_ tiên đoán thấp nhất và cao nhất tương ứng là 0,31% và 8,81% (Hình 8C). Xem xét các phân bố tần số trong khu vực này cho thấy các giá trị tiên đoán phân bố khá đối xứng quanh giá trị giữa này (Hình 8, phần dưới). Dữ liệu đầu vào năm 2007 (Hình 8B) cho thấy xu hướng tương tự nhưng lệch dương, trong khi dữ liệu cho tất cả các năm bao gồm các giá trị có biên độ rộng hơn (max=21,30%) và cho thấy lệch dương rõ rệt (Hình 8A). Xác suất các phép gán đúng nhóm lưu hành cao ở các nước châu Mỹ (Hình S4.1A trong Nghi thức S4), chủ yếu là do tính chất khá đồng nhất của giá trị *Pf*PR_2-10_ thấp từ dữ liệu khảo sát [17,18], chứ không phải do cấu trúc không gian rạch ròi nào (, Hình S1.1 trong Nghi thức S1). Kết quả này, cùng với mật độ dân số khá thấp trong vùng, tạo ra các giá trị về chỉ số mức độ không chắc chắn có trọng số theo dân số là thấp nhất (Hình 5B).

châu Phi+

Khu vực có lan truyền ổn định *P. falciparum* bao trùm một vùng rộng 18,17 triệu km^2^, với 656,61 triệu người ở trong vùng có nguy cơ và có cường độ lan truyền với biên độ rộng. Trên 4,03 triệu km^2^ (22,18%) thuộc vùng này và 114,50 triệu người (17,44%) nằm trong nhóm *Pf*PR_2-10_ ≤5%. Những vùng này ở phạm vi trung tâm và phía đông của các vùng có vĩ độ cao nhất về phía bắc và thấp nhất về phía nam (Hình 3 và 4). Nhóm mức độ lưu hành này được tiên đoán tương đối đáng tin cậy (Hình S4.1A trong Nghi thức S4). Các vùng có sự lan truyền mạnh với *Pf*PR_2-10_ ≥40% chủ yếu ở Tây Phi và phần lớn Trung Phi, bao trùm một diện tích 8,50 triệu km2, với 345,28 triệu người ở trong vùng có nguy c. Xác suất tiên đoán đúng nhóm mức độ lưu hành cao ở khhu vực Tây Phi và thấp hơn nhiều ở khu vực Trung Phi (Hình S4.1C trong Nghi thức S4.3), do có khá nhiều dữ liệu khảo sát *Pf*PR_2-10_ mới ở khu vực thứ nhất và ít dữ liệu ở khu vực thứ hai (Hình 1). Một diện tích quan trong của lục địa này (5,63 triệu km^2^) có các giá trị mức độ lưu hành trung bình, *Pf*PR_2-10_ từ >5% đến <40%, và có 196,83 triệu người ở trong vùng có nguy cơ. Nhóm mức độ lưu hành này được tiên đoán với độ tin cậy thấp nhất (Hình S4.1B trong Nghi thức S4).

Giá trị giữa của tỷ lệ nhiễm tiên đoán được đối với vùng có mức độ lưu hành ổn định thuộc lục địa này là 33,34%, với các giá trị *Pf*PR_2-10_ tiên đoán cao nhất và thấp nhất tương ứng là 0,20% và 75,40% (Hình 8C). Phân bố tần số của các giá trị tiên đoán (Hình 8) tập trung ở giá trị giữa này, với phần thứ hai kém rõ ràng hơn rất nhiều tập trung ở mức 15% (Hình 8C). Phân bố này rất khác biệt giữa dữ liệu đầu vào của tất cả các năm và dữ liệu đầu vào trong năm 2007, cả hai đều lệch dương với các giá trị tối đa tương ứng là 99,78% và 98,70% (Các Hình tương ứng 8A và 8B). Chỉ số mức độ không chắc chắn lấy trọng số theo dân số cho thấy một bức tranh không đồng nhất của khu vực này, với các giá trị cao rõ rệt ở Ethiopia đối với nhóm có mức độ lưu hành thấp và các giá trị cao rõ rệt ở Nigeria trong nhóm có mức độ lưu hành cao (Hình 5B), phản ánh mật độ các điều tra *Pf*PR_2-10_ thấp và dân số lớn cùng hiện diện ở hai quốc gia này.

Trung, Đông và Nam châu Á (TDNA)

Vùng có lan truyền ổn định *P. falciparum* ở khu vực TNDA đặc trưng bởi mức độ lưu hành số rét thấp (*Pf*PR_2-10_ ≤5%), kèm với các vùng có diện tích nhỏ về mặt địa lý nhưng có tầm quan trọng dịch tễ thuộc nhóm có nguy cơ trung bình (*Pf*PR_2-10_ từ >5 đến <40%) và cao (*Pf*PR_2-10_ ≥40%), ví dụ như bang Orissa, đông Ấn Độ, tây Myanmar và vùng thấp của New Guinea. Tổng diện tích vùng nguy cơ ổn định bao trùm 5,53 triệu km^2^, với 685,65 triệu người sống trong vùng có nguy cơ, hầu hết tập trung ở Ấn Độ và Indonesia (Hình 3 và 4). Trên 4,72 triệu km^2^ (85,54%) thuộc khu vực này và 603,61 triệu người (88,03%) thuộc nhóm *Pf*PR_2-10_ ≤5%. Giá trị giữa của tỷ lệ nhiễm tiên đoán được là 9,99%, với giá trị *Pf*PR_2-10_ tiên đoán thấp nhất và cao nhất tương ứng là 0,006% và 45,40%. Phân bố tần số của các giá trị *Pf*PR_2-10_ lệch dương (Hình 8C). Phân bố tần số của dữ liệu đầu vào năm 2007 có biên độ tương tự nhưng cho thấy sự lệch dương rõ rệt hơn (Hình 8B). Đồ thị của dữ liệu từ tất cả các năm cũng lệch dương nhưng bao trùm một biên độ rộng hơn rất nhiều, với giá trị tối đa là 93,91% (Hình 8A). Xác suất của các phép gán nhóm mức độ lưu hành tương đối cao trong khu vực TDNA, nhưng có mức độ không chắc chắn đáng kể ở các khu vực biên giữa các nhóm lưu hành thấp và trung bình (Hình S4.1A trong Nghi thức S4.3). Tính chất này, cùng với mật độ dân số cao trong khu vực, dẫn đến chỉ số mức độ không chắc chắn lấy trọng số theo dân số là cao nhất, đặc biệt là ở Ấn Độ (Hình 5B).

**Thảo luận**

Lần đầu tiên trong 40 năm qua, với thông tin mà chúng tôi có được, chúng tôi đã cung cấp một bản đồ cập nhật về mức độ lưu hành sốt rét *P. falciparum*. Bản đồ này giải quyết được các thiếu sót chính của các bản đồ phân bố nguy cơ sốt rét toàn cầu cũ như đã thảo luận ở phần trên và do vậy nó là bản đồ duy nhất xét trên các phương diện sau đây. Thứ nhất, nó căn cứ trên một cơ sở dữ liệu điều tra sốt rét có phạm vi rộng về mặt địa lý và có cung cấp tài liệu rất chi tiết (Nghi thức S1) [58] và năm 2009 sẽ được công bố trên miền công cộng (với những điều tra nào được cho phép công bố) để mọi người đều có thể dùng và đánh giá [1]. Thứ hai, các phương pháp MBG (Nghi thức S3) và các quy trình kiểm chứng (Nghi thức S4) cũng được cung cấp tài liệu rất chi tiết và mã chương trình cũng khả dụng trên miền công cộng. Do vậy toàn bộ quá trình lập bản đồ có thể lặp lại được nếu có đủ điều kiện về máy tính. Thứ ba, một quá trình đánh giá chặt chẽ mức độ không chắc chắn đi kèm với các bản đồ sản phẩm đầu ra đã được thực hiện do vậy độ tin cậy của các kết quả có thể được đánh giá một cách khách quan (Hình 5).

Hiện trạng sốt rét của thế giới năm 2007

Thế giới ít sốt rét hơn rất nhiều so với tiên đoán khi xem xét các bản đồ lịch sử [5,14], cả do sự thu nhỏ về phạm vi không gian lẫn việc giảm mức độ lưu hành trong phạm vi đó. Có sự dịch chuyển rõ rệt sang xu hướng sinh thái học sốt rét nguy cơ thấp sẽ được nghiên cứu trong tương lai.

Trong số 1,382 tỷ người trên toàn cầu ở trong vùng có nguy cơ sốt rét ổn định của năm 2007, 0,759 tỷ người sống trong các điều kiện có mức độ lưu hành sốt rét cực kỳ thấp với *Pf*PR_2-10_ ≤5% thuộc các vùng TDNA (0,604 tỷ người, 79,55%), châu Phi+ (0,115 tỷ người, 15,09%) và châu Mỹ (0,041 tỷ người, 5,36%) (Bảng 4 và Hình 7). Người dân trong khu vực này sống trong các khu vực mà viễn cảnh sinh học cho việc kiểm soát lâu dài lan truyền sốt rét ở mức rất thấp có thể thực hiện được và phù hợp để loại bỏ hoàn toàn sốt rét trên quy mô dài hạn [19]. Tuy nhiên các khuyến cáo cho những vùng đặc biệt cũng như cho từng quốc giá cần phải được thiết lập dựa trên một đánh giá đúng đắn về các yếu tố môi trường, hậu cần, tài chính và chính trị tác động đến hiệu quả của các hoạt động phòng chống có thể được thực hiện [73,74,75]. Phần còn lại của dân số toàn cầu sống trong vùng có nguy cơ sốt rét ổn định là châu Phi: 0,197 tỷ người sống trong các điều kiện có nguy cơ trung bình (*Pf*PR_2-10_ từ >5 đến <40%) và 0,345 tỷ người sống trong vùng có nguy cơ cao (*Pf*PR_2-10_ ≥40%) (Bảng 4 và Hình 7). Ở những khu vực có nguy cơ trung bình, mô hình toán học cho thấy rằng bằng cách tăng cường ITN, việc cắt đứt làn truyền của sốt rét *P. falciparum* có thể đạt được, trong khi đó ở những vùng có lan truyền cao, sự lan truyền sốt rét sẽ khó khống chế hơn và đòi hỏi sự kiểm soát chặt chẽ cũng như các biện pháp phòng chống hỗ trợ [19,55].

Thực thi thống kê và kiểm chứng mô hình

Quy trình mô hình hóa trình bày ở đây cho thấy việc thực hiện ở quy mô lớn các kỹ thuật địa thống kê kiểu Bayes và tích hợp một số thành phần mới. Việc đưa vào mô hình chuẩn hóa tuổi cho phép tạo ra sự đồng nhất nhất quán của dữ liệu điều tra thu thập được ở nhiều độ tuổi khác nhau đồng thời có tính đến mức độ không chắc chắn sinh ra do sự biến động bổ sung này. Tương tự như vậy, việc dùng trường không-thời gian ngẫu nhiên đầy đủ cho phép đưa vào các điều tra cũ đến năm 1985 cũng có thể được tích hợp trong việc tiên đoán mức độ lưu hành của *P. falciparum* hiện tại trong một hệ thống hợp lý về mặt dịch tễ học và thống kê học.

Các kỹ thuật của MBG đòi hỏi yêu cầu tính toán cực kỳ cao ngay cả với các bài toán tiên đoán nhỏ. Chúng tôi được biết đây là lần đầu tiên những quy trình này đã được áp dụng cho bệnh tật ở mức độ toàn cầu. Gánh nặng về tính toán này cũng bao hàm một số giới hạn của quy trình mô hình hóa có thể giúp cải thiện khả năng tiên đoán. Đặc biệt là mô hình hiện tại chấp nhận trị trung bình đơn và hàm hiệp phương sai trong từng vùng, thể hiện giả định về tính dừng bậc hai trong mỗi vùng. Các phép tính xấp xỉ với các trường ngẫu nhiên không tĩnh được dùng trong các nghiên cứu ở quy mô nhỏ hơn [32,76] cho thấy khả năng có thể tinh chỉnh được mô hình hiện tại, nhưng được coi là không đủ khả năng máy tính để chạy ở mức toàn cầu.

Việc đánh giá các thống kê kiểm chứng khác nhau cho thấy mô hình này đạt yêu cầu về ba phương diện: tiên đoán các giá trị điểm *Pf*PR_2-10_ và nhóm mức độ lưu hành, và cung cấp các số đo thực tế về mức độ không chắc chắn của tiên đoán. Với bản chất rất biến động trong mức độ lưu hành của *P. falciparum* ngay cả trên một khoảng cách ngắn, mối tương quan tổng cộng 0,82 giữa các tiên đoán mô hình và dữ liệu kiểm chứng, cùng với độ lớn của sai số tuyệt đối trung bình là 9,75% *Pf*PR_2-10_ cho thấy mức kỹ năng dự báo độ chính xác hơn mong đợi. Một số khía cạnh của các số đo mức độ không chắc chắn chưa phải là tối ưu: đặc biệt là xu hướng hơi ước lượng thấp xác suất *Pf*PR_2-10_ có các giá trị rất thấp. Tuy nhiên, với tính chất mức độ không chắc chắn sinh ra từ nhiều nguồn và nhân lên trong toàn hệ thống mô hình hóa, các tiên đoán mức độ không chắc chắn cung cấp một nguồn thông tin dồi dào cho việc tạo các sản phẩm đầu ra cho những người ra quyết định.

Mô hình này được điều chỉnh cho phù hợp bằng cách dùng phương pháp MCMC [77,78]. MCMC là một thuật toán rất mạnh, và duy nhất dùng được cho mục đích đại cương, thuật toán tính toán dễ dùng khả dụng cho nhiều bài toán kiểu Bayes. Tuy nhiên, nó là một thuật toán tương đối. Không có phương pháp kiểm chứng thất bại để ước lượng sai số của nó, nhưng bằng cách dùng phương pháp phỏng đoán (Nghi thức S1.3) chúng tôi đã ước lượng được “sai số Monte Carlo” là không quan trọng so với mức độ không chắc chắn trong các phân bố hậu nghiệm thực của chúng tôi.

Thông tin có trong các bản đồ trình bày ở đây và mức độ không chắc chắn liên quan thay đổi trên các phạm vi địa lý khác nhau. Sự biến động ở trên tỷ lệ lớn về mức độ lưu hành được mô tả giữa các vùng và quốc gia là rõ rệt, được định lượng một cách chắc chắn và có thể sử dụng được trực tiếp cho các nhà lập kế hoạch ở mức toàn cầu. Tuy nhiên, khi các tỷ lệ ở mức độ chi tiết hơn được xem xét, việc dùng các bản đồ này cho các nhà quản lý trong kiểm soát sốt rét ở mức cục bộ bị giảm đi mặc dù việc này phụ thuộc rất nhiều vào tính khả dụng và mật độ các điểm điều tra. Ngưỡng phù hợp và số đo mức độ không chắc chắn sẽ thay đổi rất nhiều đối với những người dùng cuối khác nhau cũng như ứng dụng của các bản đồ. Tuy nhiên có một quy ước chung được đề xuất là sự phân biệt về mức độ lưu hành giữa các vùng dưới mức ranh giới hành chính cấp đầu tiên sẽ không phù hợp đối với hầu hết các quốc gia.

Việc kiểm tra các phân bố tần số của dữ liệu *Pf*PR_2-10_ tất cả các năm và riêng năm 2007 và kiểm tra bề mặt *Pf*PR_2-10_ tiên đoán được cho thấy một số tính chất quan trọng. Thứ nhất, dữ liệu năm 2007 từ cả ba khu vực cho thấy giá trị giữa nhỏ hơn đáng kể và giá trị tối đa lệch dương nhiều hơn so với dữ liệu của tất cả các năm được xem xét chung (so sánh Hình 8 ở trên và ở giữa). Thứ hai, có các khác biệt đáng chú ý ở tất cả các vùng giữa phân bố của các giá trị dữ liệu năm 2007 và phân bố của các giá trị từ bề mặt *Pf*PR_2-10_ tiên đoán (so sánh Hình 8A và 8B). Đặc biệt, các phân bố hậu nghiệm có các giá trị giữa lớn hơn, bị lệch dương ít hơn, đối với các nước châu Mỹ và châu Phi+ thì có các giá trị tối đa nhỏ hơn đáng kể. Sự dịch chuyển tổng thể về hướng giá trị *Pf*PR_2-10_ cao hơn trong các bề mặt tiên đoán có thể là do sự gộp nhóm không gian của các địa điểm điều tra. Cần luôn nhớ rằng tập hợp các điều tra được chọn biểu thị việc lấy mẫu có tính chất cơ hội chịu ảnh hưởng của động lực và hạn chế của nhiều cá nhân, tổ chức và chính quyền. Việc khảo sát trực quan tập hợp dữ liệu này cho thấy một tỷ lệ tương đối lớn dữ liệu tập trung ở những khu vực có mức độ lưu hành thấp hơn so với tình huống lấy mẫu ngẫu nhiên, và, do vậy, thống kê tóm tắt các dữ liệu thô này cho thấy độ sai lệch đáng kể. Bằng cách tiên đoán mức độ lưu hành trên một bề mặt liên tục, tiến trình MBG đã bù lại được hoàn toàn tính chất gộp nhóm trong các bản đồ đầu ra và phân bố tần số tạo thành không bị sai lệch theo cùng phương thức trên.

Tiến trình MBG thực hiện các tiên đoán tại những vị trí không có mẫu bằng cách dùng các tổ hợp tuyến tính của dữ liệu khảo sát. Vì lý do này, các bề mặt tạo thành hiển nhiên là trơn hơn so với dữ liệu thô mà chúng được tiên đoán từ đó. Một tính chất của tiến trình làm trơn này là biên độ của các giá trị cực cao và cực thấp trong bề mặt tiên đoán được có xu hướng nhỏ hơn giá trị của dữ liệu đầu vào. Điều này giải thích tại sao các phân bố tần số của bề mặt *Pf*PR_2-10_ tiên đoán có các khoảng biên độ nhỏ hơn các giá trị của dữ liệu đầu vào. Một hệ quả của hiệu ứng làm trơn này là bề mặt tiên đoán cho phép tiên đoán tốt hơn mức độ lưu hành ở quy mô lớn nhưng lại kém khả năng hơn trong việc biểu thị xác thực các biến động ở quy mô nhỏ sinh ra trên các khoảng cách rất ngắn.

Dùng các đồng biến số môi trường để tạo các bản đồ liên tục

Các ảnh hưởng giới hạn ở mức ngưỡng của các đồng biến số môi trường đã được tích hợp một cách đầy đủ vào việc định nghĩa giới hạn ổn định và không ổn định của sự lan truyền sốt rét *P. falciparum* đã mô tả ở trên [18]. Tính chất hấp dẫn trong việc sử dụng xa hơn các đồng biến số môi trường để gia tăng mức độ phức tạp cũng như cải thiện khả năng tiên đoán của việc lập bản đồ mức độ lưu hành bằng MBG là điều không có thật. Đó là do các phân tích như vậy được căn cứ vào giả định rằng phân bố hiện tại và mức độ lưu hành của sốt rét tương quan với ổ sinh thái cơ bản của nó [79,80]. Giả định này không được xác nhận bởi vì phân bố toàn cầu của sốt rét đã bị co lại một cách đáng kể [18] do phân bố tối đa giả định của bệnh này vào khoảng năm 1900 [14]. Hơn nữa, người ra không biết các yếu tố quyết định về mặt môi trường của phân bố hiện nay phản ánh ổ sinh thái cơ bản này đến mức nào, các mối quan hệ này có thể thay đổi về mặt không gian như thế nào, và do đó, tác động giả nào có thể sinh ra bởi việc đưa chúng vào các phân tích. Ngoài ra, không dễ dàng gì thu thập được “đủ” các đồng biến số môi trường ở mức toàn cầu với độ xác thực về không gian và thời gian cần thiết [63,81]. Sau cùng, mức độ các mối quan hệ này có thể trở nên không rõ ràng hơn nữa như thế nào do nỗ lực phòng chống bệnh hiện đang diễn ra biến động về mặt không gian là không thể định lượng được. Việc gia tăng các điểm chứng minh cho những nỗ lực phòng chống bệnh này đang trở nên ngày càng quan trọng, và tăng nhanh hơn kể từ sau năm 2000 và biểu thị tính chất khảm về mặt không gian có thể tác động làm sai lệch đáng kể bất kỳ các mối quan hệ được mô hình hóa nào . Điều không có gì ngạc nhiên là không có phép thống kê hỗ trợ nào dùng được để đưa vào tập hợp các đồng biến số môi trường về khí hậu [62] và viễn thám [63] (Nghi thức S1.7).

Với việc tránh sử dụng các đồng biến số môi trường trong hệ thống phân tích này, các bản đồ đầu ra chỉ được quyết định bởi dữ liệu khảo sát đầu vào và các giả định của việc mô hình hóa. Việc chọn phương án này đảm bảo tính chất cơ bản hạn chế tối đa, mà dựa vào đó các thay đổi trong tương lai có thể được điều chỉnh.

Các cải thiện có thể về mặt địa lý thống kê

Khi theo phương pháp MBG, ý nghĩa của việc loại bỏ các điều tra có kích thước mẫu dưới 50 không còn nữa, vì mức độ không chắc chắn liên quan đến số lượng mẫu đã được mô hình hóa một cách rạch ròi trong kỹ thuật này (Nghi thức S3). Quy tắc loại bỏ này đã được đặt ra vào thời điểm trước khi MBG có thể được áp dụng cho quy mô toàn cầu và sẽ được xem xét lại trong các lần chạy lại bản đồ trong tương lai. Công tác trích xuất dữ liệu khổng lồ này không dễ dàng thực hiện đối với nỗ lực lập bản đồ hiện nay.

Độ phân giải không gian mà các kỹ thuật MBG này có thể áp dụng được cho một cụm máy tính là lưới 5x5 km. Toàn bộ tiến trình này mất trung bình một tháng với độ phân giải không gian này và với độ phân giải không gian 1x1 km ước lượng sẽ mất khoảng ba tháng. Chúng tôi không dự kiến tăng độ phân giải không gian ở quy mô toàn cầu vì chúng đủ mạnh cho các mục đích lập kế hoạch ở mức vùng, là mục tiêu chính của mô hình này. Tuy vậy, đối với các khu vực ở quy mô nhỏ hơn, ví dụ như các nước có nhiều dữ liệu *Pf*PR, nơi cần có các bản đồ có độ phân giải không gian cao hơn để hỗ trợ các kế hoạch kiểm soát ở mức quốc gia, có thể xem xét việc thực hiện các sản phẩm đầu ra MBG với lưới phân giải 1x1 km [33]. Hơn nữa, ở quy mô quốc gia này, tính chất xác thực trong việc xác định tọa độ của dữ liệu điều tra *Pf*PR đầu vào có thể có ảnh hưởng quan trọng lên mức độ không chắc chắn của các tiên đoán, do vậy các quy trình có thể trợ giúp để đưa các tác động này vào việc mô hình hóa cần phải được nghiên cứu [91-93]. Trong nghiên cứu này, mức độ không chắc chắn một phần do các sai số về xác định tọa độ được cho là không đáng kể so với quy mô của sự biến động về mặt không gian trong mức độ lưu hành ghi nhận được và các sản phẩm đầu ra mô hình ở quy mô toàn cầu.

Chúng tôi chưa thể cải thiện hiệu quả tiên đoán của mô hình bằng cách mô hình hóa độ nhạy phụ thuộc vào tuổi của các chẩn đoán hiển vi và chẩn đoán nhanh một cách riêng biệt hoặc bằng cách mô hình hoá độ đặc hiệu của chẩn đoán. Độ chính xác trong việc xác định *Pf*PR bằng phương pháp hiển vi hoặc phép chẩn đoán nhanh được giả định là như nhau trong các phân tích này, nhưng độ nhạy của kỹ thuật chẩn đoán có thể đưa vào trong các lần chạy sau này của hệ thống MBG.

Hiện vẫn chưa có giải pháp nào cho việc áp dụng các kỹ thuật MBG này ngang qua phần lớn diện tích các đại dương (ví dụ như vùng Caribe, Madagascar và quần đảo thuộc Indonesia), trong điều kiện phân bố toàn cầu của dữ liệu *Pf*PR và việc thiếu dữ liệu ở một số khu vực (Hình 1). Các ảnh hưởng tiềm tàng của yếu tố địa-sinh học lên sự lan truyền sốt rét trên các đảo bị bỏ qua trong các phân tích này. Điều lý tưởng là các lần chạy lặp bản đồ trong tương lai sẽ có đủ dữ liệu để xử lý các đảo một cách riêng biệt hoặc có đủ thông tin về phân bố của các vector *Anopheles* để hỗ trợ thông tin cho các tiên đoán [56].

Chúng tôi đã đưa vào các phân tích tính chất hiểu được xu hướng độ cũ trong dữ liệu *Pf*PR và các biến độ trong năm của sự lan truyền. Bản đồ này không cung cấp mô tả đầy đủ về động học sốt rét theo mùa , tuy nhiên thông tin bổ sung về biến động toàn cầu về tính chất theo mùa của sốt rét có thể được đưa vào các lần chạy bản đồ sau này.

Khả năng trong tương lai: miền công cộng và bản đồ động

Mọi người đều có thể truy cập vào các bản đồ kết quả này trên mạng internet cùng với việc xuất bản bài báo này. Dữ liệu nguồn được dùng trong các tiên đoán của các bản đồ được lên kế hoạch công bố vào năm 2009 [1] và kiến trúc nội tại trực tuyến để chạy dịch vụ này hiện đang được phát triển. Nhóm nghiên cứu MAP dự kiến cung cấp các cập nhật hàng năm cho bản đồ mức độ lưu hành sốt rét toàn cầu này cùng với cơ sở dữ liệu *Pf*PR đi kèm. Các cập nhật hàng năm cũng cần thiết để phản ánh thay đổi về giới hạn không gian của sự lan truyền sốt rét *P. falciparum* ở mức ổn định và không ổn định [18], để xác định chính xác giới hạn mà các tiên đoán mức độ lưu hành cần thực hiện. Nếu cộng đồng quốc tế thành công trong việc giảm bớt sốt rét, cần có các quyết định tính đến tính chất không liên tục về mặt thời gian giữa các giới hạn không gian của sự lan truyền sốt rét *P. falciparum* (nếu được thì xác định bằng trị trung bình của *Pf*API trong ba năm gần nhất [18]) và dữ liệu về mức độ lưu hành (*Pf*PR thu thập từ năm 1985).

Rõ ràng là bản đồ dự báo thể hiện một cái nhìn vắn tắt trong năm 2007 có từ mức độ lưu hành sốt rét thay đổi theo thời gian. Không có tính chất thống kê phức tạp nào có thể làm sai một thực tế là dữ liệu bổ sung sẽ làm tăng cường tính xác thực của bản đồ, bằng cách tăng độ phân giải không gian của các điều tra sốt rét hoặc cập nhật các địa điểm khảo sát hiện có với thông tin cập nhật hơn. Các phương pháp đã được phát triển một cách đặc thù sao cho các bề mặt này có thể được cập nhật nhanh chóng. Phương pháp tiếp cận chủ đạo một biến được dùng cũng có nghĩa là các thay đổi trong các lần chạy bản đồ sau này có thể được xác định tính chất một cách đáng tin cậy đối với việc tìm thêm được dữ liệu ở các vùng có mức độ chắc chắn không cao (các thay đổi về không gian) hoặc đối với các thay đổi sinh ra do thành công trong phòng chống bệnh hoặc sự giảm bớt bệnh (các thay đổi theo thời gian), chứ không phải do bất kỳ sự trộn lẫn không gian và thời gian nào của mối liên hệ dữ liệu *Pf*PR_2-10_ với các đồng biến số môi trường.

Chúng tôi rất hoan nghênh việc cung cấp dữ liệu bổ sung để cải thiện bản đồ ở các vùng có độ chính xác thấp, và dữ liệu mới để duy trì việc tạo ra bản đồ cập nhật trong tương lai. Các vùng hiện nay có độ chắc chắn thấp nhất được chỉ ra bằng cách ước lượng tương đối tốt bởi giá trị nghịch đảo của xác suất tiên đoán nhóm (Hình 5), mặc dù công việc tương lai là nhằm vào mục đích tinh chỉnh thông tin này. Do vậy, ưu tiên hiện nay là tạo ra các bản đồ khu vực thể hiện được địa điểm tối ưu của các điều tra mới cần phải được thực hiện để làm giảm thiếu tối đa phương sai trong bề mặt mức độ lưu hành hiện nay với chi phí tối thiểu. Các giải pháp này cần thiết hơn nhiều so với một danh sách các vùng có phương sai cao nhất được liệt kê ở đây bởi vì (i) mỗi điều tra mới sẽ làm thay đổi cấu trúc của phương sai không gian và tác động đến địa điểm tối ưu cho điều tra tiếp theo; (ii) cả số lượng lẫn phân bố không gian của các điều tra sẽ tác động để kết quả và cần có nhiều giả lập để tập trung được vào các giải pháp tối ưu và (iii) các địa điểm điều tra tiềm tàng cần phải được cân đối một cách phù hợp với phân bố của dân số.

Các mục tiêu tức thời của MAP

Trọng tâm ban đầu của MAP là *P. falciparum* [1] là do tầm quan trọng dịch tễ toàn cầu của nó [102] và triển vọng kiểm soát và loại bỏ nó tốt hơn trên bình diện cục bộ [19]. Chúng tôi chưa đánh giá tầm quan trọng về gánh nặng của *P. vivax* (*Pv*PR) mặc dù tầm quan trọng của nó về mặt lâm sàng ngày càng cao , tuy vậy chúng tôi đã lưu trữ trên 2500 điều tra *Pv*PR để bắt đầu tiến trình này. Một mục tiêu tức thời khác là tinh chỉnh lại gánh nặng toàn cầu về ước lượng bệnh do *P. falciparum* (cả về tỷ lệ mắc bệnh [102] lẫn tỷ lệ tử vong ) để hỗ trợ cho việc ước lượng trên quy mô toàn cầu các nhu cầu về biện pháp phòng chống cũng như trang thiết bị. Các phương pháp thống kê được dùng trong phân tích này cho phép chạy các ước lượng gánh nặng trong tương lai để biểu thị chính quy và chắc chắn hơn trong các bản đồ sắp tới về phân bố của các vector *Anopheles* chủ đạo gây bệnh sốt rét trên người [56] sẽ giúp tăng cường sức mạnh cho các nhà quản lý về kiểm soát sốt rét để có được các quyết định có tính chất hướng dẫn tốt hơn liên quan đến các biện pháp phòng chống phù hợp với sinh thái học của các nhóm vector địa phương. Trên quy mô dài hạn chúng tôi hy vọng sẽ không chỉ giám sát và đánh giá tiến trình bằng các bản đồ này và còn tăng cường khả năng của chúng tôi trong việc mô hình hóa mức độ lưu hành sốt rét trong tương lại và hỗ trợ cho việc đánh giá khách quan những nơi nào trên thế giới có thể loại bỏ hoàn toàn sốt rét.

Kết luận

Hiện trạng của sốt rét *Plasmodium falciparum* toàn cầu trong năm 2007 cho thấy một cơ hội vô cùng to lớn cho cộng đồng quốc tế hành động [109,110], tuy nhiên các hành động ấy vẫn còn thiếu nguồn lực đáng kể [111]. Bất kể các quốc gia có duy trì và kiểm soát bệnh này hay nhằm vào tham vọng cao hơn là loại bỏ sốt rét hay không thì các phương hướng phòng chống tức thời vẫn là như nhau [19]. Nguồn “tài nguyên” về bản đồ này sẽ giúp cho các quốc gia xác định nhu cầu của họ và có tác dụng làm cơ sở cho việc giám sát và đánh giá các tiến độ của các mục tiêu phòng chống. Chúng tôi hy vọng sẽ tiếp tục được làm việc với các cá nhân, các quốc gia và các khu vực để cải thiện các lần chạy bản đồ trong tương lai và hy vọng ghi nhận được các thành công trong công tác phòng chống sốt rét.

**Thông tin bổ trợ**

**Ngôn ngữ khác S1.**

Bản dịch bài báo sang tiếng Pháp của Frédéric Piel và Stéphanie Loute
Có tại doi:10.1371/journal.pmed.1000048.sd001 (1.04 MB DOC).

**Ngôn ngữ khác S2.**

Bản dịch bài báo sang tiếng Trung Quốc của Robert Li
Có tại doi:10.1371/journal.pmed.1000048.sd002 (437 KB DOC).

**Ngôn ngữ khác S3.**

Bản dịch bài báo sang tiếng Indonesia của Iqbal R.F. Elyazar và Siti Nurlela
Có tại doi:10.1371/journal.pmed.1000048.sd003 (1.07 MB DOC).

**Ngôn ngữ khác S4.**

Bản dịch bài báo sang tiếng Việt của Bùi H. Mạnh
Có tại doi:10.1371/journal.pmed.1000048.sd004 (574 KB DOC).

**Ngôn ngữ khác S5.**

Bản dịch bài báo sang tiếng Tây Ban Nha của Carlos A. Guerra
Có tại doi:10.1371/journal.pmed.1000048.sd004 (774 KB DOC).

**Nghi thức S1**.

Cơ sở dữ liệu các điều tra tỷ lệ ký sinh trùng *Plasmodium falciparum*

S1.1 Tóm tắt các quy trình tìm kiếm và tóm lược dữ liệu

S1.2 Các quy định loại bỏ dữ liệu

S1.3 Chuẩn hóa tuổi

S1.4 Semivariogram của Dữ liệu *Pf*PR_2-10_ theo vùng

S1.5 Bộ lọc địa thống kê dùng để phát hiện các giá trị ngoại biên

S1.6 Tóm tắt dữ liệu điều tra sốt rét và thống kê mô tả

S1.7 Các mối liên hệ với các đồng biến số môi trường

Có tại doi:10.1371/journal.pmed.1000048.sd005 (3.4 MB DOC).

**Nghi thức S2**.

Các cơ sở dữ liệu nhân khẩu và các quy trình liên quan đến nhân khẩu

S2.1 Các quy định phân loại điều tra tỷ lệ ký sinh trùng đô thị/gần đô thị/nông thôn
S2.2 Hiện trạng đô thị/gần đô thị/nông thôn và mức độ phổ biến
S2.3 Bề mặt dân số GRUMP alpha
S2.4 Dẫn xuất dân số trong vùng có nguy cơ

Có tại doi:10.1371/journal.pmed.1000048.sd006 (2.5 MB DOC).

**Nghi thức S3**.

Các quy trình địa thống kê dựa trên mô hình

S3.1 Tổng quan của mô hình thống kê
S3.2 Đặc thù của tiên nghiệm
S3.3 Chuẩn hóa tuổi
S3.4 Chi tiết thực hiện
S3.5 Tổng quan việc tạo bản đồ

Có tại doi:10.1371/journal.pmed.1000048.sd007 (23 MB DOC).

**Nghi thức S4**.

Các quy trình kiểm chứng mô hình

S4.1 Tạo các tập hợp kiểm chứng
S4.2 Các quy trình kiểm tra hiệu quả của mô hình
S4.3 Các kết quả bổ sung

Có tại doi:10.1371/journal.pmed.1000048.sd008 (26 MB DOC).

**Lời cảm ơn**

Việc thu thập dữ liệu về các điều tra tỷ lệ ký sinh trùng toàn cầu phụ thuộc rất nhiều vào đóng góp dữ liệu rộng lượng của một số lượng lớn các cá nhân trong các đơn vị nghiên cứu và kiểm soát soát rét và những cá nhân này được liệt kê trên trang web của MAP (http://www.map.ac.uk/acknowledgements.html). Chúng tôi cũng cám ơn Archie Clements đã có những nhận xét cho bản thảo tài liệu này. Các tác giả cám ơn hỗ trợ của Viện Nghiên cứu Y khoa Kenya (KEMRI) và tài liệu này được xuất bản với sự cho phép của giám đốc KEMRI.

**Đóng góp của các tác giả**

SIH viết bản thảo đầu tiên. RWS, AMN, CWK, BHM, IRFE và SIH tập hợp và lập bản đồ dữ liệu PfPR. SIH và RWS phát triển các thử nghiệm. AJT, AP và PWG tinh chỉnh và thực hiện các nghi thức thực nghiệm. DS phát triển quy trình chuẩn hóa tuổi. SB và RAM đóng vai trò cố vấn thống kê trong suốt quá trình nghiên cứu. Tất cả các tác giả đều đã có đóng góp cho bản thảo cuối cùng.

**Tài liệu tham khảo**

1. Hay SI, Snow RW (2006) The Malaria Atlas Project: developing global maps of malaria risk. PLoS Med 3: e473.

2. Horton (2007) Is malaria eradication possible? Lancet 370: 1459.

3. Roberts L, Enserink M (2007) Did they really say … eradication? Science 318: 1544-1545.

4. Feachem R, Sabot O (2008) A new global malaria eradication strategy. Lancet 10: 1633-1635.

5. Lysenko AJ, Semashko IN (1968) Geography of malaria. A medico-geographic profile of an ancient disease [in Russian]. In: Lebedew AW, editor. Itogi Nauki: Medicinskaja Geografija. Moscow: Academy of Sciences, USSR. pp. 25-146.

6. Boyd MF (1930) An introduction to malariology. Cambridge, Massachusetts: Harvard University Press.

7. Pampana EJ, Russell PF (1955) Le Paludisme: problème mondial. Geneva: World Health Organization. pp. 317-321.

8. Macdonald G (1957) Local features of malaria. The epidemiology and control of malaria. London: Oxford University Press. pp. 63-99.

9. W.H.O. (1966) Malaria eradication in 1965. World Health Organ Chron 20: 286-300.

10. Dutta HM, Dutt AK (1978) Malarial ecology: a global perspective. Soc Sci Med 12: 69-84.

11. W.H.O. (1977) Information on the world malaria situation. Wkly Epidemiol Rec 52.

12. W.H.O. (1994) World malaria situation in 1992. Part II. Wkly Epidemiol Rec 69: 317-324.

13. W.H.O. (1997) World malaria situation in 1994. Part I. Wkly Epidemiol Rec 72: 269-274.

14. Hay SI, Guerra CA, Tatem AJ, Noor AM, Snow RW (2004) The global distribution and population at risk of malaria: past, present, and future. Lancet Infect Dis 4: 327-336.

15. Kiszewski A, Mellinger A, Spielman A, Malaney P, Sachs SE, et al. (2004) A global index representing the stability of malaria transmission. Am J Trop Med Hyg 70: 486-498.

16. Mouchet J, Carnevale P, Coosemans M, Julvez J, Manguin S, et al. (2004) Paludisme et grandes régions biogéographiques. Biodiversité du paludisme dans le monde. Montrouge, France: John Libbey Eurotext.

17. Guerra CA (2007) Mapping the contemporary global distribution limits of malaria using empirical data and expert opinion [Doctoral thesis]. Oxford: University of Oxford. 258 p.

18. Guerra CA, Gikandi PW, Tatem AJ, Noor AM, Smith DL, et al. (2008) The limits and intensity of *Plasmodium falciparum* transmission: implications for malaria control and elimination worldwide. PLoS Med 5: e38.

19. Hay SI, Smith DL, Snow RW (2008) Measuring malaria endemicity from intense to interrupted transmission. Lancet Infect Dis 8: 369-378.

20. Yekutiel P (1980) III The Global Malaria Eradication Campaign. In: Klingberg MA, editor. Eradication of infectious diseases: a critical study. Basel, Switzerland: Karger. pp. 34-88.

21. Pampana E (1969) A textbook of malaria eradication. London: Oxford University Press.

22. Kleinschmidt I, Bagayoko M, Clarke GPY, Craig M, Le Sueur D (2000) A spatial statistical approach to malaria mapping. Int J Epidemiol 29: 355-361.

23. Kleinschmidt I, Omumbo J, Briet O, van de Giesen N, Sogoba N, et al. (2001) An empirical malaria distribution map for West Africa. Trop Med Int Health 6: 779-786.

24. Rogers DJ, Randolph SE, Snow RW, Hay SI (2002) Satellite imagery in the study and forecast of malaria. Nature 415: 710-715.

25. Omumbo JA, Hay SI, Snow RW, Tatem AJ, Rogers DJ (2005) Modelling malaria risk in East Africa at high-spatial resolution. Trop Med Int Health 10: 557-566.

26. Rogers DJ (2006) Models for vectors and vector-borne diseases. Adv Parasitol 62: 1-35.

27. Diggle PJ, Tawn JA, Moyeed RA (1998) Model-based geostatistics. J Roy Stat Soc C-App 47: 299-326.

28. Diggle P, Moyeed R, Rowlingson B, Thomson M (2002) Childhood malaria in The Gambia: a case-study in model-based geostatistics. J Roy Stat Soc C-App 51: 493-506.

29. Gemperli A, Vounatsou P, Kleinschmidt I, Bagayoko M, Lengeler C, et al. (2004) Spatial patterns of infant mortality in Mali: the effect of malaria endemicity. Am J Epidemiol 159: 64-72.

30. Gemperli A, Vounatsou P, Sogoba N, Smith T (2006) Malaria mapping using transmission models: application to survey data from Mali. Am J Epidemiol 163: 289-297.

31. Rattanasiri S, Bohning D, Rojanavipart P, Athipanyakom S (2004) A mixture model application in disease mapping of malaria. Southeast Asian J Trop Med Public Health 35: 38-47.

32. Gosoniu L, Vounatsou P, Sogoba N, Smith T (2006) Bayesian modelling of geostatistical malaria risk data. Geospat Health 1: 127-139.

33. Noor AM, Clements ACA, Gething PW, Moloney G, Borle M, et al. (2008) Spatial prediction of *Plasmodium falciparum* prevalence in Somalia. Malar J 7: 159.

34. Clements ACA, Lwambo NJS, Blair L, Nyandindi U, Kaatano G, et al. (2006) Bayesian spatial analysis and disease mapping: tools to enhance planning and implementation of a schistosomiasis control programme in Tanzania. Trop Med Int Health 11: 490-503.

35. Clements ACA, Moyeed R, Brooker S (2006) Bayesian geostatistical prediction of the intensity of infection with *Schistosoma mansoni* in East Africa. Parasitology 133: 711-719.

36. Diggle PJ, Thomson MC, Christensen OF, Rowlingson B, Obsomer V, et al. (2007) Spatial modelling and the prediction of *Loa loa* risk: decision making under uncertainty. Ann Trop Med Parasitol 101: 499-509.

37. Beck-Worner C, Raso G, Vounatsou P, N'Goran EK, Rigo G, et al. (2007) Bayesian spatial risk prediction of *Schistosoma mansoni* infection in western Cote d'Ivoire using a remotely-sensed digital elevation model. Am J Trop Med Hyg 76: 956-963.

38. Raso G, Matthys B, N'Goran EK, Tanner M, Vounatsou P, et al. (2005) Spatial risk prediction and mapping of *Schistosoma mansoni* infections among schoolchildren living in western Cote d'Ivoire. Parasitology 131: 97-108.

39. Chilès J-P, Delfiner P (1999) Geostatistics: modeling spatial uncertainty. Toronto: John Wiley and Sons. 720 p.

40. Goovaerts P (1997) Geostatistics for natural resource evaluation. New York, U.S.A.: Oxford University Press. 483 p.

41. Matheron G (1971) The theory of regionalized variables and its applications. Fontainebleau, France: Ecole Nationale Supérieure des Mines de Paris. 211 p.

42. Lawson AB (2001) Statistical methods in spatial epidemiology. Chichester: John Wiley and Sons. 277 p.

43. Best N, Richardson S, Thomson A (2005) A comparison of Bayesian spatial models for disease mapping. Stat Methods Med Res 14: 35-59.

44. Gething PW, Noor AM, Gikandi PW, Hay SI, Nixon MS, et al. (2008) Developing geostatistical space-time models to predict outpatient treatment burdens from incomplete national data. Geogr Anal 40: 167-188.

45. Gething PW, Noor AM, Goodman CA, Gikandi PW, Hay SI, et al. (2007) Information for decision making from imperfect national data: tracking major changes in health care use in Kenya using geostatistics. BMC Med 5: 37.

46. Wang XH, Zhou XN, Vounatsou P, Chen Z, Utzinger J, et al. (2008) Bayesian spatio-temporal modeling of *Schistosoma japonicum* prevalence data in the absence of a diagnostic 'gold' standard. PLoS Negl Trop Dis 2: e250.

47. Yang GJ, Vounatsou P, Zhou XN, Tanner M, Utzinger J (2005) A Bayesian-based approach for spatio-temporal modeling of county level prevalence of *Schistosoma japonicum* infection in Jiangsu province, China. Int J Parasitol 35: 155-162.

48. Hay SI, Guerra CA, Tatem AJ, Atkinson PM, Snow RW (2005) Urbanization, malaria transmission and disease burden in Africa. Nat Rev Microbiol 3: 81-90.

49. Robert V, MacIntyre K, Keating J, Trape JF, Duchemin JB, et al. (2003) Malaria transmission in urban sub-Saharan Africa. Am J Trop Med Hyg 68: 169-176.

50. Tatem AJ, Guerra CA, Kabaria CW, Noor AM, Hay SI (2008) Human population, urban settlement patterns and their impact *Plasmodium falciparum* malaria endemicity. Malar J 7: 218.

51. Smith DL, Guerra CA, Snow RW, Hay SI (2007) Standardizing estimates of the *Plasmodium falciparum* parasite rate. Malar J 6: 131.

52. Swaroop S (1959) Statistical considerations and methodology in malaria eradication. Part I. Statistical considerations. WHO/Mal/240. Geneva: World Health Organization.

53. Swaroop S (1959) Statistical considerations and methodology in malaria eradication. Part II. Statistical methodology. WHO/Mal/240. Geneva: World Health Organization.

54. Swaroop S, Gilroy AB, Uemura K (1966) Statistical methods in malaria eradication. Geneva: World Health Organization. 164 p.

55. Smith DL, Smith T, Hay SI (2009) Measuring malaria for elimination. Chapter seven. Shrinking the malaria map: a prospectus on malaria elimination. California: Malaria Elimination Group, University of California Santa Cruz. In press.

56. Hay SI, Sinka ME, Tatem AJ, Patil AP, Guerra CA, et al. (2009) Developing global maps of the dominant *Anopheles* vectors of human malaria. PLoS Med. In Press.

57. Metselaar D, Van Thiel PH (1959) Classification of malaria. Trop Geogr Med 11: 157-161.

58. Guerra CA, Hay SI, Lucioparedes LS, Gikandi PW, Tatem AJ, et al. (2007) Assembling a global database of malaria parasite prevalence for the Malaria Atlas Project. Malar J 6: 17.

59. Pull JH, Grab B (1974) Simple epidemiological model for evaluating malaria inoculation rate and risk of infection in infants. Bull World Health Organ 51: 507-516.

60. Balk DL, Deichmann U, Yetman G, Pozzi F, Hay SI, et al. (2006) Determining global population distribution: methods, applications and data. Adv Parasitol 62: 119-156.

61. CIESIN/IFPRI/WB/CIAT (2007) Global Rural Urban Mapping Project (GRUMP) alpha: Gridded Population of the World, version 2, with urban reallocation (GPW-UR). Available at http://sedac.ciesin.columbia.edu/gpw. Palisades, New York, USA: Center for International Earth Science Information Network, Columbia University / International Food Policy Research Institute / The World Bank / and Centro Internacional de Agricultura Tropical.

62. New M, Lister D, Hulme M, Makin I (2002) A high-resolution data set of surface climate over global land areas. Clim Res 21: 1-25.

63. Scharlemann JPW, Benz D, Hay SI, Purse BV, Tatem AJ, et al. (2008) Global data for ecology and epidemiology: a novel algorithm for temporal Fourier processing MODIS data. PLoS One 3: e1408.

64. Stein ML (2005) Space-time covariance functions. J Am Stat Assoc 100: 310-321.

65. Gething PW, Noor AM, Gikandi PW, Ogara EAA, Hay SI, et al. (2006) Improving imperfect data from health management information systems in Africa using space-time geostatistics. PLoS Med 3: e271.

66. Saito H, Goovaerts P (2000) Geostatistical interpolation of positively skewed and censored data in a dioxin-contaminated site. Environ Sci Technol 34: 4228-4235.

67. Brooker S, Hay SI, Bundy DA (2002) Tools from ecology: useful for evaluating infection risk models? Trends Parasitol 18: 70-74.

68. Moyeed RA, Papritz A (2002) An empirical comparison of kriging methods for nonlinear spatial point prediction. Math Geol 34: 365-386.

69. Hintze JL, Nelson RD (1998) Violin plots: a box plot-density trace synergism. Am Stat 52: 181-184.

70. U.N.P.D (2005) World urbanization prospects: population database. http://esa.un.org/unup/. New York: United Nations Population Division.

71. Hay SI, Noor AM, Nelson A, Tatem AJ (2005) The accuracy of human population maps for public health application. Trop Med Int Health 10: 1073-1086.

72. U.N.P.D. (2006) World population prospects: the 2006 revision population database. http://esa.un.org/unpp/. New York: United Nations Population Division.

73. W.H.O. (2007) Malaria elimination: a field manual for low and moderate endemic countries. Geneva: World Health Organization. 85 p.

74. Lines J, Whitty CJM, Hanson K (2007) Prospects for eradication and elimination of malaria: a technical briefing for DFID. London: London School of Hygiene & Tropical Medicine.

75. W.H.O. (2006) Informal consultation on malaria elimination: setting up the WHO agenda. WHO/HTM/MAL/2006.1114. Geneva: World Health Organization. 68 p.

76. Raso G, Vounatsou P, Gosoniu L, Tanner M, N'Goran EK, et al. (2006) Risk factors and spatial patterns of hookworm infection among schoolchildren in a rural area of western Côte d'Ivoire. Int J Parasitol 36: 201-210.

77. Gelman A, Carlin JB, Stern HS (2003) Bayesian Data Analysis. Texts in Statistical Science. Boca Raton, Florida, U.S.A.: Chapman & Hall / CRC Press LLC. 696 p.

78. Gilks WR, Spiegelhalter DJ (1999) Markov Chain Monte Carlo in Practice. Interdisciplinary statisics. Boca Raton, Florida, U.S.A.: Chapman & Hall / CRC Press LLC.

79. Hutchinson GE (1957) Concluding remarks. Cold Spring Harbor Symp Quant Biol 22: 415-427.

80. Southwood TRE (1977) Habitat, templet for ecological strategies? Presidential address to British Ecological Society, 5 January 1977. J Anim Ecol 46: 337-365.

81. Hay SI, Tatem AJ, Graham AJ, Goetz SJ, Rogers DJ (2006) Global environmental data for mapping infectious disease distribution. Adv Parasitol 62: 37-77.

82. Barat LM (2006) Four malaria success stories: how malaria burden was successfully reduced in Brazil, Eritrea, India, and Vietnam. Am J Trop Med Hyg 74: 12-16.

83. Barnes KI, Watkins WM, White NJ (2008) Antimalarial dosing regimens and drug resistance. Trends Parasitol 24: 127-134.

84. Bhattarai A, Ali AS, Kachur SP, Martensson A, Abbas AK, et al. (2007) Impact of artemisinin-based combination therapy and insecticide-treated nets on malaria burden in Zanzibar. PLoS Med 4: e309.

85. Fegan GW, Noor AM, Akhwale WS, Cousens S, Snow RW (2007) Effect of expanded insecticide-treated bednet coverage on child survival in rural Kenya: a longitudinal study. Lancet 370: 1035-1039.

86. Noor AM, Amin AA, Akhwale WS, Snow RW (2007) Increasing coverage and decreasing inequity in insecticide-treated bed net use among rural Kenyan children. PLoS Med 4: e255.

87. Nyarango PM, Gebremeskel T, Mebrahtu G, Mufunda J, Abdulmumini U, et al. (2006) A steep decline of malaria morbidity and mortality trends in Eritrea between 2000 and 2004: the effect of combination of control methods. Malar J 5: 33.

88. Okiro EA, Hay SI, Gikandi PW, Sharif SK, Noor AM, et al. (2007) The decline in paediatric malaria admissions on the coast of Kenya. Malar J 6: 151.

89. Schellenberg D, Menendez C, Aponte J, Guinovart C, Mshinda H, et al. (2004) The changing epidemiology of malaria in Ifakara Town, southern Tanzania. Trop Med Int Health 9: 68-76.

90. Noor AM, Moloney G, Borle M, Fegan GW, Shewchuk T, et al. (2008) The use of mosquito nets and the prevalence of *Plasmodium falciparum* infection in rural South Central Somalia. PLoS One 3: e2081.

91. Chapman AD, Wieczorek J (2006) Guide to best practices for georeferencing. Copenhagen: Global Biodiversity Information Facility.

92. Guralnick RP, Wieczorek J, Beaman R, Hijmans RJ (2006) BioGeomancer: automated georeferencing to map the world's biodiversity data. PLoS Biol 4: e381.

93. Wieczorek J, Guo Q, Hijmans RJ (2004) The point-radius method for georeferencing locality descriptions and calculating associated uncertainty. Int J Geogr Inf Sci 18: 745-767.

94. O'Meara WP, Collins WE, McKenzie FE (2007) Parasite prevalence: a static measure of dynamic infections. Am J Trop Med Hyg 77: 246-249.

95. Wongsrichanalai C, Barcus MJ, Muth S, Sutamihardja A, Wernsdorfer WH (2007) A review of malaria diagnostic tools: microscopy and rapid diagnostic test (RDT). Am J Trop Med Hyg 77: 119-127.

96. Zurovac D, Midia B, Ochola SA, English M, Snow RW (2006) Microscopy and outpatient malaria case management among older children and adults in Kenya. Trop Med Int Health 11: 432-440.

97. Fogg C, Twesigye R, Batwala V, Piola P, Nabasumba C, et al. (2008) Assessment of three new parasite lactate dehydrogenase (pan-pLDH) tests for diagnosis of uncomplicated malaria. Trans R Soc Trop Med Hyg 102: 25-31.

98. Metzger WG, Vivas-Martinez S, Rodriguez I, Goncalves J, Bongard E, et al. (2008) Malaria diagnosis under field conditions in the Venezuelan Amazon. Trans R Soc Trop Med Hyg 102: 20-24.

99. Hay SI, Omumbo JA, Craig MH, Snow RW (2000) Earth observation, geographic information systems and *Plasmodium falciparum* malaria in sub-Saharan Africa. Adv Parasitol 47: 173-215.

100. Hay SI, Snow RW, Rogers DJ (1998) Predicting malaria seasons in Kenya using multitemporal meteorological satellite sensor data. Trans R Soc Trop Med Hyg 92: 12-20.

101. Hay SI, Snow RW, Rogers DJ (1998) From predicting mosquito habitat to malaria seasons using remotely sensed data: Practice, problems and perspectives. Parasitol Today 14: 306-313.

102. Snow RW, Guerra CA, Noor AM, Myint HY, Hay SI (2005) The global distribution of clinical episodes of *Plasmodium falciparum* malaria. Nature 434: 214-217.

103. Price RN, Tjitra E, Guerra CA, Yeung S, White NJ, et al. (2007) Vivax malaria: neglected and not benign. Am J Trop Med Hyg 77: 79-87.

104. Tjitra E, Anstey NM, Sugiarto P, Warikar N, Kenangalem E, et al. (2008) Multidrug-resistant *Plasmodium vivax* associated with severe and fatal malaria: a prospective study in Papua, Indonesia. PLoS Med 5: e128.

105. Rogerson SJ, Carter R (2008) Severe vivax malaria: newly recognised or rediscovered. PLoS Med 5: e136.

106. Genton B, D'Acremont V, Rare L, Baea K, Reeder JC, et al. (2008) *Plasmodium vivax* and mixed infections are associated with severe malaria in children: a prospective cohort study from Papua New Guinea. PLoS Med 5: e127.

107. Rowe AK, Rowe SY, Snow RW, Korenromp EL, Schellenberg JRA, et al. (2006) The burden of malaria mortality among African children in the year 2000. Int J Epidemiol 35: 691-704.

108. Snow RW, Craig M, Deichmann U, Marsh K (1999) Estimating mortality, morbidity and disability due to malaria among Africa's non-pregnant population. Bull World Health Organ 77: 624-640.

109. G.F.T.A.M. (2007) The Global Fund. Who we are what we do. Geneva: The Global Fund to Fight AIDS, Tuberculosis and Malaria (GFTAM). 36 p.

110. Grabowsky M (2008) The billion-dollar malaria moment. Nature 451: 1051-1052.

111. Snow RW, Guerra CA, Mutheu JJ, Hay SI (2008) International funding for malaria control in relation to populations at risk of stable *Plasmodium falciparum* transmission. PLoS Med 5: e142.

112. Tanner M, de Savigny D (2008) Malaria eradication back on the table. Bull World Health Organ 86: 82.

113. Greenwood BM, Fidock DA, Kyle DE, Kappe SH, Alonso PL, et al. (2008) Malaria: progress, perils, and prospects for eradication. J Clin Investig 118: 1266-1276.

114. R.B.M.P. (2008) The global malaria action plan for a malaria free world. Geneva, Switzerland: Roll Back Malaria Partnership (R.B.M.P), World Health Organization. 274 p.

**Hình 1**

Giới hạn không gian của nguy cơ sốt rét *P. falciparum* được định nghĩa theo *Pf*API có bổ sung ý kiến chuyên gia, các mặt nạ về nhiệt độ và độ khô hạn.

Các vùng được chia thành nguy cơ ổn định (màu đỏ sẫm, với *Pf*API ≥0,1 trên 1000 người pa), không ổn định (màu xám vừa, với *Pf*API <0,1 trên 1000 người pa) và không có nguy cơ (màu xám nhạt, với *Pf*API=0 trên 1000 người pa) [17-19]. Các điều tra cộng đồng về tỷ lệ nhiễm *P. falciparum* được thực hiện trong khoảng thời gian từ ngày 1 tháng Giêng năm 1985 đến ngày 31 tháng Bảy năm 2008 được vẽ đồ thị. Trong số 8.938 điều tra thu thập được, 7.953 đạt yêu cầu đưa vào vào mô hình hóa (xem phần Phương pháp luận và Nghi thức S1.2) và được trình bày. Dữ liệu điều tra được chuẩn hóa theo tuổi [51] (*Pf*PR_2-10_) và được trình bày ở dạng liên tục vàng sang đỏ của 0-100% (xem chú giải bản đồ). Các đường chấm chấm tách biệt ra các vùng châu Mỹ; châu Phi+; và Trung và TDNA.

doi:10.1371/journal.pmed.1000048.g001

**Hình 2**. Lược đồ tổng quát các quy trình và phương pháp lập bản đồ.

Các hình thoi xanh biểu thị dữ liệu đầu vào. Các ô màu cam biểu thị các quy trình thí nghiệm và mô hình; S1, Nghi thức S1; S2, Nghi thức S2; S3, Nghi thức S3; S4, Nghi thức S4. Các chấm màu xanh lá biểu thị dữ liệu đầu vào; các đường chấm chấm biểu thị dữ liệu đầu ra trung gian, các đường liền biểu thị dữ liệu đầu ra sau cùng. U, đô thị; PU, gần đô thị; và R, nông thôn.

doi:10.1371/journal.pmed.1000048.g002

**Hình 3**. Phân bố không gian mức độ lưu hành sốt rét *P. falciparum*.

Dữ liệu là các ước lượng giá trị điểm kiểu địa thống kê dựa trên mô hình của giá trí trung bình *Pf*PR_2-10_ trong năm áp dụng cho năm 2007 nằm trong vùng giới hạn không gian có sự lan truyền của sốt rét *P. falciparum*, được trình bày ở dạng liên tục vàng sang đỏ của 0-100% (xem chú giải bản đồ). Phần còn lại của các vùng trên cạn được xác định là có nguy cơ không ổn định (màu xám vừa, nơi có *Pf*API <0,1 trên 1.000 người pa) hoặc không có nguy cơ (màu xám nhạt, nơi có *Pf*API=0 trên 1.000 người pa) [17-19].

doi:10.1371/journal.pmed.1000048.g003

**Hình 4**. Phân bố không gian của các dự báo *Pf*PR_2-10_ của sốt rét *Plasmodium falciparum* được phân chia theo nhóm mức độ lưu hành

Chúng được phân loại thành nguy cơ thấp *Pf*PR_2-10_ ≤5% màu đỏ nhạt; nguy cơ trung bình *Pf*PR_2-10_ từ >5% đến <40%, màu đỏ vừa và nguy cơ cao *Pf*PR_2-10_ ≥40% màu đỏ sậm. Bản đồ biểu thị nhóm mà *Pf*PR_2-10_ có xác suất tiên đoán cao nhất. Phần còn lại của các vùng trên cạn được phân chia thành nguy cơ không ổn định (các vùng màu xám vừa, với *Pf*API <0,1 trên 1.000 người pa) hoặc không có nguy cơ (màu xám nhạt) [17-19].

doi:10.1371/journal.pmed.1000048.g004

**Hình 5**. Các bản đồ mức độ không chắc chắc của mô hình

(A) Xác xuất *Pf*PR_2-10_ thuộc nhóm mà nó được gán vào được lập bản đồ và biểu thị thành thang màu liên tục từ vàng đến xanh dương của khoảng . Bất kỳ giá trị nào trên là tốt hơn định vị ngẫu nhiên. Phần còn lại của vùng trên cạn được chia ra thành nguy cơ không ổn định (các vùng có màu xám vừa, với *Pf*API <0,1 trên 1.000 người pa) hoặc không có nguy cơ (màu xám nhạt) [17,18,19].

(B) Chỉ số mức độ không chắc chắn lấy trọng số theo dân số. Chỉ số này biểu thị tầm quan trọng tương đối của mức độ không chắc chắn được đánh giá bằng logarit của mật độ dân số (Nghi thức S2.3) và nghịch đảo của xác suất phép gán lớp đúng, được lấy tỷ lệ lại 0-1. Chỉ số này được trình bày cho cấp mức độ lưu hành *Pf*PR_2-10_ có xác suất cao nhất. Các vùng nguy cơ không ổn định và không có nguy cơ tương tự như (A).

doi:10.1371/journal.pmed.1000048.g005

**Hình 6**. Các đồ thị kiểm chứng mô hình

(A) Biểu đồ phân tán của các giá trị điểm thực và giá trị tiên đoán của *Pf*PR_2-10_.

(B) Mẫu semi-variogram trong phần dư Pearson của mô hình được chuẩn hóa tại các điểm trễ rời rạc (các vòng tròn) và được so sánh với bao Monte Carlo (các đường chấm chấm) biểu thị biên của các giá trị ngẫu nhiên khi không có tự tương quan không gian.

(C) Các đường cong đặc trưng hoạt động của bộ thu cho mỗi nhóm mức độ lưu hành *Pf*PR_2-10_ (đường đen *Pf*PR_2-10_ ≤5%, đường đỏ *Pf*PR_2-10_ từ >5% đến <40%, đường xanh lá *Pf*PR_2-10_ ≥40%) và thống kê diện tích dưới đường biểu diễn (AUC) liên quan.

(D) Đồ thị Xác suất-xác suất so sánh các ngưỡng xác suất được tiên đoán với phần trăm của các giá trị thực vượt quá các ngưỡng đấy. Hàng trên cùng và phần dưới phải vẽ đường 1:1 cũng được trình bày (các đường chấm chấm) để tham chiếu. Xem tài liệu chính về giải thích đầy đủ các quy trình kiểm chứng và giải thích các kết quả.

doi:10.1371/journal.pmed.1000048.g006

**Hình 7**. Biểu đồ tròn biểu thị dân số trong vùng có nguy cơ (PAR) sốt rét *P. falciparum* năm 2007.

Các biểu đồ thể hiện tỷ lệ của dân số sống trong mỗi nhóm mức độ lưu hành *Pf*PR_2-10_ tiên đoán đối với châu Mỹ, châu Phi+ và TDNA và toàn thế giới. Các biểu đồ được lấy tỷ lệ theo tổng dân số trong mỗi vùng và các thành phần được tô màu cho khớp với các mức độ lưu hành trình bày trong Hình 4.

doi:10.1371/journal.pmed.1000048.g007

**Hình 8**. Các đồ thị violin cho từng vùng thể hiện các phân bố tần số của dữ liệu *Pf*PR_2-10_.

(A) tất cả các năm, (B) năm 2007 và (C) là bề mặt 2007 tiên đoán. Chiều rộng của từng đồ thị minh họa tần số tương đối của các giá trị *Pf*PR_2-10_ khác nhau trong từng vùng. Phần nền được tô màu khớp với các lớp mức độ lưu hành được trình bày trên Hình 4. Thanh trung tâm màu đen biểu thị biên của khoảng tứ phân vị và các vòng tròn màu trắng chỉ các giá trị giữa (xem tài liệu chính).

doi:10.1371/journal.pmed.1000048.g008

**Bảng 1**. Tóm tắt các thống kê kiểm chứng cho việc dự báo *Pf*PR_2-10_ liên tục theo vùng.

| **Số đo kiểm chứng** | **Châu Mỹ** | **Châu Phi+** | **TDNA** | **Thế giới** |
| --- | --- | --- | --- | --- |
|  |  |  |  |  |
| Sai số trung bình | 0.627 (20.401) | 0.805 (3.070) | 1.184 (9.203) | 0.912 (4.267) |
| Sai số tuyệt đối trung bình | 3.522 (114.639) | 11.023 (42.024) | 7.705 (59.860) | 9.750 (45.625) |
| Tương quan | 0.032 | 0.822 | 0.699 | 0.823 |

Trị trung bình của mỗi phân bố hậu nghiệm dự báo được dùng là ước lượng điểm của *Pf*PR_2-10_ để so sánh với các giá trị quan sát được. Các giá trị trong ngoặc đơn là phần trăm trị trung bình của vùng được biểu thị bằng giá trị sai số tương ứng. Xem tài liệu chính để biết giải thích đầy đủ về nguồn gốc của các thống kê này và giải thích các kết quả.

doi:10.1371/journal.pmed.1000048.t001

**Bảng 2**. Tóm tắt các thống kê kiểm chứng đối với việc dự báo nhóm mức độ lưu hành *Pf*PR_2-10_ theo vùng. Châu Phi, Yemen và Ả-rập Xê-út (châu Phi+) và Trung, Đông và Nam Á (TDNA). Xem tài liệu chính để biết giải thích đầy đủ về nguồn gốc của các thống kê này và giải thích các kết quả.

| **Số đo kiểm chứng** | **Châu Mỹ** | **Châu Phi+** | **TDNA** | **Thế giới** |
| --- | --- | --- | --- | --- |
|  |  |  |  |  |
| AUC (≤5%) | 0.452 | 0.927 | 0.885 | 0.915 |
| AUC (5% đến <40%) | 0.452 | 0.758 | 0.813 | 0.779 |
| AUC (≥40%) | - | 0.921 | 0.842 | 0.927 |
| % đúng tổng cộng | 80.000 | 70.621 | 69.874 | 70.750 |
| ≤5% bị phân loại thành ≥40% (%) | 0.000 | 0.188 | 0.418 | 0.250 |
| ≥40% bị phân loại thành ≤5% (%) | 0.000 | 0.377 | 2.092 | 0.875 |

Xem tài liệu chính để biết giải thích đầy đủ về nguồn gốc của các thống kê này và giải thích các kết quả.

**Bảng 3**. Diện tích trong vùng có nguy cơ sốt rét *P. falciparum* trong năm 2007.

| **Vùng** | **Nguy cơ không ổn định** | **Nguy cơ ổn định** | ***Pf*PR_2-10_** ≤**5%** | ***Pf*PR_2-10_ >5 đến <40%** | ***Pf*PR_2-10_** ≥**40%** | **Tổng** |
| --- | --- | --- | --- | --- | --- | --- |
|  |  |  |  |  |  |  |
| Châu Mỹ | 2.13 | 6.03 | 6.03 | 0.00 | 0.00 | 8.17 |
| Châu Phi+ | 4.21 | 18.17 | 4.03 | 5.63 | 8.50 | 22.37 |
| TDNA | 3.40 | 5.53 | 4.72 | 0.69 | 0.11 | 8.93 |
| Thế giới | 9.74 | 29.72 | 14.79 | 6.32 | 8.61 | 39.46 |

Diện tích được tính bằng triệu km^2^. Vùng có nguy cơ không ổn định (Chỉ số mắc ký sinh trùng *P. falciparum* hàng năm (PfAPI) < 0,1 trên một nghìn người trên năm (pa) và nguy cơ ổn định (PfAPI ≥0.1 trên một nghìn người pa). Vùng có nguy cơ ổn định được chia nhỏ thành ba nhóm mức độ lưu hành *Pf*PR_2-10_ liên quan đến hoạt động kiểm soát [19] và được chuẩn hóa theo tuổi [51]. Châu Phi, Yemen và Ả-rập Xê-út (châu Phi+) và Trung, Đông và Nam Á (TDNA).

**Bảng 4**. Dân số trong vùng có nguy cơ (PAR) sốt rét *P. falciparum* năm 2007.

| Vùng | Nhóm tuối | Nguy cơ không  ổn định**^a^** | Nguy cơ ổn định**^a^** | *Pf*PR_2−10_  ≤ 5% | *Pf*PR_2−10_  > 5 đến < 40% | *Pf*PR_2−10_  ≥ 40% | Tổng |
| --- | --- | --- | --- | --- | --- | --- | --- |
| Các nước châu Mỹ | 0–4 | 5.62 | 4.51 | 4.51 | 0.00 | 0.00 | 10.14 |
|  | 5–14 | 10.66 | 8.66 | 8.66 | 0.00 | 0.00 | 19.32 |
|  | 15+ | 33.78 | 27.47 | 27.47 | 0.00 | 0.00 | 61.25 |
|  | Tổng | 50.06 | 40.64 | 40.64 | 0.00 | 0.00 | 90.71 |
| Châu Phi+ | 0–4 | 3.29 | 108.64 | 15.48 | 32.90 | 60.26 | 111.93 |
|  | 5–14 | 5.64 | 170.80 | 25.35 | 51.48 | 93.98 | 176.44 |
|  | 15+ | 12.95 | 377.17 | 73.67 | 112.45 | 191.05 | 390.12 |
|  | Tổng | 21.88 | 656.61 | 114.50 | 196.83 | 345.28 | 678.49 |
| TDNA | 0–4 | 99.69 | 73.70 | 64.98 | 8.10 | 0.62 | 173.38 |
|  | 5–14 | 195.40 | 143.20 | 126.26 | 15.67 | 1.27 | 338.60 |
|  | 15+ | 616.17 | 468.75 | 412.37 | 51.52 | 4.86 | 1,084.93 |
|  | Tổng | 911.26 | 685.65 | 603.61 | 75.29 | 6.75 | 1,596.91 |
| Thế giới | 0–4 | 108.60 | 186.85 | 84.97 | 41.00 | 60.88 | 301.31 |
|  | 5–14 | 211.70 | 322.66 | 160.26 | 67.15 | 95.25 | 560.40 |
|  | 15+ | 662.90 | 873.40 | 513.51 | 163.97 | 195.91 | 1,504.41 |
|  | Tổng | 983.20 | 1,382.91 | 758.75 | 272.13 | 352.04 | 2,366.11 |

Dân số được tính bằng đơn vị triệu. (Chỉ số mắc ký sinh trùng *P. falciparum* hàng năm (PfAPI) < 0,1 trên 1.000 người pa và nguy cơ ổn định (PfAPI ≥0.1 trên 1.000 người pa). Vùng có nguy cơ ổn định được chia nhỏ thành ba nhóm mức độ lưu hành *Pf*PR_2-10_ liên quan đến hoạt động kiểm soát [19] và được chuẩn hóa theo tuổi [51]. Châu Phi, Yemen và Ả-rập Xê-út (châu Phi+) và Trung, Đông và Nam Á (TDNA). Đối với mỗi khu vực, PAR được chia nhỏ thành các nhóm tuổi 0-4, 5-14 và trên 15.

**^a^** Cần nhớ là do hạn chế về năng lực máy tính, buộc phải lấy mẫu lại ở độ phân giải không gian 5x5 km, sẽ dẫn đến hệ quả không tránh khỏi là có những thay đổi rất nhỏ về tổng dân số toàn cầu trong vùng có nguy cơ được ước lượng trước đó ở độ phân giải 1x1 km [18].

doi:10.1371/journal.pmed.1000048.t004
