# Supplementary material for: A World Malaria Map: Plasmodium falciparum Endemicity in 2007
Source: PLoS Med. 2009 Mar 24;6(3):e1000048. doi: 10.1371/journal.pmed.1000048 (PMC2659708; doi:10.1371/journal.pmed.1000048)
Supplement: Supplementary file 5 [file pmed.1000048.sd005.doc]

**Un Mapa Mundial de Malaria: Endemicidad de *Plasmodium falciparum* en el 2007**

Simon I. Hay^1,2*^, Carlos A. Guerra^1,2^, Peter W. Gething^2,3^, Anand P. Patil^2^, Andrew J. Tatem^1,2,4,5^, Abdisalan M. Noor^1,6^, Caroline W. Kabaria^1^, Bui H. Manh^7^, Iqbal R.F. Elyazar^8^, Simon Brooker^1,9^, David L. Smith^5,10^, Rana A. Moyeed^11^, Robert W. Snow^1,6^

1. Malaria Public Health and Epidemiology Group, Centre for Geographic Medicine, Medical Research Institute (KEMRI) – Univ. Oxford - Wellcome Trust Collaborative Programme, Nairobi, Kenia

2. Spatial Ecology and Epidemiology Group, Tinbergen Building, Department of Zoology, Universidad de Oxford, South Parks Road, Oxford, Reino Unido

3. Centre for Geographical Health Research, School of Geography, Universidad de Southampton, Highfield, Southampton, Reino Unido

4. Department of Geography, Universidad de Florida, Gainesville, Florida, Estados Unidos

5. Emerging Pathogens Institute, University de Florida, Gainesville, Florida, Estados Unidos

6. Centre for Tropical Medicine, Nuffield Department of Clinical Medicine, Universidad de Oxford, Centre for Clinical Vaccinology and Tropical Medicine (CCVTM), Oxford, Reino Unido

7. Oxford University Clinical Research Unit, Bach Mai Hospital, National Institute of Infectious and Tropical Diseases, Ha Noi, Vietnam

8. Eijkman-Oxford Clinical Research Unit, Jakarta, Indonesia

9. Department of Infectious and Tropical Diseases, London School of Hygiene and Tropical Medicine, Reino Unido

10. Department of Zoology, University de Florida, Gainesville, Florida, Estados Unidos

11. School of Mathematics and Statistics, Universidad de Plymouth, Drake Circus, Plymouth, Devon, Reino Unido

**Resumen**

Introducción

La asignación eficiente de recursos para el control de la malaria requiere de un entendimiento detallado de la distribución espacial del riesgo actual de malaria. Han pasado exactamente cuarenta años desde que el último mapa global de niveles endémicos de malaria fue publicado. Este artículo describe el desarrollo de un nuevo mapa mundial de endemicidad de malaria por *Plasmodium falciparum* para el año 2007.

Metodología y Hallazgos

Se identificó un total de 8 938 encuestas de prevalencia parasitaria de *P. falciparum* (*Pf*PR) utilizando exhaustivos criterios de búsqueda. De éstas, 7 953 pasaron pruebas estrictas de fidelidad de datos para ser incluidas en una base de datos global de *Pf*PR, estandarizadas al rango de 2 a 10 años de edad para ser utilizados en el mapeo de endemicidad. Se utilizó un procedimiento geo-estadístico en base a modelos para crear una superficie continua de endemicidad de malaria dentro de límites anteriormente establecidos de transmisión estable de *P. falciparum*. Estos procedimientos fueron implementados dentro de un marco estadístico Bayesiano de tal manera que la incertidumbre de estas predicciones pueda ser evaluada consistentemente. Esta incertidumbre se reflejó en la probabilidad de predecir correctamente una de tres clases de endemicidad previamente estratificadas como una guía informativa para el control de malaria. A continuación se estimaron poblaciones en riesgo, tomando en cuenta la influencia de la urbanización en África sobre la transmisión de malaria, en base a superficies de población humana en el 2007.

De los 1 380 millones de personas en riesgo de transmisión estable de malaria por *P. falciparum*, 690 millones vivían en el Asia Central y el Sudeste Asiático (CSE Asia), 660 millones en África, Yemen y Arabia Saudita (África+) y 40 millones en América. Todos aquellos expuestos a riesgo de transmisión estable en América correspondían a la clase endémica más baja (*Pf*PR2-10 ≤ 5%). La gran mayoría (88%) de aquellos que vivían en riesgo de transmisión estable en CSE Asia también se encontraron en esta clase de baja endemicidad, 11% en la clase endémica intermedia (*Pf*PR2-10 >5 a <40%) y 1% en alta endemicidad (*Pf*PR2-10 ≥ 40%). En la región África+ esta alta endemicidad es común y en ella habitan 350 millones de personas. En su gran mayoría, el resto vive en riesgo intermedio (200 millones) con una fracción menor (110 millones) que vive en bajo riesgo estable.

Conclusiones e Importancia

Niveles altos de endemicidad de malaria por *P. falciparum* son comunes en África. En América se encuentran más uniformemente niveles bajos. La baja endemicidad es también común en CSE Asia, donde aún se encuentran pequeños focos de endemicidad intermedia y, muy raramente, alta. Por lo tanto, existen oportunidades significativas de control de malaria en África y de su eliminación en América y CSE Asia. Este mapa global de endemicidad de malaria por *P. falciparum* en el 2007 es el primero de una serie mediante la cual será posible monitorear y evaluar el progreso del proceso de intervención contra la malaria.

**Introducción**

Los mapas son herramientas esenciales en todos los aspectos de la coordinación del control de malaria [1]. En un ambiente internacional de políticas donde se incita a la comunidad de control de malaria a reconsiderar la posibilidad de eliminación [2,3,4], la cartografía de malaria se convertirá en una herramienta cada vez más importante para la planificación, implementación y evaluación del impacto de intervenciones contra la malaria a nivel mundial. El último mapa global de endemicidad de *Plasmodium falciparum* fue publicado en 1968 [5]. Como todos los mapas de distribución global de malaria previamente publicados [6,7,8,9,10] y, en gran medida, aquellos que vinieron después [11,12,13,14,15,16], este mapa (i) careció de una descripción completa de los datos utilizados en su generación; (ii) definió “riesgo” utilizando reglas de opinión de expertos subjetivas y pobremente explicadas y (iii) no ofreció una cuantificación de la certeza de sus predicciones. En este artículo describimos la generación de un nuevo mapa global de endemicidad de malaria que supere estas deficiencias.

Alcance Geográfico del Modelamiento

Los límites espaciales globales de malaria por *P. falciparum* fueron mapeados recientemente triangulando datos de incidencia de casos reportados a nivel nacional, información proveniente de otras fuentes de inteligencia médica y reglas biológicas de exclusión de transmisión, derivados de los extremos de temperatura y aridez para el desarrollo de especies dominantes del vector *Anopheles* [17,18]. El resultado de este ejercicio fue una estratificación del mundo en tres clases maláricas: sin riesgo, riesgo inestable (Incidencia Parasitaria Anual de *P. falciparum* (*Pf*API) <0.1 por 1000 habitantes por año (pa)) y riesgo estable (*Pf*API ≥ 0.1 por 1000 habitantes pa) para el año 2007. Estas clases se muestran en la Figura 1. La clasificación de *Pf*API en estable-inestable deriva de una revisión de las razones estadísticas, logísticas, programáticas y pragmáticas que determinaron los niveles de *Pf*API utilizados para definir puntos de acción durante la campaña global de erradicación de la malaria [19,20,21].

El mapeo descrito aquí extiende considerablemente este esfuerzo. La más grande base de datos a nivel global de encuestas malariométricas es usada para predecir valores de prevalencia de malaria por *P. falciparum* en cada punto dentro de los límites estables de transmisión para obtener una superficie continua de endemicidad de *P. falciparum*. Para facilitar el proceso los límites espaciales antes descritos requirieron de un re-muestreo a 5x5 km utilizando ArcView GIS 3.2 (ESRI, 1999) debido a que las técnicas de mapeo adoptadas, y descritas a continuación, no podían ser implementadas a la resolución espacial original de 1x1 km a escala global dada la elevada demanda computacional.

Métodos Recientes para el Mapeo de Endemicidad de Malaria

Existe una serie de metodologías para la producción de mapas de endemicidad utilizando datos de encuestas malariométricas, todas las cuales requieren del uso de un modelo para predecir valores de endemicidad donde no están disponibles datos de encuestas parasitarias [22,23,24,25,26]. Los mapas resultantes de dichos modelos tienen una incertidumbre en las predicciones y la cuantificación de la misma es una prioridad en el mapeo de enfermedades.

Algunos estudios recientes han adoptado un marco predictivo conocido como geo-estadística en base a modelos (MBG) [27] para la predicción espacial de endemicidad de malaria [28,29,30,31,32,33] y prevalencia de otras enfermedades transmitidas por vectores o de huésped intermediario [34,35,36,37,38]. MBG brinda una interpretación estadística formal de herramientas geo-estadísticas clásicas para predicción espacial [39,40,41] y permite la incorporación de métodos Bayesianos de inferencia estadística [42,43]. La principal ventaja de MBG para el mapeo de enfermedades es el manejo riguroso de la incertidumbre introducida durante distintas etapas del proceso de modelamiento [27]. Al modelar la interacción de estas distintas fuentes de incertidumbre se genera una distribución probabilística para cada predicción, la misma que se puede resumir para dar medidas certeras de confianza alrededor de los valores predichos. El mapa resultante representará, por lo tanto, un hito contemporáneo de endemicidad global de malaria, usando técnicas de MBG para evaluar la confianza en las predicciones y ofrecer a quienes utilizan el mapa, un estimativo claro de la fidelidad de las predicciones [36].

Un principio geo-estadístico es que una predicción espacial se vuelve cada vez más incierta mientras la densidad de y la proximidad a datos cercanos decrece. Cuando los datos son recolectados a distintos tiempos y en distintas ubicaciones, este principio es tan aplicable en el tiempo como en el espacio. Los ejemplos de estudios epidemiológicos que incorporan tiempo en geo-estadística espacial son raros [44,45,46,47], pero en este estudio se desarrolló un marco geo-estadístico espacio-temporal. El incorporar la dimensión del tiempo permite una comparación directa de este producto referencial con futuros mapas. El mapa, por lo tanto, representará un marco geográfico explícito para el monitoreo y la evaluación del impacto de la comunidad de control de la malaria sobre la malaria por *P. falciparum* a nivel global.

**Métodos**

Perfil Analítico

El objetivo de estos análisis es el usar una base de datos actualizada de encuestas de prevalencia de *P. falciparum* (*Pf*PR) para generar una superficie continua y global de endemicidad de malaria por *P. falciparum* para el año 2007, utilizando métodos confiables y reproducibles, documentando a la vez la incertidumbre asociada a las predicciones.

Los principales pasos utilizados para definir el mapa global continuo de prevalencia de *P. falciparum* dentro de nuestro marco analítico están delineados en la Figura 2. Primero, fue necesario recopilar y procesar los datos de *Pf*PR con el fin de crear una base de datos geográficamente extensa y geo-posicionada en forma consistente para ser utilizada en el mapeo y en la evaluación de co-variables ambientales (Protocolo S1) y de la influencia de patrones de urbanización [48,49,50] (Protocolo S2). Segundo, esta base de datos de *Pf*PR depurada fue utilizada para generar una superficie de prevalencia de malaria continua, estandarizada a un grupo etáreo y corregida por los efectos de urbanización, en base a MBG en un marco estadístico Bayesiano (Protocolo S3). Tercero, se implementaron exhaustivos procedimientos de validación para evaluar la certeza de las predicciones de endemicidad y las medidas de incertidumbre (Protocolo S4). Finalmente, se obtuvieron estimativos globales de poblaciones en riesgo (PAR) de malaria por *P. falciparum*, los cuales fueron presentados a nivel regional, estratificados por grupo etáreo.

Creando una Base de Datos Global de Prevalencia de *P. falciparum*

De todas las potenciales medidas disponibles de endemicidad de malaria, se escogió la prevalencia parasitaria (la proporción de individuos muestreados que presentan parásitos en sangre periférica) como la base para el mapeo, debido a su ubicuidad global [18] y su sensibilidad en gran parte del espectro de transmisión de *P. falciparum* [19]. Se ha sugerido una categorización del espectro de endemicidad de malaria en el rango de edad más informativo, desde el punto de vista epidemiológico, de dos a diez años [51], guiado por el impacto potencial de endemicidad de malaria utilizando la intervención anti-malárica más ampliamente utilizada - mosquiteros impregnados con insecticida (ITNs) [19]. El nivel endémico definido por *Pf*PR en el grupo de dos a diez años (*Pf*PR2-10) corresponde a ≤5%. Debajo de este punto las encuestas de *Pf*PR requieren de tamaños de muestra prohibitivamente grandes para medir endemicidad con precisión, favoreciendo, por lo tanto, medidas malariométricas basadas en vigilancia epidemiológica [52,53,54]. Consideramos transmisión intermedia estable a *Pf*PR2-10 >5% a <40% debido a que una serie de modelos matemáticos predicen que se puede lograr interrupción de la transmisión con cobertura universal de ITNs en todas las áreas con *Pf*PR2-10 <40% [19,55]. A pesar de estar sujeto a incertidumbre debido al comportamiento y las características biológicas de los vectores *Anopheles* dominantes [56], el nivel de *Pf*PR2-10 <40% es considerado un punto de referencia conservador debido a que rara vez se distribuyen ITNs independientemente de otras intervenciones que reducirán aún más la transmisión. Las áreas de transmisión estable alta, donde intervenciones mixtas deben considerarse si se quiere alcanzar, de ser posible, la interrupción de transmisión, son aquellas donde la prevalencia está por encima de este nivel: *Pf*PR2-10 ≥40%. Esta clasificación de la transmisión de malaria se la utiliza como una guía para la interpretación de la endemicidad predicha y representa un alejamiento de la clasificación endémica tradicional [57] que se ha demostrado que no corresponde adecuadamente, en la mayor parte de modelos, a oportunidades de control y eliminación [19,55].

El proceso de identificación, recopilación y geo-posicionamiento de encuestas de prevalencia parasitaria en comunidades realizadas desde 1985 ha sido descrito anteriormente [58]. La búsqueda de datos de *Pf*PR es una actividad constante del Malaria Atlas Project (MAP, http:www.map.ox.ac.uk) y fue completada, para esta versión 2007 del mapa global de endemicidad, el 31 de julio del 2008 (Protocolo S1.1). Un total de 8 938 encuestas transversales fueron recopiladas de 78 de los 87 países endémicos para *P. falciparum* (*Pf*MECs) [18]. Los países no representados en la base de datos fueron Bangladesh, Belice, Bután, Djibouti, Guyana, Irán, Kyrgyzstan, Panamá y Republica Dominicana.

Después de utilizar seis niveles de exclusión (excluyendo encuestas representando grandes (>100 Km2) y pequeños (>25 km2) polígonos [58]; excluyendo aquellas encuestas que no pudieron ser geo-posicionadas o fueron geo-posicionadas sin precisión; y excluyendo aquellas que no pudieron ser desagregadas temporalmente en encuestas independientes o en las que la fecha de la encuesta se desconocía) quedaron 7 991 encuestas de *Pf*PR (Protocolo S1 y Figura S1.2).

Todos los datos de *Pf*PR fueron luego estandarizados al grupo etáreo de dos a diez años utilizando un algoritmo basado en modelos de conversión catalítica, adaptados a la malaria por Pull y Grab [59]. Dicho algoritmo fue el más apropiado de un grupo de procedimientos de estandarización y fue descrito en detalle en otra publicación [51] (Protocolo S1.3).

La base de datos final fue estratificada en tres regiones principales (Figura 1): América; África, Yemen y Arabia Saudita (África+); y Asia Central y el Sudeste Asiático (CSE Asia) (Protocolo S1.4). Esta división permitió considerar separadamente a estas regiones biogeográfica, entomológica y epidemiológicamente distintas [8,16] y, al mismo tiempo, contar con suficientes datos en cada región necesarios para análisis significativos. Esta división global fue además sustentada al evidenciar una estructura espacial distinta de los datos de *Pf*PR2-10 en cada región, ilustrada por sus semi-variogramas (Protocolo S1, Figura S1.1).

Se desarrollaron metodologías específicas relacionadas a la transmisión de malaria para el mapeo de superficies urbanas, semi-urbanas y rurales, las cuales ya han sido descritas en detalle [50] (Protocolo S2). Brevemente, las extensiones urbanas (UE) definidas por el Global Rural Urban Mapping Project (GRUMP UE), versión alfa [60,61] fueron identificadas a una resolución espacial de 1x1 km (Protocolo S2.1) [50]. Dentro de estas superficies, aquellas áreas con densidades poblacionales mayores a 1 000 personas por km2 de acuerdo al Gridded Population of the World versión 3 (GPW3) [60,61] fueron mapeadas [48]. Luego, todas las encuestas parasitarias fueron catalogadas como urbanas (Gridded Population of the World versión 3 ≥1 000 por km2 dentro de GRUMP UE), semi-urbanas (Gridded Population of the World versión 3 <1 000 por km2 dentro de GRUMP UE) o rurales (fuera de GRUMP UE) (Protocolo S2.2).

Externos estadísticos extremos en los datos de *Pf*PR2-10 rurales fueron identificados utilizando un filtro geo-estadístico (Protocolo S1.5). Este proceso se sirvió de semi-variogramas estadísticos para determinar si cada punto difería significativamente de puntos vecinos dadas las distancias que los separaban y los patrones regionales de variación espacial. Este procedimiento identificó 38 registros *Pf*PR2-10 rurales, los mismos que fueron descartados previo al modelamiento. De ser solicitados, los detalles de estas encuestas están disponibles.

La base de datos definitiva de *Pf*PR2-10 utilizada (n=7 953) está ilustrada en la Figura 1. La Tabla S1.2 en el Protocolo S1 presenta los atributos de esta base de datos final; junto con un gráfico de la mediana de *Pf*PR2-10 por año para el período de observación (Protocolo S1, Figura S1.3), el mismo que muestra que el tiempo fue una importante fuente de variación a ser incluida en el modelo MBG. Exploraciones preliminares similares de la relación de estos datos con una serie de co-variables climáticas [62] y ambientales derivadas de sensores remotos [63] no mostraron relaciones significativas (Protocolo S1.7, Figura S1 (S1.5) y S1 (S1.6)), brindando sustento al enfoque predominantemente uni-variable de los análisis.

Existe el concepto errado de que las encuestas malariométricas son solamente llevadas a cabo en áreas de prevalencia elevada. De hecho, la creciente tendencia a realizar encuestas nacionales representativas de todas las regiones del país y la confirmación de ausencia de *P. falciparum* durante muestreos de *P. vivax*, resultaron en muchos valores de prevalencia cero para *P. falciparum* en la base de datos. Un total de 119 de 261 encuestas reportaron valores de cero en América, 1 010 de 5 307 encuestas reportaron valores de cero en África+ y 775 de 2 385 encuestas reportaron valores de cero en CSE Asia (Figura 1).

Detalles Estadísticos

Los algoritmos geo-estadísticos generan mapas continuos al predecir valores en ubicaciones no muestreadas utilizando combinaciones lineales de los datos disponibles. En el mapeo descrito en este estudio la confianza vinculada a cada predicción de *Pf*PR2-10 en una ubicación no muestreada dada dependerá de (i) la distribución de los datos obtenidos alrededor de esa ubicación (la densidad espacial de los datos observados), (ii) la magnitud con que *Pf*PR2-10 varía en el espacio (la heterogeneidad espacial de los datos), y (iii) el número de personas muestreadas en cada encuesta (la precisión de los datos de las encuestas). Un método MBG [27] fue implementado dentro de un marco estadístico Bayesiano para incorporar estos factores en la generación de mapas continuos de *Pf*PR2-10 (Protocolo S3). Debido a que los datos fueron recolectados a distintos tiempos durante el período de estudio 1985-2008, fue importante extender la metodología únicamente espacial a un marco espacio-temporal que tome en cuenta la densidad y heterogeneidad de los datos tanto en tiempo como en espacio. El algoritmo de estandarización por edad fue integrado como un sub-modelo en este marco para permitir que los errores inherentes en este proceso sean estimados y propagados dentro de la fase de MBG (Protocolo S3).

Para cada región, un modelo geo-estadístico Bayesiano fue creado en el cual el valor *Pf*PR2-10 en el 2007, , en cada ubicación fue modelado como una transformación de un campo estructurado espacio-temporalmente superimpuesto con una variación no estructurada (aleatoria) . El número de respuestas positivas de *P. falciparum* de una muestra total de en cada ubicación fue modelado como una variable binomial condicionalmente independiente dado el valor *Pf*PR2-10 no observado [36]. El componente espacio-temporal fue representado por un proceso Gaussiano estacionario con media  y covarianza definida por una versión espacialmente anisotrópica de la función espacio-temporal de covarianza propuesta por Stein [64]. Esta función de covarianza de Stein fue modificada para permitir que el modelo tiempo-marginal incluya un componente periódico de longitud de onda de 12 meses, lo que permitió la capacidad de modelar los efectos estacionales en la estructura de covarianza temporal observada. Estos efectos surgen cuando estudios realizados en distintos años pero durante meses similares tienen una tendencia a ser más parecidos entre sí de lo que fuera esperado en ausencia de efectos estacionales. La media  fue modelada como una función lineal del tiempo, , siempre y cuando la ubicación predicha fuera urbana o semi-urbana (dada por las variables indicadoras y , , respectivamente) y no rural: . Cada encuesta fue referenciada temporalmente utilizando el punto medio (en años decimales) entre los meses de inicio y fin registrados. El estatus de urbano, semi-urbano o rural fue asignado a cada ubicación predicha utilizando la superficie urbana modificada de GRUMP descrita anteriormente (Protocolo S2.2), degradada a una resolución espacial de 5x5 km. El componente no estructurado fue representado como Gaussiano con media cero y varianza . Se utilizó el método Markov Chain Monte Carlo (MCMC) para implementar inferencia Bayesiana y generar muestras de la distribución posterior de: el campo Gaussiano en cada ubicación de los datos; los parámetros no observados , *,* , y como se indicó arriba, y parámetros no observados adicionales que definían la estructura y anisotropía de la función de covarianza espacio-temporal exponencial (Protocolo S3.4). Las distancias entre ubicaciones fueron calculadas utilizando distancia esférica para incorporar el efecto de la curvatura de la Tierra, la misma que gana importancia a escala regional. Se generaron muestras de la media anual de la distribución posterior del 2007 de en cada ubicación predicha. Para cada muestra de la unión posterior se realizaron predicciones usando simulación espacio-temporal condicional para los 12 meses del 2007 [44,65]. Estas predicciones fueron hechas en puntos sobre una red espacial regular de 5x5 km dentro de los límites espaciales de transmisión estable de *P. falciparum*. Los productos del modelo consistieron, por lo tanto, en muestras de la distribución posterior predicha de la media anual de *Pf*PR2-10 en el 2007 en cada ubicación de la red espacial, las mismas que fueron usadas para generar estimativos en puntos (correspondientes a la media de cada grupo de muestras posteriores), probabilidades de correspondencia a clase endémica y estimativos de variación estándar (Protocolo S3.4). El Protocolo S3.5 presenta una descripción más detallada de cómo los productos geo-estadísticos fueron utilizados para generar los varios mapas aquí descritos.

Validación del Modelo

Fue esencial determinar la verosimilitud de los mapas generados y algunos métodos descriptivos no triviales fueron implementados (Protocolo S4). La capacidad del modelo de predecir valores de *Pf*PR2-10 y la clase endémica más probable fueron evaluadas utilizando un procedimiento de ‘hold-out’. Un ‘set’ de validación fue generado seleccionando diez por ciento de los datos (n=800) en base a un muestreo aleatorio estratificado espacialmente desagregado; estos datos fueron luego retirados de la base de datos (Protocolo S4.1). Después, el modelo fue implementado nuevamente utilizando los 7 153 datos restantes para generar distribuciones posteriores predictivas de *Pf*PR2-10 para compararlas con valores conocidos en las ubicaciones de los 800 datos retirados. A diferencia del modelo principal, en el cual las medias anuales fueron predichas para el 2007, el modelo de validación predijo *Pf*PR2-10 para el mes correspondiente al punto medio de cada encuesta retirada, para proveer valores temporalmente comparables. Dado el tamaño de la base de datos, se consideró que un solo set de validación era suficiente para generar estadísticas de validación con el nivel de precisión requerido.

La habilidad de predecir valores conocidos de *Pf*PR2-10 fue resumida utilizando el error medio como medida de sesgo, error medio absoluto como medida de precisión y el coeficiente de correlación como medida de asociación lineal [44,66]. Estas estadísticas fueron presentadas como valores absolutos y como una proporción de la media de *Pf*PR2-10 del set de validación en cada región. La habilidad de predecir membresía a un clase endémica fue evaluada utilizando la estadística área bajo la curva (AUC) de la característica de receptor-observador (ROC), la misma que grafica sensibilidad contra la 1-especificidad para cada clase endémica [34,67]. También se implementó un procedimiento [44,68] para evaluar la magnitud con que las distribuciones posteriores predichas en cada ubicación brindaban una medida de incertidumbre adecuada. Este procedimiento permitió que la probabilidad asignada a valores de *Pf*PR2-10 predichos en cada ubicación pronosticada sea comparada con las probabilidades observadas correspondientes dentro de cada región. Más detalles de este procedimiento están disponibles en el Protocolo S4.2.

Se visualizaron las distribuciones de *Pf*PR2-10 tanto de los datos originales como de la superficie predicha utilizando gráficos de violín [69]. Estos gráficos ilustran una aproximación regularizada de la distribución de frecuencia de *Pf*PR2-10 para cada región montada sobre una barra central que muestra valores de mediana y rango inter-cuartil. Los gráficos se generaron utilizando datos de *Pf*PR2-10 para todos los años y para el 2007 en la base de datos, y un gráfico adicional se generó utilizando valores predichos para cada ubicación de la superficie de *Pf*PR2-10 producida para el 2007.

Estimando Densidad Poblacional en el 2007

La versión alfa de GRUMP ofrece valores de conteo y de densidad poblacional a una resolución espacial de 1x1 km para los años 1990, 1995 y 2000, ajustados y no ajustados a las poblaciones nacionales estimadas por las Naciones Unidas [60,61] (Protocolo S2.3). Los valores de conteo poblacional del 2000 ‘ajustados’ fueron proyectados al 2007 aplicando valores de crecimiento inter-censal nacionales [70] por medio de métodos descritos anteriormente [71] (Protocolo S2.4). Estos valores de conteo poblacional fueron luego estratificados por grupo etáreo utilizando estructuras poblacionales por edad definidas por las Naciones Unidas [72] para el año 2005; de este modo se obtuvieron superficies poblacionales para 0-5 años, 5-14 años y 15 ó más años.

Los límites de los 87 países endémicos para *P. falciparum* fueron superpuestos sobre la superficie de clases endémicas (re-proyectada a una proyección equiareal) y las áreas correspondientes a cada clase endémica fueron calculadas utilizando ArcView GIS 3.2 (ESRI, 1999) (Protocolo S2.4). Se realizó la misma superposición sobre GRUMP [60,61] para obtener estimativos de poblaciones en riesgo de *P. falciparum*, ajustados a los efectos de urbanización, por clase endémica y grupo etáreo (Protocolo S2.4). Finalmente, estas superficies fueron combinadas con los mapas de incertidumbre para proveer de un índice de incertidumbre ajustado a la población (en otras palabras, el producto de la densidad poblacional en escala logarítmica y el inverso de la probabilidad de asignación correcta de clase).

**Resultados**

Precisión del Mapa Predicho de Endemicidad de Malaria por *P. falciparum*

La superficie continua predicha de endemicidad de malaria por *P. falciparum* se muestra en la Figura 3. La clase endémica, relacionada al control, para la cual la correspondencia es más probable, se muestra en la Figura 4. La probabilidad de predecir cada clase correctamente se ilustra en la Figura 5A. A continuación se presenta una descripción detallada de la precisión de las predicciones y de la variación regional de área en estos distintos niveles de riesgo estable y la PAR asociada. En el Protocolo S4.3 se presentan medidas alternativas de la incertidumbre de las predicciones.

Prediciendo Valores Puntuales de *Pf*PR2-10

El error medio en la generación de la superficie de endemicidad de malaria por *P. falciparum* (Figura 3) reveló un sesgo mínimo en valores predichos de *Pf*PR2-10 con un valor global de 0.91, o una tendencia a sobre-estimar *Pf*PR2-10 en menos de uno por ciento (América = 0.63, África+ = 0.80, CSE Asia = 1.18) (Tabla 1). El error medio absoluto reveló una magnitud promedio de error de 9.75 (América = 3.52, África+ - 11.02, CSE Asia = 7.71) en las predicciones de *Pf*PR2-10 (Tabla 1). El coeficiente de correlación global entre valores observados y predichos fue de 0.82, indicando excelente correlación lineal a nivel global (Tabla 1); esto fue corroborado en la Figura 6A. La correlación a nivel regional fue más débil en América y CSE Asia (América = 0.03, África+ = 0.82, CSE Asia = 0.70) (Tabla 1). El semi-variograma de los residuos estandarizados del modelo (Figura 6B) mostró evidencia de una muy débil auto-correlación espacial, a intervalos de hasta dos grados decimales, aunque se observó que no fue estadísticamente significativa (Protocolo S4.2).

Prediciendo Clase Endémica

Las curvas receiver-operating-characteristic (ROC) y las estadísticas AUC para cada clase endémica se muestran en la Figura 6C y Tabla 2). Los valores globales de AUC para las tres clases endémicas excedieron el límite de 0.7 que indica una discriminación aceptable a buena, mientras que aquellos para las clases *Pf*PR2-10 ≤5% y *Pf*PR2-10 ≥40% excedieron el límite de 0.9 de discriminación excelente. En total, 70.8% de los puntos fueron clasificados correctamente (América = 80.0%, África+ = 70.6%, CSE Asia = 69.9%) y solamente 1.1% de los puntos fueron clasificados erróneamente a una clase no adyacente (América = 0.0%, África+ = 0.6%, CSE Asia = 2.5%) (Tabla 2). Una tabla de contingencia para cada clase esta disponible en el Protocolo S4.3.

Medidas Realistas de Incertidumbre para cada Predicción

El gráfico de probabilidad-probabilidad que compara umbrales de probabilidad predicha con probabilidades de cobertura observadas (Figura 6D) muestra una correspondencia cercana entre estas dos medidas, sugiriendo que el modelo brinda una representación razonablemente fiel de la incertidumbre en las predicciones puntuales. Sin embargo, la línea graficada cae ligeramente por encima de la línea 1:1 a lo largo de la mayoría de los valores umbrales, más evidentemente para umbrales de probabilidad entre 0.00 y alrededor de 0.25. Esto quiere decir que un umbral de probabilidad predicho de, por ejemplo, 0.1 probablemente se relaciona a un umbral de probabilidad real de alrededor de 0.2. En otras palabras, el modelo tiene una tendencia a subestimar la probabilidad de que *Pf*PR2-10 tome valores bajos (Protocolo S4, Figura S4.1A). Esta tendencia pudo haber provocado, a su vez, sobre-estimativos de *Pf*PR2-10 en áreas de baja endemicidad.

Mapa Global de Endemicidad de Malaria por *P. falciparum*

En el 2007 el área global de riesgo estable de *P. falciparum* fue de 29.73 millones de km2, distribuidos entre América (6.03 millones de km2, 20.30%), África+ (18.17 millones de km2, 61.10%) y CSE Asia (5.53 millones de km2, 18.60%) (Tabla 3). Hemos estimado anteriormente que, globalmente, 2 370 millones de personas viven en áreas de riesgo de transmisión de *P. falciparum* y entre éstos 980 millones viven en áreas de riesgo inestable [17,18]. Aquellas personas expuestas a riesgo estable, 1 383 millones, están distribuidas entre América (41 millones, 2.94%), África+ (657 millones, 47.48%) y CSE Asia (686 millones, 49.58%) (Tabla 4 y Figura 7). A continuación se describe la variación regional dentro de este riesgo estable de *P. falciparum*, estratificado en las clases endémicas baja (*Pf*PR2-10 ≤ 5%), intermedia (*Pf*PR2-10 >50 <40%) y alta (*Pf*PR2-10 ≥ 40%). En América y CSE Asia niños (grupos etáreos de 0-4 y 5-14 años) se acercan a un tercio (32%) del total de la población en riesgo. En África a proporción asciende a 43%.

América

El área de transmisión estable de *P. falciparum* en América se caracteriza por una endemicidad uniformemente baja (*Pf*PR2-10 ≤5%) (Figuras 3 y 4). El área total de riesgo estable cubre 6.03 millones de km2, principalmente en la cuenca Amazónica (Figuras 3 y 4). El total de las 40.64 millones de personas en esta región está expuesto a bajo riesgo. La prevalencia mediana fue de 2.17% con valores predichos máximo y mínimo de 0.31% y 8.81%, respectivamente (Figura 8C). Las distribuciones de frecuencia para esta región mostraron que los valores predichos se distribuyeron aproximadamente en forma simétrica alrededor de la mediana (Figura 8C). Los datos observados para el 2007 (Figura 8B) mostraron un rango similar pero un sesgo positivo, mientras que aquellos correspondientes a todos los años mostraron un rango mayor (max = 21.30%) y un sesgo positivo pronunciado (Figura 8A). La probabilidad de asignación correcta de clase endémica fue alta en América (Protocolo S4, Figura S4.2A), debido principalmente a la relativa uniformidad de encuestas reportando valores bajos de *Pf*PR2-10 [17,18] más que a una estructura espacial importante (Protocolo S1, Figura S1.1). Esto, combinado con la densidad poblacional relativamente baja de la región, produjo los valores más bajos del índice de incertidumbre ajustado a población (Figura 5B).

África, Yemen y Arabia Saudita (África+)

El área de transmisión estable de *P. falciparum* en África+ cubre 18.17 millones de km2, donde habitan 656.61 millones de personas en riesgo y abarca un amplio rango de intensidad de transmisión. De esta área, más de 4.03 millones de km2 (22.18%) y 114.5 millones de personas (17.44%) corresponden a *Pf*PR2-10 ≤5%. Estas áreas están ubicadas hacia el centro y este en latitudes al extremo norte y sur (Figuras 3 y 4). Esta clase endémica fue predicha con relativa confianza (Protocolo S4, Figura S4.1A). Las áreas de transmisión alta (*Pf*PR2-10 ≥40%) dominan África Occidental y gran parte de África Central, cubriendo 8.50 millones de km2 donde habitan 345.28 millones de personas. La probabilidad de predicción correcta de clase endémica fue alta en África Occidental pero mucho más baja en África Central (Protocolo S4, Figura S4.1C), debido a la relativa abundancia y escasez, respectivamente, de datos de *Pf*PR2-10 provenientes de encuestas actualizadas (Figura 1). Un área significativa del continente (5.63 millones de km2) corresponde a la clase endémica intermedia (*Pf*PR2-10 >5% a <40%) y aloja a 196.83 millones de personas en riesgo. Esta clase endémica fue predicha con la confianza más baja (Protocolo S4, Figura S4.1B).

La prevalencia mediana predicha para el área de transmisión estable del continente fue 33.34% con valores máximo y mínimo predichos de 0.20% y 75.40%, respectivamente (Figura 8C). La distribución de frecuencia de valores predichos (Figura 8) se centró en la mediana con una moda secundaria mucho menos pronunciada alrededor del 15% (Figura 8C). Esta distribución fue muy distinta a aquellas de los datos observados para todos los años y para el 2007, las que presentaron un sesgo positivo y valores máximos de 99.78% y 98.70%, respectivamente (Figuras 8A y 8B). El índice de incertidumbre ajustado a la población es heterogéneo en esta región, con valores altos evidentes en Etiopía para la clase endémica baja y valores altos evidentes en Nigeria para la clase endémica alta (Figura 5B), lo cual refleja la ocurrencia tanto de baja densidad de encuestas de *Pf*PR2-10 como de poblaciones numerosas en estas áreas.

**Asia Central y el Sudeste Asiático (CSE Asia)**

El área de transmisión estable de *P. falciparum* en Asia CSE se caracteriza por endemicidad baja (*Pf*PR2-10 ≤5%) con focos geográficamente pequeños pero epidemiológicamente importantes de riesgo intermedio (*Pf*PR2-10 >5% a <40%) y alto (*Pf*PR2-10 ≥40%) en, por ejemplo, el estado de Orissa al este de la India, el occidente de Myanmar y las zonas costeras de Papua Nueva Guinea. En total, el área de transmisión estable cubre 5.53 millones de km2 donde habitan 685.65 millones de personas en riesgo, estas últimas principalmente en India e Indonesia (Figuras 3 y 4). De éstos, más de 4.72 millones de km2 (85.54%) y 603.61 millones de personas en riesgo (88.03%) corresponden a endemicidad baja (*Pf*PR2-10 ≤5%). La prevalencia mediana predicha fue de 9.99% con valores máximo y mínimo de 0.006% y 45.40%, respectivamente. La distribución de frecuencia de valores *Pf*PR2-10 predichos estuvo positivamente sesgada (Figura 8C). La distribución de frecuencia de los datos observados para el 2007 mostró un rango similar pero un sesgo positivo más marcado (Figura 8B). El gráfico de datos observados para todos los años también mostró un sesgo positivo pero cubrió un rango mucho mayor de valores, con un máximo de 93.91% (Figura 8A). La probabilidad de predicción correcta de clase endémica fue relativamente alta en CSE Asia pero con una incertidumbre considerable en áreas limítrofes entre las clases endémicas baja e intermedia (Protocolo S4, Figura S4.1A). Este resultado combinado con la alta densidad poblacional de la región se tradujo en los valores más altos del índice de incertidumbre ajustado a la población, notorio particularmente en la India (Figura 5B).

**Discusión**

De lo que sabemos, por primera vez en 40 años, hemos contribuido con un mapa global, contemporáneo de endemicidad de malaria por *P. falciparum*. Este mapa supera las deficiencias claves, discutidas anteriormente, de mapas previos de distribución global de riesgo de malaria y es, por lo tanto, único en los siguientes aspectos. Primero, está basado en una base de datos de encuestas malariométricas que ha sido documentada minuciosamente y es geográficamente extensa (Protocolo S1) [58]; dicha base de datos será hecha pública (siempre y cuando los debidos permisos sean obtenidos) para el uso y la evaluación de todos en el 2009 [1]. Segundo, los métodos MBG (Protocolo S3) y los procedimientos de validación (Protocolo S4) utilizados han sido documentados en detalle y los códigos relevantes puestos a disposición del público. Todo el procedimiento de mapeo presentado aquí debe ser, por lo tanto, completamente reproducible por aquellos con acceso a los recursos computacionales requeridos. Tercero, se realizó una evaluación rigurosa de la incertidumbre asociada a los productos finales de tal forma que la confianza en los resultados pueda ser evaluada objetivamente (Figura 5).

La Situación de la Malaria en el Mundo en el 2007

El mundo está substancialmente menos afectado por la malaria de lo que uno podría inferir tras la revisión de mapas históricos [5,14], tanto por la reducción de sus límites espaciales como por la reducción de su endemicidad dentro de dichos límites. Se observa una impactante transición hacia una ecología de malaria de bajo riesgo que será explorada en más detalle en trabajos futuros.

De los 1 382 millones de personas expuestas a riesgo de transmisión estable de malaria en el 2007, 759 millones viven en condiciones de nivel endémico bajo de malaria (*Pf*PR2-10 ≤5%) en CSE Asia (604 millones, 79.55%), África+ (115 millones, 15.09%) y América (41 millones, 5.36%) (Tabla 4 y Figura 7). Estas poblaciones viven en condiciones donde los prospectos biológicos de control sostenido a muy bajos niveles de transmisión son alcanzables y compatibles con la meta, a largo plazo, de eliminación [19]. Por supuesto, las recomendaciones deben ser específicas a nivel sub-regional y nacional y deben obedecer a una evaluación juiciosa de factores ambientales, logísticos, financieros y políticos que puedan afectar la eficiencia con la cual los planes de intervención sean implementados [73,74,75]. En buena medida, el resto de la población global expuesta a riesgo estable de malaria es Africana: 197 millones viven bajo riesgo intermedio (*Pf*PR2-10 >5 a <40%) y 345 millones bajo condiciones de alto riesgo (*Pf*PR2-10 ≥40%) (Tabla 4 y Figura 7). En las áreas de riesgo intermedio, modelos matemáticos sugieren que la interrupción de la transmisión de *P. falciparum* puede lograrse con una distribución masiva de ITNs. Por otro lado, en áreas de alto riesgo la transmisión de malaria será más intratable y requerirá de un control más agresivo que incluya la adición de intervenciones complementarias [19,55].

Implementación Estadística y Validación del Modelo

El modelo presentado aquí representa una implementación a gran escala de técnicas geo-estadísticas Bayesianas modernas e incorpora una serie de innovaciones. La incorporación de un modelo de estandarización por edad permitió una asimilación coherente de datos de encuestas realizadas en una gran variedad de grupos etáreos, reconociendo, al mismo tiempo, la incertidumbre introducida por esta fuente adicional de variación. Asimismo, el uso de un campo aleatorio totalmente espacio-temporal permitió la incorporación de encuestas tan antiguas como las de 1985 en las predicciones de endemicidad contemporánea de *P. falciparum* en un marco plausible desde el punto de vista estadístico y epidemiológico.

Las técnicas MBG son excepcionalmente exigentes en cuanto a capacidad computacional incluso cuando se trata de problemas de predicción pequeños. A nuestro entender, esta es la primera vez que estas técnicas han sido utilizadas en la predicción de cualquier enfermedad a nivel global. Esta carga computacional impuso una serie de restricciones en el procedimiento de modelamiento que pudieron haber mejorado la capacidad predictiva. En particular, el presente modelo adopta una función única de media y covarianza en cada región, representando una suposición de estacionaridad de segundo orden en cada una. Las aproximaciones a campos aleatorios no estacionarios adoptadas en estudios de menor escala [32,76] representan posibles mejorías al presente modelo pero fueron consideradas impracticables a nivel global desde el punto de vista computacional.

La evaluación de las estadísticas de validación utilizadas reveló que el modelo se desempeñó satisfactoriamente en los tres aspectos esperados: predecir valores puntuales de *Pf*PR2-10 y clase endémica, y brindando medidas realistas de incertidumbre en las predicciones. Dada la gran variabilidad de endemicidad de *P. falciparum* incluso en distancias pequeñas, una correlación general de 0.82 entre las predicciones y los datos de validación, y una magnitud de error absoluto promedio de 9.75% *Pf*PR2-10 representan un nivel inesperado de precisión. Algunos aspectos de las medidas de incertidumbre del modelo son sub-óptimas, en particular, la tendencia a subestimar levemente la probabilidad de *Pf*PR2-10 de adoptar valores muy bajos. A pesar de ello, dada la multiplicidad de fuentes de incertidumbre que son captadas y propagadas a través del marco del modelo, las medidas de incertidumbre producidas representan una fuente de información muy útil en la generación de productos para los tomadores de decisiones.

El modelo fue ajustado utilizando MCMC [77,78]. MCMC es un algoritmo extremadamente poderoso y es el único de propósito general y factible, desde el punto de vista computacional, que esté disponible para la resolución de muchos problemas Bayesianos. Sin embargo, es un algoritmo aproximado. No existe un método infalible para estimar su error, pero utilizando un método heurístico (Protocolo S1.3) estimamos que nuestro “error de Monte Carlo” no es importante relativo a la incertidumbre de nuestras distribuciones posteriores.

La información contenida en los mapas presentados aquí y la incertidumbre asociada varían dependiendo de la escala geográfica. La variación en endemicidad a gran escala descrita entre regiones y países es inequívoca; ha sido cuantificada en forma sólida y tiene aplicación directa para quienes desarrollan planes globales. Conforme menores escalas son consideradas, la utilidad de estos mapas disminuye para planificadores de control local de malaria, aunque esto depende mucho de la disponibilidad y densidad de datos locales. Los umbrales y medidas de incertidumbre adecuadas variarán enormemente dependiendo del usuario y la aplicación de los mapas. Se sugiere que la diferenciación en endemicidad entre áreas más pequeñas que el primer nivel administrativo será inapropiada en la mayoría de países.

El estudio de las distribuciones de frecuencias de datos *Pf*PR2-10 observados, para todos los años y el 2007, y de la superficie *Pf*PR2-10 predicha reveló algunas características importantes. Primeramente, los datos del 2007 para todas las regiones presentaron una mediana y valor máximo substancialmente menores y mostraron un sesgo positivo más marcado que los datos de todos los años considerados conjuntamente (comparar las Figuras 8A y 8B). Segundo, se observaron diferencias importantes en todas las regiones entre las distribuciones de los datos del 2007 y aquellas de los valores *Pf*PR2-10 predichos (comparar las Figuras 8B y 8C). Estas últimas mostraron medianas más altas, tenían un menor sesgo positivo y, para América y África+, presentaron valores máximos substancialmente más bajos. La tendencia general hacia valores más altos de *Pf*PR2-10 en las superficies predichas puede deberse al agrupamiento espacial de las encuestas parasitarias. Debe recordarse que los datos recolectados representan una muestra oportunista dada por las motivaciones y limitaciones de una multitud de individuos, organizaciones y gobiernos. Al examinar estos datos visualmente se puede apreciar que una gran proporción de los mismos provienen de áreas de baja endemicidad, especialmente comparado con el hipotético caso de que los datos fueran el resultado de una muestra espacial aleatoria. Por lo tanto, las estadísticas de estos datos presentan un sesgo importante. Al predecir endemicidad en una superficie continua, el modelo MBG compensó implícitamente este agrupamiento y la distribución de frecuencia resultante no produjo el mismo sesgo.

El procedimiento MBG realiza predicciones en ubicaciones no muestreadas a través de combinaciones lineales de datos. Por esta razón, las superficies resultantes son inevitablemente más regulares que los datos utilizados en las predicciones. Una consecuencia de esto es que el rango de valores de la superficie predicha probablemente será menor que aquel correspondiente a los datos observados. Esto explica por qué la distribución de frecuencias de la superficie de *Pf*PR2-10 predicha cubre rangos de valores substancialmente menores. Una consecuencia importante de este efecto regularizador de la superficie es que la misma brinda una predicción sólida de endemicidad a gran escala pero menos apta para representar con precisión las variaciones que ocurren en distancias cortas.

Utilizando Co-variables Ambientales para Producir Mapas Continuos

Los efectos limitantes extremos de variables climáticas han sido incorporados en la definición de los límites estables e inestables de transmisión de malaria por *P. falciparum*, descritos anteriormente [18]. Existe una atracción ilusoria por el uso adicional de variables ambientales para aumentar la complejidad y mejorar la exactitud predictiva en el mapeo de endemicidad usando MBG. Esto ocurre porque dichos análisis se basan en la suposición de que la distribución y endemicidad contemporáneas se aproximan a su nicho fundamental [79,80]. Esta suposición es infundada porque la distribución global de la malaria se ha reducido substancialmente [18] en comparación a su hipotética distribución máxima alrededor del año 1900 [14]. Más aun, no se sabe a ciencia cierta hasta qué punto los determinantes ambientales de la distribución actual reflejan este nicho fundamental, cómo estas relaciones varían a nivel espacial y, por lo tanto, qué artefactos pudieran introducirse al incorporarlas en los análisis. Además, la consecución de variables ambientales “adecuadas” a nivel global con la fidelidad espacial y temporal requeridas [63,81] no es trivial. Finalmente, el grado en el cual estas relaciones se afectan por intervenciones existentes y espacialmente variables tampoco ha sido cuantificado. Existe una creciente evidencia de que los efectos de intervenciones pueden ser substanciales, que los mismos se han incrementado a partir del año 2000 y que dichos efectos suponen un mosaico espacial de influencia que confundiría significativamente cualquier relación existente [82,83,84,85,86,87,88,89,90]. No es sorprendente que no se haya encontrado sustento estadístico para la inclusión de una serie de variables climáticas [62] y ambientales derivadas de sensores remotos [63] (Protocolo S1.7).

Al haber evitado el uso de co-variables ambientales en este marco analítico, los mapas producidos están determinados únicamente por los datos de entrada y las suposiciones del modelo. Esta selección asegura una línea base parsimoniosa con respecto a la cual se pueden auditar cambios futuros.

Mejorías Geo-estadísticas Potenciales

Al adoptar la metodología de MBG, la exclusión de encuestas con una muestra menor a 50 individuos pierde sentido ya que la incertidumbre en relación a la población de la muestra es incorporada explícitamente por el modelo (Protocolo S3). Esta regla de exclusión fue implementada antes de que técnicas de MBG pudieran ser aplicadas a nivel global y será reconsiderada en futuras versiones del mapa.

La resolución espacial razonable a la cual las técnicas MBG pudieron ser implementadas fue de 5x5 km. Todo el procedimiento tomó en promedio un mes a esta resolución espacial y se ha estimado que tomaría alrededor de tres meses a una resolución de 1x1 km. Actualmente no existen planes de incrementar la resolución espacial de los mapas a nivel global ya que éstos son sólidos para el propósito de planificación regional para el cual fueron creados. En áreas más pequeñas, como países ricos en datos de *Pf*PR donde mapas de más alta resolución espacial pueden apoyar planes nacionales de control, productos de MBG a 1x1 km de resolución pueden ser considerados [33]. Más aun, a escalas nacionales la precisión del geo-posicionamiento de los datos *Pf*PR puede tener una influencia importante en la certeza de las predicciones, y, por lo tanto, procedimientos que puedan ayudar a incorporar estos efectos en los modelos pueden también investigarse [91,92,93]. En este estudio, la incertidumbre relacionada a errores de geo-posicionamiento fue considerada trivial en relación a las escalas de variación espacial en la endemicidad observada y dada la escala global de los productos del modelo.

No pudimos mejorar el desempeño predictivo del modelo de estandarización por edad a través del modelamiento de la sensibilidad, relacionada a la edad, de microscopia y tests rápidos de diagnostico (RDTs) separadamente o a través del modelamiento de especificidad diagnóstica. Se asumió, en estos análisis, que la precisión en la determinación de *Pf*PR por microscopia o RDTs es equivalente, pero la sensibilidad de la técnica diagnóstica [94,95,96,97,98] podría ser incluida en versiones futuras de este marco MBG.

No se encontró una solución al problema de implementar estas técnicas MBG a través de grandes distancias oceánicas (por ejemplo, el Caribe, Madagascar e Indonesia) dada la distribución global de datos de *Pf*PR y la falta de datos en algunas regiones (Figura 1). La potencial influencia de factores bio-geográficos sobre la transmisión de malaria fue ignorada en estos análisis. Futuras versiones del mapa deberían tener, idealmente, datos suficientes como para tratar a las islas separadamente y/o suficiente información acerca de la distribución de vectores *Anopheles* para informar las predicciones [56].

Hemos incorporado la habilidad de los análisis de considerar tendencias seculares de los datos de *Pf*PR y variaciones anuales en la transmisión de malaria. Este mapa nos provee de una descripción completa del carácter estacional de la malaria [99,100,101]; mayor información acerca de la variación global de estos efectos podría ser incorporada en futuras versiones del mapa.

La Ruta a Seguir: Dominio Público y Mapas Dinámicos

Los mapas presentados aquí estarán disponibles al público a partir de la publicación de este artículo. Los datos utilizados en estas predicciones serán convertidas de dominio público en el 2009 [1]. La infraestructura virtual para ofrecer este servicio está siendo desarrollada. El equipo de MAP anticipa la provisión de actualizaciones anuales del mapa global de endemicidad de malaria por *P. falciparum* y la base de datos de *Pf*PR correspondiente. Estas actualizaciones anuales reflejarán también los límites espaciales cambiantes de transmisión estable e inestable de *P. falciparum* [18] con el fin de definir con precisión los límites dentro de los cuales las predicciones de endemicidad deben implementarse. Si la comunidad internacional tiene éxito en “hacer retroceder” a la malaria, se deberán tomar decisiones informadas acerca de la discontinuidad temporal entre los límites espaciales de transmisión de *P. falciparum* (definidos, de ser posible, por el promedio de *Pf*API en los tres años más recientemente registrados [18]) y los datos de prevalencia (datos de *Pf*PR recolectados desde 1985).

Es obvio que el mapa presentado aquí es una representación estática para el año 2007 de una endemicidad de malaria que cambia con el tiempo. Ningún grado de sofisticación estadística puede eludir el hecho de que datos adicionales incrementarán la fidelidad del mapa, ya sea incrementando la resolución espacial de las encuestas malariométricas o actualizando una ubicación de donde se tienen resultados de encuestas con información más reciente. Los métodos han sido diseñados específicamente de forma que estas superficies puedan ser actualizadas rápidamente. La naturaleza uni-variable del marco estadístico también significa que los cambios en versiones futuras del mapa pueden ser atribuidos a la consecución de nuevos datos en áreas de alta incertidumbre (cambios en espacio) o a cambios resultantes de intervenciones exitosas o recesión de la enfermedad (cambios en tiempo) en lugar de a una mezcla temporal o espacial de la relación de los datos *Pf*PR2-10 a co-variables ambientales.

Alentamos la contribución de datos existentes para mejorar el mapa en áreas donde tenemos la más baja precisión espacial y de nuevos datos para sustentar la producción futura de mapas contemporáneos y actualizados. Las áreas de mayor incertidumbre pueden inferirse del inverso de la probabilidad de predicción de clase (Figura 5), pero estamos trabajando en refinar este indicador. Por lo tanto, una prioridad inmediata es generar mapas regionales que muestren la ubicación óptima de nuevas encuestas que deberían ser implementadas para reducir al máximo la varianza en la superficie de endemicidad existente al mínimo costo. Estas soluciones son más substanciales que la lista de áreas con más alta varianza provista aquí, porque (i) cada nueva encuesta cambiará la estructura de la varianza espacial y afectará la ubicación óptima de la siguiente encuesta; (ii) tanto el número como la distribución espacial de las encuestas afectará el resultado y se requerirá de múltiples simulaciones para proponer soluciones óptimas y (iii) las ubicaciones de encuestas potenciales deberán ser ponderadas adecuadamente de acuerdo a la distribución de la población humana.

Goles Inmediatos del Malaria Atlas Project

El enfoque inicial de MAP ha sido *P. falciparum* [1] debido a su relevancia epidemiológica global [102] y los mejores prospectos para su control y eliminación local [19]. Aún no hemos explorado en detalle el serio problema de malaria por *P. vivax* [103] a pesar de que su importancia clínica es cada vez más reconocida [104,105,106], pero hemos archivado más de 2 500 datos de prevalencia de *P. vivax* con los cuales podemos iniciar este proceso. Otro fin inmediato es el de refinar los estimativos de impacto global de *P. falciparum* (tanto de morbilidad [102] como de mortalidad [48,107,108]) para sustentar los estimativos globales de intervenciones contra la malaria. Los métodos estadísticos utilizados en este análisis permitirán que los siguientes estimativos de impacto representen la incertidumbre en las predicciones de manera más holística y veraz. A mediano plazo, la combinación de estos mapas globales de endemicidad con mapas de la distribución de vectores *Anopheles* dominantes [56] se tornará en una herramienta poderosa para que la comunidad de control de malaria tome decisiones más informadas sobre las intervenciones más adecuadas tomando en cuenta la bionomía de los vectores locales de importancia. En el largo plazo, esperamos no solamente monitorear y evaluar el progreso con estos mapas sino también mejorar nuestra habilidad de modelar la futura endemicidad de malaria y sustentar una evaluación objetiva hacia la eliminación de la malaria en el mundo.

Conclusiones

El estado del mundo de la malaria por *P. falciparum* en el 2007 representa una gran oportunidad para la comunidad internacional de actuar [109,110], pero estas acciones permanecen considerablemente bajas en recursos [111]. Independientemente de si las naciones promuevan el control intensivo y sostenido o, más ambiciosamente, la eliminación de la malaria [2,3,4,74,112,113,114] los pasos intermedios a seguir son parecidos [19]. Este recurso cartográfico ayudará a los países a determinar sus necesidades y servirá como una línea base para monitorear y evaluar el progreso hacia los goles de intervención. Deseamos continuar trabajando juntos con individuos, países y regiones para mejorar las futuras versiones de este mapa y, esperamos, poder documentar los éxitos de la lucha contra la malaria.

**References**

**Agradecimientos**

La elaboración de la base de datos de prevalencia parasitaria dependió críticamente de la contribución generosa de datos por parte de un gran grupo de personas vinculadas a la investigación y control de la malaria; los nombres de estas personas se encuentran listados en la página de agradecimientos del sitio Web de MAP (http://www.map.ac.uk/acknowledgements.html). Agradecemos también a Archie Clements por sus comentarios del presente artículo. Los autores reconocen al apoyo del Instituto de Investigaciones Médicas de Kenia (KEMRI); este artículo fue publicado con el permiso del director de KEMRI.

**Financiamiento**

SIH es financiado por un Senior Research Fellowship del Wellcome Trust (#079091), el cual también financia a CAG, AJT y PWG. AMN es financiado por el Wellcome Trust como un Research Training Fellow (#011829). BHM e IRFE reconocen el apoyo de la fundación Li Ka Shing. SB es financiado por el Wellcome Trust como un Career Development Fellow (#081673). RWS es un Principal Research Fellow (#079080) del Wellcome Trust, quien financia a AP. Este trabajo forma parte de los productos del Malaria Atlas Project (MAP, http://www.map.ox.ac.uk), financiado principalmente por el Wellcome Trust, Reino Unido. Las fuentes de financiamiento no cumplieron ningún rol en el diseño del estudio, la recopilación y análisis de datos, la decisión de publicar o la preparación del manuscrito.

**Contribución de los Autores**

SIH escribió el primer borrador del manuscrito. RWS, CAG, AMN, CWK, BHM, IRFE y SIH recopilaron y mapearon los datos de *Pf*PR. SIH y RWS concibieron los experimentos. AJT, AP y PWG refinaron e implementaron el protocolo experimental. DS desarrolló el algoritmo de estandarización por edad. SB y RAM contribuyeron con recomendaciones estadísticas durante todo el estudio. Todos los autores comentaron el borrador final del manuscrito.

**Conflicto de Intereses**

Los autores declaran que no existe conflicto de intereses.

**Abreviaciones**

África+	África, Yemen y Arabia Saudita

API	Incidencia Parasitaria Anual

AUC	Área Bajo la Curva

CSE Asia	Asia Central y el Sudeste Asiático

GRUMP	Global Rural-Urban Mapping Project

ITN	Mosquiteros Impregnados de Insecticida

MAP	Malaria Atlas Project

MBG	Geo-estadística Basada en Modelos

PAR	Poblaciones en Riesgo

*Pf*API	Incidencia Parasitaria Anual de *P. falciparum*

*Pf*PR	Prevalencia Parasitaria de *P. falciparum*

*PfPR*	Prevalencia Parasitaria de *P. vivax*

UE	Extensión Urbana

**Tabla 1.** Estadísticas de validación en la predicción de *Pf*PR2-10 por región. La media de cada distribución posterior predicha fue comparada con los datos observados. Los paréntesis indican el porcentaje de este valor con respecto a la media regional. África, Yemen y Arabia Saudita (África+) y Asia Central y el Sudeste Asiático (CSE Asia). Referirse al texto para una explicación completa de cómo fueron obtenidas estas estadísticas y la interpretación de los resultados.

| **Validation measure** | **America** | **Africa+** | **CSE Asia** | **World** |
| --- | --- | --- | --- | --- |
|  |  |  |  |  |
| Mean error | 0.627 (20.401) | 0.805 (3.070) | 1.184 (9.203) | 0.912 (4.267) |
| Mean absolute error | 3.522 (114.639) | 11.023 (42.024) | 7.705 (59.860) | 9.750 (45.625) |
| Correlation | 0.032 | 0.822 | 0.699 | 0.823 |

**Tabla 2.** Estadísticas de validación de la predicción de clase endémica de *Pf*PR2-10 por región. África, Yemen y Arabia Saudita (África+) y Asia Central y el Sudeste Asiático (CSE Asia). Referirse al texto para una explicación completa de cómo fueron obtenidas estas estadísticas y la interpretación de los resultados.

| **Validation measure** | **America** | **Africa+** | **CSE Asia** | **World** |
| --- | --- | --- | --- | --- |
|  |  |  |  |  |
| AUC (≤5%) | 0.452 | 0.927 | 0.885 | 0.915 |
| AUC (5%-<40%) | 0.452 | 0.758 | 0.813 | 0.779 |
| AUC (≥40%) | - | 0.921 | 0.842 | 0.927 |
| Overall % correct | 80.000 | 70.621 | 69.874 | 70.750 |
| ≤5% classed as ≥40% (%) | 0.000 | 0.188 | 0.418 | 0.250 |
| ≥40% classed as ≤5% (%) | 0.000 | 0.377 | 2.092 | 0.875 |

**Tabla 3.** Área de riesgo de malaria por *Plasmodium falciparum* en el 2007. Las áreas están representadas en millones de km^2^. Riesgo inestable (Incidencia Parasitaria Anual de *P. falciparum* (*Pf*API) <0.1 por 1000 personas por año (pa)) y riesgo estable (*Pf*API ≥0.1 por 1000 personas pa). El riesgo estable se subdivide en tres clases endémicas de *Pf*PR2-10 estandarizadas por edad [51] y relacionadas a control [19]. África, Yemen y Arabia Saudita (África+) y Asia Central y el Sudeste Asiático (CSE Asia).

| **Region** | **Unstable risk** | **Stable risk** | ***Pf*PR_2-10_** ≤**5%** | ***Pf*PR_2-10_ >5 - <40%** | ***Pf*PR_2-10_** ≥**40%** | **Total** |
| --- | --- | --- | --- | --- | --- | --- |
|  |  |  |  |  |  |  |
| America | 2.13 | 6.03 | 6.03 | 0.00 | 0.00 | 8.17 |
| Africa+ | 4.21 | 18.17 | 4.03 | 5.63 | 8.50 | 22.37 |
| CSE Asia | 3.40 | 5.53 | 4.72 | 0.69 | 0.11 | 8.93 |
| World | 9.74 | 29.72 | 14.79 | 6.32 | 8.61 | 39.46 |

**Tabla 4.** Población en riesgo (PAR) de malaria por *P. falciparum* en el 2007, expresada en millones. Riesgo inestable (Incidencia Parasitaria Anual de *P. falciparum* (*Pf*API) <0.1 por 1000 personas por año (pa)) y riesgo estable (*Pf*API ≥0.1 por 1000 personas pa). El riesgo estable se subdivide en tres clases endémicas de *Pf*PR2-10 estandarizadas por edad [51] y relacionadas a control [19]. África, Yemen y Arabia Saudita (África+) y Asia Central y el Sudeste Asiático (CSE Asia). Para cada región la PAR se divide por grupo etáreo en 0-4 años, 5-14 años y 15 o más años. ^†^Debe tomarse en cuenta que las restricciones computacionales obligaron a adoptar una resolución espacial de 5x5 km, lo que inevitablemente resulta en ligeras diferencias con respecto a estimaciones de PAR realizadas anteriormente a 1x1 km [18].

| **Region** | **Unstable risk_†_** | **Stable risk_†_** | ***Pf*PR_2-10_** ≤**5%** | ***Pf*PR_2-10_ >5 - <40%** | ***Pf*PR_2-10_** ≥**40%** | **Total** |
| --- | --- | --- | --- | --- | --- | --- |
|  |  |  |  |  |  |  |
| America |  |  |  |  |  |  |
| 0-4 | 5.62 | 4.51 | 4.51 | 0.00 | 0.00 | 10.14 |
| 5-14 | 10.66 | 8.66 | 8.66 | 0.00 | 0.00 | 19.32 |
| 15+ | 33.78 | 27.47 | 27.47 | 0.00 | 0.00 | 61.25 |
| Total | 50.06 | 40.64 | 40.64 | 0.00 | 0.00 | 90.71 |
|  |  |  |  |  |  |  |
| Africa+ |  |  |  |  |  |  |
| 0-4 | 3.29 | 108.64 | 15.48 | 32.90 | 60.26 | 111.93 |
| 5-14 | 5.64 | 170.80 | 25.35 | 51.48 | 93.98 | 176.44 |
| 15+ | 12.95 | 377.17 | 73.67 | 112.45 | 191.05 | 390.12 |
| Total | 21.88 | 656.61 | 114.50 | 196.83 | 345.28 | 678.49 |
|  |  |  |  |  |  |  |
| CSE Asia |  |  |  |  |  |  |
| 0-4 | 99.69 | 73.70 | 64.98 | 8.10 | 0.62 | 173.38 |
| 5-14 | 195.40 | 143.20 | 126.26 | 15.67 | 1.27 | 338.60 |
| 15+ | 616.17 | 468.75 | 412.37 | 51.52 | 4.86 | 1084.93 |
| Total | 911.26 | 685.65 | 603.61 | 75.29 | 6.75 | 1596.91 |
|  |  |  |  |  |  |  |
| World |  |  |  |  |  |  |
| 0-4 | 108.60 | 186.85 | 84.97 | 41.00 | 60.88 | 301.31 |
| 5-14 | 211.70 | 322.66 | 160.26 | 67.15 | 95.25 | 560.40 |
| 15+ | 662.90 | 873.40 | 513.51 | 163.97 | 195.91 | 1504.41 |
| Total | 983.20 | 1382.91 | 758.75 | 272.13 | 352.04 | 2366.11 |

**Figura 1**

Los límites espaciales de malaria por *Plasmodium falciparum* definidos por la incidencia parasitaria anual de *P. falciparum* (*Pf*API) conjuntamente con información de inteligencia médica y restricciones impuestas por límites de temperatura y aridez [17,18]. Las áreas se definieron como de riesgo estable (gris oscuro, donde *Pf*API ≥0.1 por 1000 pa), inestable (gris medio, donde *Pf*API <0.1 por 1000 pa) y sin riesgo (gris claro, donde *Pf*API =0 por 1000 pa) [17,18,19]. Se muestran encuestas de prevalencia parasitaria de *P. falciparum* realizadas entre el 1 de enero de 1985 y el 31 de julio del 2008. De los 8 938 registros obtenidos, 7 953 satisficieron los criterios de inclusión del modelo (ver Métodos y Protocolo S1.2) y son los que se muestran en este mapa. Los datos de encuestas están estandarizados por edad [51] (*Pf*PR2-10) y se muestran como un continuo de amarillo a rojo, 0-100% (ver leyenda del mapa). Las líneas punteadas separan las regiones de América; África, Yemen y Arabia Saudita (África+); y Asia Central y el Sudeste Asiático (CSE Asia).

**Figura 2**

Representación esquemática de los procedimientos y métodos de mapeo. Los rombos azules representan datos de entrada. Los cajones anaranjados denotan modelos y procedimientos experimentales; S1, Protocolo S1; S2, Protocolo S2; S3, Protocolo S3 y S4, Protocolo S4. Los cajones verdes indican datos de salida, con líneas punteadas los productos intermedios y con líneas sólidas los productos finales.

**Figura 3**

La distribución espacial de endemicidad de malaria por *Plasmodium falciparum*. Los datos corresponden a las predicciones puntuales de la media anual de *Pf*PR2-10 para el 2007 dentro de los límites de transmisión estable de *P. falciparum*, y están representados como un continuo de amarillo a rojo, 0-100% (ver leyenda del mapa). El resto de la superficie terrestre se definió como riesgo inestable (gris medio, donde *Pf*API <0.1 por 1000 pa) y sin riesgo (gris claro, donde *Pf*API =0 por 1000 pa) [17,18,19].

**Figura 4**

La distribución espacial de malaria por *Plasmodium falciparum* (*Pf*PR_2-10_) estratificada por clase endémica [19]. Se clasifica como bajo riesgo *Pf*PR_2-10_ ≤5%, rojo claro; riesgo intermedio *Pf*PR_2-10_ >5% a <40%, rojo medio, y alto riesgo *Pf*PR_2-10_ ≥40%, rojo oscuro. El mapa muestra la clase a la cual el valor *Pf*PR_2-10_ tiene la más alta probabilidad de pertenecer. El resto de la superficie terrestre se definió como riesgo inestable (gris medio, donde *Pf*API <0.1 por 1000 pa) y sin riesgo (gris claro, donde *Pf*API =0 por 1000 pa) [17,18,19].

**Figura 5**

A. El mapa ilustra la probabilidad de *Pf*PR_2-10_ de estar en la clase a la cual fue asignado en un continuo de amarillo a azul, de . Cualquier valor por encima de indica membresía asignada mejor que por azar. El resto de la superficie terrestre se definió como riesgo inestable (gris medio, donde *Pf*API <0.1 por 1000 pa) y sin riesgo (gris claro, donde *Pf*API =0 por 1000 pa) [17,18,19]. B. El índice de incertidumbre ajustado a población. Este índice muestra la importancia probable de incertidumbre calculada como el producto de la densidad poblacional en escala logarítmica (Protocolo S2.3) y el inverso de la probabilidad de asignación correcta de clase, en escala 0-1. El índice se muestra para la clase endémica más probable de *Pf*PR_2-10_. Las áreas de riesgo inestable y sin riesgo son como en A.

**Figura 6**

A. Gráfico de los datos *Pf*PR_2-10_ reales versus los predichos. B. Semi-variograma de los residuales de Pearson del modelo estandarizado estimado a distancias discretas (círculos) y comparado a un sobre de Monte Carlo (líneas punteadas) que representa el rango de valores esperados por azar en ausencia de auto-correlación espacial. C. Curvas de Característica de Receptor-Operador (ROC) para cada clase endémica de *Pf*PR_2-10_ (línea negra *Pf*PR_2-10_ ≤5%, línea roja *Pf*PR_2-10_ >5% a <40%, línea verde *Pf*PR_2-10_ ≥40%) y estadísticas asociadas de área bajo la curva (AUC). D. Gráfico de probabilidad-probabilidad que compara los umbrales de probabilidad predichos con el porcentaje actual de valores observados que exceden dichos umbrales. La línea 1:1 se muestra como referencia (línea punteada). Referirse al texto para una explicación completa de los procedimientos de validación y la interpretación de resultados.

**Figura 7**

Población en riesgo de malaria en el 2007. Los gráficos muestran la proporción de la población que habita en cada clase endémica de *Pf*PR_2-10_ predicha para América, África+, CSE Asia y el mundo. Los gráficos están representados a escala proporcionalmente al total de la población en cada región y los segmentos usan los mismos colores que en la Figura 4.

**Figura 8**

Gráficos de violín que muestran la distribución de frecuencias de datos *Pf*PR_2-10_ por región: A. todos los años, B. 2007 y C. correspondiente a la superficie predicha para el 2007. El ancho de cada polígono ilustra la frecuencia relativa de distintos valores de *Pf*PR_2-10_ en cada región. Los colores corresponden a aquellos usados en la Figura 4. Las barras negras representan los intervalos inter-cuartiles y los círculos blancos las medianas.

**Información Suplementaria**

Idioma alternativo Texto S1.

Traducido al francés por Frédéric Piel y Stéphanie Loute

En doi:10.1371/journal.pmed.1000048.sd001 (1.04 MB DOC).

Idioma alternativo Texto S2.

Traducido la chino por Robert Li

En doi:10.1371/journal.pmed.1000048.sd002 (437 KB DOC).

Idioma alternativo Texto S3.

Traducido al indonesio por Iqbal R.F. Elyazar y Siti Nurlela

En doi:10.1371/journal.pmed.1000048.sd003 (1.07 MB DOC).

Idioma alternativo Texto S4.

Traducido al vietnamita por Bui H. Manh

En doi:10.1371/journal.pmed.1000048.sd004 (574 KB DOC).

Idioma alternativo Texto S5.

Traducido al español por Carlos A. Guerra

En doi:10.1371/journal.pmed.1000048.sd005 (774 KB DOC).

**Protocolo S1**. La Base de Datos de Encuestas de Prevalencia Parasitaria de *Plasmodium Falciparum*

S1.1 Resumen de los Procedimientos de Búsqueda y Procesamiento de Datos

S1.2 Reglas de Exclusión de Datos

S1.3 Estandarización por Edad

S1.4 Semi-Variogramas de Datos de *Pf*PR_2-10_ por Región

S1.5 Filtro Geo-Estadístico para la Detección de Datos Externos Extremos

S1.6 Estadísticas Descriptivas de los Datos Malariométricos

S1.7 Relaciones con Co-Variables Ambientales

**Protocolo S2**. Bases de Datos y Procedimientos Demográficos

S2.1 Reglas de Clasificación de Encuestas Parasitarias en Urbana/Semi-Urbana/Rural

S2.2 Estatus Urbano/Semi-Urbano/Rural y Prevalencia

S2.3 Superficie Poblacional Continua GRUMP alfa

S2.4 Derivación de Poblaciones en Riesgo

**Protocolo S3**. Procedimientos Geo-Estadísticos en Base a Modelos

S3.1 Generalidades del Modelo Estadístico

S3.2 Especificación de ‘Priors’

S3.3 Estandarización por Edad

S3.4 Detalles de la Implementación

S3.5 Generalidades de la Generación de Mapas

**Protocolo S4**. Procedimientos de Validación del Modelo

S4.1 Creación de los ‘Sets’ de Validación

S4.2 Procedimientos para Evaluar el Rendimiento del Modelo

S4.3 Resultados Adicionales
