# Supplementary material for: A World Malaria Map: Plasmodium falciparum Endemicity in 2007
Source: PLoS Med. 2009 Mar 24;6(3):e1000048. doi: 10.1371/journal.pmed.1000048 (PMC2659708; doi:10.1371/journal.pmed.1000048)
Supplement: Supplementary file 6 [file pmed.1000048.sd006.doc]

**Protocol S1: The *Plasmodium falciparum* Parasite Rate Malariometric Survey Database**

The Malaria Atlas Project (MAP) *Plasmodium falciparum* parasite rate (*Pf*PR) database is the largest and most contemporary global compilation of malariometric surveys [1]. *Pf*PR data are updated through a continuous process of data search and acquisition and as of the 31 July 2008 the database approached 9,000 distinct community surveys, across 78 of the 87 *P. falciparum* malaria endemic countries (*Pf*MEC). This document describes the important aspects of the data assembly process and provides a description of key features of the *Pf*PR data used for this 2007 iteration of the global *P. falciparum* endemicity map.

**S1.1 Summary of Data Search and Data Abstraction Procedures**

The MAP *Pf*PR database has strict inclusion criteria that have been described in detail elsewhere [1]. In brief, only post-1984 *Pf*PR data were included as they were most representative of contemporary malaria endemicity. The statistical precision of malariometric surveys increases with the number of people sampled; a minimum sample size of 50 was used as the threshold for inclusion [2,3]. The surveys were also required to represent random or whole community samples of the population. Repeat surveys at the same location were recorded. The assumption of independence was required between longitudinal observations and this was supported by requiring a minimum six month observation interval between surveys, after which time the presence or absence of infections in re-sampled individuals was considered to be unrelated to their infection status in the first survey [4,5]. For studies where the survey design indicated that it had been implemented to sample seasonal variation in transmission this six month rule was relaxed. Significant search efforts were then directed to find formal (published scientific papers) and informal (“grey” literature and unpublished data) sources of information on malaria surveys that fulfilled these primary inclusion criteria.

Extensive data searches were undertaken using online reference catalogues of the formal literature, mainly PubMed (http://www.ncbi.nlm.nih.gov/sites/entrez), ISI Web of Knowledge (http://wok.mimas.ac.uk) and Scopus (http://www.scopus.com), supplemented with additional regional archives [1]. Five main searches were undertaken in: March 2006, October 2006, May 2007, March 2008 and June 2008. In addition, several monthly and weekly malaria research summary compendia (Malaria World newsletters (http://www.malaria-world.com/), RBM alerts (http://www.rollbackmalaria.org) and the Environmental Health at USAID malaria bulletins (http://www.ehproject.org/)) were checked regularly for relevant publications.

The MAP *Pf*PR database has a simple design that captures the main aspects of malariometric surveys [1] relevant to the modelling described in this paper. The abstraction of the data from primary sources has been conducted by the same individuals (CAG, RWS and SIH) throughout the two years of data assembly. On inspection, many information sources were incomplete and required follow-up with the authors for missing and additional information (for example, disaggregating data to the village level, helping find the geographic location of the surveyed communities and/or providing survey dates). In order to expedite this process, further engage the malaria research and control community and request access to additional data, a contacts database was created from these initial searches. This was augmented from various sources and used to send a request for *Pf*PR data in June 2006 [1]. These communications with malaria specialists have proven to be an extremely effective method of data provision and information retrieval and will be pursued actively for the duration of the MAP [1]. All contributors of data are gratefully acknowledged online (http://www.map.ox.ac.uk/acknowledgements.html) and arranged alphabetically by surname under the country name for which the data were provided.

One particularly challenging aspect of this data assembly was the geo-positioning of each record, for which very detailed guidelines have been developed [1]. These guidelines allowed surveys to be categorised according to the area from which they were sampled as: points (≤10 km^2^), wide-areas (>10 and ≤25 km^2^), small polygons (>25 and ≤100 km^2^) and large polygons (>100 km^2^). Records that were likely to be geo-positioned less precisely were marked as either a “good” (inaccuracy <5 kilometres) or a “rough” guess (inaccuracy >5 kilometres).

The entire database was checked for data consistency with a series of simple queries that generated reports on the number of records by data field, so that inconsistencies could be easily flagged and checked. In addition, before the data exclusion rules were applied, each row of the database was checked for missing or inconsistent information by CAG, RWS and SIH.

**S1.2. Data Exclusion Rules**

By the 31 July 2008, 8,950 temporally independent community *P. falciparum* parasite prevalence surveys were identified globally from 78 of the 87 *Pf*MEC globally [6]. Those *Pf*MECs not represented in the database were Bangladesh, Belize, Bhutan, Djibouti, Dominican Republic, Guyana, Iran, Kyrgyzstan and Panama. In addition, five countries endemic only for *P. vivax* also contributed zero prevalence *P. falciparum* estimates for the modelling: Costa Rica, Iraq, Mexico, Morocco and Turkey.

Several exclusions were applied to these data in order to optimise their spatial resolution and geo-positional fidelity in the final modelling dataset (see Figure S1.2 and Section S1.5). First, 213 large and 134 small polygons were excluded because these records represented areas larger than the 5×5 km spatial resolution grid output of the model (see main text). Second, 349 surveys that could not be geo-located were excluded as this is a pre-requisite for spatial analysis. Amongst the remaining 8,254 geo-located point or wide-area records, the accuracy of the geographic coordinates for 27 was classified as a “rough” guess. These surveys were excluded because of the likely uncertainty in the estimate of their location exceeded that of the 5×5 km spatial resolution of the model output. Finally, longitudinal surveys that could not be temporally disaggregated (n=153) and those for which the month of survey was unavailable (n=83) were also excluded as this information is needed for the space-time geostatistical procedures adopted. These exclusions left 7,991 point or wide-area, geo-located parasite prevalence records for further analysis.

**S1.3. Age-Standardisation**

*Pf*PR data are reported in a diversity of age ranges and 311 different age groups were found in the *Pf*PR database. Since population measures of malaria prevalence are age-dependent [7,8,9,10,11,12], it was necessary to standardise the *Pf*PR survey estimates to a single, representative age group for comparison. All surveys were standardized to the 2 (2.00) to 10 (9.99) year age group (hereafter *Pf*PR_2-10_) using catalytic conversion models first adapted to malaria by Pull and Grab [13] and described in detail elsewhere [14].

**S1.4. Semi-Variograms of *Pf*PR_2-10_ Data by Region**

Sample semi-variograms were computed independently for each of the three global regions (America; Africa, Yemen and Saudi Arabia (Africa+); and Central and South East Asia (CSE Asia)) to assess the extent to which the pattern of spatial heterogeneity in observed *Pf*PR_2-10_ values differed between these regions (Figure 1, main text). Sample semi-variograms are a widely used diagnostic tool that allows simple visualisations of the spatial autocorrelation structure exhibited by spatial data [15]. More formally, they represent the effect of separation distance (termed lag, ) on the expected difference between pairs of observed values (quantified using semi-variance, ) [16]. For these analyses, the sample semi-variogram was defined as:

(S1.1)

where represents a value of *Pf*PR_2-10_ observed at location and represents a second observation at a distance from the first. By discretizing lags into a series of bins of width , such that each value of actually represents a distance interval , semi-variances are computed as the mean semi-variance amongst the set of pairs of observations separated by distances within that interval. Comparison of the sample semi-variograms for each global region revealed marked differences in spatial structure (Figure S1.1). The semi-variograms for Africa+ and CSE Asia both indicate spatial autocorrelation in *Pf*PR_2-10_ values up to lags of around one decimal degree, although the much larger value of the *y*-axis intercept for Africa+ indicates a larger contribution of aspatial variation in that region. The semi-variogram for America displays consistently small semi-variances indicating much more uniform *Pf*PR_2-10_ endemicity than exhibited in Africa+ and CSE Asia, with only weak spatial autocorrelation. In conjunction with prior biogeographical, entomological and epidemiological knowledge [17,18], these distinct semi-variograms provided a rationale for the decision to stratify the modelling into the three major global regions.

**Figure S1.1.** Sample semi-variograms of *Pf*PR_2-10_ for A America (black line), B Africa+ (red line), and C the CSE Asia (green line) regions. A decimal degree is equal to approximately 111 km at the equator.

**S1.5. Geostatistical Filter for Detection of Extreme Outliers**

Section S1.2 describes the rigorous series of exclusion rules that were applied to candidate *Pf*PR survey data before inclusion in the modelling dataset used in this study. Subsequent to the age-standardisation (Section S1.3) and as a final data quality check, the data were scanned for extreme statistical outliers: values that were deemed statistically implausible for the population of surveys sampled. A non-spatial approach to identifying *Pf*PR_2-10_ values as statistical outliers might involve the use of an arbitrary threshold of deviance such as standard deviations from the mean of a global or regional population. Such an approach would neglect the fact that both the mean and variance of *Pf*PR_2-10_ may vary spatially. It is intuitive that the probability of an unusually large *Pf*PR_2-10_ value being a genuine “outlier” is larger if it is in a neighbourhood of generally much smaller values and/or the neighbourhood is generally uniform. Conversely, if that same point lies within a neighbourhood with either similarly large values, or in which values have a tendency to vary greatly, then the probability that it is an outlier is less. To incorporate these heuristic considerations a spatial filtering algorithm was developed which implemented the following procedure for each data location in turn.

A large regional neighbourhood was specified around each data location, , defined as the set of data located within 20 decimal degrees of , and excluding the datum itself. For each of the unique pairs of data within the regional neighbourhood the semi-variance was computed along with the separation distance between each datum in the pair. Taken together, this set of pair-wise values of and , calculated without reference to the datum , captured the spatial autocorrelation structure within the regional neighbourhood of location . Semi-variances were then calculated between the datum and its 20 nearest neighbours . Each of these 20 semi-variance values were then compared to regional semi-variance values computed for pairs at similar separation distances, defined as within 0.1 decimal degrees (dd). This required the definition of the subset for each of the semi-variance values . To avoid comparing data from substantially different time periods, both the local and regional semi-variance values were derived using only surveys carried out within a five year window centred on the survey at . The quantile position of each semi-variance value within the corresponding subset was recorded and the mean of the 20 quantile positions was calculated.

The value provides, for every datum , an index of the extent to which the observed *Pf*PR_2-10_ value differs from its neighbours relative to the extent expected given the distances to those neighbours, as defined by the regional semi-variances. Data for which the value exceeded 0.95 were classed as outliers, and in this way a total of 38 points (America = 3, Africa+ = 14, CSE Asia = 21) were identified and removed. Details of these surveys will be made available on request. Following the implementation of this last data exclusion procedure, the final data set used for further modelling consisted of 7,953 data (America = 261, Africa+ = 5,307, CSE Asia = 2,385) of which (America = 192, Africa+ = 4,291, CSE Asia = 1,795) represented unique survey locations. The sequence of data inclusion rules and the number of data removed at each stage are summarised in Figure S1.2 and Table S1.1.

**Table S1.1.** Summary of data exclusions by region.

|  | **America** | **Africa+** | **CSE Asia** | **Total** |
| --- | --- | --- | --- | --- |
| **Countries with *Pf*PR survey data_†_** | **14** | **48** | **21** | **83** |
|  |  |  |  |  |
| **Total identified** | **313** | **5,849** | **2,788** | **8,950** |
|  |  |  |  |  |
| Large polygons | 5 | 134 | 74 | 213 |
| Small polygons | 8 | 43 | 83 | 134 |
| Unable to geo-locate | 13 | 245 | 91 | 349 |
| Imprecise geo-location | 0 | 4 | 23 | 27 |
| Exclusion of temporally aggregated surveys | 15 | 64 | 74 | 153 |
| Exclusion of surveys with missing month | 8 | 38 | 37 | 83 |
| Geostatistical space-time outliers | 3 | 14 | 21 | 38 |
|  |  |  |  |  |
| **Total surveys** | **261** | **5,307** | **2,385** | **7,953** |
| **Spatially unique surveys** | **192** | **4,291** | **1,795** | **6,278** |

**_†_**Those countries in which *Plasmodium falciparum* parasite rate (*Pf*PR) survey data were found are listed alphabetically by region: America (Bolivia, Brazil, Colombia, Costa Rica, Ecuador, French Guiana, Guatemala, Haiti, Honduras, Mexico, Nicaragua, Peru, Suriname, Venezuela); Africa+ (Angola, Benin, Botswana, Burkina Faso, Burundi, Cameroon, Cape Verde, Central African Republic, Chad, Comoros, Congo, Côte d'Ivoire, Democratic Republic of the Congo, Equatorial Guinea, Eritrea, Ethiopia, Gabon, The Gambia, Ghana, Guinea, Guinea-Bissau, Kenya, Liberia, Madagascar, Malawi, Mali, Mauritania, Mayotte, Morocco, Mozambique, Namibia, Niger, Nigeria, Rwanda, São Tomé and Principe, Saudi Arabia, Senegal, Sierra Leone, Somalia, South Africa, Sudan, Swaziland, Togo, Uganda, United Republic of Tanzania, Yemen, Zambia, Zimbabwe) and CSE Asia (Afghanistan, Cambodia, China, India, Indonesia, Iraq, Lao PeopleSs Democratic Republic, Malaysia, Myanmar, Nepal, Pakistan, Papua New Guinea, Philippines, Solomon Islands, Sri Lanka, Tajikistan, Thailand, Timor-Leste, Turkey, Vanuatu, Viet Nam).

**Figure S1.2:** Sequence of data exclusion rules for the formulation of a refined global *P. falciparum* parasite prevalence database for modelling. For each region the number excluded, *n*, and the number remaining are shown at each stage of exclusion.

**S1.6. Malariometric Survey Data Summary and Descriptive Statistics**

This section presents summary descriptions of some of the most relevant aspects of the *Pf*PR database. Table S1.2 summarises these descriptions by region.

Data and Geographic Coordinate Sources

The most productive source of information for the database, both primary *Pf*PR data and geographic coordinates required for mapping, was direct communication with malaria specialists. Although peer-reviewed data sources were an important contributor in all regions, these represented between a sixth and a third of all the primary sources of *Pf*PR data, depending on the region (Table S1.2). In terms of geographic coordinates, personal communication, including GPS, was by far the most important source for mapping survey data across the three regions, especially in the Africa+ and CSE Asia regions (Table S1.2).

Time Periods Observed

Table S1.2 shows the frequency of *Pf*PR_2-10_ records by four time periods since 1985. In all three regions, particularly in Africa+ and CSE Asia, the frequency of surveys in the database conducted after the year 2000 increases. This is also illustrated by the increase in the gradient of the cumulative number of surveys by date (Figure S1.3). Furthermore a simple plot of the median *Pf*PR_2-10_ by year for the duration of the surveys (Figure S1.3) shows a clear secular movement of decreasing *Pf*PR_2-10_. This has important implications for the analyses (See Protocol S3 and main text), affirming the need for a full space-time geostatistical model.

**Figure S1.3.** Median (red horizontal bars) and IQR (black horizontal bars) *Pf*PR_2-10_ per year with smooth fit line (continuous thick black) generated by a loess smoother. Also shown is the cumulative number of data available through time (dashed line).

Age Ranges Archived

The large number of age ranges observed in malariometric surveys required the standardisation to a single age group of 2-10 years (see Section 1.3). Table S1.2 presents the frequency of records by four upper age limits, given the difficulty in summarising all of the 311 age groups. In Africa+, the great majority of records corresponded to an age group of children of up to 15 years. In contrast, in America and CSE Asia, most of the sampling included adults.

Diagnostic Methods

Microscopy was the dominant method of *P. falciparum* malaria diagnosis in the majority of surveys in the MAP database. Rapid diagnostic tests (RDTs), however, are increasingly used in large malariometric surveys, due to their relative ease of use and requirement of less training [19,20]. This was particularly evident in Africa+, where national malaria prevalence surveys using RDTs are increasingly common [21,22] (Figure S1.4).

**Figure S1.4**. Percentage of surveys using RDTs rather than microscopy by year for each region. Vertical bars are 95% binomial confidence intervals.

Numbers Examined in Surveys

In Africa+, most sample sizes were between 50 and 100, with a median of 95, whereas in America and CSE Asia, the majority of sample sizes were between 101 and 500, with medians of 131 and 137, respectively (Table S1.2). The model (see Protocol S3 and main text) requires from each survey both the sample size and the number of individuals that are *P. falciparum* positive. Where *Pf*PR was known, but the number of positive people was not stated explicitly, the latter was imputed using the assumed or known sample size and the *Pf*PR. Authors sometimes also stated, for instance, that “approximately 200 individuals were sampled” and a *Pf*PR for the community was reported. In these cases the approximate value for the number examined was accepted.

Despite requiring the number of blood samples examined to exceed 50, exceptions were made if this number was not specified but was strongly implied as being higher. For example, an author might state that all households of a community were sampled and the community is known to be considerably larger than 50 households. If no additional information was found, then the sample size was assumed conservatively as 50, thus maximizing the potential sampling error with their inclusion. This was the procedure for 147 records in the final database, mostly in Africa+ (Table S1.2).

**Table S1.2.** Summary figures of the most important aspects of the *Pf*PR_2-10_ data by region. Summaries are after all exclusions shown in Table S1.1.

|  | **America** | **Africa+** | **CSE Asia** | **Total** |
| --- | --- | --- | --- | --- |
|  | **(n = 261)** | **(n = 5,307)** | **(n = 2,385)** | **(n = 7,953)** |
|  |  |  |  |  |
| **Primary source of *Pf*PR data** |  |  |  |  |
| Peer reviewed sources | 102 | 888 | 492 | 1,482 |
| Personal communication | 113 | 2,735 | 1,497 | 4,345 |
| Reports_†_ | 46 | 1,684 | 396 | 2,126 |
|  |  |  |  |  |
| **Source of spatial coordinates** |  |  |  |  |
| Personal communication | 65 | 722 | 737 | 1,524 |
| Encarta | 88 | 1,396 | 230 | 1,714 |
| GPS (through pers. comm.) | 31 | 1,362 | 552 | 1,945 |
| Combination | 54 | 664 | 441 | 1,159 |
| Other digital gazetteers | 14 | 1,112 | 177 | 1,303 |
| Paper source | 8 | 14 | 11 | 33 |
| Map | 1 | 37 | 237 | 275 |
|  |  |  |  |  |
| **Date range_‡_** |  |  |  |  |
| 1985-1989 | 48 | 718 | 170 | 936 |
| 1990-1994 | 30 | 956 | 249 | 1,235 |
| 1995-1999 | 84 | 691 | 302 | 1,077 |
| 2000-2008 | 99 | 2,942 | 1,664 | 4,705 |
|  |  |  |  |  |
|  |  |  |  |  |
| **Upper age sampled** |  |  |  |  |
| <=10 | 5 | 1,697 | 190 | 1,892 |
| >10 and <=15 | 5 | 1,511 | 129 | 1,645 |
| >15 and <=20 | 0 | 410 | 124 | 534 |
| >20 | 251 | 1,689 | 1,942 | 3,882 |
|  |  |  |  |  |
| **Total unique age ranges** | 56 | 226 | 114 | 311_¥_ |
|  |  |  |  |  |
| **Method** |  |  |  |  |
| Microscopy | 242 | 4,011 | 2,155 | 6,408 |
| RDT | 19 | 1,296 | 230 | 1,545 |
|  |  |  |  |  |
| **Denominator** |  |  |  |  |
| No denominator | 0 | 138 | 9 | 147 |
| 50-100 | 104 | 2,818 | 771 | 3,693 |
| 101-500 | 124 | 2,078 | 1,404 | 3,606 |
| >500 | 33 | 273 | 201 | 507 |
| Median (IQR) | 131 (77-259) | 95 (56-164) | 137 (88-216) | 103 (63-190) |

_†_Ministry of Health reports, theses and other unpublished sources. **_‡_**The end year of the survey was used to assign the date range. _¥_These figures do not sum as some age ranges are repeated across regions.

**S1.7. Relationships with Environmental Covariates**

Global Synoptic Climate Data

Global gridded climate data at 10 minute (18×18 km) spatial resolution, created through the interpolation of meteorological station data from 1961-1990 to form synoptic monthly images, were obtained [23]. From these data, the images representing monthly mean temperature, precipitation and relative humidity were extracted. These monthly images were then subject to temporal Fourier processing [24], and for each of the three climatic variables, the Fourier variables representing the mean (amplitude 0), amplitude of the peak of the annual and biannual cycles (amplitudes 1 and 2), and timing of the peak of the annual and biannual cycles (phases 1 and 2), were obtained for analysis.

In each plot of the panel an environmental covariate from New *et al.* [23] is plotted against logit*Pf*PR_2-10_ (Figure S1.5). The argument of the logit function is the posterior expectation of the underlying *Pf*PR in the 2-10 age group using a uniform prior. The logit function maps this value from the interval [0,1] to the real line. *Pf*PR_2-10_ is estimated using the method of Smith *et al.* [14] rather than the Bayesian method described in Protocol S3. Each relationship is fit with the linear model and the variance explained marked in the top right of each plot. The wide scatter in the plots is evident, with the variance explained always below 10%.

**Figure S1.5.** A panel of plots showing environmental covariates from New *et al.* [23] against logit*Pf*PR_2-10_. On the *x*-axes, the first two letters indicate the environmental covariate: tm = temperature, rh = relative humidity, pr = precipitation. The second two letters indicate the temporal Fourier variable: a0 = mean, a1 = amplitude of the annual cycle, a2 = amplitude of the biannual cycle, p1 = phase of the annual cycle, p2 = phase of the biannual cycle. Temperature values are in degrees Celsius.

Global MODIS Satellite Sensor Data

Global MODerate Resolution Imaging Spectrometer (MODIS) imagery from the Terra satellite [25] were acquired for the period 2001-2005 at 1×1 km spatial resolution. These were processed to create environmental variables that have proven valuable in the mapping of vector-borne disease distributions [24]: land surface temperature (LST), middle infrared reflectance (MIR) and enhanced vegetation index (EVI), an improved index over the widely used normalized difference vegetation index (NDVI). The 2001-2005 sequences of these variables were then subject to temporal Fourier processing, as described in Scharlemann *et al*. [26], and for each of the three environmental variables, the Fourier variables representing the mean (amplitude 0), amplitude of the peak of the annual and biannual cycles (amplitudes 1 and 2), and timing of the peak of the annual and biannual cycles (phases 1 and 2), were obtained for analysis.

In each plot of the panel an environmental covariate from Scharlemann *et al.* [26] is plotted against logit*Pf*PR_2-10_ (Figure S1.6). The argument of the logit function is computed as in Figure S1.5. The wide scatter in the plots is again evident, with the variance explained always below 5%.

**Figure S1.6.** A panel of plots showing environmental covariates from Scharlemann *et al.* [26] against logit*Pf*PR_2-10_. On the x-axes, the first three letters indicate the environmental covariate: LST = land surface temperature, MIR = middle infrared reflectance, EVI = enhanced vegetation index. The following two letters indicate the temporal Fourier variable: a0 = mean, a1 = amplitude of the annual cycle, a2 = amplitude of the biannual cycle, p1 = phase of the annual cycle, p2 = phase of the biannual cycle. LST values are in degrees Celsius.

**References**

1. Guerra CA, Hay SI, Lucioparedes LS, Gikandi PW, Tatem AJ, et al. (2007) Assembling a global database of malaria parasite prevalence for the Malaria Atlas Project. Malar J 6: 17.

2. Gregory RD, Blackburn TM (1991) Parasite prevalence and host sample size. Parasitol Today 7: 316-318.

3. Jovani R, Tella JL (2006) Parasite prevalence and sample size: misconceptions and solutions. Trends Parasitol 22: 214-218.

4. Sama W, Owusu-Agyei S, Felger I, Vounatsou P, Smith T (2005) An immigration-death model to estimate the duration of malaria infection when detectability of the parasite is imperfect. Stat Med 24: 3269-3288.

5. Smith DL, McKenzie FE, Snow RW, Hay SI (2007) Revisiting the basic reproductive number for malaria and its implications for malaria control. PLoS Biol 5: e42.

6. Guerra CA, Gikandi PW, Tatem AJ, Noor AM, Smith DL, et al. (2008) The limits and intensity of *Plasmodium falciparum* transmission: implications for malaria control and elimination worldwide. PLoS Med 5: e38.

7. Baird JK (1995) Host age as a determinant of naturally acquired-immunity to *Plasmodium falciparum*. Parasitol Today 11: 105-111.

8. Baird JK, Jones TR, Danudirgo EW, Annis BA, Bangs MJ, et al. (1991) Age-dependent acquired protection against *Plasmodium falciparum* in people having two years exposure to hyperendemic malaria. Am J Trop Med Hyg 45: 65-76.

9. Baird JK, Purnomo, Basri H, Bangs MJ, Andersen EM, et al. (1993) Age-specific prevalence of *Plasmodium falciparum* among six populations with limited histories of exposure to endemic malaria. Am J Trop Med Hyg 49: 707-719.

10. Baird JK (1995) Host age as a determinant of naturally acquired immunity to *Plasmodium falciparum*. Parasitol Today 11: 105-111.

11. Baird JK, Jones TR, Danudirgo EW, Annis BA, Bangs MJ, et al. (1991) Age-dependent acquired protection against *Plasmodium falciparum* in people having two years exposure to hyperendemic malaria. Am J Trop Med Hyg 45: 65-76.

12. Baird JK, Purnomo, Basri H, Bangs MJ, Andersen EM, et al. (1993) Age-specific prevalence of *Plasmodium falciparum* among six populations with limited histories of exposure to endemic malaria. Am J Trop Med Hyg 49: 707-719.

13. Pull JH, Grab B (1974) Simple epidemiological model for evaluating malaria inoculation rate and risk of infection in infants. Bull World Health Organ 51: 507-516.

14. Smith DL, Guerra CA, Snow RW, Hay SI (2007) Standardizing estimates of the *Plasmodium falciparum* parasite rate. Malar J 6: 131.

15. Diggle PJ, Ribeiro PJ (2007) Model-based geostatistics; Bickel P, Diggle P, Fienberg S, Gather U, Olkin I et al., editors. New York: Springer. 228 p.

16. Goovaerts P (1997) Geostatistics for natural resource evaluation. New York, U.S.A.: Oxford University Press. 483 p.

17. Macdonald G (1957) Local features of malaria. The epidemiology and control of malaria. London: Oxford University Press. pp. 63-99.

18. Mouchet J, Carnevale P, Coosemans M, Julvez J, Manguin S, et al. (2004) Paludisme et grandes régions biogéographiques. Biodiversité du paludisme dans le monde. Montrouge, France: John Libbey Eurotext.

19. Moody A (2002) Rapid diagnostic tests for malaria parasites. Clin Microbiol Rev 15: 66-78.

20. Murray CK, Gasser RA, Jr., Magill AJ, Miller RS (2008) Update on rapid diagnostic testing for malaria. Clin Microbiol Rev 21: 97-110.

21. Kleinschmidt I, Sharp B, Benavente LE, Schwabe C, Torrez M, et al. (2006) Reduction in infection with *Plasmodium falciparum* one year after the introduction of malaria control interventions on Bioko Island, Equatorial Guinea. Am J Trop Med Hyg 74: 972-978.

22. Noor AM, Clements ACA, Gething PW, Moloney G, Borle M, et al. (2008) Spatial prediction of *Plasmodium falciparum* prevalence in Somalia. Malar J 7: 159.

23. New M, Lister D, Hulme M, Makin I (2002) A high-resolution data set of surface climate over global land areas. Clim Res 21: 1-25.

24. Hay SI, Tatem AJ, Graham AJ, Goetz SJ, Rogers DJ (2006) Global environmental data for mapping infectious disease distribution. Adv Parasitol 62: 37-77.

25. Tatem AJ, Goetz SJ, Hay SI (2004) Terra and Aqua: new data for epidemiology and public health. Int J Appl Earth Obs Geoinformation 6: 33-46.

26. Scharlemann JPW, Benz D, Hay SI, Purse BV, Tatem AJ, et al. (2008) Global data for ecology and epidemiology: a novel algorithm for temporal Fourier processing MODIS data. PLoS One 3: e1408.
