# Supplementary material for: A World Malaria Map: Plasmodium falciparum Endemicity in 2007
Source: PLoS Med. 2009 Mar 24;6(3):e1000048. doi: 10.1371/journal.pmed.1000048 (PMC2659708; doi:10.1371/journal.pmed.1000048)
Supplement: Supplementary file 7 [file pmed.1000048.sd007.doc]

**Protocol S2. Demographic Databases and Procedures**

A brief description of the steps taken to define urban/peri-urban/rural status for the environments in which age-standardized *Plasmodium falciparum* parasite rate surveys, *Pf*PR_2-10_, (see Protocol S1) were undertaken and the impact of such status on prevalence is presented here. Full details on the exploratory analyses and experiments undertaken are provided elsewhere [1].

**S2.1 Parasite Rate Survey Urban/Peri-Urban/Rural Classification Rules**

In the age-standardized *Pf*PR_2-10_ database (up to and including Protocol S1.3), 37.8% of surveys include an author-defined urban or rural assignment (617 urban, 2400 rural). For Africa, Yemen and Saudi Arabia (hereafter Africa+), significantly lower *Pf*PR_2-10_ values were observed in author-defined urban areas compared to rural areas when spatially (within 100 km) and temporally (within five years) proximate survey pairs of urban and rural *Pf*PR_2-10_ were compared [1]. No significant differences were observed for urban-rural survey pairs elsewhere in the *P. falciparum* malaria endemic world. The ability to use this information in further analyses required defining the urban-rural status for all surveys and exploring the significance of this relationship for the entire *Pf*PR_2-10_ database.

Author definitions were assumed to be the most epidemiologically pertinent definitions of urban and rural status for these surveys. These definitions were used to test which of several global urban extent maps available in the public-domain matched these author-defined definitions most accurately. The best surface would then provide the basis for an uniform urban-rural assignation of all *Pf*PR_2-10_ surveys.

The results showed that the urban extent mask created as part of the Global Rural Urban Mapping Project (GRUMP) [2] mapped most accurately the author-defined urban/rural designations of *Pf*PR_2-10_ surveys [1]. These were tested using confusion matrix-based accuracy measures, including percentage correct, user’s and producer’s accuracies and the kappa coefficient of agreement [3,4]. The GRUMP urban extent mask (GRUMP-UE) mapped 88.73% of author-defined designations correctly, with a kappa value of 0.664.

The GRUMP-UE surface is created principally using night-time lights satellite imagery, supplemented with data derived from tactical pilotage charts and known settlement points [2,5,6]. Through using satellite night-time lights as the basis for mapping urban areas, GRUMP-UE has been shown to overestimate urban extent due to the “overglow” effects seen in such imagery [7], resulting in the inclusion of less intensely-urban “peri-urban” areas. An approach was therefore required to identify the intensely urban areas within the urban extents defined by GRUMP-UE. Previous work has shown that identifying areas where population density is greater than or equal to 1000 people per km^2^ maps a malaria-relevant divide between urban and peri-urban malaria transmission in Africa [8]. This definition was used and implemented with the Gridded Population of the World version 3 (GPW3) [2] population density database for 2000, projected to 2007 using standard techniques (see Section S2.4).

The GPW3 human population density surface is derived from the most recent available national census and other demographic data, resolved at the highest possible administrative boundary level, and areal weighted [9] to a 5×5 km spatial resolution grid. Those areas where GPW3 population densities were greater than or equal to 1000 people per km^2^, and within urban extents defined by GRUMP-UE, were mapped as “urban”. Those areas within GRUMP-UE extents with GPW3 population densities lower than 1000 people per km^2^ were mapped as “peri-urban”. The final urban and peri-urban extents are shown in Figure S.2.1.

**Figure S.2.1**. Urban, peri-urban and rural extents for all malaria endemic countries. Full details of their derivation are provided in the text

**S2.2 Urban/Peri-Urban/Rural Status and Prevalence**

The *Pf*PR_2-10_ points were overlaid on the new urban classified map and then re-assigned as urban (n = 334), peri-urban (n = 541) or rural (n = 7052). This changed the author-defined assignment of “rural” to peri-urban in 126 cases and urban in 21 cases, while changing the author-defined assignment of “urban” to rural for 193 surveys and peri-urban for 196. A reanalysis of the urban/peri-urban/rural status and *Pf*PR_2-10_ was then conducted on the complete *Pf*PR_2-10_ database.

All sets of surveys mapped as urban, peri-urban and rural within 100 km and ten years of each other were identified, and their *Pf*PR_2-10_ values plotted. Where multiple surveys existed for the same location, the survey closest in time to the counterpart urban/peri-urban/rural survey was used. No sets of such points existed for the America region, while just six were available for analyses in CSE Asia (Figure S2.2).

Tests on neighbouring “urban-peri-urban”, “urban-rural” and “rural-peri-urban” sets of *Pf*PR_2-10_ surveys consistently showed transmission differences in Africa+ (significant at the p<0.001 level using the Friedman’s ranking test [10]) (Figure S2.2). No significant differences were found for the CSE Asia region. These urban, peri-urban and rural classifications were then made available to the model based geostatistical approaches described in Protocol S3.

**Figure S2.2**. A boxplot showing the differences in average *Pf*PR_2-10_ between GRUMP-UE/GPW3-defined rural, peri-urban and urban areas using 145 sets of spatially and temporally proximate malariometric survey pairs for Africa+ [1]. The central bar is the median (rural *Pf*PR_2-10_ = 33.9%, peri-urban *Pf*PR_2-10_ = 26.9%, and urban *Pf*PR_2-10_ = 13.5%), the box defines the positions of the first and third quartiles of the distribution, while the whiskers extend to include all points no more than 1.5 times the inter-quartile range defined by the boxes. Points beyond this range are marked as outliers (the circles).

**S2.3 GRUMP alpha Human Population Surface**

The GRUMP spatial population database provides gridded population counts and population density estimates for the years 1990, 1995 and 2000, both adjusted and unadjusted to the United Nations’ national population estimates. The data and methods used to construct GRUMP are described in detail elsewhere [2,11]. In brief, the initial procedures are identical to those of GPW3 except that the areal weighting [9] is implemented on a 1×1 km spatial resolution grid. The urban extent mask, GRUMP-UE (see section S2.1), is then used to adjust population numbers within each extent to match estimated totals for each settlement in question. Finally, the individual national population surfaces are projected to the years 1990, 1995 and 2000 and adjusted to match the national population totals estimated by the United Nations’ Population Division [12]. The clearly documented methods, the substantially larger number of administrative units used to create the database, and tests showing its higher accuracy over other products [9,13], have led to the adoption of GRUMP products within the Malaria Atlas Project (MAP, http://www.map.ox.ac.uk).

**S2.4 Population and Area at Risk Derivation**

GRUMP UN-adjusted population counts were obtained for the year 2000. This population surface was projected to 2007 by applying national, medium variant, inter-censal growth rates by country [12]. An estimate of population in 2007 was produced using the following equation; where is the required 2007 population within a pixel, is the population within the same pixel at year , is the number of years between year and 2007, and is the average growth rate [14]. This resulted in a population count surface of 1×1 km spatial resolution for 2007. These population counts were then stratified nationally by age group using UN defined age structures [12] to obtain under 5 years, 5-14 years and 15+ years population count surfaces.

By overlaying the 5×5 km predicted endemicity classes on each of the 2007 population count surfaces, populations at risk in each class were estimated globally. The quantification of areas within each endemicity class was undertaken by first reprojecting the predicted endemicity class map from geographic to Mollweide equal area projection in ArcGIS 9.2 (ESRI, 2006). The areas covered by each class were then calculated in km^2^.

**References**

1. Tatem AJ, Guerra CA, Kabaria CW, Noor AM, Hay SI (2008) Human population, urban settlement patterns and their impact *Plasmodium falciparum* malaria endemicity. Malar J 7: 218.

2. Balk DL, Deichmann U, Yetman G, Pozzi F, Hay SI, et al. (2006) Determining global population distribution: methods, applications and data. Adv Parasitol 62: 119-156.

3. Congalton RG (1991) A review of assessing the accuracy of classifications of remotely sensed data. Remote Sens Environ 37: 35-46.

4. Congalton RG, Green K (1993) A practical look at the sources of confusion in error matrix generation. Photogramm Eng Remote Sens 59: 641-644.

5. Elvidge CD, Baugh KE, Dietz JB, Bland T, Sutton PC, et al. (1999) Radiance calibration of DMSP-OLS low-light imaging data of human settlements. Remote Sens Environ 68: 77-88.

6. Elvidge CD, Hobson VR, Nelson IL, Safran JM, Tuttle BT, et al. (2003) Overview of DMSP OLS and scope of applications. In: Mesev V, editor. Remotely Sensed Cities. London: Taylor and Francis. pp. 281-333.

7. Tatem AJ, Noor AM, Hay SI (2005) Assessing the accuracy of satellite derived global and national urban maps in Kenya. Remote Sens Environ 96: 87-97.

8. Hay SI, Guerra CA, Tatem AJ, Atkinson PM, Snow RW (2005) Urbanization, malaria transmission and disease burden in Africa. Nat Rev Microbiol 3: 81-90.

9. Hay SI, Noor AM, Nelson A, Tatem AJ (2005) The accuracy of human population maps for public health application. Trop Med Int Health 10: 1073-1086.

10. Friedman M (1940) A comparison of alternative tests of significance for the problem of *m* rankings. Ann Math Stat 11: 86-92.

11. CIESIN/IFPRI/WB/CIAT (2007) Global Rural Urban Mapping Project (GRUMP) alpha: Gridded Population of the World, version 2, with urban reallocation (GPW-UR). Available at http://sedac.ciesin.columbia.edu/gpw. Palisades, New York, USA: Center for International Earth Science Information Network, Columbia University / International Food Policy Research Institute / The World Bank / and Centro Internacional de Agricultura Tropical.

12. U.N.P.D. (2006) World population prospects: the 2006 revision population database. http://esa.un.org/unpp/. New York: United Nations Population Division (U.N.D.P).

13. Tatem AJ, Noor AM, von Hagen C, Di Gregorio A, Hay SI (2007) High resolution population maps for low income nations: combining land cover and census in East Africa. PLoS One 2: e1298.

14. Deichmann U (1996) A review of spatial population database design and modelling. Santa Barbara, California, U.S.A.: National Center for Geographic Information and Analysis (NCGIA), University of California, Santa Barbara (UCSB).
