# Supplementary material for: A World Malaria Map: Plasmodium falciparum Endemicity in 2007
Source: PLoS Med. 2009 Mar 24;6(3):e1000048. doi: 10.1371/journal.pmed.1000048 (PMC2659708; doi:10.1371/journal.pmed.1000048)
Supplement: Supplementary file 8 [file pmed.1000048.sd008.doc]

**Protocol S3. Model-based geostatistical procedures**

**S3.1 Overview of the Statistical Model**

Bayesian Inference

In Bayesian model-based geostatistics, conversion of geo-referenced data to a continuous surface begins with specification of a probability model. Such models are usually collections of interconnected conditional probability statements, for example, “given the number of individuals participating in a sample and the population-wide *Plasmodium falciparum* parasite rate (*Pf*PR), the number of positive individuals in that sample is distributed binomially.” These statements, including “prior” probability distributions for basic model parameters, should represent the modeller's understanding of the relationship between the inferential targets (the *Pf*PR surface) and the data (the sample outcomes). The most broadly applicable class of methods for fitting Bayesian probability models is Markov chain Monte Carlo, or MCMC [1,2].

Model Overview

The probability model used in the current study assumed that individuals participating in each sample (*Pf*PR survey) were *P. falciparum* positive with a probability that was the product of two components: (i) a continuous function of the time and location of the survey and (ii) a factor that depended on the age range of individuals included in the survey. The continuous function of time and location was modelled as a Gaussian process or Gaussian random field [3]. The distributions of the age-standardization factors were modelled using a Bayesian version of the procedure described by Smith *et al*. [4], and inferred based on detailed age-stratified information reported by a small number of surveys specified in that study.

This section first presents a schematic graphical representation of the full model (Figure S3.1). Such representations are helpful for visualizing complicated probability models. The main probability model is then presented formally. Section S3.2 gives a discussion of the specifications of some prior distributions; S3.3 discusses the age-standardization sub-model; S3.4 describes the Markov chain Monte Carlo algorithm used to fit the model; and S3.5 describes the conversion of the posterior predictive distribution to imagery.

**Figure S3.1** A schematic of the probability model, expressed as a directed acyclic graph. Ovals represent variables in the model. Grey ovals represent variables that have been observed. Arrows indicate conditional distributions written down in the model. For example, the distribution of the number positive in a parasite rate survey is specified based on the corresponding age-standardization factor, the underlying *Pf*PR surface and the time and location of the survey. It is possible to fit the age-standardization sub-model separately with minimal inconsistency. The maps presented in this study are summaries of the posterior of the upper right-hand node, the *Pf*PR_2-10_ surface.

Formal Presentation of Model

Each of the individuals in sample was assumed *P. falciparum* positive with probability , so the number positive was distributed binomially:

The coefficient was modelled as a Gaussian process. The factor converted to the probability that individuals within the age range reported for study were *P. falciparum* positive, and that the infection was detected, thereby accounting for the influence of age on the probability of detection [4]. The age-standardization factor in each population was assumed drawn independently from a distribution whose parameters were the lower and upper ages reported in study :

The form of is described in section S3.3.

*Pf*PR_2-10_ is the *P. falciparum* parasite rate for individuals between ages 2 (2.00) and 10 (9.99). Its value at an arbitrary location and time is the product of and another age-standardization factor, , distributed as :

The factor converted to the probability that individuals between ages 2 and 10 at location are *P. falciparum* positive. The age-standardization factor of a survey is the product of the age-standardization factor associated with the same place, time and age range and the sensitivity of the survey.

The coefficient at arbitrary location and time was modelled as the inverse-logit function applied to a random field evaluated at , plus an unstructured (random) component .

The components were assumed independent and identically distributed for each location and time and a standard diffuse but proper prior with expectation 0.25 was assigned to their variance .

The random field was modelled as a Gaussian process whose mean was a linear function of time and whose covariance was :

The variables and indicated whether was urban or peri-urban, respectively. The covariance of the field was modelled using a version of the spatiotemporal covariance function recently recommended by Stein [5] (equation 12):

is the modified Bessel function of the second kind of order , and is the gamma function [6,7].

Spatial distance between a pair of points and was computed as great-circle distance multiplied by a factor that depends on the angle of inclination of the vector pointing from to . was computed as if latitude and longitude were Euclidean coordinates (on a cylindrical projection):

Computing distance in this way allows for anisotropy.

When (that is, for points at the same location but different times), the covariance function reduces to

As temporal separation increases, the covariance approaches a limiting sinusoid rather than zero. When , on the other hand (for points at different locations but the same time), it reduces to a standard exponential form with range parameter . Unlike standard sum-product models, this covariance function does not have problematic ridges along its axes [5].

The square root of the partial sill and the spatial range parameter were assigned skew-normal priors:

and their specification is described further in section S3.2.

The standard “one-over-*x*” prior for the temporal scale parameter resulted in collapse to zero, a common artefact when data do not contain strong information. A relatively vague but proper prior, which has an expectation of ten years, was used instead.

A uniform prior was assigned to the direction of anisotropy parameter and to the square of the “eccentricity” parameter , which controls the amount of anisotropy,

a uniform prior was assigned to the limiting autocorrelation in the temporal direction,

and a standard prior was assigned to the components of the mean:

**S3.2 Prior Specification**

Although standard priors such as the improper “flat” prior [1] were assigned to most of the basic model parameters, subjective skew-normal priors [8] were specified for the range and partial sill parameters and , because MCMC mixing time appeared to be bottlenecked by correlations involving these parameters and because their effects on the *Pf*PR_2-10_ surface were relatively easy to visualize. A program written in the R language [9] (available on request) was prepared to help those authors with a more extensive experience of malaria geography to visualize the parameters’ effects. The required inputs were values for and , as well as a handful of hypothetical surveys (latitude, longitude, number examined and number positive). The outputs were realizations from, and quantile surfaces for, the approximate posterior predictive distribution of *Pf*PR_2-10_ over a small geographic area. The authors (PWG, SIH) who provided the priors preferred to express their opinions as triples (lower 95% credible interval, mode, upper 95% credible interval), which were converted to skew-normal priors [8] on the log scale. A table of these triples, with the corresponding parameters of the log-skew-normal distribution, is shown below. The units of are radians on the Earth’s surface.

|  | lower 95% | mode | upper 95% |  |  |  |
| --- | --- | --- | --- | --- | --- | --- |
| Africa+, | 1 | 2 | 6 | 0.0535 | 0.559 | 3.21 |
| America, | 0.1 | 1 | 4 | 0.607 | 1.24 | -1.17 |
| CSE Asia, | 1 | 2 | 6 | 0.0535 | 0.559 | 3.21 |
| Africa+, | 0.0157 | 0.0784 | 0.392 | -2.54 | 0.704 | -0.0150 |
| America, | 0.0157 | 0.0784 | 0.470 | -2.58 | 0.741 | 0.0498 |
| CSE Asia, | 0.00784 | 0.0470 | 0.157 | -2.97 | 0.571 | -0.143 |

**Table S3.1** The prior modes and credible intervals specified for the spatial range and the spatial partial sill in the three regions, and the corresponding parameters of the log-scale skew-normal prior [8].

**S3.3 Age-Standardization**

The Age-Standardization Model

This section explains in more detail the age-standardization model that underpins the distributions and of the age-standardization factors And . Following Smith *et al*. [4], the functional form of Pull and Grab [10] was used to model the probability that an individual of age is *P. falciparum* positive:

which is increasing. In addition, the age dependent probability of detection of a *P. falciparum* infection (the sensitivity) was modelled as:

which is decreasing for . The resulting model for the probability that a *P. falciparum* infection would be detected in an individual drawn from the population within age range is:

where is the age distribution of the study participants. Some allowance for population-to-population variation was introduced into the age-standardization procedure of Smith *et al*. [4]. The age distribution was assumed to be drawn from some probability distribution . Similarly, , , and were assumed to be drawn from a probability distribution . Further, the ages of participants in study were assumed to be drawn uniformly from the population’s age distribution within the published age limits and . The distributions and , if they are known, can be converted into the probability distributions and required in the previous section.

Adaptation of the Smith *et al*. Model

The sub-model for age-standardization used in this study is presented formally below. In the following, indexes populations (within the training set) and indexes age bins. Each individual in population and age class was assumed *P. falciparum* positive with probability given by , where and bound age class :

The probability that an individual in population and age class was *P. falciparum* positive was modelled as a function of the local detection-probability parameters , and , and epidemiological parameters and , as well as the age distribution of study participants using the Smith *et al*. model for age dependence:

The age distribution of study participants was assumed to have been generated by randomly drawing study participants from the population-wide age distribution :

The age distribution in each population was assumed drawn from a common Dirichlet distribution [4]. Dirichlet random variables are discrete probability distributions: positive vectors that sum to one. The distribution was “centered” on a typical age distribution :

The parameter controlled the extent to which deviates from such that large values of resulted in small deviations. A Dirichlet prior was assigned to the typical age distribution :

The epidemiological and detection-probability parameters, except , in each population were assumed drawn from a common distribution. in each population was modelled as independent.

This assumption is inconsistent with the main spatial model, but it allows the age-standardization model to be fitted separately. Separating a single large model into several smaller models is usually computationally advantageous when MCMC is used for fitting.

A standard prior was used for the mean of the transformed epidemiological and detection-probability parameters’ distribution:

Following Gelman *et al*. [1], the covariance matrix was factored into the vector of marginal standard deviations and the correlation matrix . A standard prior was used for the marginal standard deviations and for the off-diagonal elements of the upper triangle of , subject to the constraint that must be positive definite:

A standard prior was also assigned to , the parameter controlling the concentration of the distribution of the ’s.

The posterior predictive distribution of (the predictive distribution of *Pf*PR in a single age bin given that is equal to 1) is shown in the figure S3.2.

**Figure S3.2** The posterior predictive distribution of the relative probability of *P. falciparum* infection and detection (assuming ) as a function of age. The heavy black line shows the median, and the dark and light grey regions show the centred 50% and 90% credible intervals, respectively.

Validation of the Age-standardization Model

Smith *et al*. validated their methods using a testing set consisting of 121 *Pf*PR surveys, each of which considered two distinct age ranges [4]. To assess the performance of the Bayesian version of their method presented here, the ratios of the paired measurements were considered. These ratios were independent of , a necessary feature for predictive validation since no predictive distribution for had been inferred at that stage. For each pair in the testing set, a predictive distribution for *Pf*PR/*Pf*PR was computed. Averaging these distributions gave a predictive distribution for *Pf*PR/*Pf*PR for any survey drawn at random from the testing set. Figure S3.3 shows the empirical CDF from the testing set and the CDF of the posterior predictive distribution.

**Figure S3.3** A comparison of the empirical (solid) and predicted (broken) CDFs of the log of the ratio of *Pf*PR values for paired observations drawn from the testing set. The observed CDF increases more slowly than the predicted CDF, indicating that there was more variation in the testing set than predicted.

The observed CDF increases more slowly than the predicted CDF, demonstrating that there was relatively more probability mass in the tails of the empirical distribution than predicted by the model. Future research will be directed toward obtaining better predictions. The data in the training set did not exhibit extra-binomial variation, so it may be appropriate to use more flexible distributions, such as Dirichlet process mixtures, for the parameters and the age distributions .

**S3.4 Implementation Details**

Implementation of the MBG procedure was divided into two computational tasks: (i) an inference stage in which MCMC was used to generate samples from the posterior distribution of the parameter set and of the space-time random field at the data locations; and (ii) a prediction stage in which samples were generated from the posterior distribution of *Pf*PR_2-10_ at each prediction location on a 5×5 km grid within the limits of stable transmission. Each stage is explained in more detail below.

Markov chain Monte Carlo Algorithm

Both the main geostatistical model and the age-standardization sub-model were fitted using the MCMC algorithm [1,2]. The algorithm was implemented in the Python [11] and FORTRAN programming languages using the open-source Bayesian statistics package PyMC [12] and the numerical packages SciPy and NumPy [13]. The code (which contains working values for all tuning parameters) is available on request, as are the dynamic traces of all unobserved parameters.

MCMC Algorithm for the Main Spatial Model

The parameter and the evaluation of at the sampling locations and times were updated using Gibbs steps [1]. The evaluation of the uncorrelated process was updated one point at a time using random-walk Metropolis steps [1]. The model parameters , , , , , , , , and were updated jointly using the method of Haario, Saksman and Tamminen [14].

Within the MCMC loop, the age-standardization factors were not imputed explicitly. We were not interested in their particular values, and marginalizing out ”nuisance parameters” ahead of time usually improves the mixing of MCMC algorithms. Before the MCMC loop began, the marginal likelihood:

was approximated using standard Monte Carlo integration for several values of . That is, values for the model parameters , , and and the age distribution were drawn from their posterior predictive distributions, then expression (S3.1) was evaluated to obtain , then the binomial probability was evaluated for several values of . The probabilities resulting from many such draws were averaged. Inside the MCMC loop, the marginal likelihood function for arbitrary values of was evaluated by interpolation.

MCMC Algorithm for the Age Correction Model

The age distribution parameters , and are independent of the relative *Pf*PR parameters , , , , , , and given the data, so these two groups of parameters were inferred using separate MCMC algorithms.

In the MCMC for the age distribution parameters, the survey populations’ age distributions were updated using Gibbs steps [1]. The concentration parameter was updated using random-walk Metropolis steps [1]. The typical age distribution was represented as a normalized sequence of gamma random variables [15], and these variables were updated one at a time using random-walk Metropolis steps [1].

In the MCMC for the relative *Pf*PR parameters, the distributional parameters , and were updated jointly using the method of Haario, Saksman and Tamminen [14]. The parameters , , , and were updated jointly for each population using the same method.

Spatiotemporal Prediction and Map Generation

The output of the MCMC stage consisted of samples from the posterior of the parameter set and a corresponding samples from the posterior of the space-time random field at each of the data locations . For every sample, the conditional distribution of the 2007 annual mean of the space-time random field was predicted at each prediction location on the nodes of a regular 5×5 km grid within the spatial limits of stable *P. falciparum* transmission [16]. The distribution of the 2007 annual mean for prediction location was modelled as the joint multivariate normal distribution of the 12 predicted monthly values for that year specified by a 12 element mean vector and 12 × 12 variance-covariance matrix :

The mean vector was computed using:

where and were the predicted mean of the random field at each of the 12 prediction times at spatial location and at each of the data locations respectively, and were the data-to-prediction and data-to-data covariance matrices respectively, and was the vector of data values. The 12 × 12 variance-covariance matrix was computed using:

The value of the sample of , the variance of the unstructured component , was then added to the diagonal of the matrix and 1000 draws were made randomly from the distribution specified in equation S3.34. These draws represented samples from the posterior distribution of and were subject to an inverse logit transform and then multiplied by the sample of the age-standardization parameter to form the sample from the posterior distribution of the predicted mean annual 2007 *Pf*PR_2-10_ endemicity surface at location :

This procedure was repeated for every sample to form the set of samples for each prediction location. The point estimate of *Pf*PR_2-10_ endemicity at each location was defined as the mean of this set, whilst the probability of membership to each class was computed as the proportion of these samples falling within each class definition: *Pf*PR_2-10_ ≤5%; *Pf*PR_2-10_ >5%-<40%; *Pf*PR_2-10_ ≥40%.

Monte Carlo Standard Errors

Monte Carlo standard errors for the expectations of the scalar parameters and the class membership probabilities of selected points were estimated using the procedure recommended by Flegal *et al*. [17]. For the class membership probabilities, it was first verified by direct simulation that the Monte Carlo standard error of the estimated class membership probabilities given each sample from the posterior was negligible. That is, we set large enough that the estimated class membership probability was, for all intents and purposes, a deterministic function of the model parameters and the value of at the data locations. This observation allowed us to apply the method of Flegal *et al*. [17] to the class membership probabilities directly. The largest half-widths amongst several representative prediction points were found to be approximately 0.3%.

**S3.4 Overview of Map Generation**

A deterministic model outputting a single predicted value of *Pf*PR_2-10_ for each pixel would lead to a single predicted map. The output of the MBG model for each pixel, however, was not a single prediction but a large set of possible values representing the predicted posterior distribution of *Pf*PR_2-10_ and, together, provide a complete model of our uncertainty. Examples of these posterior distributions are shown in Figure S3.4 for three pixels selected from nations in each modelled region (Brazil (America), Côte d'Ivoire (Africa+) and Papua New Guinea (CSE Asia)). The information contained in each pixel’s posterior distribution was summarized in different ways to make three different global maps. First, the mean of each posterior distribution was calculated which became our “point estimate” of *Pf*PR_2-10_ (3%, 68% and 22% respectively for the three example sites in Figure S3.4) and these values for each pixel generated the global map shown in Figure 3 of the main text. We also calculated the probability of membership to each of the three endemicity classes which was found by calculating the relative proportion of the posterior distribution falling within each class. These class probabilities were used to generate three further maps. The map shown in Figure 4 of the main text displays, for each pixel, which of the three classes had the largest class probability and was therefore considered the ‘most likely’ endemicity class for that pixel (<=5%, >=40% and >5%-<40% respectively for the three example sites). The map shown in Figure 5 of the main text displays the class probability for that most likely class (0.89, 0.80 and 0.4 respectively for the three example sites). This latter map can be interpreted as a summary of the modelled uncertainty in assigning class memberships. Values close to one indicated a high degree of certainty in class assignment whilst values close to one-third indicated a high degree of uncertainty; that is even the “most likely class” was only marginally more likely than the other classes). Figure S4.1A-C show the probability of membership to each class individually, regardless of which was calculated as most likely. The map shown in Figure S4.2 displays the standard deviation of each posterior distribution, which can be interpreted as a further indicator of uncertainty. Where predictions have a high uncertainty the posterior distribution will be dispersed across a wide range of possible *Pf*PR_2-10_ values and will therefore have a larger standard deviation that a more certain, less dispersed posterior distribution.

Figure S3.4. The posterior distribution predicted from the MBG model, displayed as a probability density across the range of possible *Pf*PR_2-10_, for selected 5×5 km pixels in A Brazil (note probability density off scale), B Côte d'Ivoire and C Papua New Guinea. The equivalent posterior distributions from each pixel on a 5×5 km global grid were used to generate a series of maps, as described in the text. The three coloured regions on each plot highlight the section of each distribution corresponding to the three endemicity classes (<=5%, >=40% and >5%-<40%), the area of which is equivalent to the probability of membership to that class.

**References**
