# Supplementary material for: A World Malaria Map: Plasmodium falciparum Endemicity in 2007
Source: PLoS Med. 2009 Mar 24;6(3):e1000048. doi: 10.1371/journal.pmed.1000048 (PMC2659708; doi:10.1371/journal.pmed.1000048)
Supplement: Supplementary file 9 [file pmed.1000048.sd009.doc]

**Protocol S4. Model Validation Procedures**

Assessing the plausibility of the model output is essential for reliable interpretation of the mapped output. Many different measures of map uncertainty are available. Three different aspects of the performance of the predictive model were assessed using a range of validation statistics as described in the main text. This section describes in more detail the procedures used to define a validation set, obtain validation data, and compute a series of summary validation statistics and plots. This supplement also provides information on additional types of uncertainty quantification and provides some discussion on how they should be interpreted.

**S4.1 Creation of the Validation Sets**

Validation statistics obtained via prediction of a validation set are representative of model performance only if the validation set itself is a representative sample of the prediction space. Visual examination of the *Pf*PR_2-10_ point data used in this study revealed clear evidence of spatial clustering (Figure 1, main text). As such, a simple random sample drawn from this set would be similarly clustered and not spatially representative of the predicted *Pf*PR_2-10_ surface as a whole. To generate a spatially representative validation set, the full set of 7,953 data locations was stratified into the three global regions as defined in the main text (America, Africa+ and CSE Asia) and a spatially declustered sampling procedure was implemented within each region. Thiessen polygons were defined around each data location within each region. A Thiessen polygon defines the area closest to each data point in Euclidian space relative to surrounding points. Each datum was then assigned a weight defined as where is the area of the Thiessen polygon surrounding the data location, . A sample of size was drawn without replacement from the regional set where each datum had a probability of selection proportional to its weight . Those surveys located outside the stable limits of transmission were excluded from selection. The sample size was defined as the larger of = 30 or 10% of the total data within each region, rounded to the nearest integer value, where 30 was chosen as a rule-of-thumb value for an acceptable minimum sample size.

Hold-out sets of size = 30, = 531, and = 239 were defined in this way for the America, Africa+, and CSE Asia regions respectively. The model was then run in full for each region independently using the corresponding thinned sets of = 231, = 4,776, and = 2,146 data to predict *Pf*PR_2-10_ at the validation locations. In contrast to the main model run in which *Pf*PR_2-10_ was predicted as an annual mean for 2007, the validation run predicted values for the time corresponding to the mid-point of each validation survey to enable fairer comparisons of the observed and predicted *Pf*PR_2-10_ values.

**S4.2 Procedures for Testing Model Performance**

Predictive performance of the model was tested using three different approaches: the ability of the model to (i) predict the correct endemicity class at unsampled locations; (ii) predict point-values of *Pf*PR_2-10_ at unsampled locations; and (iii) provide realistic measure of uncertainty for each prediction.

Predicting Endemicity Class

The accuracy of predicting class membership was determined in terms of sensitivity and specificity using the area under curve (AUC) of a receiver-operating characteristic (ROC) curve [1,2,3,4]. These statistics provide a summary of model performance across a range of sensitivity and specificity values, with values of AUC=1 representing a perfect model and AUC=0.5 a model with no discriminatory power. ROC plots and AUC statistics were computed for each of the three endemicity classes *Pf*PR_2-10_ ≤5 %, *Pf*PR_2-10_ >5 % - <40%, and *Pf*PR_2-10_ ≥40%. Benchmarks for interpreting AUC values are inherently arbitrary [5], but values exceeding 0.7 are commonly recognised as representing fair to good discrimination, and values exceeding 0.9 as representing excellent discrimination. Simpler statistics relating to the prediction of endemicity class were also computed: the percentage of points assigned to the correct endemicity class, the percentage of points incorrectly assigned to a non-adjacent class, that is, points in the *Pf*PR_2-10_ ≤5 % class assigned as *Pf*PR_2-10_ ≥40% or vice versa, as well as a full 3 × 3 class contingency table.

Predicting Point Values of *Pf*PR_2-10_

The validation procedure generated = 800 point estimates of *Pf*PR_2-10_, where point estimates were calculated using the mean of each predicted posterior distribution. This set of point estimates (where the asterisk denotes a prediction) was then compared to the corresponding set of known *Pf*PR_2-10_ values at the validation locations. The ability of the model to predict point-values of *Pf*PR_2-10_ at unsampled locations was quantified using three simple summary statistics: the correlation coefficient between the predicted and actual set, the mean prediction error (ME) defined as:

,	(S4.1)

and the mean absolute prediction error (MAE) defined as:

(S4.2)

The correlation coefficient provides a straightforward measure of linear association between the data and prediction sets, the ME provides a measure of the bias of the predictor (the overall tendency to over or under predict *Pf*PR_2-10_ values), and the MAE provides a measure of the mean accuracy of individual predictions (the average magnitude of difference between each actual and predicted value). ME and MAE values were presented as both absolute values and as a proportion of the mean *Pf*PR_2-10_ in each region as calculated from the validation set. A scatter plot was also generated as a visualisation of the correspondence between point estimates of *Pf*PR_2-10_ and the corresponding known values.

A sample semi-variogram was calculated from standardised model residuals to assess the presence of residual spatial autocorrelation unexplained by the model output. Standardised Pearson [6] residuals were defined for each validation location as:

(S4.3)

where is the number of individuals surveyed in survey , is the age-standardized number of *P. falciparum* positive responses in that survey and is the corresponding point-prediction of *Pf*PR_2-10_. This standardisation follows established procedures [7,8] and rescales the raw model residuals to account for their variance characteristics as proportion values. Following the procedure outlined by Diggle and Ribeiro [9] this sample semi-variogram was compared to a Monte Carlo envelope computed from 99 random permutations of the same residual set. This envelope represents the range of semi-variograms that could be expected by chance in the absence of any spatial structure. Where the semi-variogram of interest lies entirely within this envelope, it can be considered to display no significant spatial structure.

Providing Realistic Measures of Uncertainty for Each Prediction

Posterior distributions arising from Bayesian models provide an estimate of the relative probability of a particular outcome and can be used to characterize uncertainty of prediction [10]. Our model generated a posterior distribution for each unsampled location and a procedure [11,12,13] was implemented to test how well the validation set of 800 posterior distributions captured the true uncertainty in our model output. Each such distribution provided a prediction of the probability with which *Pf*PR_2-10_ exceeded any given value in the range 0-100%. The distribution of each validation survey was discretized into 101 probability thresholds, that is, the set of *Pf*PR_2-10_ values that were predicted to exceed the known *Pf*PR_2-10_ value with a probability of . Consider any one of these probability thresholds. The value , for example, denotes the value of *Pf*PR_2-10_ at location that was predicted to have a probability of of exceeding the known value . The value could be defined at each of the 800 validation locations and, at each, compared to the corresponding known value . If the value of was predicted correctly at all 800 validation locations, then 95% of these values would be expected to exceed the corresponding known *Pf*PR_2-10_ value whilst 5% would be smaller. This rationale can be extended such that, for any given probability threshold , the proportion of the 800 values that exceed their corresponding known value should equal the probability threshold . The proportion is also termed the coverage probability [11]. In a perfect model of uncertainty, values of the coverage probabilities would correspond exactly to the probability thresholds and the P-P plot comparing with would show values lying perfectly along the 1:1 line.

**S4.3 Additional Results**

A contingency matrix was generated (Table S4.1) showing, for each region, the numbers of validation points that were classified into each of the three endemicity classes in relation to their known class. For all regions, the majority of points were correctly classified as shown by the shaded cells along the diagonal of each matrix. Classification errors were proportionally smaller for the highest and lowest endemicity class. Globally, 16% (50/308) of validation points from the ≤5% class were wrongly classified as being in the 5-40% class and 0.6% (2/308) in the ≥40% class. Four percent (7/172) of validation points from the ≥40% class were wrongly classified as being in the ≤5% class and 22% (37/172) in the 5-40% class. Errors were larger for the middle 5-40% class in which globally 25% (81/320) were wrongly classified in the ≤5% class and 18% (57/320) in the ≥40% class.

|  | America | | | Africa+ | | | CSE Asia | | |
| --- | --- | --- | --- | --- | --- | --- | --- | --- | --- |
|  | ≤5% | 5-40% | ≥40% | ≤5% | 5-40% | ≥40% | ≤5% | 5-40% | ≥40% |
| ≤5% | 24 | 2 | 0 | 143 | 30 | 1 | 89 | 18 | 1 |
| 5-40% | 4 | 0 | 0 | 44 | 109 | 50 | 33 | 73 | 7 |
| ≥40% | 0 | 0 | 0 | 2 | 29 | 123 | 5 | 8 | 5 |

Table S4.1. Contingency table for the America, Africa+ and CSE Asia regions comparing the observed (rows) endemicity classes of the validation surveys with those class memberships predicted (columns) by the model. If the prediction was perfect all numbers would appear in the grey highlighted cells.

Further maps illustrating the uncertainty of the predictions are also provided. In Figure S4.1 the predicted probability of *Pf*PR_2-10_ falling in each endemicity class is shown. Note the use of a probability scale from zero to one differs from the one third (0.333) to one (1.000) scale used in Figure 5A of the main text. This difference arises because the latter plot displays only probabilities of membership to the most likely class which, by definition must have a probability in excess of one third. In Figure S4.2, the standard deviation of each per-pixel posterior distribution in units of *Pf*PR_2-10_ is provided as an additional index of relative uncertainty.

Figure S4.3 presents a combined visualisation of the *Pf*PR_2-10_ endemicity class assignments (presented in Figure 4 of the main text) and the certainty of those class assignments (presented in Figure 5A of the main text). Each of the three classes has a different primary colour associated and the shading for each pixel is derived as a composite of these three colours in proportion to the probability of membership to each class. Pure shades therefore represent highly certain class assignments (the membership probability to one class was very high and to the other two very low), whilst mixed shades represent less certain assignments (membership probability was shared more evenly across two or three classes).

Figure S4.1. The predicted probability of *Pf*PR_2-10_ falling in each endemicity class: A *Pf*PR_2-10_ ≤5%; B *Pf*PR_2-10_ >5%-<40%; C *Pf*PR_2-10_ ≥40% within the stable limits of *P. falciparum* transmission. Yellow values indicate a small probability of class membership, and blue a large probability. Dark grey areas indicate the limits of unstable risk (*Pf*API <0.1 per thousand people pa) and pale grey no risk (*Pf*API = 0 per thousand people pa) [14,15,16].

Figure S4.2. The standard deviation (in units of *Pf*PR_2-10_, 0-100%) of each per-pixel posterior distribution within the stable limits of *P. falciparum* transmission. These values act as an index of relative uncertainty. Where predictions were made with large uncertainty, the resulting posterior distributions were dispersed across a wide range of possible values of *Pf*PR_2-10_ so the standard deviation of these values was relatively high. Conversely, where uncertainty was small, posterior distributions were well defined across a narrow range of values resulting in low standard deviations. Dark grey areas indicate the limits of unstable risk (*Pf*API <0.1 per thousand people pa) and pale grey no risk (*Pf*API = 0 per thousand people pa) [14,15,16].

Figure S4.3. Three-colour composite showing combined probability of membership to three classes of *Pf*PR_2-10_ endemicity categorized as low risk *Pf*PR_2-10_ ≤5% (pure blue); intermediate risk *Pf*PR_2-10_ >5%-<40% (pure green) and high risk *Pf*PR_2-10_ ≥40% (pure red). The shading for each pixel is derived as a composite of the three pure class colours in proportion to the probability of membership to each class. Thus, pure shades indicate a highly certain assignment to a single class whilst mixed intermediate shades indicate an uncertain assignment with membership probability shared across two or three classes. Dark grey areas indicate the limits of unstable risk (*Pf*API <0.1 per thousand people pa) and pale grey no risk (*Pf*API = 0 per thousand people pa) [14,15,16].

**References**

1. Brooker S, Hay SI, Bundy DA (2002) Tools from ecology: useful for evaluating infection risk models? Trends Parasitol 18: 70-74.

2. Clements ACA, Lwambo NJS, Blair L, Nyandindi U, Kaatano G, et al. (2006) Bayesian spatial analysis and disease mapping: tools to enhance planning and implementation of a schistosomiasis control programme in Tanzania. Trop Med Int Health 11: 490-503.

3. Fawcett T (2006) An introduction to ROC analysis. Pattern Recognition Lett 27: 861-874.

4. Metz CE (1978) Basic principles of ROC analysis. Semin Nucl Med 8: 283-298.

5. Conraths FJ, Schares G (2006) Validation of molecular-diagnostic techniques in the parasitological laboratory. Vet Parasitol 136: 91-98.

6. McCullagh P, Nelder JA (1989) Generalized Linear Models. Boca Raton, Florida: Chapman and Hall / CRC Press. 540 p.

7. Clements ACA, Moyeed R, Brooker S (2006) Bayesian geostatistical prediction of the intensity of infection with *Schistosoma mansoni* in East Africa. Parasitology 133: 711-719.

8. Diggle P, Moyeed R, Rowlingson B, Thomson M (2002) Childhood malaria in The Gambia: a case-study in model-based geostatistics. J Roy Stat Soc C-App 51: 493-506.

9. Diggle PJ, Ribeiro PJ (2007) Model-based geostatistics; Bickel P, Diggle P, Fienberg S, Gather U, Olkin I et al., editors. New York: Springer. 228 p.

10. Congdon P (2003) Applied Bayesian Modelling. Chichester: John Wiley and Sons Ltd. 457 p.

11. Gething PW, Noor AM, Gikandi PW, Hay SI, Nixon MS, et al. (2008) Developing geostatistical space-time models to predict outpatient treatment burdens from incomplete national data. Geogr Anal 40: 167-188.

12. Goovaerts P (2001) Geostatistical modelling of uncertainty in soil science. Geoderma 103: 3-26.

13. Moyeed RA, Papritz A (2002) An empirical comparison of kriging methods for nonlinear spatial point prediction. Math Geol 34: 365-386.

14. Hay SI, Smith DL, Snow RW (2008) Measuring malaria endemicity from intense to interrupted transmission. Lancet Infect Dis 8: 369-378.

15. Guerra CA (2007) Mapping the contemporary global distribution limits of malaria using empirical data and expert opinion [Doctoral thesis]. Oxford: University of Oxford. 258 p.

16. Guerra CA, Gikandi PW, Tatem AJ, Noor AM, Smith DL, et al. (2008) The limits and intensity of *Plasmodium falciparum* transmission: implications for malaria control and elimination worldwide. PLoS Med 5: e38.
